# A Multicomponent Reaction-Based Platform Opens New Avenues in Aryl Hydrocarbon Receptor Modulation

**DOI:** 10.1021/acscentsci.5c00194

**Published:** 2025-04-10

**Authors:** Pau Nadal Rodríguez, Frederick Hartung, Marina Pedrola, Seemon Coomar, Alejandro Diaz-Moreno, Anna M. Hätälä, Katharina M. Rolfes, Ismael Sánchez-Vera, Joan Gil, Elies Molins, Antonio Viayna, Alexander Hanzl, Nicolas H. Thomä, Thomas Haarmann-Stemmann, F. Javier Luque, Rodolfo Lavilla, Ouldouz Ghashghaei

**Affiliations:** † Laboratory of Medicinal Chemistry, Faculty of Pharmacy and Food Sciences and Institute of Biomedicine (IBUB), Universitat de Barcelona, Av. Joan XXIII 27-31, 08028 Barcelona, Spain; ‡ IUF Leibniz Research Institute for Environmental Medicine, 40225 Düsseldorf, Germany; § Friedrich Miescher Institute for Biomedical Research, Fabrikstrasse 24, Basel 4056, Switzerland; ∥ Departament de Ciències Fisiològiques, Facultat de Medicina i Ciències de la Salut, Universitat de Barcelona, Institut d’Investigació Biomèdica de Bellvitge (IDIBELL), L’Hospitalet de Llobregat, 08907 Barcelona, Spain; ⊥ Institut de Ciència de Materials de Barcelona (ICMAB-CSIC), Campus UAB, E-08193 Cerdanyola, Spain; # Department of Nutrition, Food Science and Gastronomy, Faculty of Pharmacy and Food Sciences, Institute of Biomedicine (IBUB) and Institute of Theoretical and Computational Chemistry (IQTC-UB), Universitat de Barcelona, Av. Prat de la Riba 171, 08921 Santa Coloma de Gramenet, Spain; ∇ Swiss Institute for Experimental Cancer Research (ISREC), EPFL, Station 19, Lausanne 1015, Switzerland

## Abstract

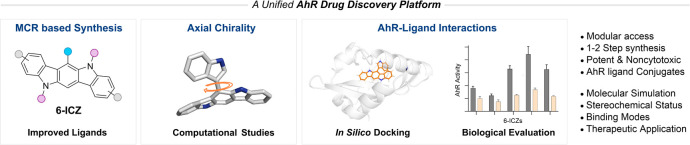

A multidisciplinary platform is presented to address aryl hydrocarbon receptor (AhR) modulation. A rewired Yonemitsu multicomponent reaction with indole 2-carboxaldehydes and nucleophilic species was designed to access a family of 6-substituted indolocarbazoles. The conformational behavior of these compounds was examined to rationalize their axial chirality. *In silico* docking and molecular simulations highlighted key features implicated in their binding to AhR. Furthermore, the synthesis of linkable derivatives allowed the direct development of conjugated entities. Reporter gene and target gene expression analyses identified these novel structures as potent noncytotoxic activating AhR ligands, that can be extended to bifunctional molecules. The anti-inflammatory properties of these AhR agonists were assessed in interleukin-13 treated keratinocytes. Altogether, the synergistic research in synthetic and computational chemistry integrated with biological studies opens novel avenues toward understanding the biological roles of AhR and the development of targeted therapeutics.

## Abstract 2

A multicomponent reaction platform yields 6-substituted indolocarbazoles as potent, safe, and modular activators of the Aryl hydrocarbon Receptor (AhR), opening new avenues in AhR research.

## Introduction

**Figure 1:**
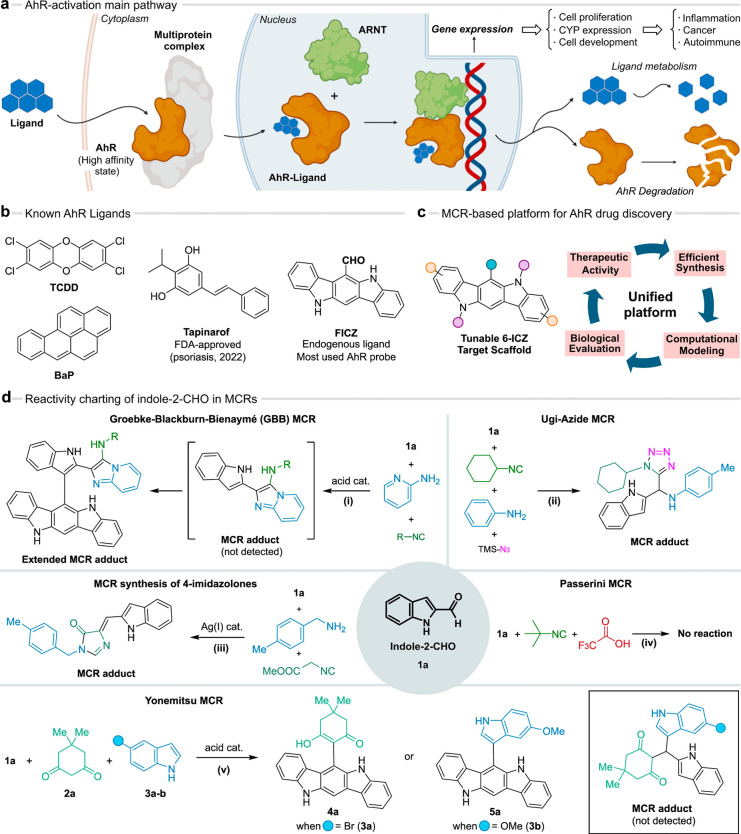
AhR-based drug discovery. (a) Main signaling pathway of AhR after ligand activation. (b) Known AhR ligands. (c) This work: a unified platform for AhR-based drug discovery. (d) The role of indole-2-CHO in various MCRs. Representative reaction conditions: (i) Yb(OTf)_3_·H_2_O (20 mol %), CH_3_CN, rt, 72 h; (ii) MeOH, rt, 8 h; (iii) AgNO_3_ (10 mol %), MeOH, rt, 17 h; (iv) THF, reflux, 17 h; (v) l-proline (10 mol %), EtOH, 80 °C, 4 h. See Figure S1 for detailed reaction conditions.

The aryl hydrocarbon receptor (AhR) belongs to the basic helix–loop–helix/PER–ARNT–SIM (bHLH/PAS) family of transcription factors. It is involved in various biological processes including xenobiotic metabolism and immune responses. In a simplified view, AhR is embedded in a cytosolic multiprotein complex which stabilizes AhR in a state poised for response to both endo- and exogenous ligands.^1^ Upon binding, the AhR-ligand complex translocates into the nucleus, where it interacts with AhR nuclear translocator (ARNT). The formed heterodimer regulates the expression of target genes, particularly encoding cytochrome P450 (*CYP1A1* and *CYP1B1*) and pro- and anti-inflammatory cytokines. Following gene transactivation, AhR is exported from the nucleus and degraded by natural cellular machinery, among others the proteasome. Through this main signaling pathway, AhR plays key roles in xenobiotic metabolism, cell proliferation and differentiation, immune response regulation, etc.^2^ As such, AhR modulation stands as a promising strategy against several autoimmune and inflammatory diseases, cancers, and viral infections (Figure 1a).^3−6^

Nevertheless, AhR-based drug discovery faces several challenges. The often-multifaceted outcome of AhR modulation depends not only on the physicochemical properties of the ligands^7,8^ but also on the cellular context. Hence, a holistic understanding of the complex AhR pathways and their cellular crosstalk is currently still lacking despite numerous snapshots of its functions under diverse physiological conditions. Therefore, AhR modulators with a precise, disease-tailored impact could significantly advance our grasp on the biological mechanisms at play, as well as future drug development efforts. However, many known AhR ligands are polycyclic aromatic hydrocarbons that are difficult to synthesize and/or derivatize, thus offering limited structural diversity.^9^ Moreover, the majority of these ligands raise serious toxicity concerns, such as 2,3,7,8-tetrachlorodibenzodioxin (TCDD)^10^ and benzo[*a*]pyrene (BaP, Figure 1b).^11^ Finally, AhR-based drug discovery has mainly relied on functional screening of compound libraries. In recent years, thanks to the recent advances in AhR structural biology,^12−16^ relevant reports have tackled the development of improved ligands via a combined biological screening approach and structure-guided rational design.^17−19^ Nevertheless, AhR-based therapies are still underdeveloped, and the bacterial metabolite tapinarof is the only FDA-approved AhR ligand to date (psoriasis treatment, 2022, Figure 1b).^20^ Together with the complex signaling pathways of AhR, these challenges illustrate the need to develop probes and modulators to better understand the underlying mechanisms of AhR biology and to trigger AhR-based drug discovery programs. In this context, the 6-formylindolo[3,2-*b*]carbazole (FICZ, Figure 1b) is an endogenous, highly potent, and selective AhR agonist.^21^ FICZ is commercially available and is widely used as an AhR probe. However, its unsuitable drug-like features (mainly attributable to its CHO group), long synthesis, and limited options for structural diversification^22^ restrict its therapeutic projection. Arguably, FICZ analogues, i.e. 6-substituted indolo[3,2-*b*]carbazole (6-ICZ), could become the prime scaffold for an AhR-based drug discovery campaign. This would require modular, yet short and preparative access to 6-ICZ derivatives. Incidentally, the 6-ICZ unit is an attractive pseudonatural skeleton,^23^ with relevant applications in material sciences.^24^ Nevertheless, in contrast to their symmetrically disubstituted 6,12-ICZ counterparts, a reliable synthetic approach to the 6-ICZ scaffold remains almost unexplored.^25−27^

Multicomponent Reactions (MCRs) are one-pot transformations consisting of three or more substrates that yield a single adduct through a unified mechanism. They offer remarkable capabilities to build diverse and modular chemical libraries, particularly appealing for medicinal chemistry.^28−30^ Here we present a unified platform in which an MCR synthetic framework yields tunable 6-ICZs as potent and nontoxic AhR ligands, providing insights into their binding modes, biological validation, and therapeutic potential, to address the aforementioned challenges (Figure 1c).

## Results and Discussion

### Indole 2-Carboxaldehydes Are Unique Substrates in MCRs

We recently reported that the Groebke–Blackburn–Bienaymé (GBB) MCR with indole-2-carboxaldehyde (indole-2-CHO) yields 6-ICZ adducts in an extended fashion (Figure 1d).^31^ Extended MCRs refer to processes where the initial MCR adduct keeps reacting intra- or intermolecularly to attain complex connectivities, enhancing the synthetic reach of these transformations. Particularly interesting is the dual role of indole aldehydes in these domino processes,^32^ as they can first act as electrophiles and, after the initial MCR event, become nucleophilic partners. In this way, we synthesized a family of compounds that were found to be potent AhR activators, albeit with unsuitable features for medicinal chemistry purposes.^31^

We speculated that charting the reactivity of indole 2-CHO in other MCRs may provide relevant mechanistic insights and expand the synthetic reach of these processes.^33^ Previous reports indicated that the Povarov MCR generates simple MCR adducts.^34^ Similarly, we found that the Ugi-Azide MCR and a newly described process by the group^35^ did not undergo extended pathways and only yielded the standard MCR adducts. Moreover, the Passerini MCR was not productive under tested conditions (Figure 1d and Figure S1). Notably, when we turned to the Yonemitsu MCR, which combines aldehydes, 1,3-dicarbonyls, and indoles,^36,37^ neither the MCR product nor the extended adduct was detected. Instead, we observed a rewired^38^ multicomponent process leading to the formation of 6-ICZ adducts **4a** and **5a** (Figure 1d). These results suggested that we could develop efficient and modular access to simple and tunable 6-ICZ derivatives, suitable as potential AhR ligands.

### A Rewired MCR Provides Tunable Access to 6-ICZs

**Figure 2:**
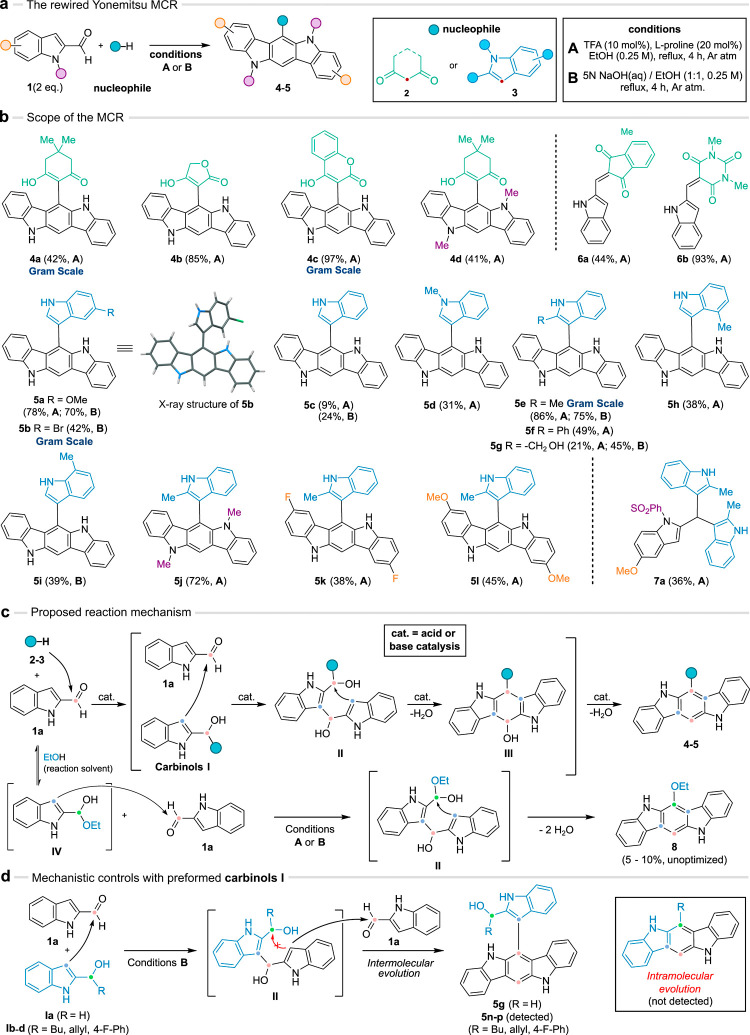
The rewired Yonemitsu MCR: scope and mechanistic insights. (a) The optimized ABB′ process and the standard reaction conditions. (b) The scope of the process. (c) Proposed reaction mechanism and competitive pathway with the reaction solvent leading to compound **8**. (d) Reaction with equimolar amounts of indole-2-CHO **1a** and preformed carbinols **Ia**–**d**.

The selective formation of compound **4a** or **5a** indicated a competitive incorporation of the incoming species depending on their relative nucleophilicity,^39,40^ suggesting that a variety of nucleophiles could participate in the MCR. Thus, we designed and optimized a new ABB′ process,^41^ which combines 2 equiv of indole-2-CHO with a nucleophile, through an extensive charting of the reaction space of the MCR.^35^ We found that our MCR is productive under a range of acid and base catalysts, allowing the generation of ICZs with several diversity points in a single step (Figure 2a and Table S1). As the nucleophilic species represents the modular component in the C-6 of the ICZ core, we first studied the scope of the nucleophiles (Table S2). 1,3-Dicarbonyls including dimedone, tetronic acid, and 4-hydroxycoumarin conveniently afforded the adducts **4a**–**d** in good yields (40–97%, Figure 2b). In contrast, less enolizable 1,3-dicarbonyls only yielded the Knoevenagel adducts **6a** (44%) and **6b** [93%, Supporting Information (SI) Section 3.3.3], and acyclic 1,3-dicarbonyls did not participate in the MCR (Table S2). Concerning heterocyclic nucleophiles, a variety of indoles (naked, *N*-, 2-, 4-, 5-, and 7-substituted) consistently gave the expected adducts **5a**–**5l** (9–86%). The structure of **5b** was confirmed by X-ray crystallography (Figure 2b and SI Section 8.1). Interestingly, the MCR with pyrroles resulted in dimeric-type species, which were detected in low amounts (Figure S5). 5-Aminopyrazoles have been recently reported to yield substituted ICZs.^42^ Other nucleophiles, including heterocycles, phenols, isocyanides, cyanide, and nitromethane led to complex mixtures, competitive processes, or unproductive reactions (Table S2 and Figure S8). Furthermore, the ICZ core can be decorated by using substituted indole-2-CHOs. In this way, compounds **5k** (38%) and **5l** (45%) were obtained from electron deficient 5-F and electron rich 5-OMe indole-2-CHO, respectively. Moreover, *N*-substituted 6-ICZs **4d** (40%) and **5j** (72%) were synthesized from the 1-Me derivative. However, the highly deactivated *N*-sulfonyl indole-2-CHO only yielded the tris-indolylmethane **7a** (36%, Figure 2b and Figure S4). Importantly, although indole-3-CHO could in principle generate the expected 6-ICZs **4**-**5**, in this case the reaction only proceeded to compounds **6** or **7**, depending on the nucleophilic species. A preliminary appraisal based on stability terms was consistent with the experimental results (SI Section 3.3.3).^43^

### Mechanistic Studies Reveal Competing Pathways

As for the formation of the 6-ICZs **4** and **5**, we assumed that the mechanism starts with the addition of nucleophiles **2** or **3** to the aldehyde to generate the putative carbinol **I**. Next, the polarity inversion of the indole presumably allows the addition of carbinol **I** to a second unit of indole-2-CHO, ensuing a domino process that includes electrophilic cyclization and final dehydration to generate the 6-ICZs **4**-**5** (Figure 2c). Notably, analogous carbinols typically evolve toward the Knoevenagel pathway via dehydration or to bis/tris-indolylmethanes upon subsequent indole addition.^44−46^ Indeed, we observed the formation of adducts **6** and **7** in some cases, but importantly they did not convert to the respective 6-ICZs **4** and **5** under the tested conditions, suggesting that the two outcomes are independent, supporting the proposed mechanism (Figures S3 and S4). During this study, a competitive incorporation of EtOH (solvent) was observed in many cases leading to small amounts of 6-ethoxy-ICZ **8**, likely generated from the hemiacetal intermediate **IV** (Figure 2c and Figure S11). Lastly, the self-condensation of indole-2-CHO under strong basic conditions afforded the unsubstituted **ICZ** (Figures S6, S7, and S11). For a more detailed discussion of the reaction mechanism, see SI Section 3.3.2.

To further clarify the role of the putative carbinols **I** as key intermediates of the transformation, we attempted its isolation with a variety of nucleophiles. However, the incorporation of the second unit of aldehyde and the ensuing cascade are presumably faster than the first nucleophilic addition, which precluded their detection (SI Section 3.3.1). Thus, we envisioned the reaction of equimolar amounts of indole-2-CHO with the preformed carbinol **Ia**. Interestingly, we only generated the corresponding 6-indolyl-ICZ **5g**, bypassing the alternative intramolecular cyclization (Figure 2d). Other α-substituted carbinols **Ib**–**d** resulted in analogous results, supporting our mechanistic hypothesis (Figure 2d and Figure S9). Interestingly, a precedent work reported the formation of trace amounts of a 6-ICZ adduct from the acid-catalyzed dimerization of an allyl carbinol.^47^

### Post-Transformation Reactions Enrich the Chemical Diversity of the 6-ICZ Scaffold

**Figure 3:**
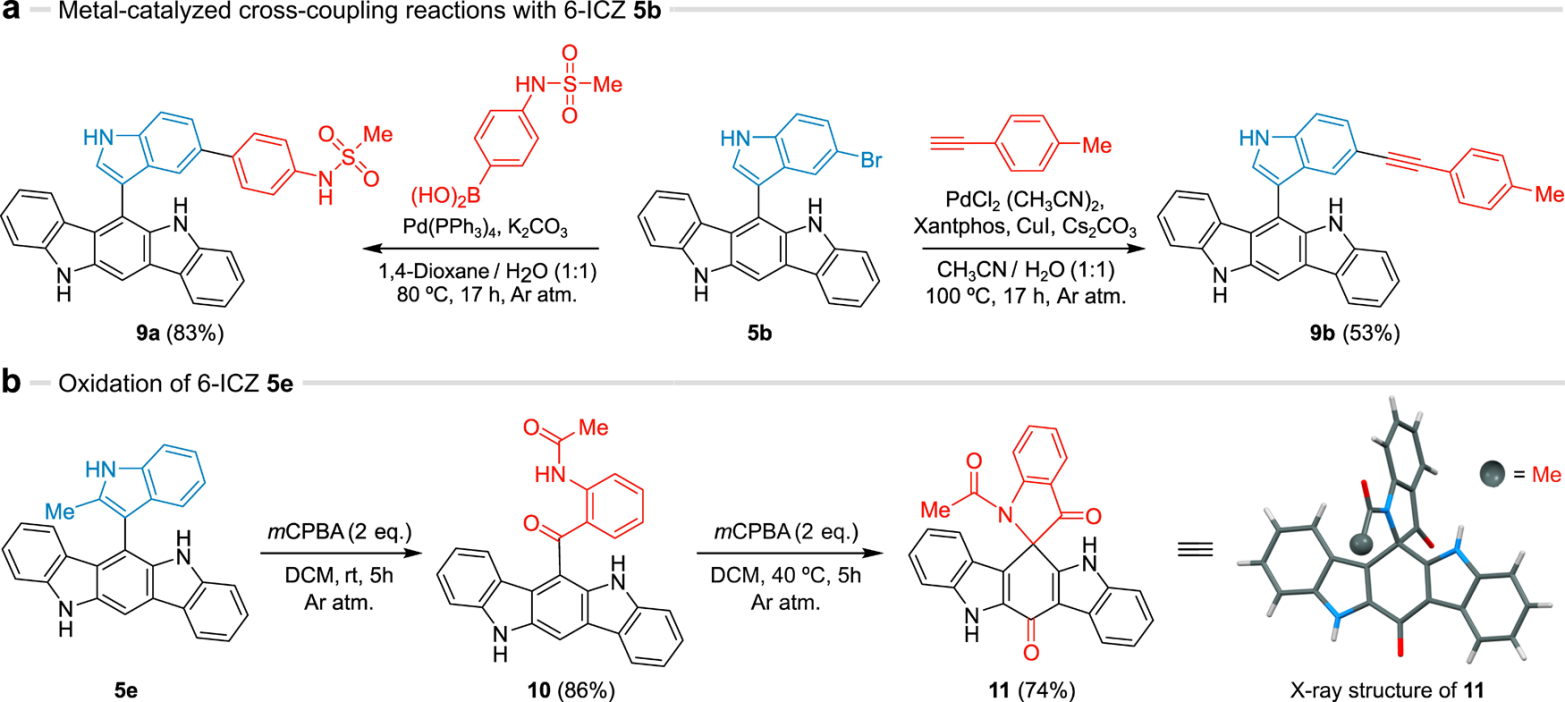
Post-transformation modifications of the 6-ICZs. (a) Metal catalyzed cross-couplings with compound **5b**. (b) Oxidation of compound **5e**.

To expand the chemical diversity of the generated adducts, we envisioned a series of post-transformation modifications. From the bromo adduct **5b** we obtained the indolyl C-5 substituted compounds **9a** (83%) and **9b** (53%) after Suzuki and Sonogashira cross-couplings, respectively (Figure 3a). Next, we were interested in obtaining closer analogues to the endogenous ligand FICZ, bearing a carbonyl motif in the C-6 of the ICZ scaffold. To that end, we considered the oxidative cleavage of the C2–C3 double bond of the indolyl residue in compound **5e** with *m*CPBA.^48^ Under controlled conditions the oxidation was chemoselective to generate ketoamide **10** (86%), avoiding overoxidation of the ICZ core to quinone-type products (Figure 3b). In this context, the subsequent oxidation of compound **10** did not yield the expected Baeyer–Villiger adducts and interestingly afforded the spiro adduct **11** (74%), whose structure was confirmed by X-ray crystallography (Figure 3b and SI Section 8.2).

### Substitution at the Indolyl C-2 Induces Atropoisomerism

**Figure 4:**
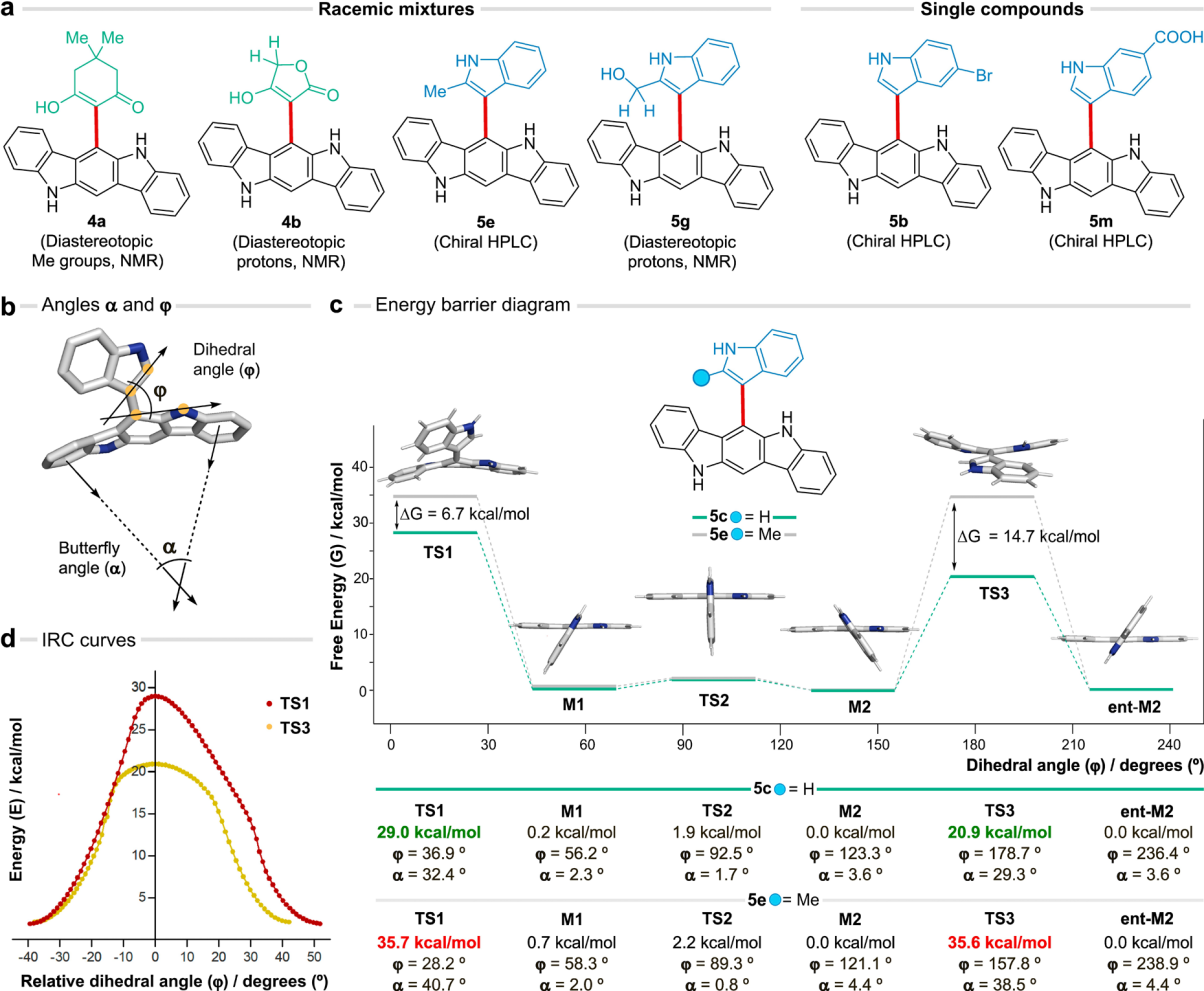
Axial chirality of 6-ICZs. (a) Experimental observations. (b) Definition of the dihedral (ϕ) and butterfly (α) angles. (c) Energy barrier diagram for the ICZ-indolyl axis rotation for compounds **5c** (green) and **5e** (gray). Only the snapshots of **5c** are shown. (d) IRC analysis of **TS1** and **TS3** for compound **5c**.

The stereochemical status of these adducts is a potential concern for therapeutic applications as substituents at the ICZ’s C-6 position may induce axial chirality due to steric clashes preventing full bond rotation.^49−52^ Indeed, the existence of atropoisomers was unambiguously confirmed in **4a**,**b** and **5g** (diastereotopic ^1^H NMR signals) and **5e** (peak splitting in chiral HPLC). However, no evidence was found to support chirality in **5b** and **5m**, featuring 2-*H* indolyl substituents on ICZ, suggesting a free rotation around the ICZ-indolyl axis in case of an unencumbered framework (Figure 4a and SI Section 5.1).

To explore the effect of the indolyl C-2 substitution on the rotational behavior of **5c** (R = H) and **5e** (R = Me), density functional theory (M062X/6-31G(d,p)) calculations were performed. We defined the dihedral (ϕ) and butterfly (α) angles to describe the ICZ-indolyl rotation and the planarity of the ICZ pentacycle, respectively (Figure 4b). Both compounds adopt minimum energy structures (**M**) characterized by the rotation of the indole residues (**M1**, ϕ ≈ 50°; **M2**, ϕ ≈ 122°) relative to the planar ICZ (α < 4.5°, Figure 4c). These values are close to the arrangement found in the X-ray structure of **5b** (ϕ = 122.1°, α = 3.8°, SI Section 8.1). Rotation of the indole residue is hindered by strong steric clashes at dihedral angles ϕ close to 0° and 180°. However, the hindrance is alleviated through the concerted distortion of the ICZ and the indolyl residue in the transition state (**TS**) structures. This involves the bending of the indole toward one face of the molecular plane, accompanied by the synchronous, butterfly like warp of the ICZ toward the opposite face to angles **α** up to 30–40° (Figure 4c). The free energy barriers reflected a marked destabilization of **TS1** and **TS3** for **5e** (gray, ca. 35 kcal/mol) in contrast to **5c** (green, 20–30 kcal/mol). These values reflect the energy strain caused by the bending of ICZ, as noted in previous studies for acenes,^53,54^ and the steric clash of the substitution at the indolyl C-2 with the bent ICZ. Remarkably, the geometry distortion of **5c** in one direction (**TS3**, 20.9 kcal/mol) is more favorable than the reverse sense (**TS1**, 29.0 kcal/mol). The former value, which is in line with the rotational barrier reported for C2-unsubstituted naphthyl indoles,^51^ underscores a preferred pathway for the racemization of **5c**. Importantly, intrinsic reaction coordinate (IRC) calculations confirmed that the proposed TS lead to the minimum energy structures (Figure 4d). Similar trends were observed in the energy profiles determined from MP2 calculations (Figures S22 and S23). Furthermore, the calculated rotational barriers were not notably affected by the solvation of acetonitrile or water (Table S3). Interestingly, variable temperature NMR studies with compound **5g** did not result in the coalescence of the diastereotopic signals up to 150 °C, in agreement with the calculated energy barriers (Figure S24). This behavior opens a new perspective to understand the structural features of 6-ICZs and analogous systems, so far described as permanently flat arrangements.^55−57^ The allowed dynamic bending of these aromatic pentacycles may also increase their suitability in docking events.

### The 6-ICZs Unravel Novel Binding Modes to AhR

**Figure 5:**
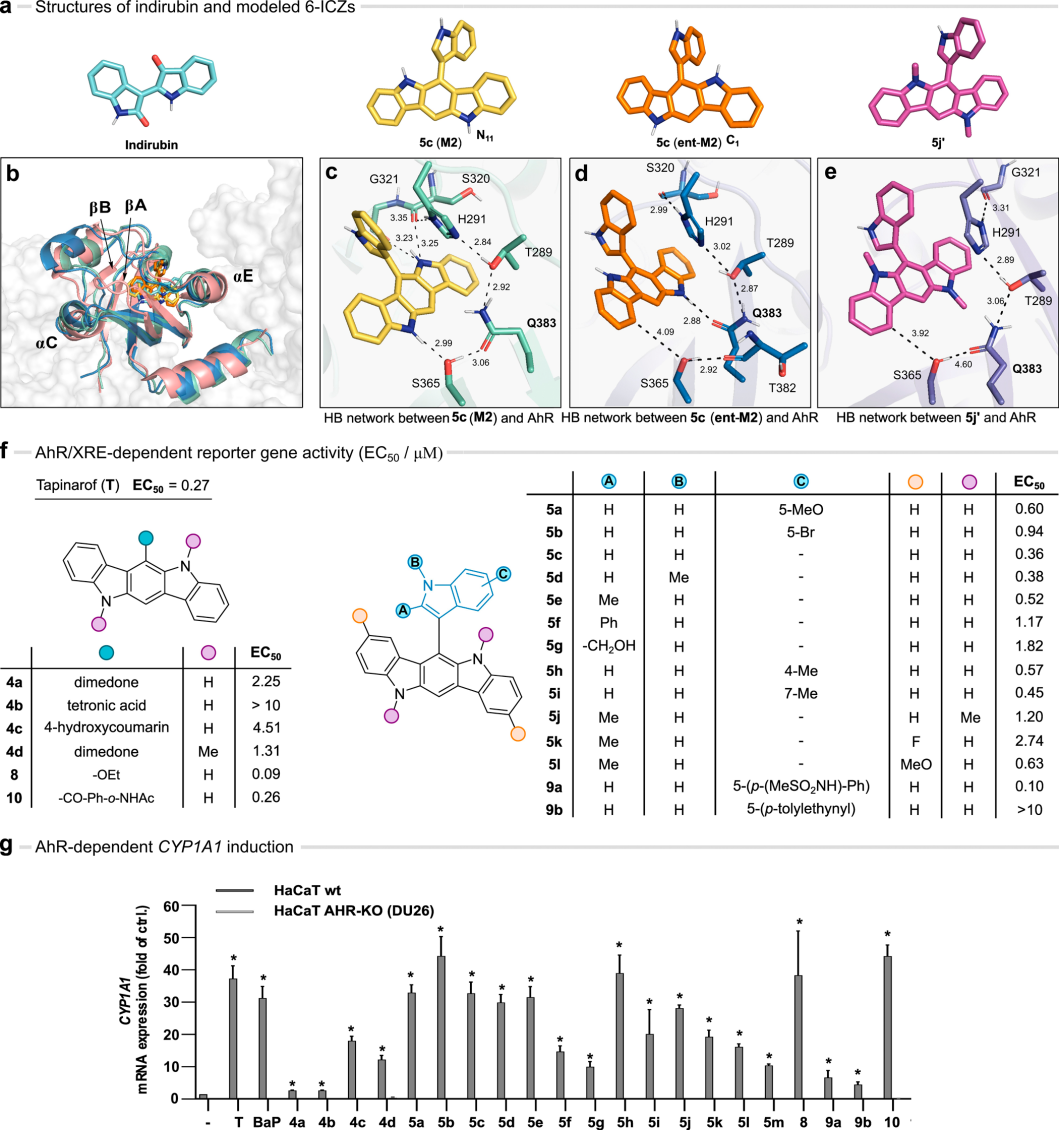
Binding and bioactivity of the 6-ICZs. (a) Molecular structures of indirubin, **5c** (**M2**, **ent-M2**) and **5j′**. (b) Superposition of the cryo-EM structure of the AhR-indirubin complex (ID 7ZUB, light red cartoon) and the last snapshot taken from the MD simulation of AhR with **5c** (**M2**, **ent-M2**: green and blue cartoon). (c–e) Binding modes of **5c M2** (c), **5c ent-M2** (d), and **5j′** (e). The HB network is shown as dashed lines. Distances (Å) denote the average value determined in the last 250 ns of the trajectory (SD varies between 0.2 and 0.4 Å). The conformation of the Q383 side chain is given by torsional angles N–C_α_–C_β_–C_γ_, C_α_–C_β_–C_γ_–C_δ_, and C_β_–C_γ_–C_δ_–N_amide_ of (b) −32, −65, and −179°, (c) 54, −106, and 115°, and (e) 57, −159 and 169°. Labels denote the numbering of residues in the cryo-EM structure. (f) AhR/XRE-dependent reporter gene activity results (EC_50_/μM). (g) AHR-driven *CYP1A1* gene expression, as fold of 0.1% DMSO control (−) in HaCaT wt cells (gray) vs HaCaT AhR-deficient cells (white). 8 h treatment. Concentrations: 6-ICZs and tapinarof (**T**), 3 μM; **BaP**, 2.5 μM. *n* = 3–4. For statistical analysis a two-way ANOVA (Tukey posthoc) was performed and data are shown as mean ± SEM (*, *p* ≤ 0.05).

Molecular simulations were performed to assess the ability of **5c** to mimic FICZ as an AhR binder. First, docking of the isoenergetic conformers **M2** and **ent-M2** (Figure 4c) pointed out that the ICZ moiety can bind to the hydrophobic cavity of AhR, leading to a close overlap with indirubin as found in its cryo-EM complex with AhR (Figure 5a,b and SI Section 6.1).^12^ It is worth noting that the binding of **M2** and **ent-M2** primarily affects the arrangement of the loops located between βA and βB and between helices C and E, while the heteromer interface remains unaltered (Figure 5b and SI Section 6.1). Therefore, the binding of **5c** does not affect the interaction of AhR with the other complex subunits. The pentacycle of compound **5c** fills a highly hydrophobic cavity formed by F287, C303, L308, L315, I325, M340, I349, L353, F69, A367, V381, and I377, and the indolyl residue is located at the mouth of the pocket, where it partially stacks against the imidazole ring of H291. In agreement with previous studies,^14^ the N_11_H unit of **M2** forms a hydrogen bond (HB) with S365, which participates in a HB network with Q383, T289, and H291, in turn interacting with S320 and G321, similarly as observed in the AhR-indirubin cryo-EM complex (Figure 5c and Figure S25).^12^ In contrast, **ent-M2** adopts a pose where the ICZ moiety is rotated ∼180°, enabling the formation of a weak C_1_–H···O HB between ICZ and S365, which is hydrogen-bonded to the amide carbonyl group of Q383 (Figure 5d). Notably, the HB network that bridges S365 and S320 is preserved due to the conformational readjustment of the Q383 side chain, which adopts distinct conformations in the two poses (Figure 5c,d). Molecular Dynamics (MD) simulations supported the structural stability of the two binding modes [RMSD of 2.2 ± 0.1 Å (**M2**) and 1.8 ± 0.2 Å (**ent-M2**), Figure S26]. Finally, it is worth noting that the binding poses of **M2** and **ent-M2** show that the C-6 indolyl substituent is pointing to the bulk solvent. This suggests the feasibility of designing bivalent ligands that may be used to tune the biological activity of the synthesized compounds or to develop suitably modified probes (see below). This computational outcome will ideally deal with the design and calculations of prospective ligands in a fast, accurate, and efficient manner.

### The 6-ICZs are Potent and Noncytotoxic Activators of AhR

On the basis of these results, we examined the AhR-stimulating activity of the prepared 6-ICZs. Almost all the tested compounds increased the AhR/xenobiotic response element (XRE)-dependent reporter gene activity in hepatoma cells in a dose-dependent manner, with several analogues in the sub-μM range, indolyl 6-ICZs **5** being more promising than the 1,3-dicarbonyl counterparts **4** (Figure 5f and Figure S28). Moreover, most 6-ICZ adducts induced the expression of *CYP1A1* and *CYP1B1* in HaCaT keratinocytes. Notably, the activity was AhR-dependent, as no expression was induced in AhR-deficient (DU26) HaCaT cells. In this regard, 6-ICZs **5a**–**e**,**h**, **8**, and **10** were comparable to the reference compounds tapinarof (**T**, 3 μM) and benzo[*a*]pyrene (**BaP**, 2.5 μM, Figure 5f,g and Figure S29). Additionally, compounds **5a**, **5d**, **5e**, and **5k** induced receptor proteolysis in MCF7 cells, an observation consistent with the effects of other known AhR agonists (Figure S30).^58^

While these results support the ability of the binding pocket to accommodate the ICZ pentacycle modified with small-size substituents at positions C-2, C-8, N-5, and N-11, it is striking that the activity of the *N*,*N*′-Me derivative **5j** is reduced by only 2.3-fold compared to its *N*,*N*′-H analog **5e** despite the loss of the HB with Q383 (Figure 5f). In this regard, the cost of losing said HB may be counterbalanced by the gain in stability due to the burial of the methyl group in the hydrophobic cavity,^59^ taking into account that the binding affinity of drug-like compounds is largely driven by hydrophobicity.^60^ Furthermore, MD simulations of the model structure **5j′** pointed out that the *N*,*N*′-Me ICZ binds to the AhR mimicking the pose of **5c** (**ent-M2**), albeit losing the partial overlap with H291 (Figure 5d,e). This is due to the conformational readjustment of the Q383 side chain, which enables the accommodation of the *N*-methyl unit while retaining the HB connection between S365 and H291 (Figure 5e). This may also explain the similar activity of *N*,*N*′-Me adduct **4d** compared to its *N*,*N*′-H counterpart **4a** (Figure 5f,g). Finally, the proposed binding mode supports the slight decrease in activity of the indolyl C-2 substituted derivatives due to the proximity to the side chain of Y322 (Figure 5f,g and Figure S27).

Importantly, most of our 6-ICZs did not induce apoptotic cell death or cytotoxicity at concentrations up to 10 μM, and some compounds were practically noncytotoxic even at 100 μM (Figure S35). Furthermore, we tested the potential phototoxicity of our 6-ICZ derivatives, as FICZ is a nanomolar sensitizer for UVA radiation.^61,62^ In contrast to FICZ, none of the selected compounds showed any signs of phototoxicity at a concentration of 100 nM, similarly to FDA-approved tapinarof. Notably, some of the our most potent 6-ICZs such as compounds **8** and **10** showed minimal overlap between their effective and toxic concentrations, the latter—our closest structural analogue to FICZ—even exhibiting no phototoxicity at 1 μM (Figure S36). Altogether, these results showcase the potential of our synthetic platform to obtain safe and potent AhR activators.

### A Conjugatable AhR-Ligand Provides Access to AhR Probes

Proximity inducing pharmacology through bivalent molecules has emerged as an effective method to modulate protein activity.^63^ The context-dependent protumorigenic and immunosuppressive activities of AhR make it a compelling target for therapeutic intervention. Thus, we hypothesized that bifunctional molecules inhibiting AhR activity could prove exceptionally useful. To this end, we envisaged the design of a conjugatable AhR ligand as a building block for AhR probes. Molecular modeling studies suggested that the indole’s C-6 position is a suitable extension point.

**Figure 6:**
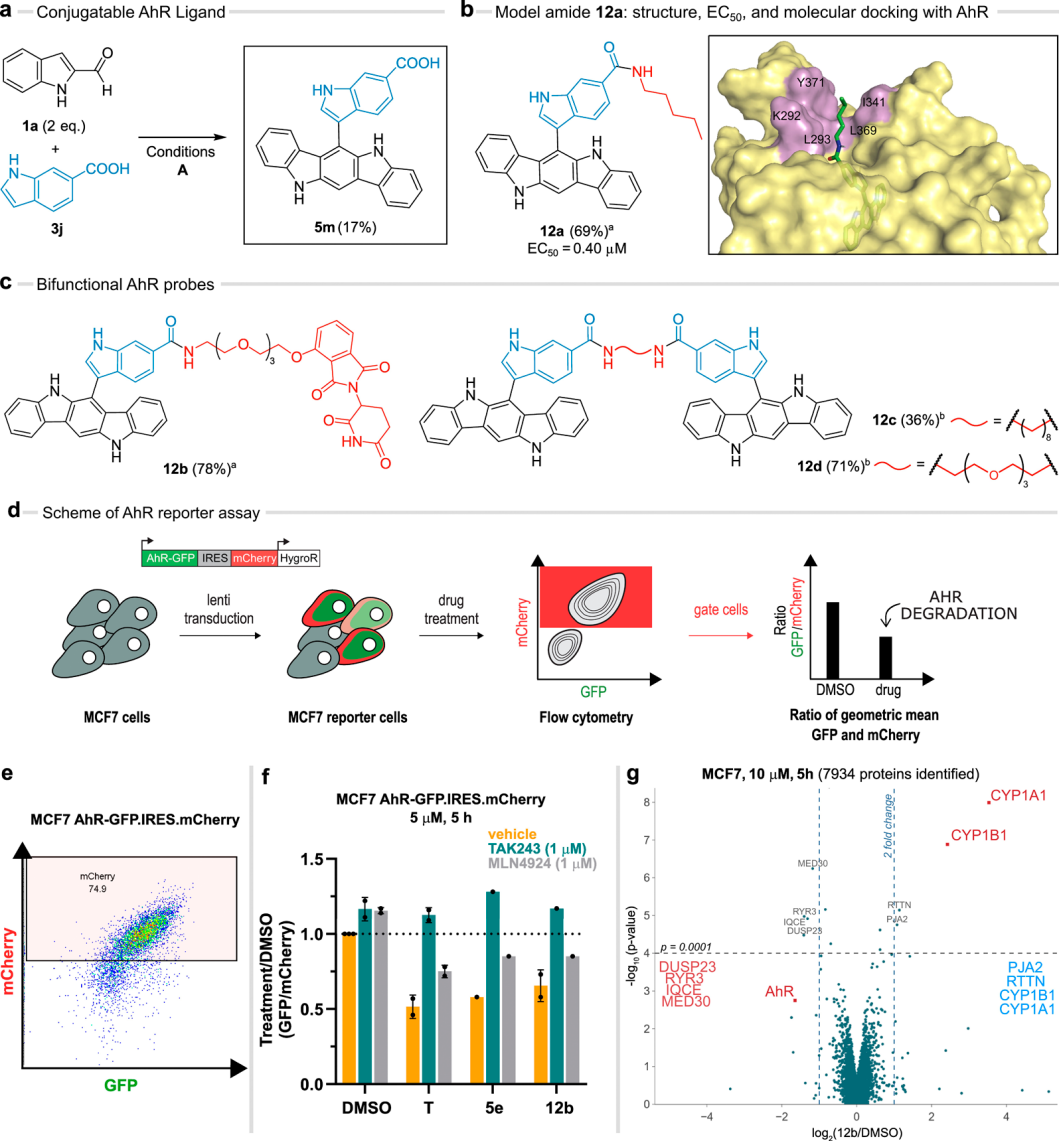
Bifunctional AhR probes and AhR reporter in MCF7 cells. (a) Synthetic access to the conjugatable AhR ligand **5m**. (b) Structure, yield, and EC_50_ value of the model amide **12a** and MD simulation of AhR with **12a** (last snapshot). (c) PROTAC-like compound **12b** and dual probes **12c**–**d**. Reaction conditions: (a) **5m** (1 equiv), amine (1 equiv), HATU, DIPEA, DMF, rt, 24 h, Ar atm; (b) **5m** (2 equiv), diamine (1 equiv), HATU, DIPEA, DMF, rt, 24 h, Ar atm. (d) General setup of the reporter system for quantifying AhR protein levels. (e) The population of MCF7 reporter cells (MCF7 AhR-GFP.IRES.mCherry) gated by flow cytometry and used for quantification of AhR. (f) Change in AhR levels relative to vehicle (DMSO) treatment in the reporter cells treated with tapinarof (**T**), **5e**, and **12b** at a concentration of 5 μM for 5 h, in the presence or absence of TAK243 (1 μM) and MLN4924 (1 μM). Values represent the ratio of the geometric mean of GFP and mCherry values in the gated population. (g) Change in protein levels relative to vehicle treatment (DMSO) in MCF7 cells treated with **12b** at 10 μM for 5 h quantified by TMT labeling and LC/MS analysis versus p-Value.

Thus, we exploited our MCR platform to prepare AhR ligand conjugates. In this regard, indole-6-carboxylic acid **3j** readily generated compound **5m** (17%, unoptimized), which stood out as a linkable 6-ICZ (Figure 6a). We synthesized the model conjugate **12a** (69%) after a conventional amide coupling of **5m** with amylamine. MD simulations confirmed the ability of **12a** to bind to AhR, as the alkyl chain protrudes into the bulk solvent, filling a hydrophobic gorge formed by the apolar residues L293, I341, L369, and Y371 and the methylene chain of K292, without interfering with the AhR heteromeric complex (Figure 6b). Furthermore, the EC_50_ determined for **12a** (0.40 μM) was similar to that determined for **5c** (0.36 μM, Figures 5f and 6b). This computational and experimental evidence suggests considerable engagement with AhR and confirmed the suitability of adduct **5m** as a conjugatable AhR-ligand for the development of bifunctional AhR probes through two different strategies. In a first approach, we linked thalidomide-PEG-amine to **5m** to conveniently obtain **12b** (78%), which bears thalidomide, a ligand of the E3 ubiquitin ligase CRBN.^64^ Such bivalent molecules have been termed Proteolysis Targeting Chimeras (PROTACs) for their ability to induce targeted protein degradation (TPD) in cells.^65,66^ For our second approach, we hypothesized that dual AhR ligands could elicit their inhibitory effect by forming an inactive homodimer and synthesized the bivalent species **12c** (36%) and **12d** (71%) in a single step from unprotected diamine-type linkers (Figure 6c). The modulation of protein function through small molecule induced homodimerization or higher-order oligomerization has been demonstrated to be an effective strategy across various examples.^67−69^

To test the effect of these compounds on AhR abundance, we established a cellular reporter in the breast cancer cell line MCF7. AhR in this case is fused to GFP bearing an independently translated mCherry on the same transcript to allow correction for general transcriptional interference of treatments (Figure 6d,e). In this reporter system, compounds **5e** and **12b** showed pronounced AhR degradation. Cotreatment with an inhibitor of the E1-ubiquitin activating enzyme (TAK243) but not with a cullin E3 ligase specific inhibitor of neddylation (MLN4924) rescued this degradation effect (Figure 6f). Furthermore, we created a reporter cell line with an AhR variant lacking the PasB domain (AhRΔ275-242), which showed no degradation upon compound treatment, underscoring the significance of agonist binding to the pocket (Figure S31). Degradation of endogenous AhR was further confirmed via Western blotting and global proteomics measurements in cells treated with **12b**. These experiments highlighted a selectivity for AhR degradation as well as an induced transcriptional activity, demonstrated by the upregulation of *CYP1A1* and *CYP1B1* (Figure 6g and Figure S32). We could reproduce these effects in orthogonal assays in HaCaT cells, where treatment with **12b**–**d** caused a transient decline of the AhR protein level (Figure S33). At the same time, **12b**–**d** induced AhR/XRE-dependent luciferase activity and *CYP1A1*/*CYP1B1* gene expression (Figures S28 and S29). These results indicated that 6-ICZ conjugates behave as AhR agonists, which typically induce proteolysis of the receptor.^58^ Intriguingly, these results also suggest no CRBN dependent degradation mechanism with the PROTAC molecule **12b**. This is further exemplified by the absence of degradation of common CRBN-thalidomide off-target factors such as zinc finger proteins and GSPT1 (Figure 6g).^70^

Given these biological effects, we believe that the agonist **5m** retains its AhR-directed activity when integrated into bifunctional molecules **12b**–**d**. In HaCat cells we observed the coimmunoprecipitation of CUL4B, an E3 ligase suggested responsible for the degradation of AhR (Figure S34).^71^ At the same time, however, inhibition of cullin activity in MCF-7 cells appeared to have a limited impact on the degradation of AhR when treated with **12b**–**d**. Together, this suggests that different E3 ubiquitin ligases might be responsible for AhR degradation upon activation in different cellular contexts.^72,73^

### Compound **8** Is a Potent Anti-Inflammatory AhR Agonist

**Figure 7:**
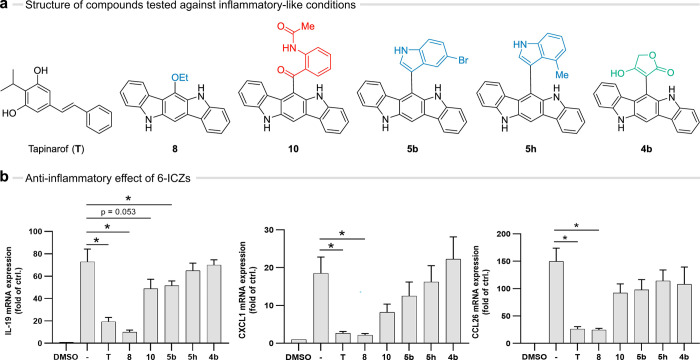
Anti-inflammatory effect of the 6-ICZs. (a) Structure of the tested compounds and the control tapinarof (**T**). (b) IL-13 triggered gene expression of proinflammatory IL-19, CXCL1, and CCL26 expressed as fold of 0.1% DMSO control. HaCaT keratinocytes were treated with IL-13 (10 ng/mL) for 24 h, then either untreated (−) or treated with positive control **T** (1 μM) or 6-ICZs (1 μM). *n* = 3–4. For statistical analysis an unpaired *t*-test was performed and data are shown as mean ± SEM (*, *p* ≤ 0.05 compared to IL-13 (10 ng/mL)).

As AhR activation is beneficial for pathologies including chronic inflammatory skin diseases,^74^ we assessed the potential anti-inflammatory role of our ligands by cotreating HaCaT keratinocytes with the proinflammatory cytokine IL-13 and a selection of 6-ICZs (Figure 7a). A 24 h treatment with IL-13 alone induced the gene expression of the proinflammatory IL-19, CXCL1, and CCL26,^75^ whereas cotreatment with **8** significantly reduced the IL-13-triggered induction of those genes. Notably, compound **8** was slightly more potent than the positive control tapinarof, indicating that our 6-ICZs may potentially serve as scaffolds for the development of novel AhR-targeting anti-inflammatory drugs (Figure 7b).

## Conclusion

To sum up, we have developed novel tools for AhR research through a unified drug discovery platform. A rewired MCR with indole 2-CHOs was designed to generate novel AhR activators. This specific and relevant process enables streamlined synthetic access to a highly valuable scaffold endowed with intrinsic tunability. The conformational dynamics of the 6-ICZ adducts provided a structural basis to understand their axial chirality. Moreover, the MD studies confirmed the binding of these ligands to the AhR adaptative pocket. These studies allowed the rational design of a linkable AhR-ligand and the synthesis of conjugated derivatives. Given their biological effects, we believe that in bifunctional molecules the AhR directed moiety primarily determines the mode-of-action. In distinct cellular contexts we observed further indications of different E3 ligases being responsible for AhR degradation, which highlights the multifaceted cellular response to AhR modulation. Some of our analogues also significantly reduced IL-13 induced inflammation-like conditions in vitro, competing with the current benchmarks in potency. These noncytotoxic, yet potent AhR ligands, which preserve native interactions, could prove valuable as tools to shed light into the varied responses to AhR modulation. Our MCR-based platform efficiently addresses the design, synthesis, and biological evaluation of novel ICZ ligands for AhR drug discovery. We believe that the exploration of the dark chemical space around MCRs could prove useful for addressing other scaffolds and targets.

## Data Availability

Crystallographic data for the structures reported in this article have been deposited at the Cambridge Crystallographic Data Center, under deposition numbers CCDC: 2360557 (**5b**) and CCDC: 2360558 (**11**).

## Supporting Information Available

Coordinates of selected snapshot representative of the binding mode of **5c** (**M2**) to AhR (PDB)
Coordinates of selected snapshot representative of the binding mode of **5c** (**ent-M2**) to AhR (PDB)
Coordinates of selected snapshot representative of the binding mode of **5j′** to AhR (PDB)
Complementary discussions, detailed chemical procedures, characterization data from the chemical compounds, detailed biological experimentation methods, copies of NMR spectra, and X-ray crystallography data (PDF)
Transparent Peer Review report available (PDF)

## Supplementary Material

oc5c00194_si_001.pdb

oc5c00194_si_002.pdb

oc5c00194_si_003.pdb
