# Supplementary material for: A Multicomponent Reaction-Based Platform Opens New Avenues in Aryl Hydrocarbon Receptor Modulation
Source: ACS Cent Sci. 2025 Apr 10;11(4):629–41. doi: 10.1021/acscentsci.5c00194 (PMC12022909; doi:10.1021/acscentsci.5c00194)

Supporting Information for Publication

A Multicomponent Reaction-Based Platform Opens New Avenues in Aryl Hydrocarbon Receptor Modulation

Pau Nadal Rodríguez,[a]+ Frederick Hartung,[b]+ Marina Pedrola,[a] Seemon Coomar,[c] Alejandro Diaz-Morena,[a] Anna M. Hätälä,[b] Katharina M. Rolfes,[b] Ismael Sánchez-Vera,[d] Joan Gil,[d] Elies Molins,[e] Antonio Viayna,[f] Alexander Hanzl,[c] Nicolas H. Thomä,[c,g] Thomas Haarmann-Stemmann,*[b] F. Javier Luque,*[f] Rodolfo Lavilla*[a] and Ouldouz Ghashghaei*[a]

[a] Laboratory of Medicinal Chemistry, Faculty of Pharmacy and Food Sciences and Institute of Biomedicine (IBUB), Universitat de Barcelona. Av. Joan XXIII, 27-31, 08028, Barcelona, Spain.

Email: rlavilla@ub.edu, ghashghaei@ub.edu

[b] IUF Leibniz Research Institute for Environmental Medicine. 40225 Düsseldorf, Germany.

E-mail: Thomas.Haarmann-Stemmann@iuf-duesseldorf.de

[c] Friedrich Miescher Institute for Biomedical Research, Fabrikstrasse 24, Basel 4056, Switzerland.

[d] Departament de Ciències Fisiològiques, Facultat de Medicina i Ciències de la Salut. Universitat de Barcelona. Institut d'Investigació Biomèdica de Bellvitge (IDIBELL). L'Hospitalet de Llobregat, 08907 Barcelona, Spain.

[e] Institut de Ciència de Materials de Barcelona (ICMAB-CSIC). Campus UAB, E-08193 Cerdanyola, Spain.

[f] Department of Nutrition, Food Science and Gastronomy, Faculty of Pharmacy and Food Sciences, Institute of Biomedicine (IBUB) and Institute of Theoretical and Computational Chemistry (IQTC-UB), Universitat de Barcelona. Av. Prat de la Riba 171, 08921 Santa Coloma de Gramenet, Spain.

E-mail: fjluque@ub.edu

[g] Swiss Institute for Experimental Cancer Research (ISREC), EPFL, Station 19, Lausanne 1015, Switzerland.

+ Equal Contribution.

Table of Contents

1. General Information 3
2. Preliminary Results 4
3. Reaction Space Charting 5
   3.1. Reaction Conditions Optimization 5
   3.2. The Reaction Scope 6
      3.2.1. MCR Inputs 6
      3.2.2. Scope Limitations 7
   3.3. Mechanistic Studies 10
      3.3.1. Experimental Observations 10
      3.3.2. Detailed reaction mechanism under acidic and basic conditions 14
      3.3.3. Computational Studies on the Reactivity Trends of Aldehydes 1a-b with 1,3-Dicarbonyls 3 15
4. Experimental Procedures and Characterization Data 18
   4.1. General Synthetic Procedures 18
   4.2. Synthesis and Characterization of Starting Materials 19
   4.3. Characterization Data of Final Adducts 23
      4.3.1. 6-ICZs (Series 4-5) 23
      4.3.2. Knoevenagel Adducts and Bis- / Tris-indolyl Compounds (Series 6-7) 29
      4.3.3. 6-Ethoxy-ICZ (8) 31
      4.3.4. Post-transformation Products (Series 9-11) 31
      4.3.5. Conjuated 6-ICZs and bifunctional AhR probes (Series 12) 33
5. Axial Chirality of 6-ICZs 36
   5.1. Experimental Evidence 36
   5.2. Computational Studies 38
   5.3. Variable Temperature NMR Experiment 43
6. AhR-Modulating Activity 44
   6.1. Computational Modelling 44
   6.2. Biological Assays 46
      6.2.1. Detailed Results 46
      6.2.2. Detailed Methods 50
7. Toxicity Studies 53
   7.1. Cytotoxicity 53
   7.2. Phototoxicity 54
8. X-Ray Crystallography 55
   8.1. Compound 5b 55
   8.2. Compound 11 56
9. References 57
10. Copies of NMR Spectra 59
   10.1. Starting Materials 59
   10.2. 6-ICZs (Series 4-5) 66
   10.3. Knoevenagel Adducts and Bis- / Tris-indolyl Compounds (Series 6-7) 84
   10.4. 6-Ethoxy-ICZ (8) 88
   10.5. Post-transformation products (Series 9-11) 89
   10.6. Conjuated 6-ICZs and bifunctional AhR probes (Series 12) 93
   10.7. Compounds S 97

1. General Information

Organic synthesis: All chemicals were purchased from commercial sources and were used as received unless otherwise stated. All reactions were performed under argon in dried glassware, unless otherwise stated. Microwave-irradiated reactions were carried out using a Biotage Initiator Classic. No unexpected or unusually high safety hazards were encountered. Column chromatographies were carried out on commercial silica gel. Flash column chromatographies were carried out using an Isolera Prime Biotage equipped with dual UV detection over prepacked normal phase silica gel columns (4, 12 and 24 g) or the prepacked reverse phase C18 columns (12g). Thin layer chromatographies (TLC) were done using pre-coated Merk silica gel 60 F254 plates and visualized under UV light at 254 nm and 365 nm. The ^{1}H NMR spectra were recorded on 400 MHz or 600 MHz NMR spectrometers. The ^{13}C NMR spectra were recorded at 100 MHz or 150 MHz. Chemical shifts were reported in ppm(δ) as s (singlet), d (doublet), t (triplet), dd (doublet of doublet), m (multiplet), brs (broad singlet), etc. The residual solvent signals were used as references. HPLC-MS spectra were carried out using the following settings: a) Agilent 1260 Infinity II. The analysis was conducted on a Poroshell 120 EC-C15 (4.6 mm × 50 mm, 2.7 μm) at 40 °C with mobile phase A (H_2O + 0.05% formic acid) and B (ACN + 0.05% formic acid) using a gradient elution and flow rate 0.6 mL/min. The DAD detector was set at 254 or 220 nm, the injection volume was 5 μL and oven temperature was 40 °C. b) Waters 2795 Alliance A ZORBOX Extended-C181 (2.1 mm × 50 mm, 3.5 μm) at 35 °C with mobile phase A (H_2O + 0.05% formic acid) and B (ACN + 0.05% formic acid) using a gradient elution and flow rate 0.7 mL/min. The DAD detector was set to the range of 210-600 nm and the injection volume was 5 μL. The High-Resolution Mass Spectrometry was performed by the Mass Spectrometry and Molecular Characterization Unit from the Scientific and Technological Centers in Universitat de Barcelona (CCiTUB).

Statistics and reproducibility: All experiments were carried out independently, the number of replicates is indicated in the figure captions. Data are shown as mean ± SEM (standard error of the mean). Graph Pad Prism Version 8.1.2 (GraphPad Prism Software, San Diego, California, USA) was used for statistical evaluation. For the comparison of two single groups, an unpaired, two-tailed Student’s t-test was performed. One-way ANOVA (post-hoc: Dunnett’s test) was performed to analyze dose studies. For two groups, a two-way ANOVA (post-hoc: Tukey) was conducted. Statistical differences were considered significant at $p \leq 0.05$ and are indicated by an asterisk (*).

2. Preliminary Results

Figure S1. Reported and preliminary results in our laboratory of MCRs with indole-2-CHO.

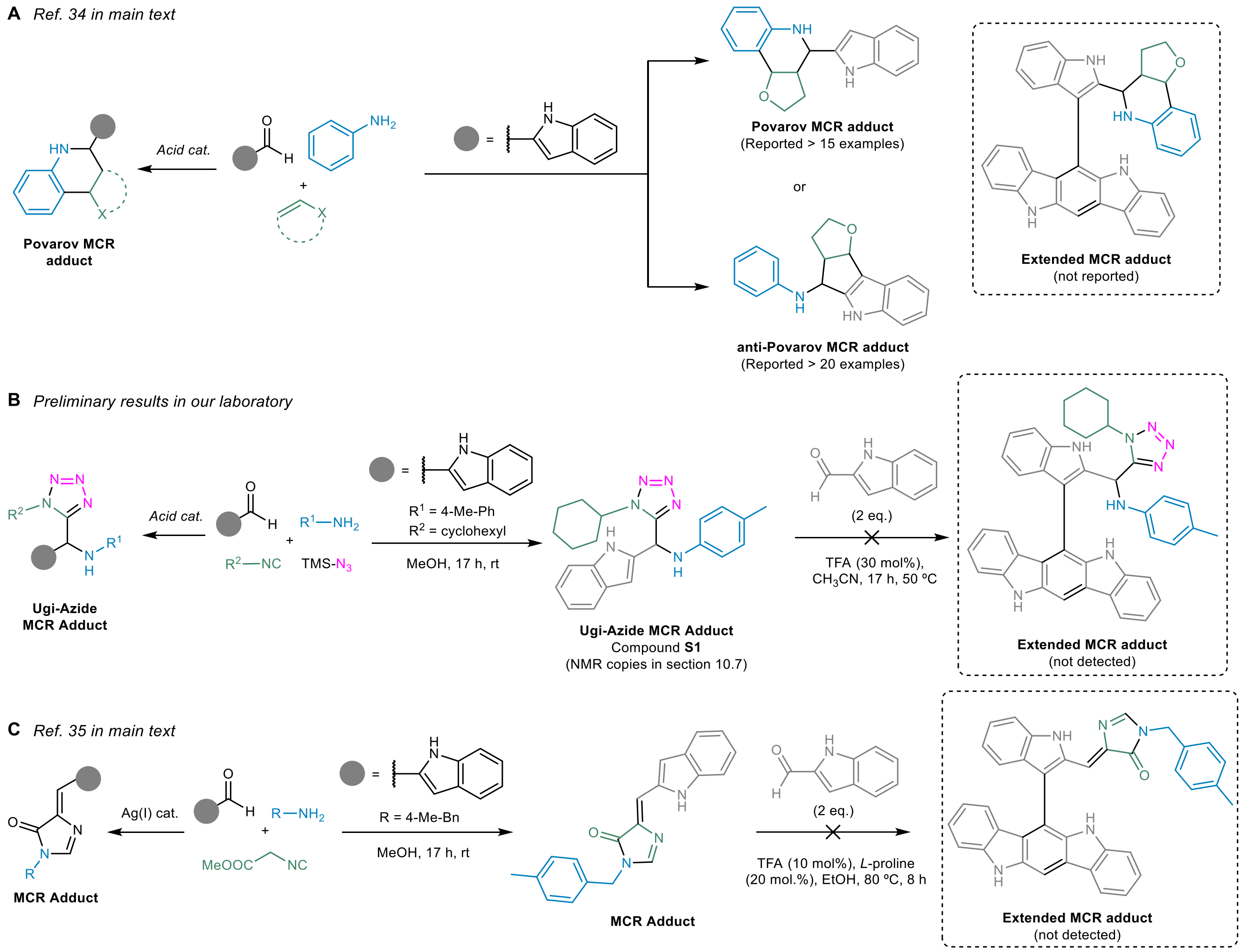

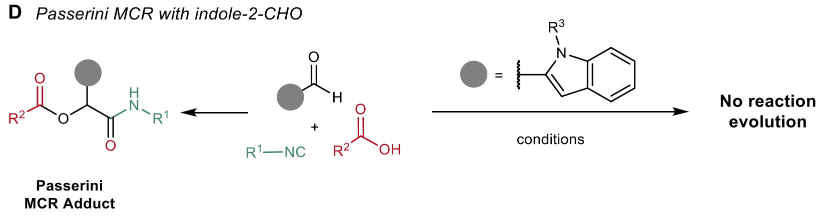

conditions

R^1	R^2	R^3	solvent	temperature	time
t-Bu	CF_3	H	CH_2Cl_2	40 ºC	17 h
t-Bu	CF_3	H	THF	60 ºC	17 h
cyclohexyl	CH_3	H	HFIP	60 ºC	17 h
cyclohexyl	CH_3	Me	HFIP	60 ºC	17 h

3. Reaction Space Charting

3.1. Reaction Conditions Optimization

The reaction conditions for the generation of ICZs **4**-**5** was performed using indole-2-carboxalehyde **1a** and 2-methyl-1*H*-indole **3f**. We started the optimization by performing the MCR under inert atmosphere and TFA catalysis, which afforded the desired 6-ICZ **5e** in a good yield (78%, Table S1, entry A). Moreover, based on bibliographic precedents we considered the use of *L*-proline as a co-catalyst,[1] which increased the productivity (86%, entry B). The reaction did not take place under *L*-proline catalysis without TFA (entry C). Using metal complexes as Lewis Acids and *L*-proline co-catalysis, the reaction also proceeded to the formation of the expected 6-ICZ **5e** with a clean profile and in an acceptable yield (*ca*. 70%, entry D).[2] The MCR also worked in open air, albeit with lower yields (60%, entry E). Finally, the 6-ICZ **5e** was also generated in basic conditions in a similar yield to the acidic ones (entry F, 75%). The best conditions were replicated with dimedone **2a** as the nucleophile (entries G-H).

Conditions in entries B and G were defined as **standard conditions A**, and conditions in entry F were defined as **standard conditions B**, as shown in the main text.

Table S1. Tested reaction conditions.

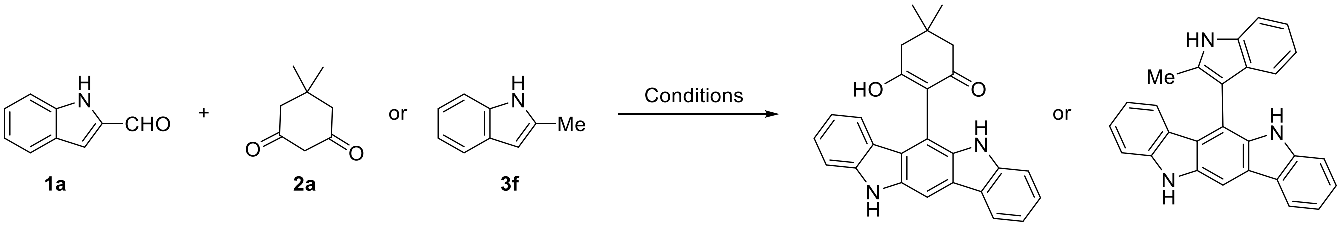

1a **2a** **3f** **4a** **5e**

Entry	Nucleophile	Solvent	Time Temperature	Atm.	Additive (mol %)	Co-catalyst	Product (Yield %)[a]
A	**3f**	EtOH	4 h<br>80 ºC	Argon	TFA<br>(20%)	-	**5e** (78)
B	**3f**	EtOH	4 h<br>80 ºC	Argon	TFA<br>(20%)	L-proline<br>(10%)	**5e** (86)
E	**3f**	EtOH	4 h<br>80 ºC	Argon	-	L-proline<br>(10%)	No conversion
D	**3f**	EtOH	4 h<br>80 ºC	Argon	$M(OTf)_3$ [b]<br>(10%)	L-proline<br>(10%)	**5e** (*73*%)[c]
E	**3f**	EtOH	4 h<br>80 ºC	Open air	TFA<br>(20%)	L-proline<br>(10%)	**5e** (60)
F	**3f**	EtOH / H_2O	2 h<br>90 ºC	Argon	NaOH<br>(10 eq.)	-	**5e** (75)
G	**2a**	EtOH	4 h<br>80 ºC	Argon	TFA<br>(20%)	L-proline<br>(10%)	**4a** (42)
H	**2a**	Toluene	4 h<br>110 ºC	Open air	Piperidine<br>(40%)	-	**4a** (50)

[a] Yield obtained after purifying the reaction crude. [b] Three different Lewis Acid have been tested: $In(OTf)_3$ 10% mmol, $Eu(OTf)_3$ 10% mmol, and $Y(OTf)_3$ 10% mmol. All of them provided comparable conversions by LC-MS. [c] After purifying the crude corresponding to the reaction with $In(OTf)_3$, we obtained 73% of yield.

3.2. The Reaction Scope

3.2.1. MCR Inputs

Figure S2. The reactants (commercially available or synthesized) used in the MCR.

Aldehydes 1

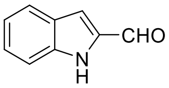
1a

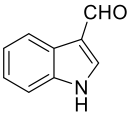
1b

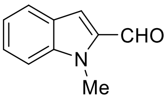
1c

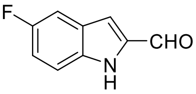
1d

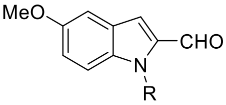
1e R = SO_2Ph
1f R = H

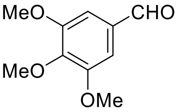
1g

Nucleophiles 2-3

1,3-Dicarbonyls **2**

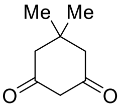
2a

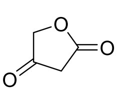
2b

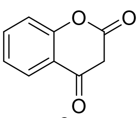
2c

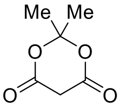
2d

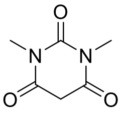
2e

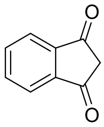
2f

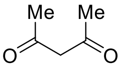
2g

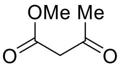
2h

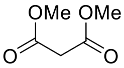
2i

Heterocycles **3**

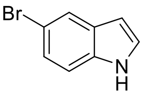
3a

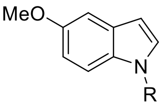
3b R = H
3c R = SO_2Ph

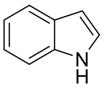
3d

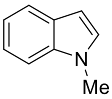
3e

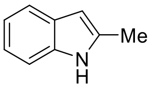
3f

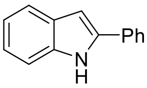
3g

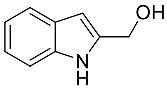
3h
(Also carbinol **Ia**)

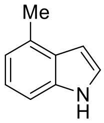
3i

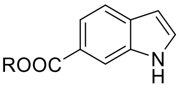
3j R = H
3k R = Me

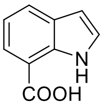
3l

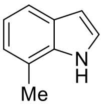
3m

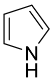
3n

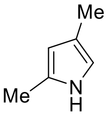
3o

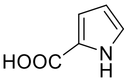
3p

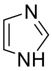
3q

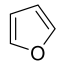
3r

Other nucleophiles (S1-4)

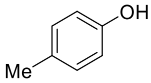
S1

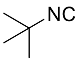
S2

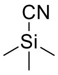
S3

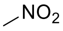
S4

3.2.2. Scope Limitations

Depending on the nature of the inputs of the MCR, certain limitations arose resulting in unproductive reactions or alternative results.

Aldehydes

Indole-3-carboxaldehyde **1b** did not evolve to the desired indolocarbazoles **4-5** in any case. With most 1,3-dicarbonyls **2**, the reaction proceeded to the Knoevenagel adducts **6c-f**. With indoles **3**, in some cases compounds **7** were detected (Figure S3). See section 2.3.2 for a detailed study on the reactivity trends of indole aldehydes **1a** and **1b** with 1,3-dicarbonyls **2**.

A likely explanation could be the lower nucleophilicity of indole C-2 (vs. C-3), together with the lower electrophilicity of the formyl group at indole C-3 in comparison when it is located at C-2, taking into account its pronounced vinylogous amide character in the former case.

Figure S3. Pathways of the MCR with indole-3-carboxaldehydes depending on the nucleophile and scope of the Knoevenagel adducts **6** with indole-3-carboxalehyde **1b**.

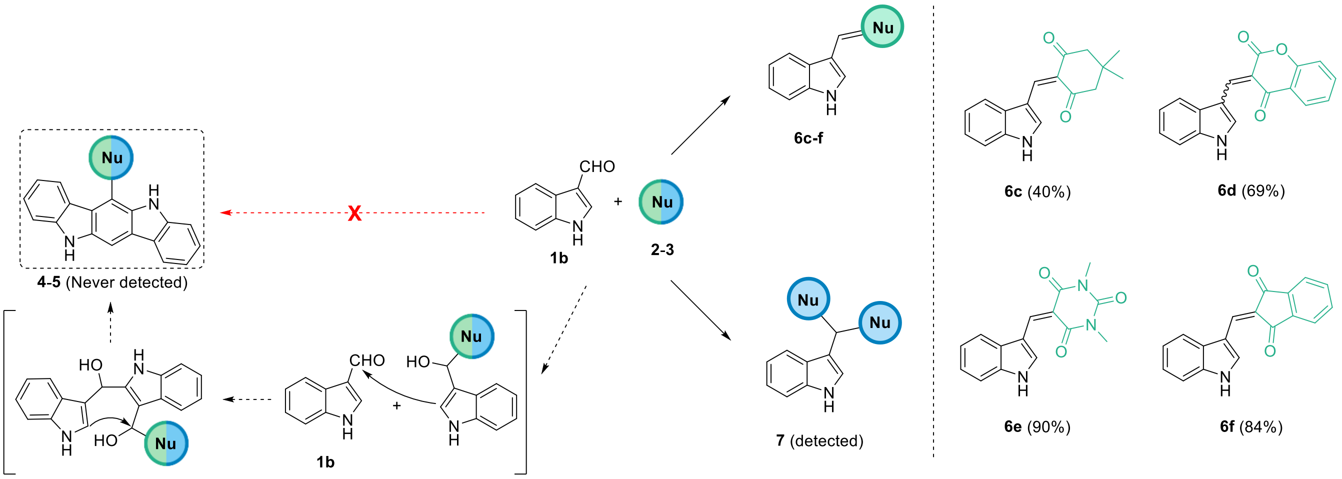

With 5-methoxy-1-(phenylsulfonyl)-1*H*-indole-2-carbaldehyde **1e** and 3,4,5-trimethoxybenzalehyde **1g**, the MCR with 2-methyl-1*H*-indole **3f** did not yield the desired indolocarbazoles **5** under the **standard conditions A**, and instead adducts **7a-b** were isolated from the respective reaction mixtures (Figure S4).

Figure S4. MCR with indole **3f** and aldehydes **1e** and **1g**.

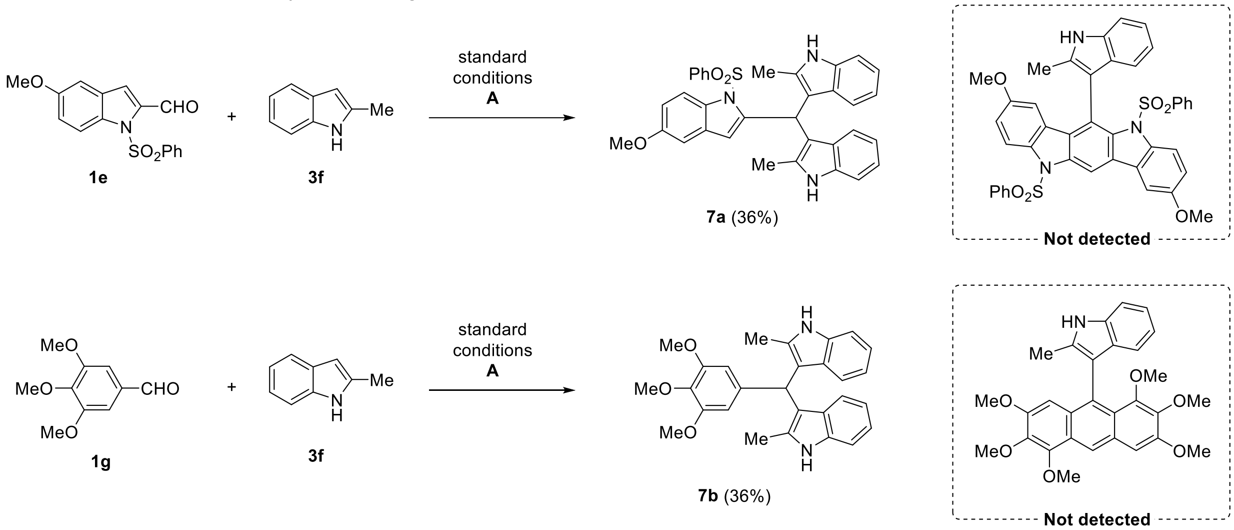

Nucleophiles 2-3 and S1-4

The Table S2 summarizes the results from the reaction of indole-2-CHO **1a** with various nucleophilic species **2**-**3** and **S1**-**4** under **standard conditions A**.

Table S2. Reaction of indole-2-carboxaldehyde **1a** with various nucleophilic species and observed results.

Entry	Input		Observations
A	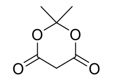	**2d**	No consumption of SM. After increasing the reaction time up to 4 days, a trace of product was detected through LC-MS.
B	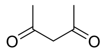	**2g**	No consumption of SM. After increasing the reaction time up to 4 days, a trace of product was detected through LC-MS.
C	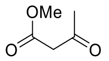	**2h**	No consumption of SM
D	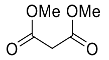	**2i**	No consumption of SM
E	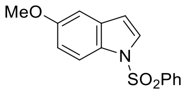	**3c**	Full consumption of **1a** and **3c**. Highly complex crude. LC-MS analysis suggested trace formation of the putative trimer of indole-2-CHO **S5** (Figure S10).
H	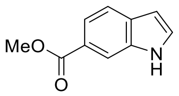	**3k**	Full consumption of **1a** and **3k.** Highly complex crude. LC-MS analysis suggested trace formation of the putative trimer of indole-2-CHO **S5** (Figure S10).
I	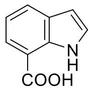	**3l**	Full consumption of **1a** and **3l**. Highly complex crude.
J	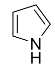	**3n**	Full consumption of **1a** and **3n**. Excessive polymerization and highly complex crudes. Vague evidence on the formation of bis-ICZ **S6** (Figure S5).
K	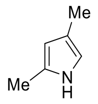	**3o**	Full consumption of **1a** and **3o**. Excessive polymerization and highly complex crudes. Vague evidence on the formation of bis-ICZ **S7** (Figure S5).
L	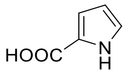	**3p**	Full consumption of **1a** and **3p**. Excessive polymerization and highly complex crudes.
M	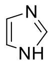	**3q**	No consumption of SM.
N	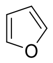	**3r**	No consumption of SM. Analogous results were observed for thiophene.
O	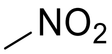	**S1**	Complex mixture.
P	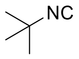	**S2**	^{1}H NMR of the crude suggested mostly unreacted **1a**. LC-MS analysis suggested trace formation of the putative trimer of indole-2-CHO **S5** (Figure S10).
Q	CN^-	**S3**	^{1}H NMR of the crude suggested mostly unreacted **1a**. Formation of cyanohydrin **Ih** and trace formation of ether **8** (Figure S8).
R	CH_3NO_2	**S4**	No consumption of SM.

With pyrroles **3n** and **3o**, the detected masses suggested the formation of the putative bis-indolocarbazole structures. They could not be isolated in their pure forms and fully characterized due to the very low conversion and dirty crudes. However, the ^{1}H NMR spectra of the isolated mg-scale fractions also indicated similar evidence. For instance, for the adduct arising from 2,4-dimethyl pyrrole **3o**, the NMR signals corresponding to the non-symmetric bis-indolocarbazole **S7** with 2 methyl groups, 5 azole NH peaks and the characteristic two singlet peaks of indolocarbazole residues were detectable (Figure S5A). For the assumably symmetric pyrrole **3n**-derived adduct **S6**, the NH signals with 1:2:2 ratio as well as the singlet for the indolocarbazole residue (in blue) were observed (Figure S5B). These hypothetical structures are consistent with the higher reactivity of pyrroles and their tendency to polymerize compared to indoles.

Figure S5. Experimental evidence on the putative bis-ICZ species arising from the interaction indole-2-CHO and pyrroles **3n-o**. A) ^{1}H NMR and LC-MS data of the putative adduct **S7** with 2,4-methyl pyrrole **3o**. B) ^{1}H NMR and LC-MS data of the putative adduct **S6** with pyrrole **3n**.

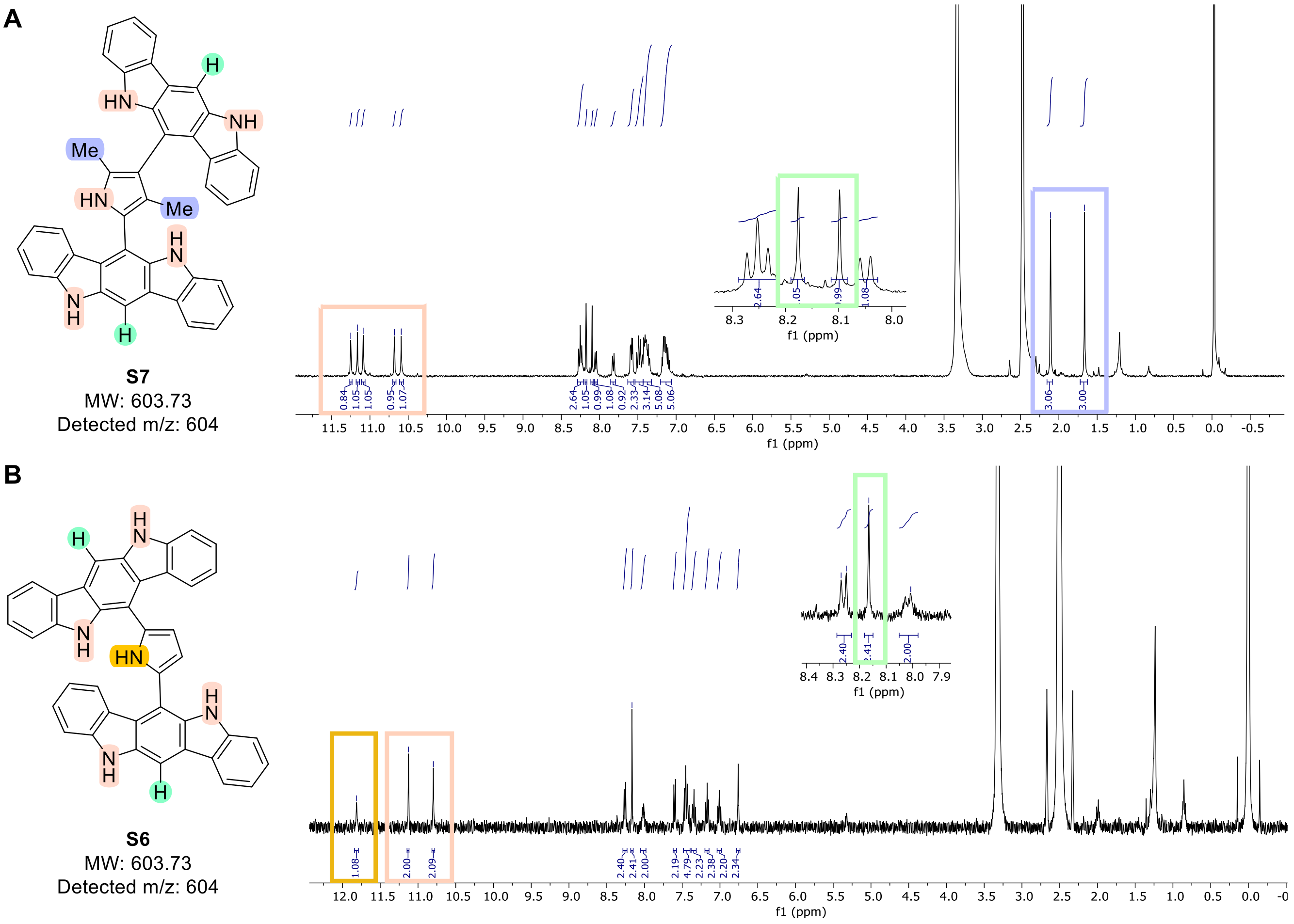

3.3. Mechanistic Studies

3.3.1. Experimental Observations

The reaction of indole-2-CHO **1a** with most nucleophilic species **3** (exemplified with model 2-methylindole **3f** in figure S6) gave only the corresponding 6-ICZ **5** under the **standard conditions A or B**. Other basic conditions failed to provide the expected 6-ICZ or the putative carbinol intermediate **Ie** (Figure S6A). Even with lesser nucleophilic partners such as 5-bromoindole **3a** and *p*-cresol **S1**, the putative carbinols were not detected either. However, in these cases we detected the competitive formation of ICZs **8** and **S8** (Figure S6B-C). Finally, we discovered that indole-2-CHO **1a** auto condensates in basic conditions to give **ICZ S8** quantitively, although in our hands the crude was untreatable to obtain pure samples (Figure S6D and 7). Other electron rich aldehydes did not follow this pattern and instead proceeded through the expected Cannizzaro rearrangement.

Note: all percentages given in this section represent approximate conversion calculated by crude ^{1}H NMR. Percentages in brackets represent isolated yields. Quant. = quantitative conversion. Nd = not detected.

Figure S6. A) Outcomes of the reaction between **1a** and **3f** under different conditions. B) Outcomes of the reaction between **1a** and **3a** under different conditions. C) Outcomes of the reaction between **1a** and **S4** under different conditions. D) Outcomes of the self-condensation of **1a** under different conditions.

A

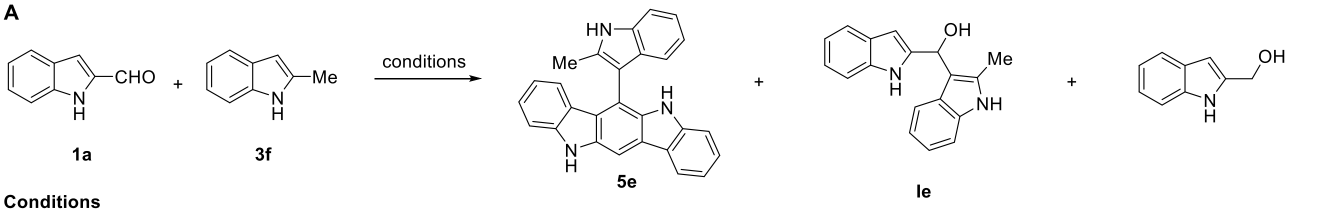

Conditions	5e	Ie	
a) Standard conditions A	*quant.*	*nd.*	-
b) Standard conditions B	*quant.*	*nd.*	-
c) DMF, Cs_2CO_3, rt, 17 h	*nd*	*nd*	-
d) Std. conds. B, slow addition of **1a** for 2 h	*traces*	*nd*	-
e) LDA, THF, 0 -> rt	*nd*	*nd*	quant. (reduction of aldehyde **1a**)

B

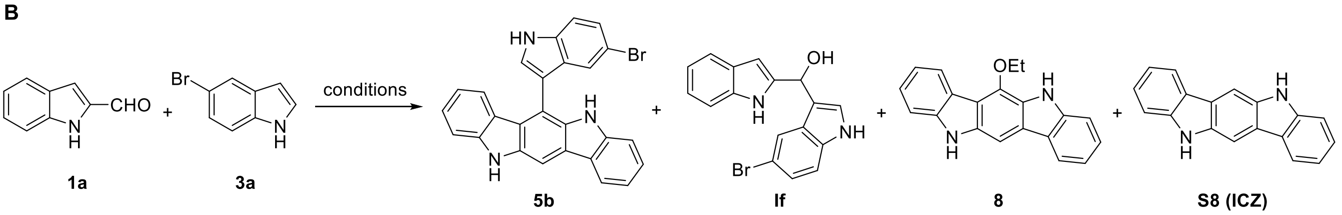

Conditions	5b	If	8	S8 (ICZ)
a) Standard conditions A	40%	*nd.*	*ca.* 10%	*nd*
b) Standard conditions B	60%	*nd.*	<5%	*ca.* 10%

C

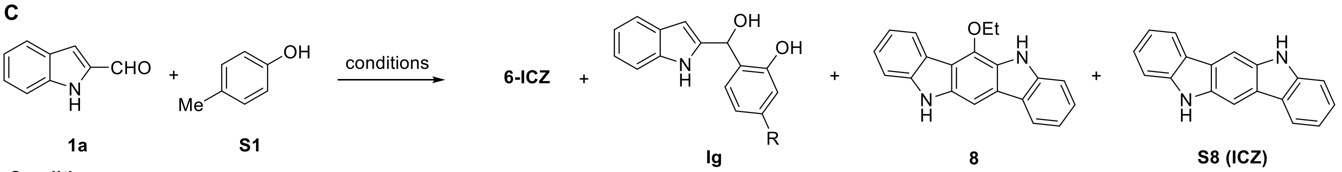

Conditions	6-ICZ	Ig	8	S8 (ICZ)
a) Standard conditions A	*nd*	*nd.*	<5%	*nd.*
b) Standard conditions B	*nd*	*nd.*	<5%	*quant.*

D

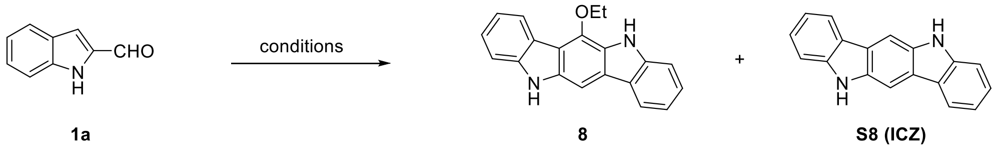

Conditions	8	S8 (ICZ)
a) Standard conditions A	*ca.* 10%	*nd.*
b) Standard conditions B	<5%	*quant.*
c) Sodium ethoxide, EtOH	*nd*	*nd*

Figure S7. Reaction crude ^{1}H NMR spectra of the auto condensation of indole-2-CHO **1a** under **standard conditions B**. **^{1}H NMR** (400 MHz, DMSO-*d$_6$*) δ 11.06 (s, 1H), 8.19 (d, *J* = 7.7 Hz, 1H), 8.11 (s, 1H), 7.45 (d, *J* = 8.0 Hz, 1H), 7.36 (ddd, *J* = 8.1, 7.0, 1.2 Hz, 1H), 7.12 (ddd, *J* = 7.4, 6.9, 1.1 Hz, 1H). NMR data are consistent with those previously reported in the literature.

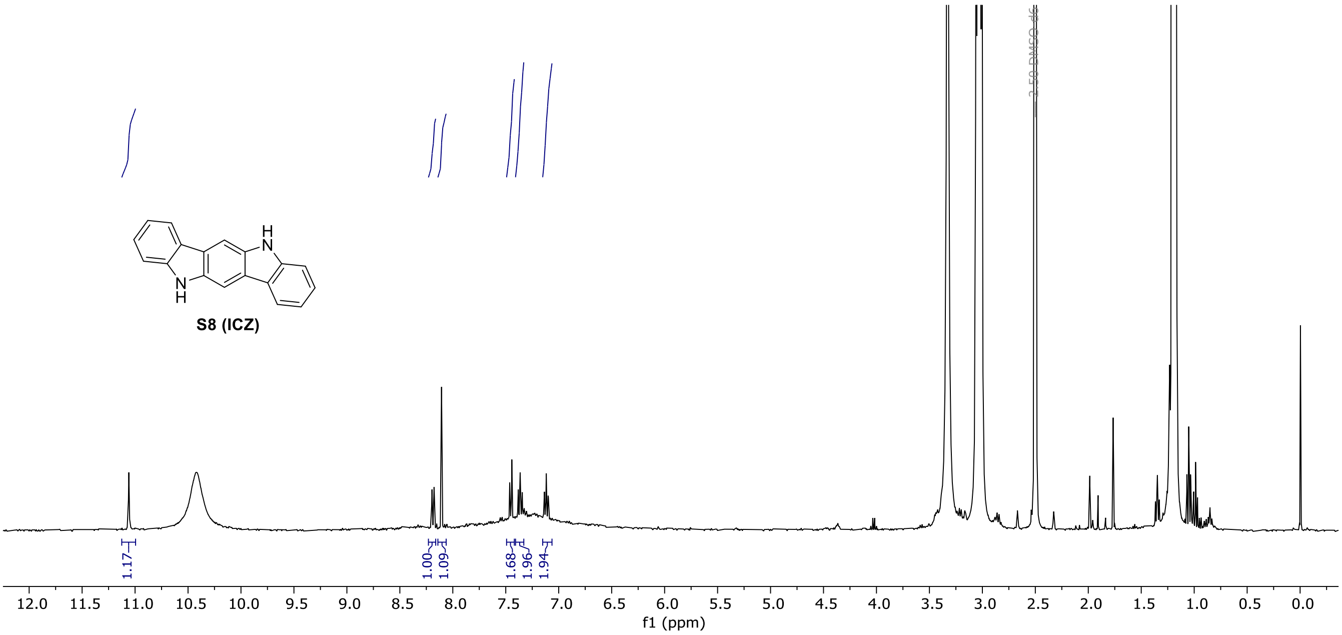

Other nucleophiles were used in the attempt to generate the putative reactive carbinol **I**. With cyanide **S3**, the cyanohydrin **Ih** was detected. It was only isolated in trace amounts due to its instability, and it was not converted to the desired 6-ICZ **S10** adduct, likely due to the lower reactivity of the indolyl C-3 due to the electron withdrawing effect of the -CN residue (Figure S8A). With nitromethane **S4**, nitroaldol **Ii** was never detected, and the reaction mostly resulted in the dehydration Henry adduct **S11**, which failed to convert to the desired 6-ICZ **S12**, consistently with the proposed reaction mechanism (Figure S8B).

Figure S8. A) Outcomes of the reaction between **1a** and **S3** under different conditions. B) Outcomes of the reaction between **1a** and **S4** under different conditions.

A

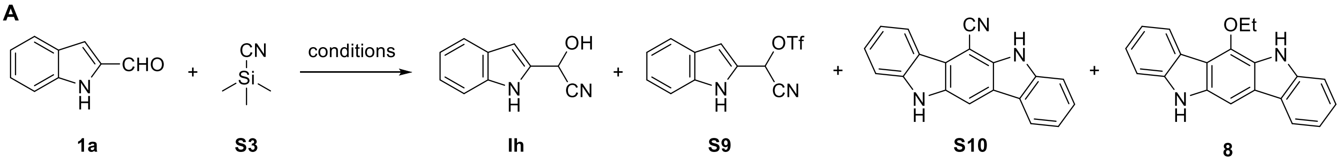

Conditions	Ih	S9	S10	8
a) Standard conditions A	30%	*nd*	*nd*	< 5%
b) Yb(OTf)$_3$ (20 mol%), CH_3CN, reflux, 3 h	20%	15%	*nd*	*nd*
c) Yb(OTf)$_3$ (20 mol%), CH_3CN, 0ºC to rt, 3 h	25%	*nd*	*nd*	*nd*
d) $MgBr_2$·Et_2O (10 mol%), CH_2Cl_2, rt, 3 h	60% (<3%)	*nd*	*nd*	*nd*
e) DABCO, THF, reflux, 17 h	50%	*nd*	*nd*	*nd*

B

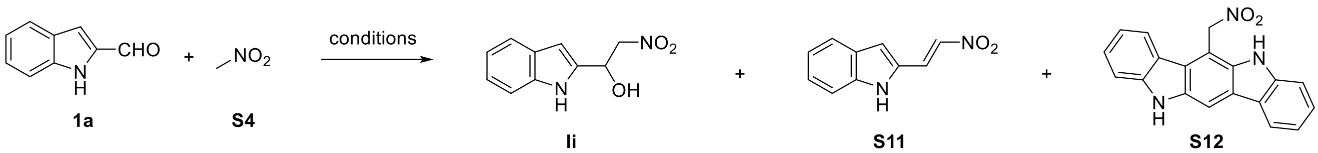

Conditions	Ii	S11	S12
a) Standard conditions A	*nd*	*nd*	*nd*
b) Cu(OAc)$_2$·TMEDA (20 mol%), CH_3CN, rt, 60 h	*nd*	quant.	*nd*
c) NH_4OAc (0.4 eq.), µW, 90 ºC, 1 h	*nd*	quant.	*nd*
d) Et_3N (1.5 eq.), THF, 0 ºC to rt	*nd*	*nd*	*nd*

Next, it was proposed to react **1a** with preformed carbinols **Ia** (R = H) and **Ib-d** (R = Bu, 4-F-Ph, and allyl, respectively). We hypothesized that the intermediate arising from the first nucleophilic attack of the carbinols **Ib-d** onto aldehyde **1a** could bypass the domino process to cyclize intramolecularly to generate adducts **S15**. However, carbinols **Ib-d** reacted as usual to give 6-ICZs **5g** and **5n-p**, respectively. Notably, this means that the intermediate domino process is faster / preferred over the intramolecular cyclization (Figure S9).

Figure S9. A) Outcomes of the reaction between **1a** and carbinols **Id-f** under different conditions. B) Possible pathways arising from the interaction of **1a** and carbinols **3**. C) ^{1}H NMR of the product **5n** after silica gel flash chromatography.

A

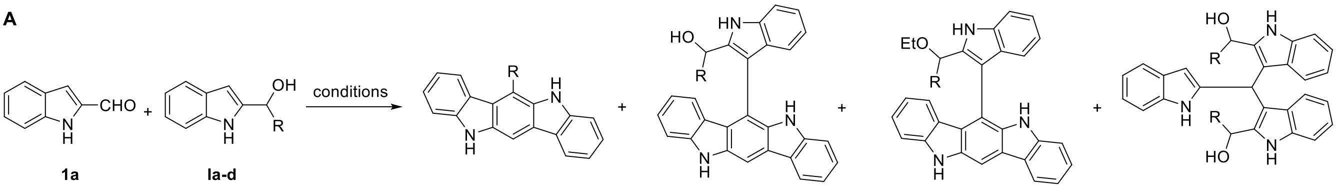

Conditions		S15	5g or 5n-p	S17	7c
**Ia** R = H	a) Standard conditions A	*nd*	30% (21%)	30%	*nd*
	b) Standard conditions B	*nd*	80% (45%)	*nd*	*nd*
	c) Piperidine, Toluene, reflux, 17 h	*nd*	*traces*	*nd*	80%
**Ib-d** R = Bu, 4-F-Ph, allyl	a) Standard conditions A	*nd*	*nd*	*nd*	*nd*
	b) Yb(OTf)$_3$, CH_3CN, 90 ºC, 3 h	*nd*	*nd*	*nd*	*nd*
	c) Standard conditions B	*nd*	*ca.* 60%	*nd*	*nd*

B

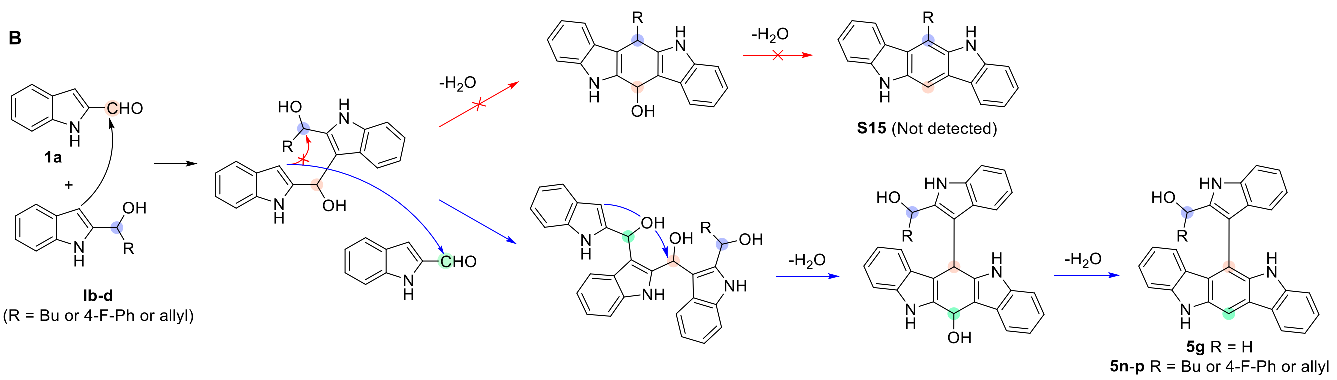

C

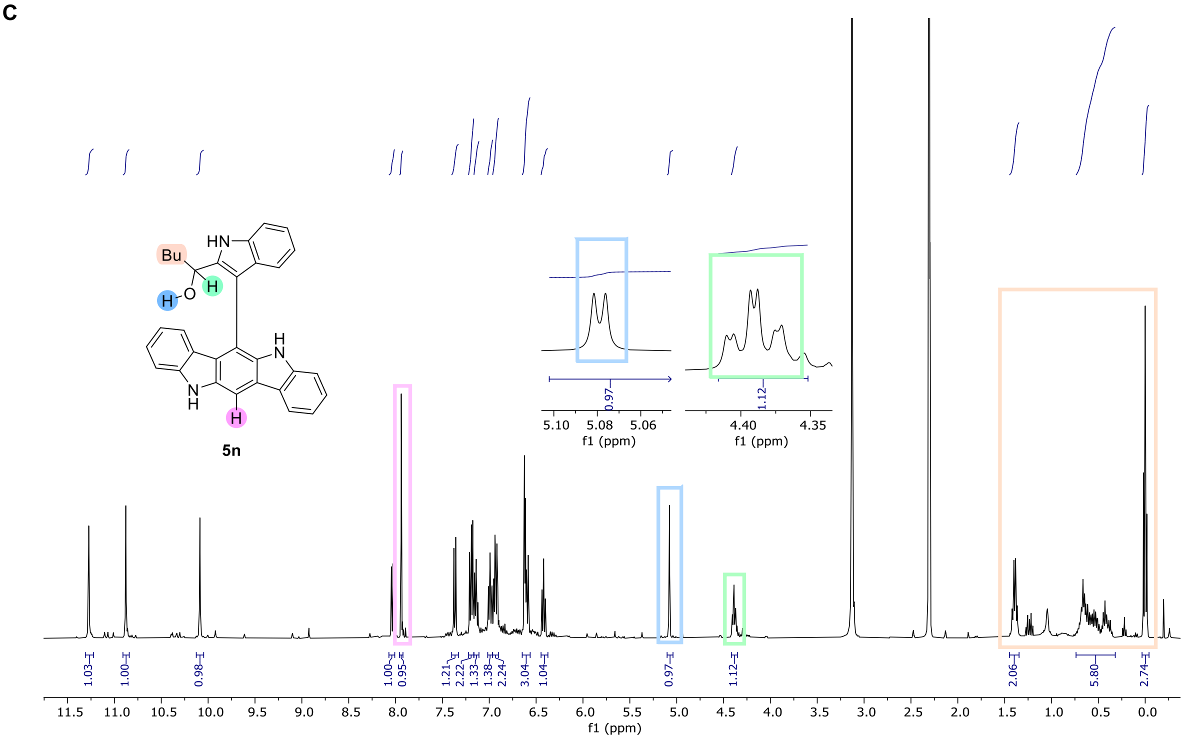

In the stated precedent cases, putative trimers of the indole-2-CHO **1a** were observed. In blank experiments, the self-oligomerization of the substrate was ruled out as no evolution was observed under **standard conditions A**. However, in the presence of a weak nucleophiles complex mixtures were produced, allowing the obtention of samples containing the supposed trimer **S5**. These materials were either detected by LC-MS or were isolated in mg-scale amounts. They were not fully characterized due to their possibly unstable nature and small isolated amounts. Thus, we cannot fully confirm their structures. However, their ^{1}H NMR and LC-MS data suggested some evidence regarding the presence of putative trimer **S5**. Hypothetically, their formation would arise from a reversible addition of the nucleophile upon the carbonyl, followed by the usual domino process leading to the indolocarbazole trimer or ensuing structures. Their instability may come from the reactive aldehyde moiety in the adduct or from the cationic heterocyclic arrangement (Figure S10).

Figure S10. Experimental evidence on the formation of putative trimeric species of **1a**. A) The reaction profiles using weak nucleophiles. B) The ^{1}HNMR of the isolated trimer **S5** from the reaction of **1a** with 4-bromophenol and representative signals. C) The proposed mechanism.

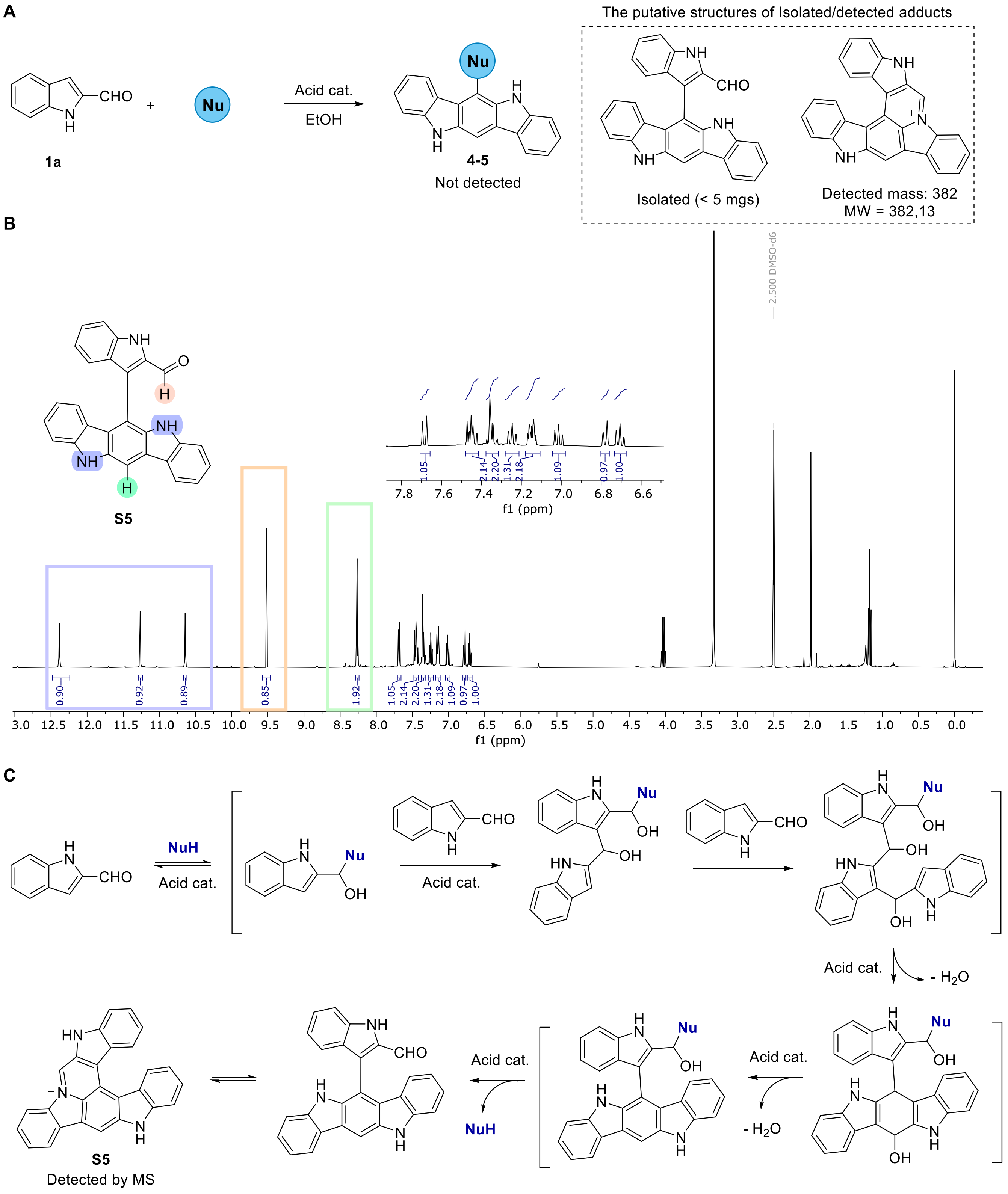

3.3.2. Detailed reaction mechanism under acidic and basic conditions

Regarding the reaction mechanism under standard acidic conditions A, we propose an electrophilic cyclization step of the putative intermediate **B** (Figure S11A). Likewise, the mechanistic pathway under basic conditions parallelizes that under acidic media, presumably involving a neutral conjugated moiety suffering the intramolecular attack of an ionized indole nucleophile in the putative intermediate **E** (Figure S11B). Both hypothetical mechanistic approaches share the natural electrophilicity of the formyl group (enhanced in the acid-catalyzed process) and the native nucleophilicity of the C-3 position of an indole ring (increased in basic conditions). Incidentally, under standard conditions B, the formation of ICZ S8 could be explained through an ethoxy-mediated reduction of the diol **G**, which is supported by precedent reports on similar systems.[3,4]

Figure S11. A) Detailed putative reaction mechanism under standard conditions A. B) Detailed putative reaction mechanism under standard conditions B, including the self-condensation of indole-2-CHO **2a** to give the **ICZ** compound.

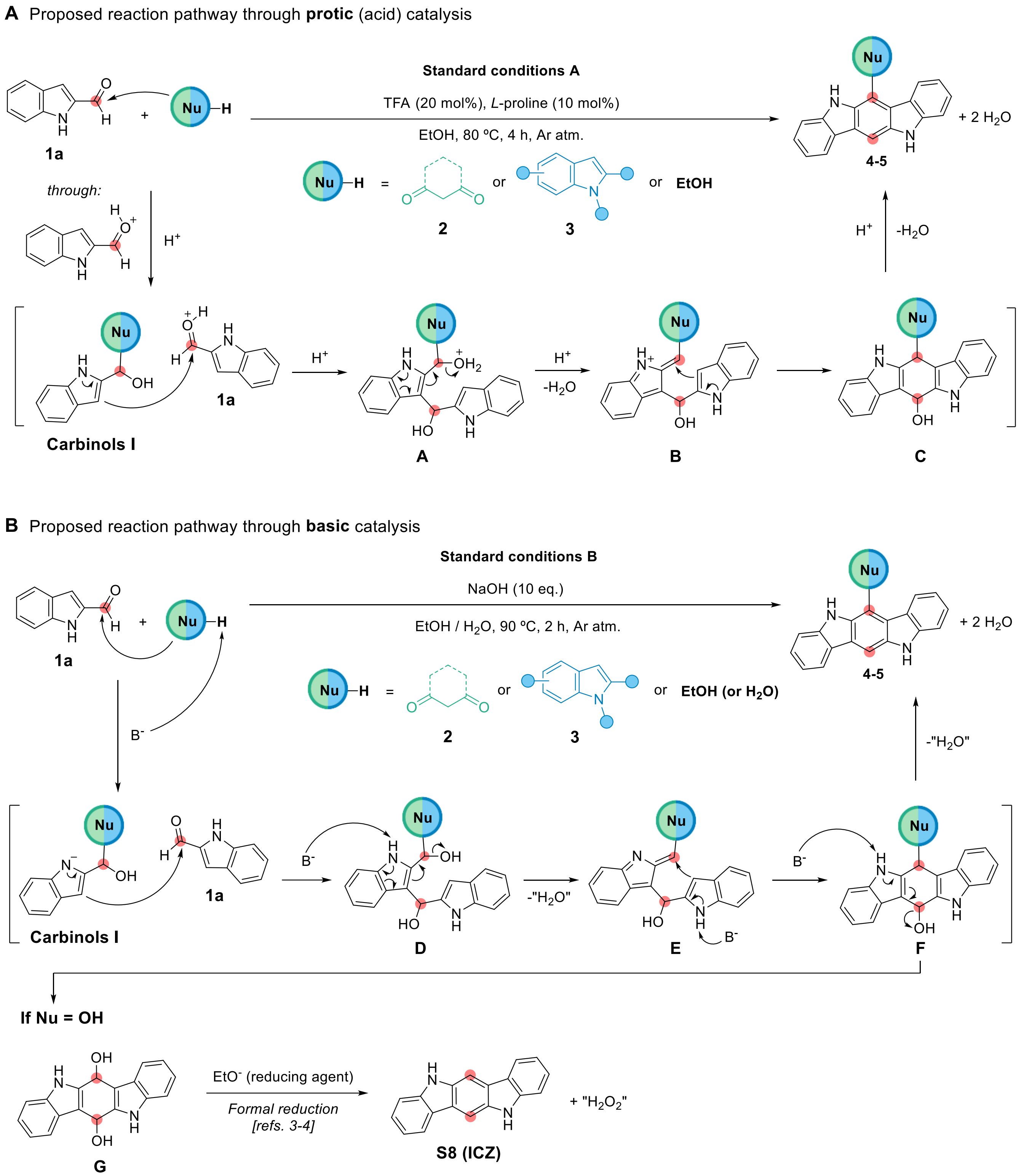

3.3.3. Computational Studies on the Reactivity Trends of Aldehydes 1a-b with 1,3-Dicarbonyls 3

According to the proposed mechanism (see main text and SI, section 3.3.1), indole aldehydes **1** and 1,3-dicarbonyls **2** would react to give carbinol intermediates **I**, which in turn could either react with another equivalent of aldehyde to generate ICZ **4** or dehydrate to form the Knoevenagel adduct **6**. A clear dichotomy was observed, as indole-2-caboxaldehyde **1a** generally proceeded to 6-ICZs **4**, while indole-3-carboxaldehyde **1b** always gave Knoevenagel adducts **6** (Figure S12).

Figure S12. A) Reaction profile for the formation of indolocarbazoles **4** and Knoevenagel adducts **6** from β-dicarbonyl compounds **2**. B) Tested combinations of **1a-b** and **2a-f** and results.

A

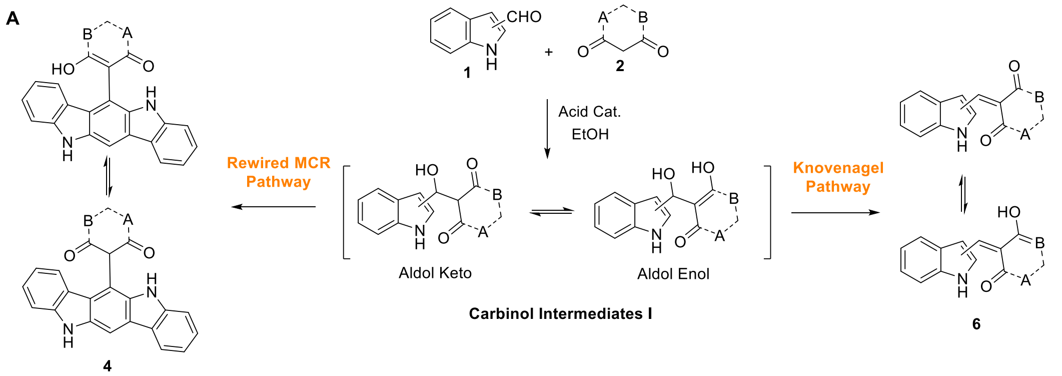

B

	2a	2b	2c	2d	2e	2f
1a	**4a**	**4f**	**4g**	**4** (traces, by LC-MS)	**6a**	**6b**
1b	**6c**	**6** (traces, by LC-MS)	**6d**	**6** (traces, by LC-MS)	**6e**	**6f**

The results from the reaction of indole-2-CHO **1a** with N-methyl baribituric acid **2e** and indanone **2f**, may be explained by the degree of enolization of the 1,3-dicarbonyl residues in carbinols **I**. Presumably, a lower enolization degree can relatively slow down the dehydration leading to the Knoevenagel adduct, whereas the opposite trend would favor the 6-ICZ formation, by coordination with an incoming aldehyde (Figure S13).

Figure S13. Putative enolization influences the Indolocarbazole formation.

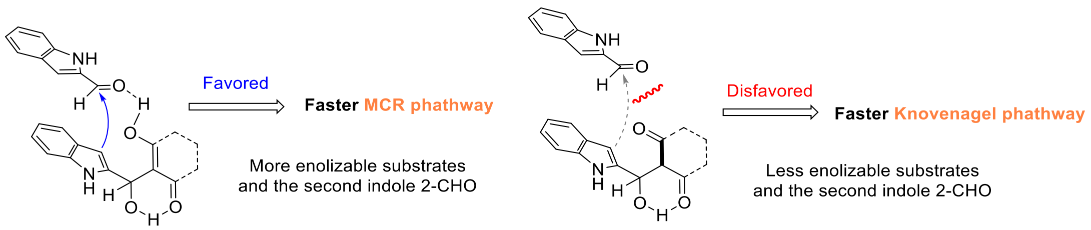

As for the dichotomy observed between the indole-2-CHO **1a** and indole-3-CHO **1b**, it was hypothesized that the dehydration of the intermediate carbinols **I** was favored in the latter, in agreement with the facilitated activation of indole 3-carbinols due to the more efficient electron donation from the aromatic nucleus, in comparison with the situation found with indole 2-derivatives (Figure S14).

Figure S14. Favored dehydration form indole 3-derivatives in route to Knoevenagel adducts.

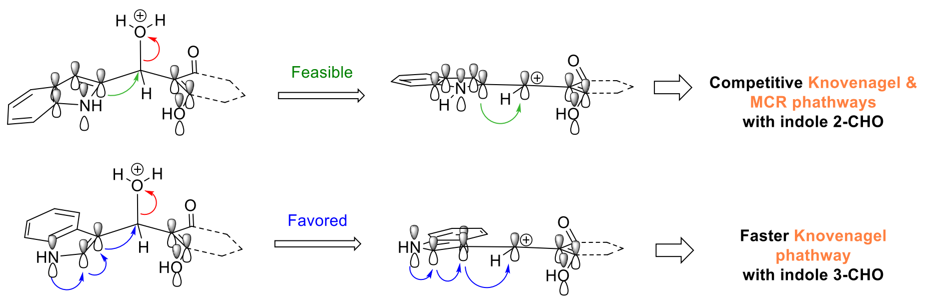

Moreover, the stability of intermediates shown in the Figure S11 were calculated in vacuo after optimization through molecular mechanics (MMF) and semiempirical (PM3) methods using a SPARTAN suite (Figure S15-18).[5] As seen, in practically all series, the Knoevenagel adducts coming from indole-3-CHO are more stable than their 2-CHO counterparts. We anticipate that reaction barriers may parallel the differences in energy, justifying the observed facts in a qualitative manner.

Figure S15. Calculated geometries and energies for the species arising from the interaction of dimedone **2a** with indole 2-CHO **1a** (top) and indole-3-CHO **1b** (bottom).

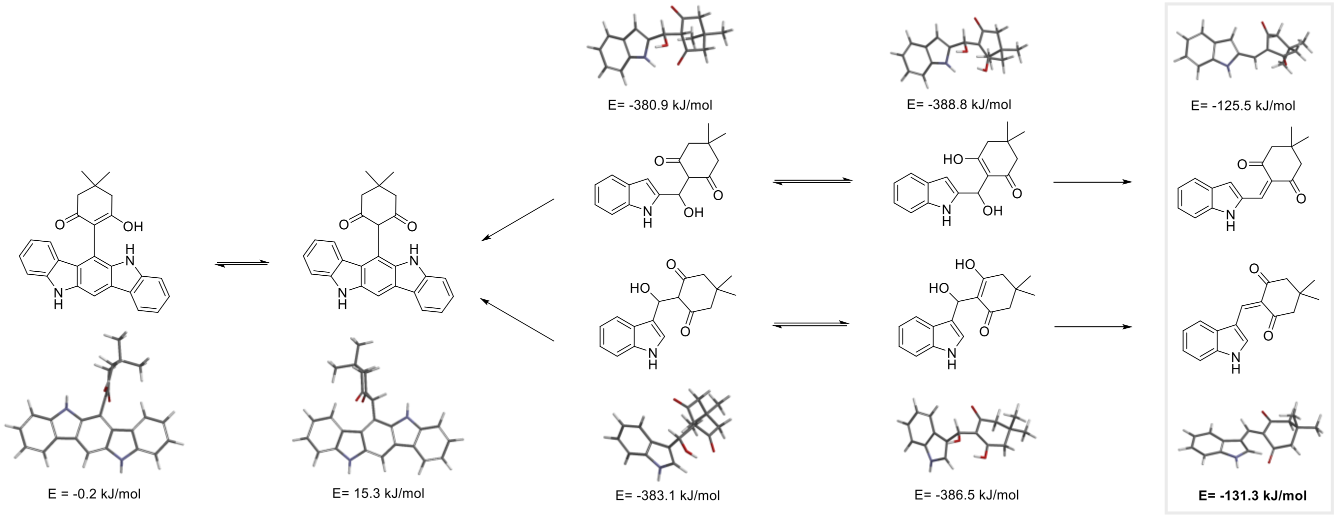

Figure S16. Calculated geometries and energies for the species arising from the interaction of tetronic acid **2b** with indole-2-CHO **1a** (top) and indole-3-CHO **1b** (bottom).

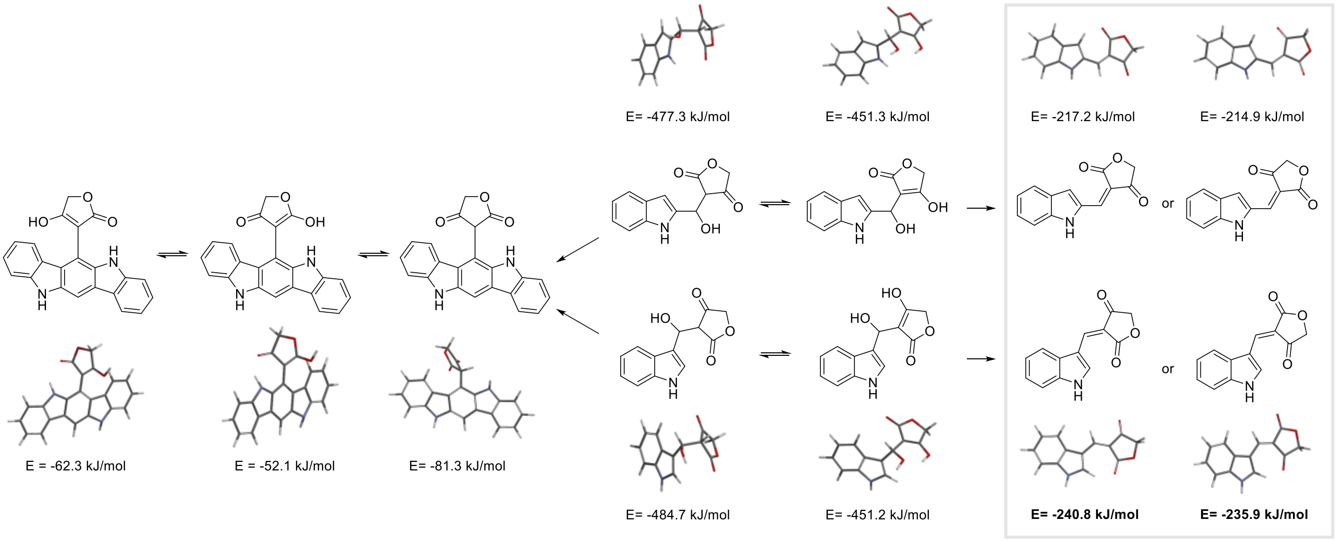

Figure S17. Calculated geometries and energies for the species arising from the interaction of Meldrum's acid **2d** with indole-2-CHO **1a** (top) and indole-3-CHO **1b** (bottom).

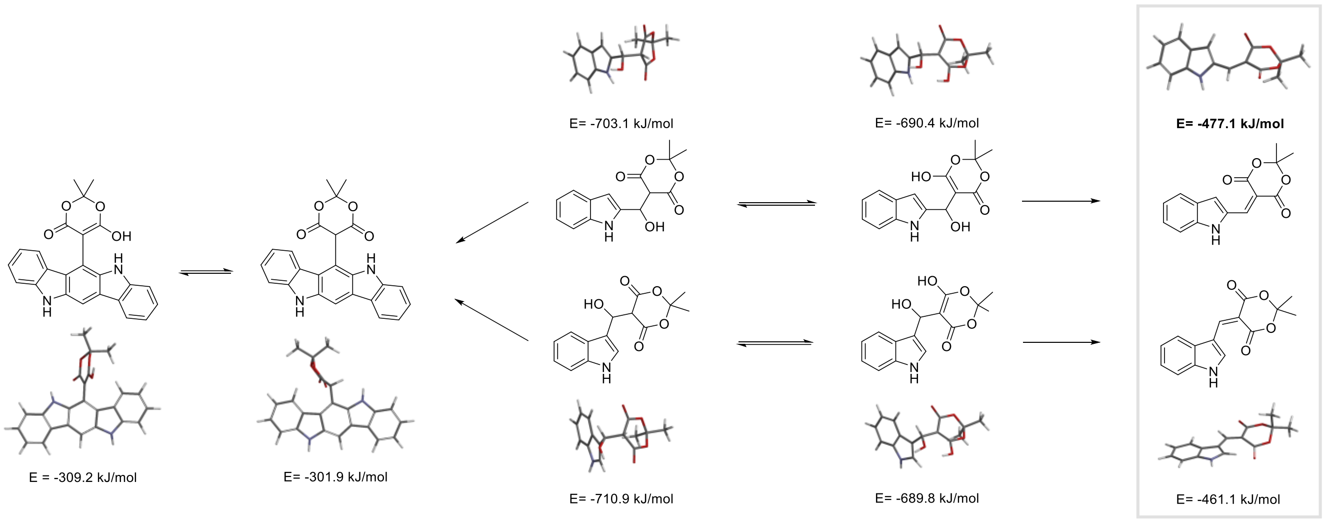

Figure S18. Calculated geometries and energies for the species arising from the interaction of N-methyl barbituric acid **2e** with indole-2-CHO **1a** (top) and indole-3-CHO **1b** (bottom).

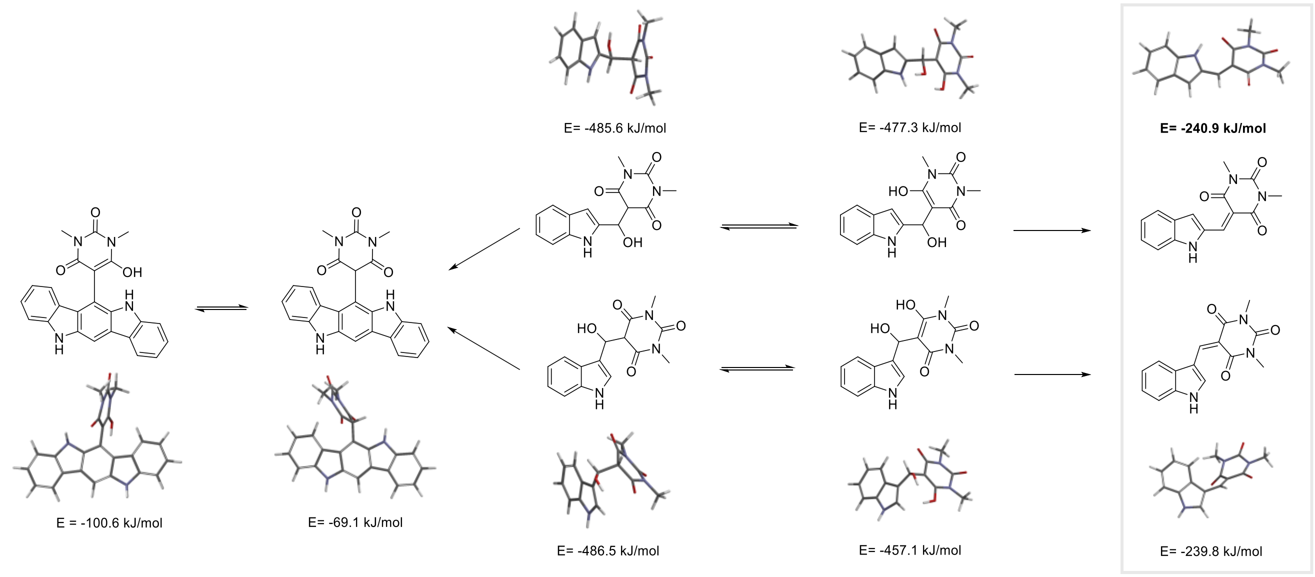

4. Experimental Procedures and Characterization Data

4.1. General Synthetic Procedures

General Procedure A

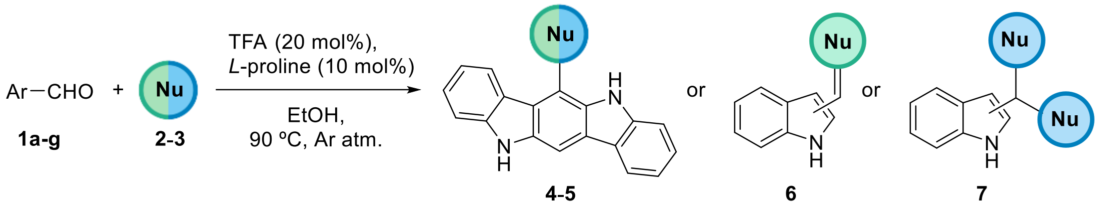

To a solution of aldehyde **1** (2 eq.) and nucleophile **2** or **3** (1 eq.) in EtOH (0.25 M) under inert atmosphere was added *L*-proline (10 mol%) and trifluoroacetic acid (TFA, 20 mol%). The reaction was purged with vacuum-argon cycles, heated to 80 ºC and stirred for 4 h or until full consumption of starting materials (TLC or LC-MS control). The reaction was diluted with EtOAc and washed with a saturated aqueous solution of $NaHCO_3$ and saturated brine. The organic layer was dried over sodium sulfate, filtered, and concentrated under reduced pressure. The residue was purified using the indicated purification protocol to afford the pure products **4**-**6**. In the stated cases, the precipitate formed during the reaction was filtered washing with cold EtOH to afford the pure products **4**-**7**.

General Procedure B

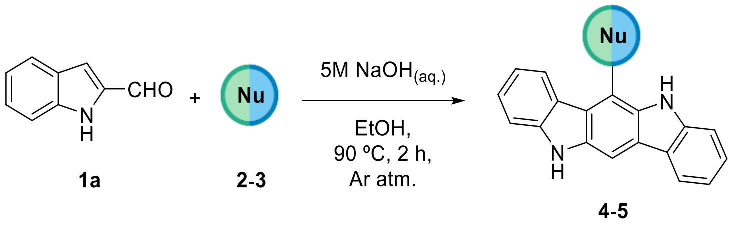

A solution of aldehyde **1a** (2 eq.) and nucleophile **2** or **3** (1 eq.) in EtOH / 5M $NaOH_{(aq.)}$ (1:1, 0.25 M) under inert atmosphere was heated to 90 ºC and stirred for 2 h or until full consumption of starting materials (TLC or LC-MS control). The reaction was diluted with EtOAc and washed with 1M aqueous HCl solution, water, and saturated brine. The organic layer was dried over sodium sulfate, filtered, and concentrated under reduced pressure. The crude was purified using the indicated purification protocol to afford the pure products **4**-**5**.

General Procedure C

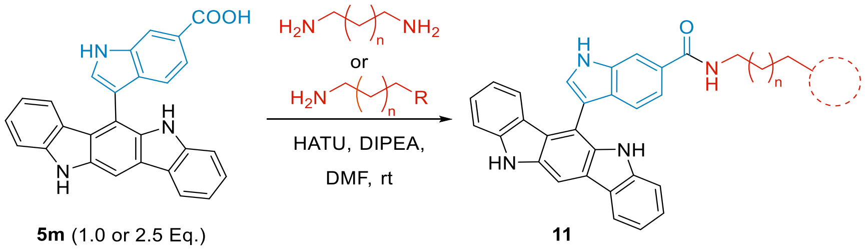

To a solution of **5m** (1.0 eq. or 2.5 eq.) in dry DMF (0.025 M) under argon atmosphere was added DIPEA (1.0 – 5.0 Eq.) and the mixture was stirred for 5 min. Then was added HATU (1.2 eq.) and the mixture was stirred for 10 min. Then was added a solution of the amine or linker (1.1 Eq.) in 0.5 mL of DMF and the reaction was left to stir at room temperature for the indicated time. After reaction completion (TLC control) the reaction mixture was partitioned between EtOAc and water. The aqueous layer was extracted with EtOAc (3 x 40 mL), and the combined organic layers were washed with saturated brine (20 mL), dried over $MgSO_4$, filtered, and concentrated under reduced pressure. The residue was purified via silica gel flash chromatography using the indicated solvent system to afford the pure products **11**.

4.2. Synthesis and Characterization of Starting Materials

Synthesis of Compounds **1a**, **1d**, and **3h**

The starting materials **1a** and **1d** were prepared adapting a reported procedure for the functionalization of ethyl-indole-carboxylates.[6] Compound **1a** was also purchased from a commercial source.

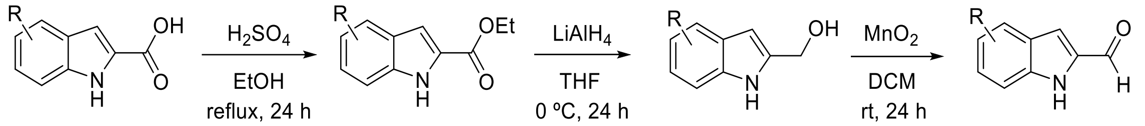

(i) **S5** R = H **S6** R = 5-F **(ii)** **3h** R = H **S7** R = 5-F **(iii)** **1a** R = H **1d** R = 5-F

1*H*-Indole-2-carbaldehyde (1a)

(ii) A solution of commercially available **S5** (1.14 g, 6 mmol) in dry THF (8 mL) was cooled to 0 ºC under inert atmosphere, and $LiAlH_4$ (1 M solution in THF, 30 mL) was added dropwise with an addition funnel. The resulting mixture was warmed up to room temperate and stirred for 24 hours. After reaction completion (TLC control), the crude mixture was quenched with water (150 mL) and extracted with diethyl ether (4 x 200 mL). The combined organic layers were washed with water, brine, and dried over anhydrous Na_2SO_4. The mixture was then filtered and evaporated under reduced pressure to obtain (1*H*-Indole-2-yl)methanol **3h** (0.800 g, 90%) as an off-white powder, which was used in the next step and in the MCR without further purification. **^{1}H NMR** (400 MHz, DMSO-*d₆*) δ 10.97 (br s, 1H), 7.44 (ddt, *J* = 7.8, 1.4, 0.8 Hz, 1H), 7.31 (dq, *J* = 8.0, 0.9 Hz, 1H), 7.01 (ddd, *J* = 8.1, 7.0, 1.3 Hz, 1H), 6.93 (ddd, *J* = 8.0, 7.0, 1.1 Hz, 1H), 6.25 (dq, *J* = 1.9, 0.9 Hz, 1H), 5.21 (t, *J* = 5.6 Hz, 1H), 4.60 (dd, *J* = 5.6, 0.8 Hz, 2H).

(iii) To a solution of **3h** (0.663 g, 4.30 mmol) in DCM (10 mL) was added manganese oxide (1.91 g, 22 mmol) and the reaction mixture was stirred at room temperature for 24 hours. After reaction completion (TLC control), the reaction was filtered through Celite washing with DCM to remove inorganics, and the filtrate was concentrated under reduced pressure. The crude was purified *via* silica gel flash column chromatography (EtOAc/hexane gradient from 0:100 to 50:50 v/v) to afford the title compound **1a** as a light brown powder (0.450 g, 72%). **^{1}H NMR** (400 MHz, DMSO-d_6) δ 11.96 (br s, 1H), 9.85 (s, 1H), 7.75 (dt, *J* = 8.1, 1.1 Hz, 1H), 7.46 (dq, *J* = 8.4, 1.0 Hz, 1H), 7.39 (d, *J* = 1.0 Hz, 1H), 7.33 (ddd, *J* = 8.3, 6.9, 1.2 Hz, 1H), 7.11 (ddd, *J* = 8.0, 6.9, 1.0 Hz, 1H).

5-Fluoro-1*H*-indole-2-carbaldehyde (1d)

(i) To a solution of 5-fluoro-1H-indole-2-carboxylic acid (2.0 g, 11.16 mmol) in EtOH (60 mL) was added H_2SO_4 (5 mL, 90 mmol) and the reaction was heated to reflux temperature and stirred for 24 h. After reaction completion (TLC control), the reaction mixture was cooled down to 0 ºC (ice bath) and a precipitate formed. The precipitate was filtered washing with cold EtOH to afford ethyl 5-fluoro-1H-indole-2-carboxylate **S6** (1.92 g, 82%) as a white solid, which was used in the next step without further purification. **^{1}H NMR** (400 MHz, DMSO-*d_6*) δ 11.99 (s, 1H), 7.49 – 7.38 (m, 2H), 7.17 – 7.08 (m, 2H), 4.34 (q, J = 7.1 Hz, 2H), 1.34 (t, J = 7.1 Hz, 3H).

(ii) A solution of **S6** (1 g, 4.7 mmol) in dry THF (6.6 mL) was cooled to 0 ºC under inert atmosphere, and $LiAlH_4$ (1 M solution in THF, 30 mL) was added dropwise with an addition funnel. The resulting mixture was warmed up to room temperate and stirred for 24 hours. After reaction completion (TLC control), the crude mixture was quenched with water (150 mL) and extracted with diethyl ether (4 x 200 mL). The combined organic layers were washed with water, brine, and dried over anhydrous Na_2SO_4. The mixture was then filtered and evaporated under reduced pressure to obtain (5-fluoro-1H-indol-2-yl)methanol **S7** (0.62 g, 79%) as a yellow solid, which was used in the next step. **^{1}H NMR** (400 MHz, DMSO-*d_6*) δ 11.08 (s, 1H), 7.29 (dd, J = 8.7, 0.7 Hz, 1H), 7.20 (dd, J = 10.0, 0.6 Hz, 1H), 6.85 (ddd, J = 9.6, 8.7, 2.6 Hz, 1H), 6.26 (s, 1H), 5.27 (t, J = 5.6 Hz, 1H), 4.59 (d, J = 0.8 Hz, 2H).

(iii) To a solution of **S6** (0.59 g, 4.1 mmol) in DCM (8.3 mL) was added manganese oxide (1.6 g, 18 mmol) and the reaction mixture was stirred at room temperature for 24 hours. After reaction completion (TLC control), the reaction was filtered through Celite washing with DCM to remove inorganics, and the filtrate was concentrated under reduced pressure to afford the title compound **1d** as a light brown solid (0.32 g,55%) which was used in the MCR without further purification. **^{1}H NMR** (400 MHz, DMSO-*d_6*) δ 12.07 (s, 1H), 9.86 (s, 1H), 7.52 (ddd, *J* = 9.6, 2.6, 0.7 Hz, 1H), 7.46 (ddt, *J* = 9.0, 4.6, 0.8 Hz, 1H), 7.37 (dd, *J* = 2.2, 0.9 Hz, 1H), 7.21 (td, *J* = 9.2, 2.6 Hz, 1H).

Synthesis of Compounds **1c** and **3e**

The starting materials **1c** and **3e** were prepared adapting a reported procedure for the synthesis of 1-methyl-1*H*-indole-2-carboxaldehyde.[7] Compound **3e** was also purchased from a commercial source.

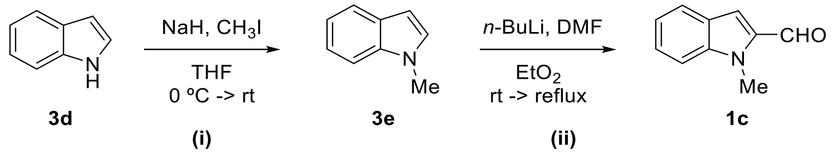

1-Methyl-1*H*-indole (3e)

(i) To a 0 ºC (ice bath) solution of indole (4.9 g, 41.8 mmol, 1 Eq.) in THF (120 mL, 0.35 M) was added NaH (60% dispersion in mineral oil, 2.9 g, 72.5 mmol, 1.7 Eq.). The heterogeneous mixture was stirred at 0ºC for 15 min and at rt for 1 h. After this time the reaction was cooled to 0 ºC (ice bath) and was treated with methyl iodide (3.5 mL, 56.0 mmol, 1.3 eq). The reaction was allowed to warm to rt and was stirred for 30 min. After reaction completion (TLC control), the mixture was cooled using an ice bath and the reaction was quenched by addition of saturated aqueous NH_4Cl (50 mL). The aqueous layer was extracted with ether (3 x 100 mL). The organic layers were combined, washed with saturated brine, dired over sodium sulfate, filtered, and concentrated under reduced pressure to afford the title compound **3e** as a red-colored liquid (5.4 g, 98%), which was used without further purification. **^{1}H NMR** (400 MHz, $CDCl_3$) δ 7.63 (dt, *J* = 7.9, 1.0 Hz, 1H), 7.38 – 7.30 (m, 1H), 7.26 – 7.20 (m, 1H), 7.17 – 7.07 (m, 1H), 7.06 (d, *J* = 3.0 Hz, 1H), 6.49 (dd, *J* = 3.1, 0.9 Hz, 1H), 3.80 (s, 3H).

1-Methyl-1*H*-indole-2-carboxaldehyde (1c)

(ii) To a 0 ºC (ice bath) solution of **3e** (2.4 g, 18.3 mmol, 1 Eq.) in Et_2O (50.0 mL) was added dropwise *n*-BuLi (2.5 M in hexanes, 7.3 mL, 1 Eq.). The rection was heated to reflux temperature and stirred for 2 h. After this time the reaction mixture was allowed to cool to rt and was added dropwise DMF (2.1 mL, 27.4 mmol, 1.5 Eq.). The reaction was heated to reflux temperature and stirred for 2 h. After reaction completion (TLC control) mixture was cooled using an ice bath and the reaction was quenched by addition of saturated aqueous NH_4Cl (20 mL). The aqueous layer was extracted with ether (3 x 50 mL). The organic layers were combined, washed with saturated brine, dired over sodium sulfate, filtered, and concentrated under reduced pressure. The residue was purified *via* silica gel flash chromatography (EtOAc / hexane gradient from 0:100 to 10:90 v/v) to afford the title compound **1c** as an off-white solid (1.5 g, 52%). **^{1}H NMR** (400 MHz, $CDCl_3$) δ 9.90 (s, 1H), 7.74 (dt, *J* = 8.1, 1.0 Hz, 1H), 7.46 – 7.38 (m, 2H), 7.26 (d, *J* = 0.8 Hz, 1H), 7.29 – 7.23 (m, 0H), 7.18 (ddd, *J* = 8.0, 6.2, 1.7 Hz, 1H), 4.11 (s, 3H).

Synthesis of Compounds **3c** and **1e-f**

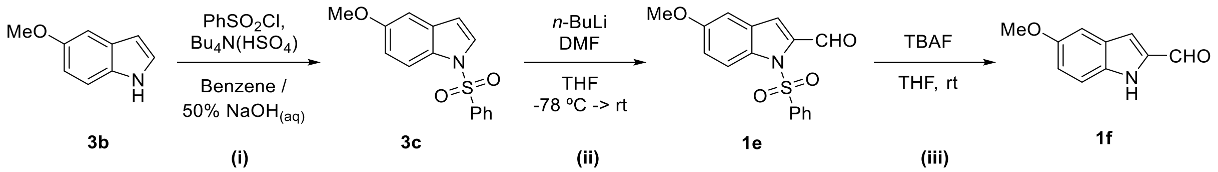

5-Methoxy-1-(phenylsulfonyl)-1*H*-indole (3c)

(i) The synthesis of **3c** was done following a literature procedure for the synthesis of *N*-phenylsulfonyl indoles.[8] To a solution of 5-methoxy-1*H*-indole (2 g, 13.6 mmol, 1 Eq.) and tetrabutylammonium hydrogen sulfate (60 mg, 0.18 mmol, 0.01 Eq.) in benzene (60 mL) was added a 50% aqueous NaOH solution (20 mL) and the mixture was stirred for 5 min. After this time, it was added dropwise a solution of chlorobenzene sulfonyl chloride (3.7 g, 21 mmol, 1.5 Eq.) in benzene (20 mL). The reaction was stirred at rt for 60 h. After reaction completion (TLC control) the phases were separated, and the organic layer was washed with water (2 x 50 mL). The organic layer was washed with saturated brine, dried over sodium sulfate, filtered, and concentrated under reduced pressure to afford the title compound **3c** as a white solid (3.7 g, 95%). **^{1}H NMR** (400 MHz, DMSO-d_6) δ 7.95 – 7.90 (m, 2H), 7.82 (dt, *J* = 9.0, 0.6 Hz, 1H), 7.75 (dd, *J* = 3.6, 0.5 Hz, 1H), 7.70 – 7.64 (m, 1H), 7.60 – 7.54 (m, 2H), 7.11 (dd, *J* = 2.6, 0.5 Hz, 1H), 6.94 (ddd, *J* = 9.1, 2.6, 0.5 Hz, 1H), 6.76 (dd, *J* = 3.6, 0.8 Hz, 1H), 3.74 (s, 3H).

5-Methoxy-1-(phenylsulfonyl)-1*H*-indole-2-carboxaldehyde (1e)

(ii) To a -78 ºC solution of **3c** (1.1 g, 3.9 mmol, 1 Eq.) in anhydrous THF (20 mL, 0.2 M) was added dropwise *n*-BuLi (2.5 M in hexanes, 1.6 mL, 1 Eq.). The reaction mixture was stirred at -78 ºC for 1 h. After this time was added DMF (1.4 mL, 18 mmol, 4.6 Eq.) and the reaction was allowed to warm to rt and was stirred for 2 h. After reaction completion (TLC control), the reaction was quenched by addition of aqueous saturated $NaHCO_3$ (100 m) and extracted with EtOAc (3 x 100 mL). The combined organic layers were dried over sodium sulfate, filtered, and concentrated under recued pressure to give a yellow solid. The crude product was purified *via* silica gel flash chromatography (EtOAc/hexane gradient form 0:100 to 20:80 v/v) to afford the title compound **1e** as an off-white

solid (1.1 g, 95%). ^{1}**H NMR** (400 MHz, DMSO-d_6) δ 10.35 (s, 1H), 8.04 (dt, *J* = 9.1, 0.7 Hz, 1H), 7.88 – 7.83 (m, 2H), 7.69 (ddt, *J* = 8.0, 6.9, 1.2 Hz, 1H), 7.60 – 7.54 (m, 3H), 7.25 (d, *J* = 2.1 Hz, 1H), 7.21 (dd, *J* = 9.2, 2.6 Hz, 1H), 3.77 (s, 3H).

5-Methoxy-1*H*-indole-2-carboxaldehyde (1f)

(iii) The deprotection of compound **1e** was done adapting a literature procedure for a related substrate. To a solution of **1e** (392 mg, 1.2 mmol, 1 Eq.) in THF (11 mL, 0.1 M) was added TBAF (1 M in THF, 1.9 mL, 1.5 Eq.) and the reaction was stirred at rt for 17 h. After reaction completion (TLC control) the reaction was diluted with EtOAc (50 mL) and washed with water (3 x 30 mL) and saturated brine, was dried over sodium sulfate, filtered, and concentrated under reduced pressure to afford the title compound **1f** as a brown solid (209 mg, 96%), which was used in the next step without further purification. ^{1}**H NMR** (400 MHz, DMSO-d_6) δ 11.83 (s, 1H), 9.80 (s, 1H), 7.35 (dt, *J* = 9.0, 0.8 Hz, 1H), 7.28 (dd, *J* = 2.2, 1.0 Hz, 1H), 7.18 (dd, *J* = 2.5, 0.8 Hz, 1H), 6.99 (dd, *J* = 9.0, 2.5 Hz, 1H), 3.77 (s, 3H).

Synthesis of compound **3m**

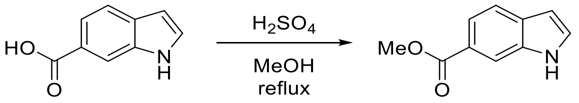

3j 3k

Methyl 1*H*-indole-6-carboxylate (3k)

To a solution of indole-6-carboxylic acid **3j** (1 g, 6.7 mmol, 1 Eq.) in MeOH (20 mL, 0.34 M) was added concentrated H_2SO_4 (188 µL, 3.4 mmol, 0.5 Eq.) and the reaction was stirred at reflux temperature for 8 h. After reaction completion (TLC control) the reaction was allowed to cool down to rt and it was quenched by addition of saturated aqueous $NaHCO_3$ until pH > 7. The aqueous layer was extracted with DCM (3 x 100 mL), and the organic layers were combined, dried over sodium sulfate, filtered, and concentrated under reduced pressure to afford the title compound **3k** as a light yellow solid (0.9 g, 76%), which was used without further purification. ^{1}**H NMR** (400 MHz, $CDCl_3$) δ 8.44 (s, 1H), 8.17 (dt, *J* = 1.6, 0.8 Hz, 1H), 7.82 (dd, *J* = 8.4, 1.5 Hz, 1H), 7.66 (dt, *J* = 8.3, 0.8 Hz, 1H), 7.38 (dd, *J* = 3.2, 2.5 Hz, 1H), 6.61 (ddd, *J* = 3.1, 2.0, 1.0 Hz, 1H), 3.94 (s, 3H).

Synthesis of compounds **Ib-d**

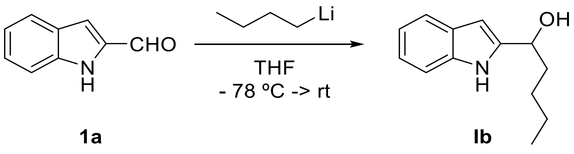

1a Ib

1-(1*H*-Indol-2-yl)pentan-1-ol (Ib)

To a – 78 ºC (acetone / dry ice bath) solution of **1a** (145 mg, 1 mmol, 1 eq.) in THF (7 mL, 0.15 M) was slowly added a 2.5 M *n*-BuLi solution in hexanes (0.9 mL, 2 mmol, 2 eq.). The reaction was allowed to warm to rt and stirred for 1 h, until full consumption of **1a** (TLC control). The reaction was quenched by addition of sat. NH_4Cl and extracted with EtOAc. The organic layer was washed with water and saturated brine, dried over sodium sulfate, filtered, and concentrated under reduced pressure to give the title compound **Ib** as a pale brown solid (203 mg, 98%), which was used without further purification. ^{1}**H NMR** (400 MHz, $CDCl_3$) δ 8.35 (br s, 1H), 7.57 (dq, *J* = 7.8, 0.9 Hz, 1H), 7.35 (dq, *J* = 8.1, 0.9 Hz, 1H), 7.17 (ddd, *J* = 8.2, 7.1, 1.3 Hz, 1H), 7.10 (ddd, *J* = 8.0, 7.0, 1.1 Hz, 1H), 6.36 (dt, *J* = 1.9, 0.8 Hz, 1H), 4.90 (td, *J* = 6.6, 4.1 Hz, 1H), 1.96 – 1.86 (m, 3H), 1.51 – 1.30 (m, 4H), 0.96 – 0.86 (m, 3H).

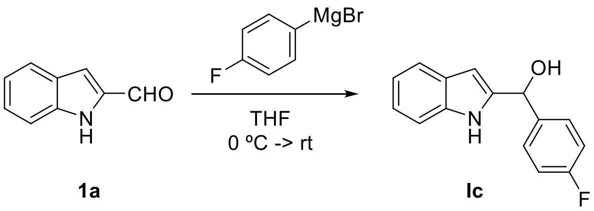

1a Ic

Exact Mass: 241,09

(4-Fluorophenyl)(1*H*-indol-2-yl)methanol (Ic)

To a – 0 ºC (ice bath) solution of **1a** (145 mg, 1 mmol, 1 eq.) in THF (7 mL, 0.15 M) was slowly added a 1 M (4-fluorophenyl)magnesium bromide solution in hexanes (3 mL, 2.2 mmol, 2.2 eq.). The reaction was allowed to warm to rt and stirred for 3 h. The reaction was quenched by addition of sat. NH_4Cl and extracted with EtOAc. The organic layer was washed with water and saturated brine, dried

over sodium sulfate, filtered, and concentrated under reduced pressure to give the title compound **Ic** as a pale brown solid. Formation of compound **Ie** was confirmed by LC-MS (detected *m/z* 242 [MH+], > 80% purity), and the crude product was used without further purification.

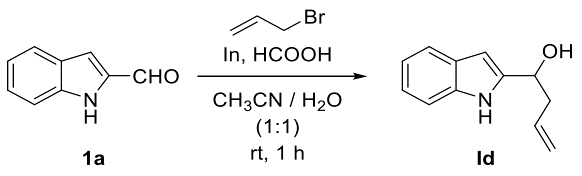

1-(1*H*-Indol-2-yl)but-3-en-1-ol (Id)

To a solution of **1a** (2 g, 13.8 mmol, 1 eq.), allyl bromide (1.3 mL, 15.2 mmol, 1.1 eq.), and formic acid (64 µL, 1.4 mmol, 0.1 eq.) in CH3CN (30 mL) / H2O (30 mL) (1:1, 0.25 M) under argon atmosphere was added indium (1.9 g, 16.5 mmol, 1.2 eq.). The reaction was stirred at rt for 1 h, until full consumption of **1a** (TLC control). The mixture was diluted with DCM, and the organic layer was washed with water and saturated brine, dried over sodium sulfate, filtered, and concentrated under reduced pressure to give the title compound **Id** as a pale brown oil (2.6 g, 100%), which was used without further purification. **^{1}H NMR** (400 MHz, DMSO-d_6) δ 10.93 (s, 1H), 7.43 (dt, J = 7.8, 0.9 Hz, 1H), 7.31 (dq, J = 8.0, 0.9 Hz, 1H), 7.00 (ddd, J = 8.1, 7.0, 1.3 Hz, 1H), 6.92 (ddd, J = 8.0, 7.0, 1.1 Hz, 1H), 6.24 (dt, J = 1.8, 0.8 Hz, 1H), 5.81 (ddt, J = 17.1, 10.2, 6.9 Hz, 1H), 5.39 (d, J = 4.9 Hz, 1H), 5.07 (dt, J = 17.2, 1.9 Hz, 1H), 4.99 (ddt, J = 10.2, 2.3, 1.2 Hz, 1H), 4.75 (td, J = 6.7, 5.4 Hz, 1H), 2.58 – 2.52 (m, 2H).

4.3. Characterization Data of Final Adducts

4.3.1. 6-ICZs (Series 4-5)

2-(5,11-Dihydroindolo[3,2-*b*]carbazol-6-yl)-3-hydroxy-5,5-dimethylcyclohex-2-en-1-one (4a)

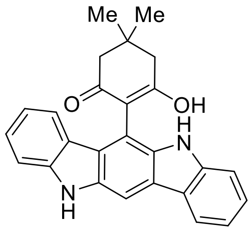

Following the *General Procedure A*, compound **4a** was obtained as an orange powder (83 mg, 42%) from **1a** (145 mg, 1.0 mmol, 2 eq.), **2a** (70 mg, 0.5 mmol, 1.0 eq), *L*-proline (6 mg, 0.05 mmol, 0.1 eq.), and TFA (8 µL, 0.1 mmol, 0.2 eq.) in EtOH (2 mL, 0.25 M) after stirring for 30 min. The product precipitated from the reaction mixture and was isolated by filtration under vacuum. **^{1}H NMR** (400 MHz, DMSO-*d$_6$*) δ 10.97 (s, 1H), 10.51 (s, 1H), 10.09 (s, 1H), 8.17 (d, *J* = 7.7 Hz, 1H), 8.02 (s, 1H), 7.80 (d, *J* = 7.8 Hz, 1H), 7.47 (dt, *J* = 8.1, 1.0 Hz, 1H), 7.42 (dt, *J* = 8.1, 0.9 Hz, 1H), 7.35 (ddd, *J* = 8.2, 7.1, 1.2 Hz, 1H), 7.30 (ddd, *J* = 8.1, 7.1, 1.2 Hz, 1H), 7.10 (ddd, *J* = 7.9, 7.0, 1.0 Hz, 1H), 7.00 (ddd, *J* = 8.1, 7.1, 1.1 Hz, 1H), 2.85 – 2.40 (m, 4H, overlapped with residual solvent signal), 1.36 (s, 3H), 1.32 (s, 3H). **^{13}C NMR** (101 MHz, DMSO-*d$_6$*) δ 195.80, 172.20, 141.14, 140.94, 135.41, 134.65, 125.13, 124.64, 123.13, 122.87, 121.95, 121.77, 121.70, 120.04, 117.36, 117.18, 110.52, 110.27, 110.02, 98.92, 50.82, 43.28, 31.56, 29.65, 28.40. **HRMS:** m/z calcd for $C_{26}H_{23}N_2O_2^+$ $[M+H]^+$: 395.1754; found 395.1753.

3-(5,11-Dihydroindolo[3,2-b]carbazol-6-yl)-4-hydroxyfuran-2(5H)-one (4b)

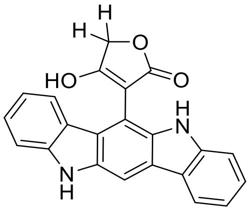

Following the *General Procedure A*, compound **4b** was obtained as dark brown powder (151 mg, 85%) from **1a** (145 mg, 1.0 mmol, 2 eq.), **2b** (50 mg, 0.5 mmol, 1.0 eq), *L*-proline (6 mg, 0.05 mmol, 0.1 eq.), and TFA (8 µL, 0.1 mmol, 0.2 eq.) in EtOH (2 mL, 0.25 M). The product precipitated from the reaction mixture and was isolated by filtration under vacuum. **^{1}H NMR** (400 MHz, DMSO-*d$_6$*) δ 12.43 (s, 1H), 11.11 (s, 1H), 10.74 (s, 1H), 8.22 (d, *J* = 7.7 Hz, 1H), 8.14 (s, 1H), 7.82 (d, *J* = 7.8 Hz, 1H), 7.49 – 7.44 (m, 2H, overlapped signals), 7.42 – 7.33 (m, 2H, overlapped signals), 7.17 – 7.11 (m, 1H), 7.09 – 7.02 (m, 1H), 5.16 (d, *J* = 16.0 Hz, 1H), 4.95 (d, *J* = 15.9 Hz, 1H). **^{13}C NMR** (101 MHz, DMSO-*d$_6$*) δ 176.22, 173.14, 141.24, 140.94, 135.19, 134.83, 125.50, 125.14, 122.60, 122.52, 122.01, 121.82, 121.39, 120.32, 117.64, 117.58, 110.47, 110.23, 104.39, 100.29, 96.58, 67.50. **HRMS:** m/z calcd for $C_{22}H_{15}N_2O_3^+$ $[M+H]^+$: 355.1077; found 355.1068.

3-(5,11-Dihydroindolo[3,2-*b*]carbazol-6-yl)-4-hydroxy-2*H*-chromen-2-one (4c)

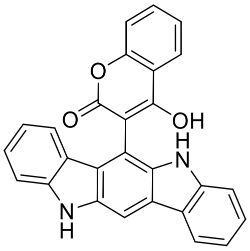

Following the *General Procedure A*, compound **4c** was obtained as dark red powder (140 mg, 97%) from **1a** (100 mg, 0.7 mmol, 2 eq.), **2c** (56 mg, 0.35 mmol, 1.0 eq), *L*-proline (4 mg, 0.035 mmol, 0.1 eq.), and TFA (5.5 µL, 0.07 mmol, 0.2 eq.) in EtOH (1.5 mL, 0.25 M). The product precipitated from the reaction mixture and was isolated by filtration under vacuum. **^{1}H NMR** (400 MHz, DMSO-*d$_6$*) δ 11.30 (br s, 1H), 11.12 (s, 1H), 10.80 (s, 1H), 8.23 (d, *J* = 7.8 Hz, 1H), 8.17 (s, 1H), 8.06 (dd, *J* = 7.8, 1.6 Hz, 1H), 7.77 (ddd, *J* = 8.6, 7.3, 1.6 Hz, 1H), 7.61 – 7.53 (m, 2H, overlapped signals), 7.51 – 7.42 (m, 2H), 7.42 – 7.31 (m, 2H), 7.30 (ddd, *J* = 8.2, 7.1, 1.2 Hz, 1H), 7.13 (ddd, *J* = 8.0, 6.2, 1.9 Hz, 1H), 6.93 (ddd, *J* = 8.0, 7.1, 1.0 Hz, 1H). **^{13}C NMR** (101 MHz, DMSO-*d$_6$*) δ 161.91, 153.67, 141.69, 135.89, 135.64, 132.86, 125.85, 125.51, 124.53, 124.40, 123.27, 123.10, 122.86, 122.13, 121.45, 120.71, 118.10, 118.04, 116.87, 110.85, 110.82, 101.10. **HRMS:** m/z calcd for $C_{27}H_{15}N_2O_3^-$ $[M-H]^-$: 415.1088; found 415.1091.

2-(5,11-Dimethyl-5,11-dihydroindolo[3,2-*b*]carbazol-6-yl)-3-hydroxy-5,5-dimethylcyclohex-2-en-1-one (4d)

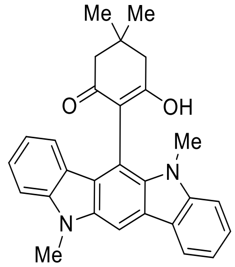

Following the *General Procedure A*, compound **4d** was obtained as a yellow solid (130 mg, 41%) from **1c** (254 mg, 1.6 mmol, 2 eq.), **2a** (112 mg, 0.8 mmol, 1.0 eq), *L*-proline (9 mg, 0.08 mmol, 0.1 eq.), and TFA (18 µL, 0.16 mmol, 0.2 eq.) in EtOH (3.2 mL, 0.25 M) after silica gel flash chormatography purification (EtOAc / hexane gradient from 0:100 to 50:50 v/v). **^{1}H NMR** (400 MHz, DMSO-d_6) δ 10.67 (br s, 1H), 8.27 (d, *J* = 8.3 Hz, 2H), 8.26 (s, 1H), 7.86 (dt, *J* = 7.9, 1.0 Hz, 1H), 7.56 – 7.48 (m, 2H, overlapped signals), 7.47 – 7.42 (m, 1H), 7.43 – 7.38 (m, 1H), 7.19 (ddd, *J* = 7.9, 6.9, 1.1 Hz, 1H), 7.09 (ddd, *J* = 8.1, 7.1, 1.1 Hz, 1H), 3.96 (s, 3H), 3.85 (s, 3H), 2.80 – 2.52 (m, 4H), 1.34 (s, 3H), 1.28 (s, 3H). **^{13}C NMR** (101 MHz, DMSO-d_6) δ 142.72, 142.24, 137.06, 135.22, 126.08, 125.45, 123.31, 123.16, 122.55, 122.20, 121.96, 120.32, 118.37, 118.18, 112.29, 111.33, 109.04, 108.69, 98.38, 40.67, 32.11, 31.60, 30.15, 29.58, 28.91. **HRMS:** m/z calcd for $C_{28}H_{27}N_2O_2^+$ [M+H]$^+$: 423.2067; found 423.2074.

6-(5-Methoxy-1*H*-indol-3-yl)-5,11-dihydroindolo[3,2-*b*]carbazole (5a)

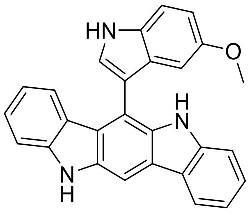

Following the *General Procedure B*, compound **5a** was obtained as a light brown powder (140 mg, 70%) from from **1a** (145 mg, 1.0 mmol, 2 eq.), **3b** (74 mg, 0.5 mmol, 1.0 eq), 5M NaOH$_{aq.}$ (1 mL, 5 mmol, 10 eq.) in EtOH (1 mL, final concentration 0.25 M) after silica gel flash chormatography purification (DCM / hexane gradient from 0:100 to 40:60 v/v). **^{1}H NMR** (400 MHz, DMSO-d_6) δ 11.43 (d, *J* = 2.5 Hz, 1H), 11.09 (s, 1H), 10.33 (s, 1H), 8.22 (d, *J* = 7.4 Hz, 1H), 8.13 (s, 1H), 7.67 (d, *J* = 2.5 Hz, 1H), 7.52 (dd, *J* = 8.8, 0.5 Hz, 1H), 7.46 – 7.39 (m, 2H, overlapped signals), 7.32 (ddd, *J* = 8.2, 7.0, 1.2 Hz, 1H), 7.24 (ddd, *J* = 8.2, 7.0, 1.2 Hz, 1H), 7.11 (ddd, *J* = 8.0, 7.0, 1.0 Hz, 1H), 7.07 (d, *J* = 8.1 Hz, 1H), 6.85 (dd, *J* = 8.8, 2.5 Hz, 1H), 6.73 (ddd, *J* = 8.1, 7.1, 1.1 Hz, 1H), 6.46 (d, *J* = 2.4 Hz, 1H), 3.42 (s, 3H). **^{13}C NMR** (101 MHz, DMSO-d_6) δ 153.89, 141.73, 141.69, 135.97, 135.45, 132.17, 127.66, 125.99, 125.69, 125.27, 123.22, 123.19, 122.62, 122.44, 121.91, 120.62, 118.00, 117.42, 113.07, 112.16, 111.48, 111.34, 110.50, 110.45, 101.66, 99.51, 55.63. **HRMS:** m/z calcd for $C_{27}H_{20}N_3O^+$ [M+H]$^+$: 402.1601; found 402.1606.

6-(5-Bromo-1*H*-indol-3-yl)-5,11-dihydroindolo[3,2-*b*]carbazole (5b)

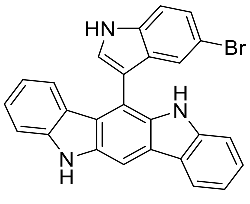

Following the *General Procedure A*, compound **5b** was obtained as a yellow powder (155 mg, 42%), from **1a** (290 mg, 2.0 mmol, 2 eq.), **3a** (196 mg, 1 mmol, 1 eq), *L*-proline (12 mg, 0.1 mmol, 0.1 eq.), and TFA (16 µL, 0.2 mmol, 0.2 eq.) in EtOH (4 mL, 0.25 M) after silica gel flash chormatography purification (DCM / hexane gradient from 0:100 to 50:50 v/v). The title compound **5b** crystallized from the column fractions containing *ca.* 50:50 DCM / hexane mixture (v/v) The crystals were filtered washing with DCM, dried under high vacuum and used for NMR characterization and X-Ray crystallography (see section 8.1). The residual DCM present in the sample and observed in the NMR copies could not be removed even under high vacuum. **^{1}H NMR** (400 MHz, DMSO-d_6) δ 11.80 (d, *J* = 2.5 Hz, 1H), 11.15 (s, 1H), 10.40 (s, 1H), 8.23 (d, *J* = 7.8 Hz, 1H), 8.16 (s, 1H), 7.80 (d, *J* = 2.4 Hz, 1H), 7.61 (dd, *J* = 8.6, 0.6 Hz, 1H), 7.44 (dt, *J* = 8.0, 0.9 Hz, 1H), 7.41 (dt, *J* = 8.1, 1.0 Hz, 1H), 7.33 (ddd, *J* = 8.2, 7.0, 1.2 Hz, 2H), 7.31 (dd, *J* = 8.6, 2.0 Hz, 1H), 7.25 (ddd, *J* = 8.2, 7.0, 1.2 Hz, 1H), 7.12 (ddd, *J* = 8.0, 7.0, 1.1 Hz, 1H), 7.07 (d, *J* = 1.9 Hz, 1H), 6.97 (d, *J* = 8.1 Hz, 1H), 6.74 (ddd, *J* = 8.0, 7.1, 1.0 Hz, 1H). **^{13}C NMR** (101 MHz, DMSO-d_6) δ 141.75, 141.70, 135.88, 135.78, 135.55, 129.23, 127.08, 125.80, 125.41, 124.35, 123.18, 122.99, 122.71, 121.99, 121.88, 121.82, 120.70, 118.10, 117.59, 114.48, 112.06, 111.42, 110.69, 110.39, 110.09, 99.99. **HRMS:** m/z calcd for $C_{26}H_{17}N_3Br^+$ [M+H]$^+$: 450.0600, 452.0580; found 450.0613, 452.0592.

6-(1*H*-Indol-3-yl)-5,11-dihydroindolo[3,2-*b*]carbazole (5c)

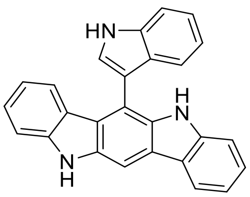

Following the *General Procedure A*, compound **5c** was obtained as a light brown powder (45 mg, 23%) from **1a** (145 mg, 1.0 mmol, 2 eq.), **3d** (59 mg, 0.5 mmol, 1.0 eq), *L*-proline (6 mg, 0.05 mmol, 0.1 eq.), and TFA (8 μL, 0.1 mmol, 0.2 eq.) in EtOH (2 mL, 0.25 M) after silica gel flash chromatography purification (DCM / hexane gradient from 10:90 to 75:25 v/v). **^{1}H NMR** (400 MHz, DMSO-*d$_6$*) δ 11.56 (d, *J* = 2.5 Hz, 1H), 11.10 (s, 1H), 10.34 (s, 1H), 8.22 (d, *J* = 7.8 Hz, 1H), 8.13 (s, 1H), 7.71 (d, *J* = 2.4 Hz, 1H), 7.62 (dt, *J* = 8.3, 0.9 Hz, 1H), 7.42 (dt, *J* = 3.7, 0.9 Hz, 1H), 7.40 (dt, *J* = 3.7, 0.9 Hz, 1H), 7.32 (ddd, *J* = 8.2, 7.0, 1.2 Hz, 1H), 7.25 – 7.16 (m, 2H, overlapped signals), 7.11 (ddd, *J* = 7.9, 7.0, 1.1 Hz, 1H), 7.03 – 6.97 (m, 2H, overlapped signals), 6.91 (ddd, *J* = 7.9, 6.9, 1.0 Hz, 1H), 6.68 (ddd, *J* = 8.0, 7.1, 1.0 Hz, 1H). **^{13}C NMR** (101 MHz, DMSO-*d$_6$*) δ 141.27, 141.19, 136.56, 135.47, 135.03, 126.82, 125.19, 124.81, 124.76, 122.73, 122.14, 121.85, 121.50, 121.36, 120.15, 119.57, 118.94, 117.51, 116.94, 111.88, 110.97, 110.75, 110.17, 110.02, 99.08. **HRMS:** m/z calcd for $C_{26}H_{18}N_3^+$ [M+H]$^+$: 372.1495; found 372.1487.

6-(1-Methyl-1*H*-indol-3-yl)-5,11-dihydroindolo[3,2-*b*]carbazole (5d)

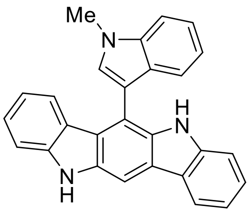

Following the *General Procedure A*, compound **5d** was obtained as an off-white powder (60 mg, 31%) from **1a** (145 mg, 1 mmol, 2 eq.), **3e** (66 mg, 0.5 mmol, 1 eq.), *L*-proline (6 mg, 0.05 mmol, 0.1 eq.), and TFA (8 μL, 0.1 mmol, 0.2 eq.) in EtOH (2 mL, 0.25 M) after silica gel flash chromatography purification (DCM / hexane gradient from 0:100 to 50:50 v/v). **^{1}H NMR** (400 MHz, DMSO-*d$_6$*) δ 11.11 (s, 1H), 10.38 (s, 1H), 8.22 (d, *J* = 7.8 Hz, 1H), 8.13 (s, 1H), 7.73 (s, 1H), 7.66 (dt, *J* = 8.3, 0.9 Hz, 1H), 7.45 – 7.37 (m, 2H, overlapped signals), 7.32 (ddd, *J* = 8.2, 7.0, 1.2 Hz, 1H), 7.29 – 7.19 (m, 2H, overlapped signals), 7.12 (ddd, *J* = 8.0, 7.0, 1.1 Hz, 1H), 7.04 – 6.98 (m, 2H, overlapped signals), 6.95 (ddd, *J* = 7.9, 6.8, 0.9 Hz, 1H), 6.68 (ddd, *J* = 8.1, 7.1, 1.0 Hz, 1H), 4.04 (s, 3H). **^{13}C NMR** (101 MHz, DMSO-*d$_6$*) δ 141.76, 141.62, 137.48, 135.98, 135.52, 129.67, 127.62, 125.74, 125.28, 123.22, 123.11, 122.62, 122.41, 121.93, 121.82, 120.67, 120.33, 119.56, 118.04, 117.46, 111.33, 110.78, 110.61, 110.52, 109.82, 99.64, 33.31. **HRMS:** m/z calcd for $C_{27}H_{19}N_3^{\cdot+}$ [M$^\cdot$]$^+$: 385.1573; found 385.1576.

6-(2-Methyl-1*H*-indol-3-yl)-5,11-dihydroindolo[3,2-*b*]carbazole (5e)

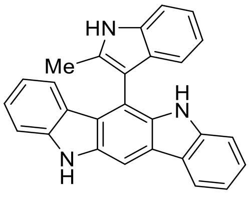

Following the *General Procedure A*, compound **5e** was obtained as a light brown powder (1.65 g, 86%) from **1a** (1.46 g, 10.06 mmol, 2 eq.), **3f** (0.66 g, 5.03 mmol, 1.0 eq), *L*-proline (58 mg, 0.5 mmol, 0.1 eq.), and TFA (77 μL, 1 mmol, 0.2 eq.) in EtOH (10 mL, 0.5 M) after silica gel flash chromatography purification (DCM / hexane gradient from 0:100 to 40:60 v/v). **^{1}H NMR** (400 MHz, DMSO-*d$_6$*) δ 11.40 (s, 1H), 11.10 (s, 1H), 10.29 (s, 1H), 8.22 (d, *J* = 7.8 Hz, 1H), 8.14 (s, 1H), 7.50 (d, *J* = 8.1 Hz, 1H), 7.46 – 7.37 (m, 2H, overlapped signals), 7.31 (ddd, *J* = 8.2, 7.0, 1.2 Hz, 1H), 7.23 (ddd, *J* = 8.2, 7.0, 1.2 Hz, 1H), 7.11 (m, 2H), 6.94 (d, *J* = 7.9 Hz, 1H), 6.91 – 6.80 (m, 2H), 6.71 (ddd, *J* = 8.0, 7.0, 1.0 Hz, 1H), 2.25 (s, 3H). **^{13}C NMR** (101 MHz, DMSO-*d$_6$*) δ 141.73, 141.70, 136.45, 135.88, 135.41, 134.19, 128.33, 125.64, 125.23, 123.25, 123.23, 122.53, 122.20, 122.10, 120.92, 120.62, 119.20, 119.06, 117.95, 117.59, 111.40, 111.35, 110.75, 110.48, 107.78, 99.60, 12.89. **HRMS:** m/z calcd for $C_{27}H_{20}N_3^+$ [M+H]$^+$: 386.1652; found 386.1600.

6-(2-Phenyl-1*H*-indol-3-yl)-5,11-dihydroindolo[3,2-*b*]carbazole (5f)

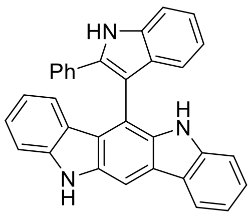

Following the *General Procedure A*, compound **5f** was obtained as a yellow solid (75 mg, 49%), from **1a** (145 mg, 1.0 mmol, 2 eq.), **3g** (100 mg, 0.5 mmol, 1.0 eq), *L*-proline (6 mg, 0.05 mmol, 0.1 eq.), and TFA (8 μL, 0.1 mmol, 0.2 eq.) in EtOH (2 mL, 0.25 M) after silica gel flash chromatography purification (EtOAc / hexane gradient from 0:100 to 80:20 v/v). **^{1}H NMR** (400 MHz, DMSO-d_6) δ 11.87 (s, 1H), 11.12 (s, 1H), 10.37 (s, 1H), 8.23 (dt, *J* = 7.9, 0.9 Hz, 1H), 8.19 (d, *J* = 0.5 Hz, 1H), 7.63 (dt, *J* = 8.2, 0.9 Hz, 1H), 7.51 – 7.45 (m, 2H), 7.39 (dt, *J* = 8.0, 0.9 Hz, 1H), 7.33 – 7.25 (m, 2H, overlapped signals), 7.22 – 7.15 (m, 2H, overlapped signals), 7.14 – 7.01 (m, 4H, overlapped signals), 6.89 – 6.80 (m, 3H, overlapped signals), 6.65 (ddd, *J* = 8.0, 7.1, 1.0 Hz, 1H). **^{13}C NMR** (101 MHz, DMSO-d_6) δ 141.66, 137.24, 135.84, 135.62, 134.98, 133.28, 129.42, 128.83, 127.54, 126.71, 125.68, 125.32, 123.23, 122.68, 122.52, 122.33, 121.57, 120.69, 119.77, 119.56, 117.99, 117.77, 112.00, 111.34, 110.97, 110.59, 108.34, 100.21. **HRMS:** m/z calcd for $C_{32}H_{20}N_3^-$ [M-H]$^-$: 446.1663; found 446.1656.

(3-(5,11-Dihydroindolo[3,2-*b*]carbazol-6-yl)-1*H*-indol-2-yl)methanol (5g)

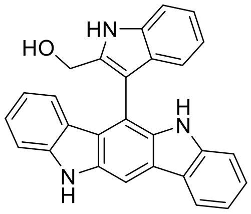

Following the *General Procedure B*, compound **5g** was obtained as an off-white solid (180 mg, 45%) from **1a** (290 mg, 2 mmol, 2 eq.), **3h** (147 mg, 1 mmol, 1 eq.), 5M $NaOH_{aq.}$ (2 mL, 10 mmol, 10 eq.) in EtOH (2 mL, final concentration 0.25 M) after silica gel flash chromatography purification (DCM / hexane gradient from 50:50 to 100:0 v/v). **^{1}H NMR** (400 MHz, DMSO-d_6) δ 11.52 (s, 1H), 11.11 (s, 1H), 10.23 (s, 1H), 8.22 (d, *J* = 7.8 Hz, 1H), 8.15 (s, 1H), 7.57 (dt, *J* = 8.2, 0.9 Hz, 1H), 7.44 – 7.37 (m, 2H, overlapped signals), 7.32 (ddd, *J* = 8.1, 7.0, 1.2 Hz, 1H), 7.22 (ddd, *J* = 8.2, 7.1, 1.2 Hz, 1H), 7.16 – 7.10 (m, 2H, overlapped signals), 6.93 – 6.88 (m, 2H, overlapped signals), 6.85 (ddd, *J* = 7.9, 6.7, 1.0 Hz, 1H), 6.68 (ddd, *J* = 8.0, 7.1, 1.0 Hz, 1H), 5.16 (dd, *J* = 5.5, 4.6 Hz, 1H), 4.57 (dd, *J* = 13.2, 4.6 Hz, 1H), 4.43 (dd, *J* = 13.1, 5.4 Hz, 1H). **^{13}C NMR** (101 MHz, DMSO-d_6) δ 141.73, 141.64, 137.48, 136.79, 135.89, 135.45, 127.76, 125.71, 125.26, 123.26, 123.16, 122.56, 122.34, 122.23, 121.59, 120.66, 119.70, 119.29, 118.07, 117.54, 112.05, 111.37, 110.45, 110.26, 107.25, 99.76, 56.25. **HRMS:** m/z calcd for $C_{27}H_{19}N_3O^{\cdot+}$ [M$^{\cdot}$]$^+$: 401.1523; found 401.1524.

6-(4-Methyl-1*H*-indol-3-yl)-5,11-dihydroindolo[3,2-*b*]carbazole (5h)

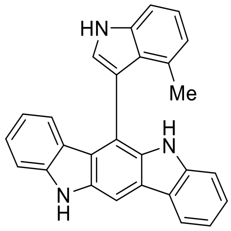

Following the *General Procedure A*, compound **5h** was obtained as a pale brown solid (300 mg, 38%) from **1a** (580 mg, 4 mmol, 2 eq.), **3i** (262 mg, 2 mmol, 1 eq.), *L*-proline (23 mg, 0.2 mmol, 0.1 eq.), and TFA (31 μL, 0.4 mmol, 0.2 eq.) in EtOH (8 mL, 0.25 M) after silica gel flash chromatography purification (DCM / hexane gradient from 0:100 to 50:50 v/v). **^{1}H NMR** (400 MHz, DMSO-d_6) δ 11.46 (s, 1H), 11.04 (s, 1H), 10.36 (s, 1H), 8.21 (d, *J* = 7.8 Hz, 1H), 8.11 (s, 1H), 7.48 (d, *J* = 2.4 Hz, 1H), 7.45 – 7.37 (m, 3H, overlapped signals), 7.31 (ddd, *J* = 8.2, 7.0, 1.2 Hz, 1H), 7.21 (ddd, *J* = 8.2, 7.0, 1.3 Hz, 1H), 7.13 – 7.04 (m, 3H, overlapped signals), 6.84 (d, *J* = 7.9 Hz, 1H), 6.71 – 6.63 (m, 2H, overlapped signals), 1.65 (s, 3H). **^{13}C NMR** (101 MHz, DMSO-d_6) δ 141.76, 141.63, 137.25, 136.41, 135.48, 130.58, 126.45, 125.60, 125.26, 124.73, 123.75, 123.38, 123.21, 122.22, 121.82, 121.51, 120.66, 120.49, 117.92, 117.62, 113.27, 111.37, 110.79, 110.57, 110.21, 99.63, 18.62. **HRMS:** m/z calcd for $C_{27}H_{20}N_3^+$ [M+H]$^+$: 386.1652; found 386.1664.

6-(7-Methyl-1*H*-indol-3-yl)-5,11-dihydroindolo[3,2-*b*]carbazole (5i)

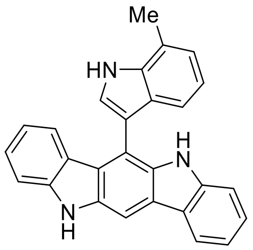

Following the *General Procedure B*, compound **5i** was obtained as white powder (300 mg, 39%), from **1a** (580 mg, 4 mmol, 2 eq.), **3m** (262 mg, 2 mmol, 1.0 eq), 5M $NaOH_{aq.}$ (4 mL, 20 mmol, 10 eq.) in EtOH (4 mL, final concentration 0.25 M) after silica gel flash chromatography purification (DCM / hexane gradient from 20:80 to 80:20 v/v). **^{1}H NMR** (400 MHz, DMSO-*d$_6$*) δ 11.54 (d, *J* = 2.5 Hz, 1H), 11.09 (s, 1H), 10.32 (s, 1H), 8.24 – 8.19 (m, 1H), 8.12 (d, *J* = 0.5 Hz, 1H), 7.69 (d, *J* = 2.5 Hz, 1H), 7.44 – 7.38 (m, 2H, overlapped signals), 7.31 (ddd, *J* = 8.2, 7.0, 1.2 Hz, 1H), 7.22 (ddd, *J* = 8.2, 7.1, 1.2 Hz, 1H), 7.11 (ddd, *J* = 7.9, 7.0, 1.1 Hz, 1H), 7.05 – 7.01 (m, 1H), 6.99 (ddd, *J* = 5.7, 2.4, 1.0 Hz, 1H), 6.87 – 6.79 (m, 2H, overlapped signals), 6.69 (ddd, *J* = 8.0, 7.1, 1.0 Hz, 1H), 2.68 – 2.61 (m, 3H). **^{13}C NMR** (101 MHz, DMSO-*d$_6$*) δ 141.74, 141.66, 136.54, 135.95, 135.52, 127.02, 125.65, 125.23, 124.96, 123.24, 123.20, 122.59, 122.37 (2 overlapped signals), 121.95, 121.44, 120.62, 119.63, 117.97, 117.72, 117.42, 111.46, 111.43, 111.12, 110.48, 99.52, 17.47. **HRMS:** m/z calcd for $C_{27}H_{19}N_3^{\cdot+}$ $[M^{\cdot}]^+$: 385.1573; found 385.1575.

5,11-Dimethyl-6-(2-methyl-1*H*-indol-3-yl)-5,11-dihydroindolo[3,2-*b*]carbazole (5j)

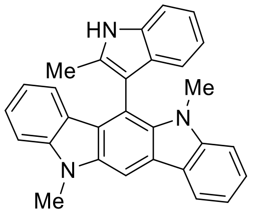

Following the *General Procedure A*, compound **5j** was obtained as a pale yellow solid (150 mg, 72%), from **1c** (160 mg, 1 mmol, 2 eq.), **3f** (66 mg, 0.5 mmol, 1.0 eq), *L*-proline (6 mg, 0.05 mmol, 0.1 eq.), and TFA (8 μL, 0.1 mmol, 0.2 eq.) in EtOH (2 mL, 0.25 M) after silica gel flash chromatography purification (DCM / hexane gradient from 0:100 to 20:80 v/v). **^{1}H NMR** (400 MHz, DMSO-*d$_6$*) δ 11.46 (s, 1H), 8.44 (s, 1H), 8.38 (d, *J* = 7.7 Hz, 1H), 7.59 – 7.53 (m, 2H, overlapped signals), 7.53 – 7.45 (m, 2H, overlapped signals), 7.36 (ddd, *J* = 8.3, 7.0, 1.3 Hz, 1H), 7.27 (ddd, *J* = 7.9, 6.1, 1.9 Hz, 1H), 7.16 (ddd, *J* = 8.2, 6.4, 1.8 Hz, 1H), 6.97 – 6.88 (m, 2H, overlapped signals), 6.79 (ddd, *J* = 8.0, 7.0, 1.0 Hz, 1H), 6.71 (ddd, *J* = 7.9, 1.3, 0.6 Hz, 1H), 4.06 (s, 3H), 3.37 (s, 3H), 2.20 (s, 3H). **^{13}C NMR** (101 MHz, DMSO-*d$_6$*) δ 143.08, 142.36, 136.83, 135.98, 135.60, 134.15, 129.87, 126.19, 125.57, 123.36, 123.03, 123.00, 122.75, 121.58, 121.21, 120.51, 119.66, 118.70, 118.58, 118.07, 111.47, 111.16, 109.24, 108.71, 108.44, 98.80, 30.82, 29.63, 12.43. **HRMS:** m/z calcd for $C_{29}H_{23}N_3^{\cdot+}$ $[M^{\cdot}]^+$: 413.1886; found 413.1889.

2,8-Difluoro-6-(2-methyl-1*H*-indol-3-yl)-5,11-dihydroindolo[3,2-*b*]carbazole (5k)

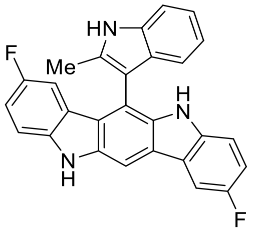

Following the *General Procedure A*, compound **5k** was obtained as a light green solid (80 mg, 38%), from **1d** (163 mg, 1 mmol, 2 eq.), **3f** (66 mg, 0.5 mmol, 1.0 eq), *L*-proline (6 mg, 0.05 mmol, 0.1 eq.), and TFA (8 μL, 0.1 mmol, 0.2 eq.) in EtOH (2 mL, 0.25 M) after silica gel flash chromatography purification (DCM / hexane gradient from 0:100 to 40:60 v/v). **^{1}H NMR** (400 MHz, DMSO-*d$_6$*) δ 11.47 (s, 1H), 11.20 (s, 1H), 10.37 (s, 1H), 8.18 (s, 1H), 8.09 (dd, *J* = 9.5, 2.6 Hz, 1H), 7.51 (dt, *J* = 8.2, 0.9 Hz, 1H), 7.44 – 7.35 (m, 2H, overlapped signals), 7.18 (ddd, *J* = 9.4, 8.7, 2.6 Hz, 1H), 7.15 – 7.07 (m, 2H, overlapped signals), 6.91 – 6.83 (m, 2H, overlapped signals), 6.54 (dd, *J* = 10.2, 2.7 Hz, 1H), 2.25 (s, 3H). **^{13}C NMR** (101 MHz, DMSO-*d$_6$*) δ 157.21 (d, *J* = 59.1 Hz), 154.92 (d, *J* = 57.8 Hz), 138.42, 138.35, 136.82, 136.41, 136.31, 134.33, 128.09, 123.45 (d, *J* = 9.6 Hz), 123.29 (d, *J* = 9.8 Hz), 122.94 (d, *J* = 4.0 Hz), 122.32 (d, *J* = 4.2 Hz), 121.12, 119.41, 118.95, 113.48 (d, *J* = 25.1 Hz), 112.95 (d, *J* = 25.1 Hz), 112.10 (d, *J* = 8.7 Hz), 111.47, 111.13 (d, *J* = 9.5 Hz), 111.08, 107.32 (d, *J* = 24.3 Hz), 107.15, 106.38 (d, *J* = 23.5 Hz), 100.34, 12.84. **^{19}F NMR** (376 MHz, DMSO-*d$_6$*) δ -126.11 (td, *J* = 9.6, 4.8 Hz), -126.41 (td, *J* = 9.7, 4.4 Hz). **HRMS:** m/z calcd for $C_{27}H_{17}F_2N_3^{\cdot+}$ $[M^{\cdot}]^+$: 421.1385; found 421.1384.

2,8-Dimethoxy-6-(2-methyl-1*H*-indol-3-yl)-5,11-dihydroindolo[3,2-*b*]carbazole (5l)

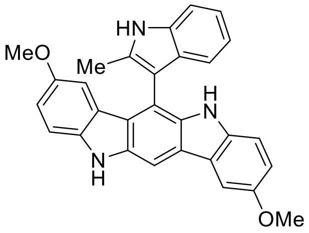

Following the *General Procedure A*, compound **5l** was obtained as a pale brown solid (55 mg, 45%), from **1f** (96 mg, 0.55 mmol, 2 eq.), **3f** (36 mg, 0.27 mmol, 1.0 eq), *L*-proline (3 mg, 0.03 mmol, 0.1 eq.), and TFA (4 μL, 0.05 mmol, 0.2 eq.) in EtOH (1.1 mL, 0.25 M) after silica gel flash chromatography purification (DCM / hexane gradient from 0:100 to 50:50 v/v). **^{1}H NMR** (400 MHz, DMSO-d_6) δ 11.39 (s, 1H), 10.82 (s, 1H), 10.07 (s, 1H), 8.08 (s, 1H), 7.78 (d, *J* = 2.5 Hz, 1H), 7.49 (dt, *J* = 8.0, 0.9 Hz, 1H), 7.31 (dd, *J* = 3.5, 0.5 Hz, 1H), 7.29 (dd, *J* = 3.6, 0.5 Hz, 1H), 7.09 (ddd, *J* = 8.2, 5.7, 2.5 Hz, 1H), 6.96 (dd, *J* = 8.7, 2.5 Hz, 1H), 6.90 – 6.85 (m, 3H, overlapped signals), 6.41 (d, *J* = 2.6 Hz, 1H), 3.88 (s, 3H), 3.26 (s, 3H), 2.26 (s, 3H). **^{13}C NMR** (101 MHz, DMSO-d_6) δ 152.81, 151.99, 136.81, 136.72, 136.52, 136.40, 135.86, 134.22, 128.33, 123.56, 123.50, 122.80, 122.21, 120.88, 119.32, 119.17, 114.91, 113.65, 112.00, 111.28, 110.70, 110.58, 107.74, 106.06, 103.64, 99.61, 56.18, 55.17, 12.97. **HRMS:** m/z calcd for $C_{29}H_{23}N_3O_2^{\cdot+}$ [M$^{\cdot}$]$^+$: 445.1785; found 445.1785.

3-(5,11-Dihidroindolo[3,2-*b*]carbazole-6-yl)-1*H*-indole-6-carboxylic acid (5m)

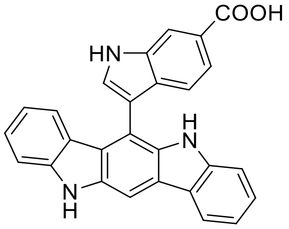

Following the *General Procedure A*, compound **5m** was obtained as a dark red powder (210 mg, 17%), from **1a** (870 mg, 6 mmol, 2 eq.), **3j** (483 mg, 3 mmol, 1.0 eq), *L*-proline (35 mg, 0.3 mmol, 0.1 eq.), and TFA (46 μL, 0.6 mmol, 0.2 eq.) in EtOH (12 mL, 0.25 M) after silica gel flash chromatography purification (EtOAc + 1% Et_3N / hexane gradient from 0:100 to 85:15 v/v). **^{1}H NMR** (400 MHz, DMSO-d_6) δ 12.53 (s, 1H), 11.96 (s, 1H), 11.14 (s, 1H), 10.41 (s, 1H), 8.28 (s, 1H), 8.23 (d, *J* = 7.8 Hz, 1H), 8.16 (s, 1H), 7.97 (d, *J* = 2.5 Hz, 1H), 7.54 (dd, *J* = 8.3, 1.5 Hz, 1H), 7.43 (d, *J* = 8.0 Hz, 1H), 7.40 (d, *J* = 8.0 Hz, 1H), 7.32 (t, *J* = 8.1 Hz, 1H), 7.23 (t, *J* = 8.2 Hz, 1H), 7.12 (t, *J* = 6.9 Hz, 1H), 7.06 (d, *J* = 8.3 Hz, 1H), 6.95 (d, *J* = 7.9 Hz, 1H), 6.70 (t, *J* = 7.5 Hz, 1H). **^{13}C NMR** (101 MHz, DMSO-d_6) δ 168.43, 141.31, 141.24, 135.92, 135.47, 135.05, 130.23, 128.62, 125.32, 124.89, 123.61, 122.72, 122.58, 122.25, 121.65, 121.42, 120.23, 120.04, 119.19, 117.63, 117.08, 114.05, 110.94, 110.74, 110.17, 109.90, 99.45. **HRMS:** m/z calcd for $C_{27}H_{18}N_3O_2^+$ [M+H]$^+$: 416.1394; found 416.1399.

A replicate of the reaction during longer time (24 hours) or lower temperature (50 ºC) resulted in similar final yields.

4.3.2. Knoevenagel Adducts and Bis- / Tris-indolyl Compounds (Series 6-7)

5-((1*H*-Indol-2-yl)methylene)-1,3-dimethylpyrimidine-2,4,6(1*H*,3*H*,5*H*)-trione (6a)

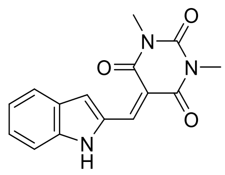

Following the *General Procedure A*, compound **6a** was obtained as a yellow powder (62 mg, 44%) from **1a** (348 mg, 2.4 mmol, 2 eq.), **2e** (193 mg, 1.2 mmol, 1 eq.), *L*-proline (14 mg, 0.12 mmol, 0.1 eq.), and TFA (18 μL, 0.24 mmol, 0.2 eq.) in EtOH (4.8 mL, 0.25 M) after silica gel flash chromatography purification (EtOAc + 1% Et_3N / hexane gradient from 0:100 to 85:15 v/v). **^{1}H NMR** (400 MHz, DMSO-*d₆*) δ 12.32 (s, 1H), 8.40 (s, 1H), 7.79 – 7.75 (m, 2H, overlapped signals), 7.73 (d, *J* = 8.1 Hz, 1H), 7.44 – 7.36 (m, 1H), 7.14 (t, *J* = 7.5 Hz, 1H), 3.28 (s, 3H, overlapped with H_2O signal), 3.26 (s, 3H). **HRMS:** m/z calcd for $C_{15}H_{14}N_3O_3^+$ [M+H]$^+$: 284.1030; found 284.1029. The ^{13}C NMR spectrum could not be recorded due to the low solubility of the compound in the tested solvents.

2-((1*H*-Indol-2-yl)methylene)-1*H*-indene-1,3(2*H*)-dione (6b)

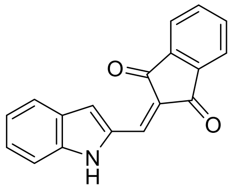

Following the *General Procedure A*, compound **6b** was obtained as a yellow powder (89 mg, 93%) from **1a** (102 mg, 0.7 mmol, 2 eq.), **2f** (51 mg, 0.35 mmol, 1 eq.), *L*-proline (4 mg, 0.035 mmol, 0.1 eq.), and TFA (5 μL, 0.07 mmol, 0.2 eq.) in EtOH (1.5 mL, 0.25 M) after silica gel flash chromatography purification (DCM / hexane gradient from 0:100 to 75:25 v/v). **^{1}H NMR** (400 MHz, DMSO-d_6) δ 12.20 (s, 1H), 8.04 – 8.00 (m, 1H), 7.98 – 7.95 (m, 3H), 7.92 (s, 1H), 7.76 – 7.71 (m, 3H), 7.40 (ddd, *J* = 8.4, 6.9, 1.1 Hz, 1H), 7.14 (ddd, *J* = 8.1, 6.9, 1.0 Hz, 1H). NMR data are consistent with those previously reported in the literature.[9]

2-((1*H*-Indol-3-yl)methylene)-5,5-dimethylcyclohexane-1,3-dione (6c)

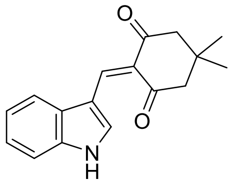

Following the *General Procedure A*, compound **6c** was obtained as a bright yellow powder (107 mg, 40%) from **1b** (290 mg, 2 mmol, 2 eq.), **2a** (140 mg, 1 mmol, 1 eq.), *L*-proline (23 mg, 0.1 mmol, 0.1 eq.), and TFA (16 μL, 0.2 mmol, 0.2 eq.) in EtOH (1.5 mL, 0.25 M). The product precipitated from the reaction mixture and was isolated by filtration under vacuum. **^{1}H NMR** (400 MHz, DMSO-d_6) δ 12.60 (s, 1H), 9.46 (s, 1H), 8.48 (s, 1H), 7.93 – 7.79 (m, 1H), 7.63 – 7.49 (m, 1H), 7.36 – 7.21 (m, 2H, overlapped signals), 2.57 (br s, 4H), 1.03 (s, 6H). NMR data are consistent with those previously reported in the literature.[10]

3-((1*H*-Indol-3-yl)methylene)chromane-2,4-dione (6d)

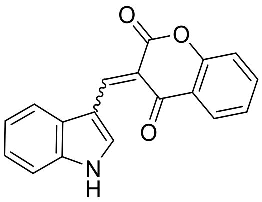

Following the *General Procedure A*, compound **6d** was obtained as an orange powder (70 mg, 69%) from **1b** (102 mg, 0.7 mmol, 2 eq.), **2c** (57 mg, 0.35 mmol, 1 eq.), *L*-proline (4 mg, 0.035 mmol, 0.1 eq.), and TFA (5 μL, 0.07 mmol, 0.2 eq.) in EtOH (1.5 mL, 0.25 M). The product precipitated from the reaction mixture and was isolated by filtration under vacuum. Compound **5d** was obtained as a 50:50 mixture of diastereomers. **^{1}H NMR** (400 MHz, DMSO-d_6) δ 13.16 (s, 2H), 10.00 (s, 1H), 9.58 (s, 1H), 9.14 (s, 1H), 8.97 (s, 1H), 8.09 – 8.01 (m, 2H), 8.02 – 7.95 (m, 2H), 7.78 – 7.68 (m, 2H), 7.67 – 7.63 (m, 2H), 7.44 – 7.32 (m, 8H). NMR data are consistent with those previously reported in the literature.[11]

5-((1*H*-Indol-3-yl)methylene-1,3-dimethylpyrimidine-2,4,6(1*H*,3*H*,5*H*)-trione (6e)

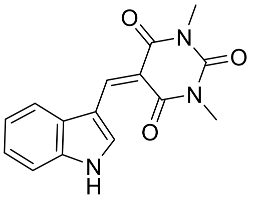

Following the *General Procedure A*, compound **6e** was obtained as a yellow solid (98 mg, 90%) from **1b** (112 mg, 0.8 mmol, 2 eq.), **2e** (60 mg, 0.4 mmol, 1 eq.), *L*-proline (4.5 mg, 0.04 mmol, 0.1 eq.), and TFA (6 µL, 0.08 mmol, 0.2 eq.) in EtOH (1.6 mL, 0.25 M). The product precipitated from the reaction mixture and was isolated by filtration under vacuum. **^{1}H NMR** (400 MHz, DMSO-d_6) δ 12.81 (s, 1H), 9.54 (s, 1H), 8.81 (s, 1H), 7.93 – 7.82 (m, 1H), 7.67 – 7.54 (m, 1H), 7.38 – 7.30 (m, 2H), 3.27 (s, 3H), 3.25 (s, 3H). NMR data are consistent with those expected and previously reported in the literature for similar structures.[12]

2-((1*H*-Indol-3-yl)methylene)-1*H*-indene-1,3(2*H*)-dione (6f)

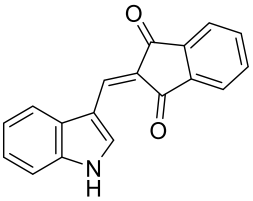

Following the *General Procedure A*, compound **6f** was obtained as a yellow powder (81 mg, 84%) from **1b** (102 mg, 0.7 mmol, 2 eq.), **2f** (51 mg, 0.35 mmol, 1 eq.), *L*-proline (4 mg, 0.035 mmol, 0.1 eq.), and TFA (5 µL, 0.07 mmol, 0.2 eq.) in EtOH (1.5 mL, 0.25 M). The product precipitated from the reaction mixture and was isolated by filtration under vacuum. **^{1}H NMR** (500 MHz, DMSO-d_6) δ 12.75 (s, 1H), 9.64 (s, 1H), 8.18 (s, 1H), 8.05 – 8.01 (m, 1H), 7.93 – 7.85 (m, 4H), 7.63 – 7.58 (m, 1H), 7.36 – 7.30 (m, 2H). NMR data are consistent with those previously reported in the literature.[13]

3,3'-((5-methoxy-1-(phenylsulfonyl)-1*H*-indol-2-yl)methylene)bis(2-methyl-1H-indole) (7a)

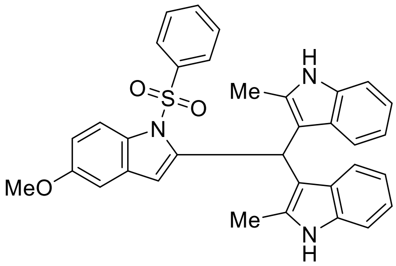

Following the *General Procedure A*, compound **7a** was obtained as an off-white solid (65 mg, 36%) from **1e** (202 mg, 0.64 mmol, 2 eq.), **3f** (42 mg, 0.32 mmol, 1 eq.), *L*-proline (4 mg, 0.032 mmol, 0.1 eq.), and TFA (5 µL, 0.064 mmol, 0.2 eq.) in EtOH (1.3 mL, 0.25 M) after silica gel flash chromatography purification (DCM / hexane gradient from 0:100 to 60:40 v/v). **^{1}H NMR** (400 MHz, DMSO-d_6) δ 10.73 (s, 2H), 7.97 (d, J = 9.1 Hz, 1H), 7.54 (t, J = 7.5 Hz, 1H), 7.43 (d, J = 7.5 Hz, 2H), 7.31 (t, J = 7.7 Hz, 2H), 7.19 (d, J = 8.0 Hz, 2H), 7.03 (d, J = 2.6 Hz, 1H), 6.94 – 6.81 (m, 5H), 6.66 (t, J = 7.5 Hz, 2H), 6.43 (s, 1H), 6.26 (s, 1H), 3.71 (s, 3H), 2.02 (s, 6H). **HRMS:** m/z calcd for $C_{34}H_{29}N_3NaO_3S^+$ [M+Na]$^+$: 582.1822; found 582.1820. The **^{13}C NMR** spectrum could not be recorded due to the low solubility of the compound in the tested solvents.

3,3'-((3,4,5-Trimethoxyphenyl)methylene)bis(2-methyl-1*H*-indole) (7b)

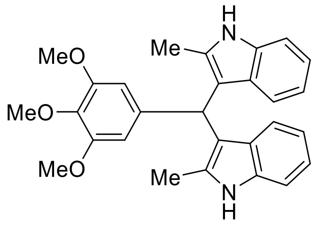

Note that the title compound was not purified, instead it was described using the ^{1}H NMR of the reaction crude.

Following the *General Procedure A*, compound **7a** was obtained as an off-white solid (100%, by crude ^{1}H NMR) from **1g** (392 mg, 2 mmol, 2 eq.), **3f** (131 mg, 1 mmol, 1 eq.), *L*-proline (12 mg, 0.1 mmol, 0.1 eq.), and TFA (15 µL, 0.2 mmol, 0.2 eq.) in EtOH (4 mL, 0.25 M). The volatiles were evaporated under reduced pressure and the crude ^{1}H NMR was recorded. **^{1}H NMR** (400 MHz, DMSO-*d*$_6$) δ 10.70 (s, 2H), 7.26 (s, 1H), 7.19 (d, *J* = 8.6 Hz, 2H), 6.92 – 6.85 (m, 4H), 6.70 (ddd, *J* = 7.9, 7.0, 1.1 Hz, 2H), 6.55 (s, 2H), 5.86 (s, 1H), 3.86 (s, 3H), 3.54 (s, 6H), 2.07 (s, 6H). **HRMS:** m/z calcd for $C_{28}H_{29}N_2O_3^+$ [M+H]$^+$: 441.2173; found 441.2174.

4.3.3. 6-Ethoxy-ICZ (8)

6-Ethoxy-5,11-dihydroindolo[3,2-*b*]carbazole (8)

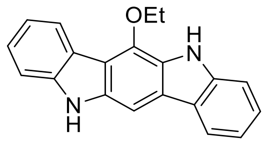

The title compound **8** was obtained as a side product in the synthesis of some adducts **5**. For the characterization and the biological tests, it was isolated from the reaction mixture of the synthesis of compound **5b**. **^{1}H NMR** (400 MHz, DMSO-*d*$_6$) δ 11.07 (s, 1H), 10.97 (s, 1H), 8.23 (dt, *J* = 7.8, 1.3 Hz, 1H), 8.17 (dt, *J* = 7.8, 1.3 Hz, 1H), 7.87 (s, 1H), 7.49 (dt, *J* = 8.1, 0.9 Hz, 1H), 7.45 (dt, *J* = 8.1, 1.0 Hz, 1H), 7.42 – 7.34 (m, 2H, overlapped signals), 7.18 – 7.09 (m, 2H, overlapped signals), 4.39 (q, *J* = 7.0 Hz, 2H), 1.56 (t, *J* = 7.0 Hz, 3H). **^{13}C NMR** (101 MHz, DMSO-*d*$_6$) δ 141.61, 141.21, 138.01, 136.72, 127.73, 126.11, 125.55, 125.10, 123.34, 122.42, 121.74, 120.82, 118.40, 118.28, 115.50, 111.35, 110.66, 96.40, 68.46, 16.31. **HRMS:** m/z calcd for $C_{20}H_{17}N_2O^+$ [M+H]$^+$: 301.1335; found 301.1337.

4.3.4. Post-transformation Products (Series 9-11)

***N*-(4-(3-(5,11-Dihydroindolo[3,2-*b*]carbazol-6-yl)-1*H*-indol-5-yl)phenyl)methanesulfonamide (9a)**

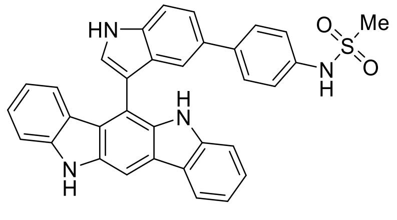

A Schlenk flask was charged with **5b** (50 mg, 0.11 mmol, 1 eq.), 4-methanesulfonamidephenylboronic acid (26 mg, 0.12 mmol, 1.1 eq.), K_2CO_3 (46 mg, 0.33 mmol, 3 eq.), 1,4-dioxane (1 mL), and water (1 mL, final concentration 0.5 M). The reaction mixture was degassed and purged with vacuum-argon cycles (3x). Then was added $Pd(PPh_3)_4$ (6.5 mg, 0.006 mmol, 0.05 eq.) and the reaction was heated to 80 ºC and stirred for 17 h. After reaction completion (TLC control), the reaction was filtered through a pad of celite. The filtrate was partitioned between EtOAc and water. The organic layer was washed with sat. brine, dried over sodium sulfate, filtered, and concentrated under reduced pressure. The crude product was purified *via* silica gel flash chromatography (DCM / hexane gradient from 50:50 to 100:0 v/v then EtOH / DCM gradient from 0:100 to 10:90 v/v) to afford the pure title compound **9a** (50 mg, 83 %) as an off-white solid. **^{1}H NMR** (400 MHz, DMSO-*d*$_6$) δ 11.65 (d, *J* = 2.5 Hz, 1H), 11.13 (s, 1H), 10.39 (s, 1H), 9.61 (s, 1H), 8.23 (d, *J* = 7.7 Hz, 1H), 8.15 (s, 1H), 7.76 (d, *J* = 2.5 Hz, 1H), 7.69 (dd, *J* = 8.5, 0.7 Hz, 1H), 7.48 (dd, *J* = 8.5, 1.8 Hz, 1H), 7.43 (dt, *J* = 3.6, 0.9 Hz, 1H), 7.41 (dt, *J* = 3.6, 0.9 Hz, 1H), 7.38 – 7.28 (m, 3H, overlapped signals), 7.22 (ddd, *J* = 8.2, 7.1, 1.2 Hz, 1H), 7.18 (d, *J* = 1.7 Hz, 1H), 7.15 – 7.05 (m, 4H, overlapped signals), 6.69 (ddd, *J* = 8.1, 7.1, 1.1 Hz, 1H), 2.89 (s, 3H). **^{13}C NMR** (101 MHz, DMSO-*d*$_6$) δ 141.74, 141.70, 137.97, 137.08, 136.56, 135.97, 135.53, 131.49, 127.85, 127.82, 126.26, 125.73, 125.31, 123.21, 123.15, 122.69, 122.30,

121.94, 121.21, 120.66, 118.04, 117.68, 117.44, 111.46, 111.09, 111.01, 110.59, 99.68. The missing ^{13}C signal (-SO_2CH_3), likely to overlap with residual solvent according to predictions. **HRMS:** m/z calcd for $C_{33}H_{25}N_4O_2S^+$ $[M+H]^+$: 541.1693; found 541.1712.

6-(5-(*p*-Tolylethynyl)-1*H*-indol-3-yl)-5,11-dihydroindolo[3,2-*b*]carbazole (9b)

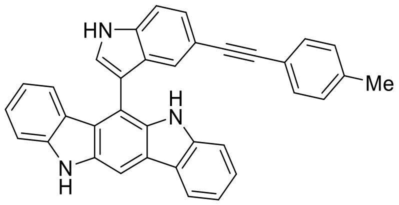

A Schlenk flask was charged with **5b** (30 mg, 0.07 mmol, 1 eq.), 4-ethynyltoluene (25 µL, 0.20 mmol, 3 eq.), Cs_2CO_3 (54 mg, 0.17 mmol, 2.5 eq.), Xantphos (8 mg, 0.13 mmol, 0.2 eq.), CuI (2 mg, 0.01 mmol, 0.15 eq.), CH_3CN (0.6 mL), and water (0.6 mL, final concentration 0.05 M). The reaction mixture was degassed and purged with vacuum-argon cycles (3x). Then was added Bis(acetonitrile)dichloropalladium(II) (1 mg, 0.003 mmol, 0.05 eq.) and the reaction was heated to 100 ºC and stirred for 17 h. After reaction completion (TLC control), the reaction was filtered through a pad of celite. The filtrate was partitioned between EtOAc and water. The organic layer was washed with sat. brine, dried over sodium sulfate, filtered, and concentrated under reduced pressure. The crude product was purified *via* silica gel flash chromatography (EtOAc / hexane gradient from 20:80 to 100:0) to afford the pure title compound **9b** (17 mg, 53 %) as pale brown solid. **^{1}H NMR** (400 MHz, DMSO-d_6) δ 11.81 (d, *J* = 2.5 Hz, 1H), 11.15 (s, 1H), 10.41 (s, 1H), 8.24 (d, *J* = 7.8 Hz, 1H), 8.17 (s, 1H), 7.80 (d, *J* = 2.4 Hz, 1H), 7.66 (dd, *J* = 8.5, 0.8 Hz, 1H), 7.44 (dt, *J* = 8.1, 0.9 Hz, 2H), 7.41 (dt, *J* = 8.1, 1.0 Hz, 1H), 7.37 – 7.30 (m, 2H, overlapped signals), 7.28 – 7.21 (m, 3H, overlapped signals), 7.14 (d, *J* = 1.0 Hz, 1H), 7.13 – 7.10 (m, 1H), 7.09 (d, *J* = 7.9 Hz, 1H), 6.97 (d, *J* = 7.4 Hz, 1H), 6.72 (ddd, *J* = 8.0, 7.1, 1.0 Hz, 1H), 2.25 (s, 3H). **^{13}C NMR** (101 MHz, DMSO-d_6) δ 141.75, 141.69, 138.10, 136.81, 135.88, 135.61, 131.37, 129.61, 127.46, 126.68, 125.78, 125.39, 125.19, 123.27, 123.19, 123.07, 122.70, 122.03, 121.98, 120.71, 120.37, 118.07, 117.60, 113.22, 112.95, 111.41, 111.00, 110.68, 110.28, 99.94, 91.10, 87.34, 21.40. **HRMS** m/z calcd for $C_{35}H_{24}N_3^+$ $[M+H]^+$: 486.1965; found 486.1965.

***N*-(2-(5,11-Dihydroindolo[3,2-*b*]carbazole-6-carbonyl)phenyl)acetamide (10)**

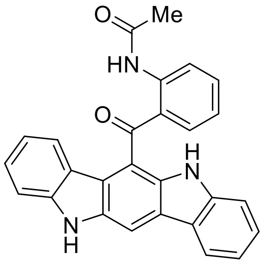

To a solution of **5d** (500 mg, 1.3 mmol, 1 eq.) in dry DCM (8 mL, final 0.1 M) under inert atmosphere was added dropwise a suspension of MCPBA (70% w/w, 569 mg, 2.6 mmol, 2 eq.) in DCM (5 mL). The vessel was sealed, and the reaction was stirred at rt for 5 h, until full consumption of starting material (TLC control). The reaction was diluted with DCM and washed with saturated $NaHCO_3$, saturated brine and water. The organic layer was dried over sodium sulfate, filtered, and concentrated under reduced pressure. The crude residue was purified *via* silica gel flash chromatography (DCM / hexane gradient from 0:100 to 50:0 v/v) to afford the pure title compound **10** (450 mg, 83%) as a bright orange solid. **^{1}H NMR** (400 MHz, DMSO-d_6) δ 11.57 (s, 1H), 11.43 (s, 1H), 10.92 (s, 1H), 8.55 (dd, *J* = 8.4, 1.1 Hz, 1H), 8.39 (d, *J* = 0.5 Hz, 1H), 8.28 (d, *J* = 7.9 Hz, 1H), 7.67 – 7.58 (m, 1H), 7.50 (dt, *J* = 8.1, 0.9 Hz, 1H), 7.46 – 7.30 (m, 5H, overlapped signals), 7.21 – 7.15 (m, 1H), 6.95 – 6.91 (m, 1H), 6.91 – 6.86 (m, 1H), 2.28 (s, 3H). **^{13}C NMR** (101 MHz, DMSO-d_6) δ 199.80, 169.70, 141.91, 141.82, 141.01, 135.65, 135.23, 134.07, 133.35, 126.62, 126.24, 124.86, 123.68, 122.47, 122.34, 121.41, 121.20, 120.95, 119.39, 118.88, 118.35, 114.63, 111.45, 111.39, 104.31, 25.47. **HRMS:** m/z calcd for $C_{27}H_{20}N_3O_2^+$ $[M+H]^+$: 418.1550; found 418.1548.

1-Acetyl-5'*H*-spiro[indoline-2,6'-indolo[3,2-*b*]carbazole]-3,12'(11'*H*)-dione (11)

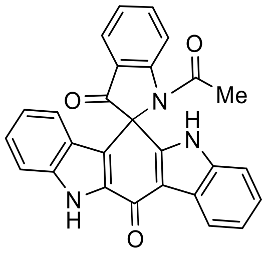

To a solution of **10** (260 mg, 0.6 mmol, 1 eq.) in dry DCM (4 mL, final 0.1 M) under inert atmosphere was added dropwise a suspension of MCPBA (70% w/w, 287 mg, 1.2 mmol, 2 eq.) in DCM (2 mL). The vessel was sealed, and the reaction was stirred at reflux temperature for 5 h, until full consumption of starting material (TLC control). The reaction was diluted with DCM and washed with saturated $NaHCO_3$, saturated brine and water. The organic layer was dried over sodium sulfate, filtered, and concentrated under reduced pressure. The crude residue was purified *via* silica gel flash chromatography (DCM / hexane gradient from 30:70 to 70:30 v/v) to afford the title compound **11** (200 mg, 74%) as a light brown solid. Samples for X-Ray crystallography were obtained by slow evaporation of a THF solution of the pure compound **10**. **^{1}H NMR** (400 MHz, MeOD) δ 8.88 (d, *J* = 7.2 Hz, 1H), 8.27 – 8.20 (m, 1H), 7.93 (ddd, *J* = 8.6, 7.3, 1.5 Hz, 1H), 7.77 (ddd, *J* = 7.7, 1.5, 0.7 Hz, 1H), 7.47 (dt, *J* = 8.4, 0.9 Hz, 1H), 7.38 (td, *J* = 7.5, 0.8 Hz, 1H), 7.35 – 7.30 (m, 1H), 7.24 – 7.20 (m, 2H, overlapped signals), 7.17 (ddd, *J* = 8.3, 7.0, 1.1 Hz, 1H), 6.83 (ddd, *J* = 8.0, 7.0, 1.0 Hz, 1H), 6.72 (dt, *J* = 8.2, 1.0 Hz, 1H), 1.53 (s, 3H). **^{13}C NMR** (101 MHz, MeOD) δ 191.81, 175.75, 170.08, 154.53, 144.92, 138.64, 137.99, 137.38, 134.14, 125.36, 125.26, 124.35, 124.01, 122.36, 122.29, 121.67, 120.98, 120.97, 119.16, 118.54, 116.51, 114.74, 113.09, 111.83, 69.16, 21.83. **HRMS:** m/z calcd for $C_{27}H_{18}N_3O_3^+$ [M+H]$^+$: 432.1343; found 432.1344.

4.3.5. Conjuated 6-ICZs and bifunctional AhR probes (Series 12)

3-(5,11-Dihydroindolo[3,2-*b*]carbazole-6-yl)-*N*-pentyl-1*H*-indole-6-carboxamide (12a)

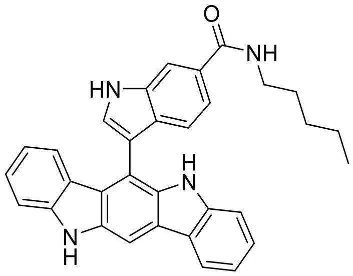

Following the *General Procedure C*, compound **12a** was obtained as a pale brown solid (16 mg, 69%), from **4j** (20 mg, 0.05 mmol, 1 eq.), amylamine (6 μL, 0.053 mmol, 1.1 eq.), HATU (22 mg, 0.06 mmol, 1.2 eq.), and DIPEA (6.2 mg, 0.05 mmol, 1.0 eq.) in DMF (2 mL) after stirring for 24 h. The pure compound **12a** was obtained after flash chromatography purification (EtOAc / hexane gradient from 0:100 to 80:20 v/v). **^{1}H NMR** (400 MHz, DMSO-*d$_6$*) δ 11.85 (d, *J* = 2.4 Hz, 1H), 11.12 (s, 1H), 10.39 (s, 1H), 8.39 (t, *J* = 5.7 Hz, 1H), 8.25 – 8.20 (m, 1H), 8.16 (dd, *J* = 1.6, 0.7 Hz, 1H), 8.14 (d, *J* = 0.5 Hz, 1H), 7.88 (d, *J* = 2.5 Hz, 1H), 7.45 – 7.42 (m, 1H), 7.40 (dt, *J* = 9.1, 0.9 Hz, 1H), 7.32 (ddd, *J* = 8.1, 7.0, 1.2 Hz, 1H), 7.22 (ddd, *J* = 8.2, 7.1, 1.2 Hz, 1H), 7.12 (ddd, *J* = 7.9, 7.0, 1.1 Hz, 1H), 7.01 (d, *J* = 8.4 Hz, 1H), 6.97 – 6.93 (m, 1H), 6.69 (ddd, *J* = 8.1, 7.1, 1.1 Hz, 1H), 3.29 – 3.25 (m, 2H), 1-58 – 1.51 (m, 2H), 1.36 – 1.26 (m, 4H), 0.93 – 0.83 (m, 3H). **^{13}C NMR** (101 MHz, DMSO-*d$_6$*) δ 166.99, 141.28, 141.20, 135.99, 135.46, 135.00, 128.81, 128.11, 127.45, 125.27, 124.82, 122.72, 122.61, 122.21, 121.70, 121.42, 120.19, 118.86, 118.02, 117.58, 117.02, 111.73, 110.93, 110.42, 110.20, 110.11, 99.29, 29.00, 28.79, 21.94, 13.98, 0.11. **HRMS:** m/z calcd for $C_{32}H_{28}N_4O^{\cdot+}$ [M$^{\cdot}$]$^+$: 484.2258; found 484.2268.

5m-PEG3-thalidomide (12b)

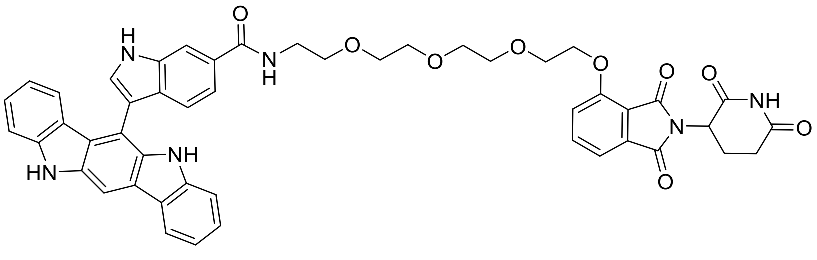

Following the *General Procedure C*, compound **12b** was obtained as a cream solid (0.040 g, 78%) from 0.057 mmol of **4j** (1.1 Eq., 0.024 g), 0.052 mmol of the commercial **Thalidomide 4'-ether-PEG3-amine** (1.0 Eq., 0.025 g), HATU (1.2 Eq., 0.062 mmol, 0.025 g) and DIPEA (4.5 Eq., 0.232 mmol, 40 μL) in 2.1 mL of DMF after stirring at room temperature for 14 hours under inert atmosphere. The pure product **12b** was obtained from a reverse phase purification by automated column chromatography, using H_2O+TFA (0.05%) / ACN+TFA (0.05%) (gradient from 2:98 to 100:00, v/v) as solvent system. **^{1}H NMR** (400 MHz, DMSO-*d$_6$*) δ 11.88 (d, *J* = 2.5 Hz, 1H), 11.13 (s, 1H), 11.09 (br s, 1H), 10.40 (s, 1H), 8.44 (t, *J* = 5.6 Hz, 1H), 8.22 (d, *J* = 7.8 Hz, 1H), 8.17 (d, *J* = 1.5 Hz, 1H), 8.15 (s, 1H), 7.89 (d, *J* = 2.5 Hz, 1H), 7.76 (dd, *J* = 8.5, 7.3 Hz, 1H), 7.50 – 7.38 (m, 5H, overlapped signals), 7.32 (ddd, *J* = 8.1, 7.0, 1.2 Hz, 1H), 7.22 (ddd, *J* = 8.2, 7.0, 1.2 Hz, 1H), 7.11 (ddd, *J* = 8.0, 7.0, 1.1 Hz, 1H), 7.01 (d, *J* = 8.4 Hz, 1H), 6.94 (d, *J* = 8.0 Hz, 1H), 6.68 (ddd, *J* = 8.0, 7.1, 1.1 Hz, 1H), 5.07 (dd, *J* = 12.8, 5.4 Hz, 1H), 4.33 – 4.26 (m, 2H), 3.80 – 3.73 (m, 2H), 3.65 – 3.59 (m, 2H), 3.58 – 3.49 (m, 8H), 3.44 (q, *J* = 6.1 Hz, 2H), 2.93 – 2.81 (m, 1H), 2.61 – 2.53 (m, 2H), 2.06 – 1.96 (m, 1H). **^{13}C NMR** (101 MHz, DMSO-*d$_6$*) δ 173.26, 170.41, 167.66, 167.28, 165.73, 156.27, 141.75, 141.67, 137.42, 136.45, 135.94, 135.46, 133.70, 129.39, 128.21, 128.05, 125.75, 125.30, 123.19, 123.07, 122.69, 122.19, 121.88, 120.67, 120.44, 119.39, 118.49, 118.06, 117.50, 116.77, 115.84, 112.29, 111.40, 110.92, 110.63, 110.59, 99.78, 70.62, 70.31, 70.22, 70.07, 69.52, 69.27, 69.13, 49.21, 40.90, 31.42, 22.46. **HRMS:** m/z calcd for $C_{48}H_{43}N_6O_9^+$ [M+H]$^+$: 847.3086; found 847.3080.

Bis-5m-diaminodecane (12c)

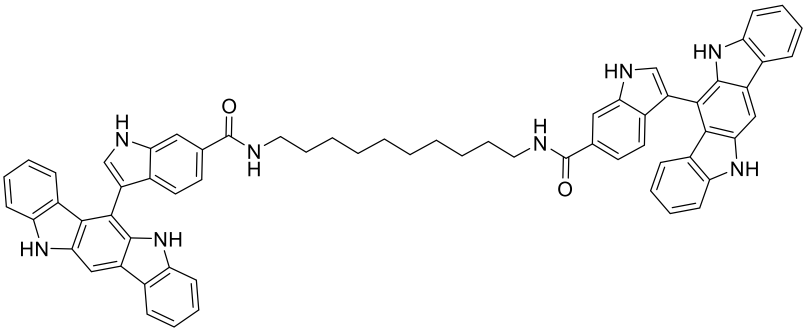

Following the *General Procedure C*, compound **12c** was obtained as a dark red solid (0.016 g, 36%) from 0.108 mmol of **4j** (2.3 Eq., 0.045 g), 0.046 mmol of **1,10-diaminodecane** (1.0 Eq., 8 mg), HATU (3.0 Eq., 0.139 mmol, 0.053 g) and DIPEA (6.5 Eq., 0.302 mmol, 0.053 mL) in 0.500 mL of DMF after stirring for 24 hours at room temperature under inert atmosphere. The pure product **12c** was obtained from a column chromatography, using Hexane / EtOAc (gradient from 100:0 to 0:100, v/v) and then EtOAc / EtOH (95:5, v/v) as solvent system. **^{1}H NMR** (400 MHz, DMSO-*d$_6$*) δ 11.85 (d, *J* = 2.5 Hz, 2H), 11.12 (s, 2H), 10.40 (s, 2H), 8.39 (t, *J* = 5.6 Hz, 2H), 8.22 (d, *J* = 7.8 Hz, 2H), 8.16 (dd, *J* = 1.4, 0.7 Hz, 2H), 8.14 (s, 2H), 7.88 (d, *J* = 2.5 Hz, 2H), 7.46 – 7.37 (m, 6H, overlapped signals), 7.32 (ddd, *J* = 8.2, 7.0, 1.3 Hz, 2H), 7.21 (ddd, *J* = 8.2, 7.1, 1.3 Hz, 2H), 7.11 (ddd, *J* = 7.9, 7.0, 1.1 Hz, 2H), 7.01 (d, *J* = 8.4 Hz, 2H), 6.95 (d, *J* = 7.9 Hz, 2H), 6.68 (ddd, *J* = 8.0, 7.1, 1.0 Hz, 2H), 3.30 – 3.24 (m, 4H, overlapped signals), 1.57 – 1.50 (m, 4H, overlapped signals), 1.34 – 1.28 (m, 12H, overlapped signals). **^{13}C NMR** (101 MHz, DMSO-*d$_6$*) δ 167.47, 141.75, 141.67, 136.46, 135.93, 135.47, 129.28, 128.58, 127.92, 125.74, 125.29, 123.19, 123.08, 122.68, 122.18, 121.89, 120.66, 119.33, 118.49, 118.05, 117.50, 112.21, 111.41, 110.89, 110.67, 110.58, 99.77, 29.80, 29.53, 29.35, 27.08. The ^{13}C signal corresponding to the linker carbons closer to the amide is estimated at around 40 ppm, and thus likely overlapped with the residual solvent signal. **HRMS:** m/z calcd for $C_{64}H_{54}N_8O_2^{\cdot+}$ [M$^\cdot$]$^+$: 966.4364; found 966.4373.

Bis-5m-PEG3 (12d)

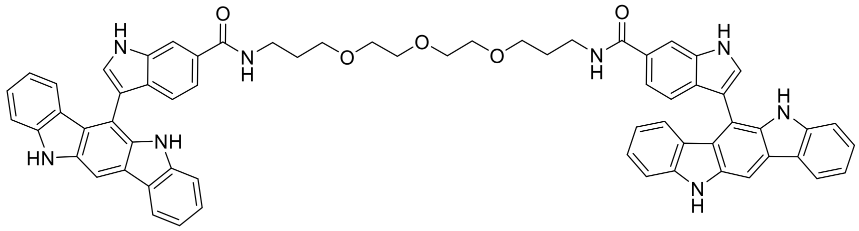

Following the *General Procedure C*, compound **12d** was obtained as a pink solid (0.056 g, 71%) from 0.193 mmol of **4j** (2.5 Eq., 0.080 g), 0.077 mmol of **PEG-3-diamine** (1.0 Eq., 0.017 g), HATU (3.0 Eq., 0.231 mmol, 0.090 g) and DIPEA (6.3 Eq., 0.481 mmol, 0.084 mL) in 1.6 mL of DMF after stirring for 24 hours at room temperature under inert atmosphere. The pure product **12d** was obtained from a column chromatography, using Hexane / EtOAc (gradient from 100:0 to 0:100, v/v) and then EtOAc / EtOH (95:5, v/v) as solvent system. **^{1}H NMR** (400 MHz, DMSO-d_6) δ 11.86 (d, J = 2.5 Hz, 2H), 11.12 (d, J = 3.2 Hz, 2H), 10.39 (s, 2H), 8.44 (s, 1H), 8.39 (t, J = 5.6 Hz, 2H), 8.22 (d, J = 8.1 Hz, 4H), 8.16 (s, 4H), 8.14 (s, 4H), 7.88 (d, J = 2.5 Hz, 2H), 7.45 – 7.38 (m, 5H), 7.32 (t, J = 9.1 Hz, 3H), 7.21 (t, J = 8.2 Hz, 3H), 7.11 (t, J = 7.4 Hz, 3H), 7.01 (d, J = 8.4 Hz, 2H), 6.95 (d, J = 7.5 Hz, 2H), 6.68 (t, J = 8.1 Hz, 2H), 3.56 – 3.45 (m, 16H), 1.78 (t, J = 6.7 Hz, 4H). **^{13}C NMR** (101 MHz, DMSO-d_6) δ 167.10, 141.27, 141.20, 135.98, 135.46, 134.99, 128.85, 127.99, 127.49, 125.26, 124.81, 122.71, 122.61, 122.21, 121.70, 121.41, 120.18, 118.88, 117.99, 117.57, 117.02, 111.74, 110.93, 110.43, 110.18, 110.10, 99.29, 69.79, 69.58, 68.42, 54.91, 36.74, 29.55. **HRMS:** m/z calcd for $C_{64}H_{55}N_8O_5^+$ [M+H]$^+$: 1015.4290; found 1015.4256.

5. Axial Chirality of 6-ICZs

5.1. Experimental Evidence

Figure S19. NMR spectroscopy evidence Structures and diastereotopic groups in compounds **4a**, **4b**, and **5g**. A) ^{1}H NMR spectra of **4a**, where the highlighted methyl groups appear as a diastereotopic set of signals. B) ^{1}H NMR spectra of **4b**, where the highlighted hydrogens of the methylene group appear as a diastereotopic set of signals. C) ^{1}H NMR spectra of **5g**, where the highlighted hydrogens of the methylene group and the hydrogen of the hydroxyl group appear as a diastereotopic set of signals.

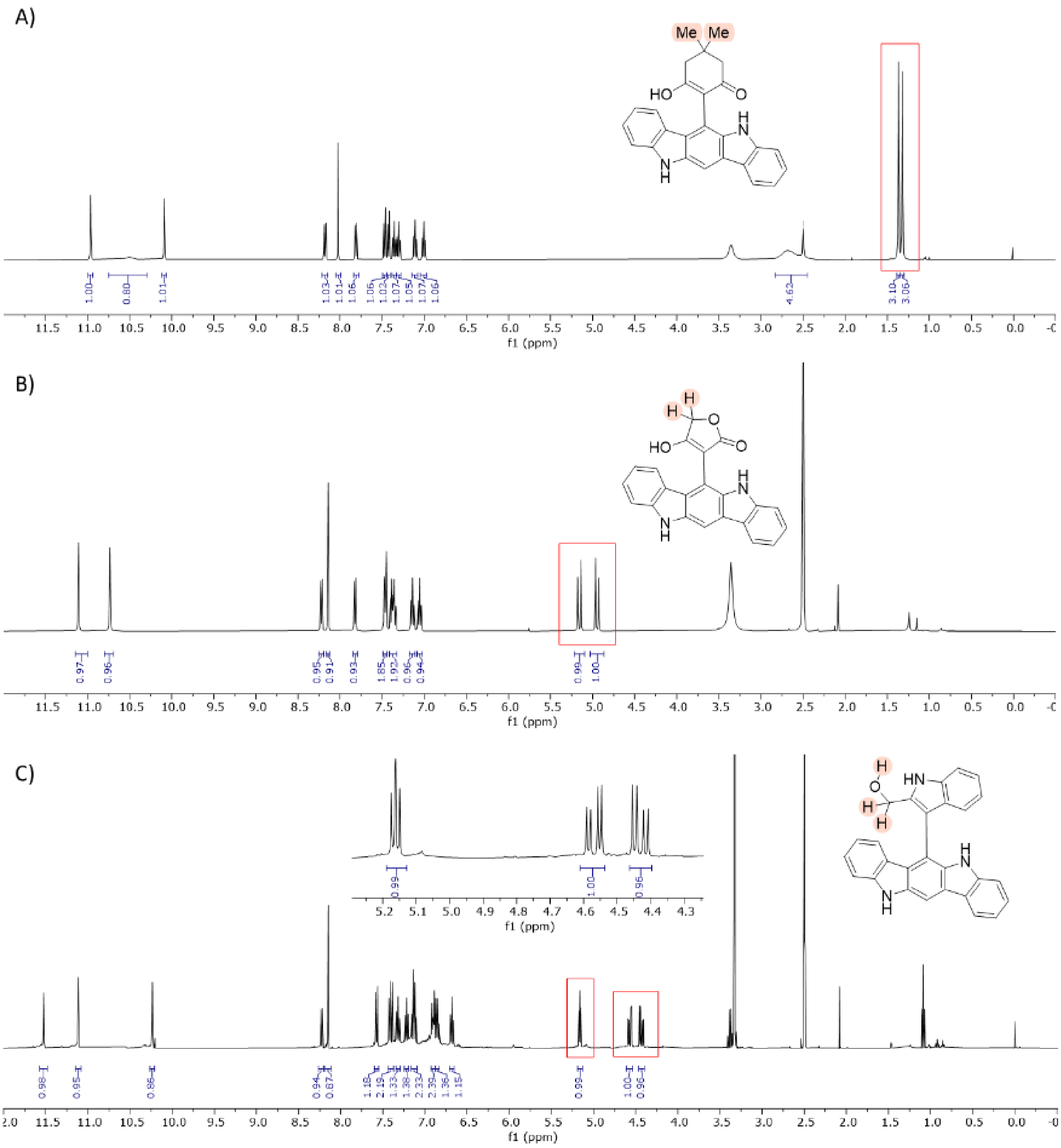

Figure S20. Chiral HPLC Analysis of Representative Compounds 5. Chiral HPLC of representative 6-ICZs **5b**, **5e**, and **5m** was run on the chiral column CHIRALPAK AD-H (Daicel Chemical Ind.) of 4.6 mm x 250 mm (particle size 5 µm), running time = 50 min, eluent either CH_3CN + 0.07% TFA / H_2O + 0.1% TFA (acidic media, method I) or CH_3CN / H_2O (neutral media, method II). A) The chiral HPLC profile of the compound **5b** (bearing a substituent at **C-5** and no substituent at **C-2**), was studied using two different methods: acidic and neutral media. In both cases a single peak was detected, at 19.128 min (acidic media) and 19.046 min (neutral media), indicating the existence of a single compound. B) The chiral HPLC profile of the compound **5m** (bearing a substituent at **C-6** and no substituent at **C-2**), was studied using the two different methods: acidic and neutral media. In both cases a single peak was detected, at 11.536 min (acidic media) and 11.971 min (neutral media), indicating the existence of a single compound. C) The chiral HPLC profile of compound **5e** (bearing a substituent at **C-2**) in acidic media showed two peaks, with retention times of 19.094 min and 20.711 min, respectively, indicating the existence of two enantiomers.

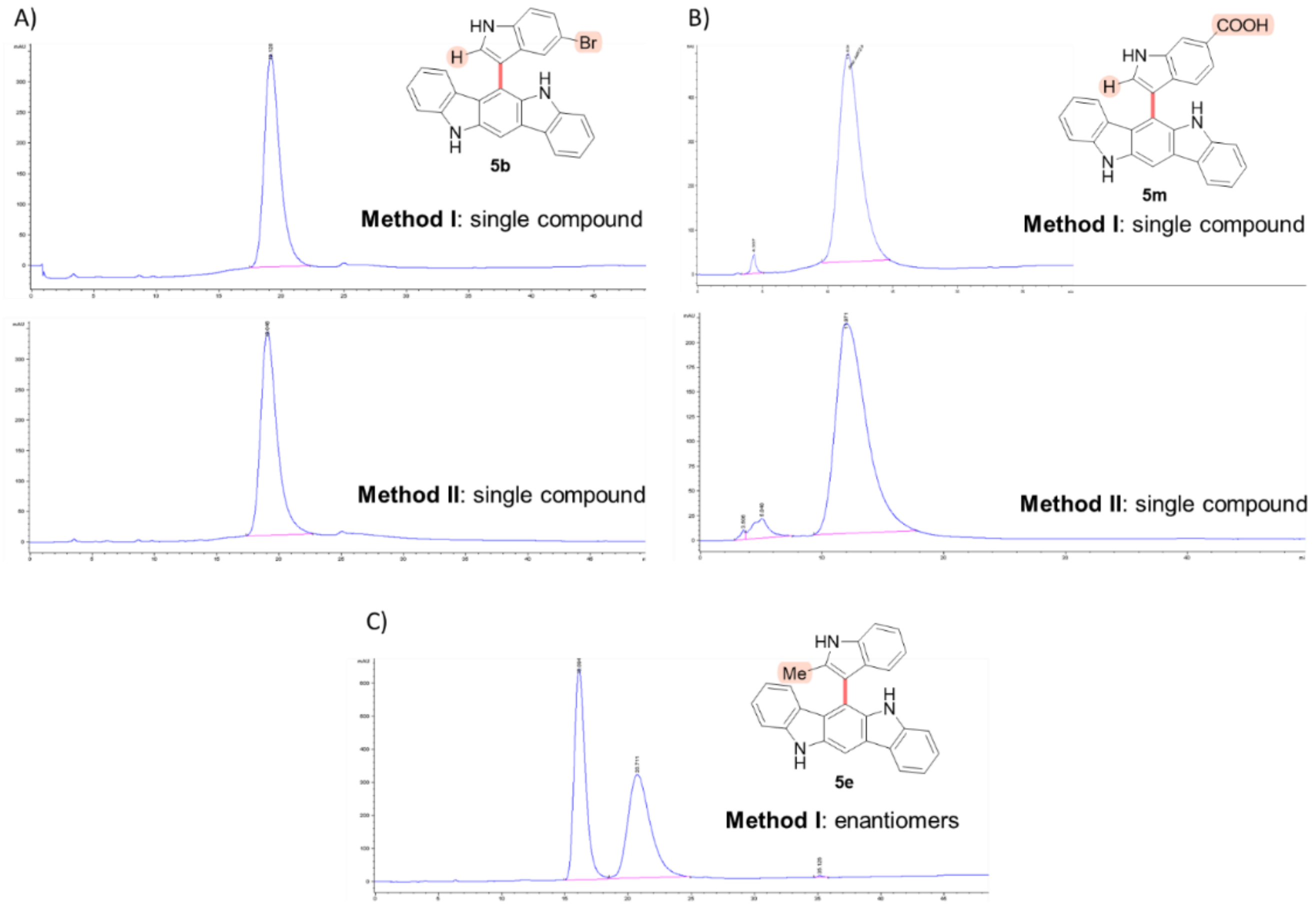

5.2. Computational Studies

Density functional theory calculations

Full geometry optimizations at the M062X/6-31G(d,p)[14-16] level were performed to locate the stationary points corresponding to energy minima and transitions states in conjunction with the Gaussian16 program.[17] The nature of the stationary points was verified upon inspection of the harmonic vibrational frequencies (with zero and one imaginary frequency for energy minimum and transition state structures). The relative stabilities in the gas phase were estimated by adding the zero-point energy, thermal and entropy corrections to the relative energies estimated at 1 atm. and 298 K. For the sake of comparison, additional computations were performed at the second order Möller-Plesset perturbation theory (MP2)[18] in conjunction with the 6-31G(d,p) basis set. Finally, the relative stability in acetonitrile and water was estimated by adding the corresponding solvation free energy determined by using the SMD[19] and MST[20] continuum solvation models.

Docking calculations

Docking of compounds **5c** and **5j'** was performed with Glide using the XP score function.[21] The docking grid was defined with a 30/45 Å inner/outer box centered on the original position of the ligand in the selected cryo-EM structure (PDB ID: 7ZUB). The grid center is given by coordinates x = 30.1, y = - 6.3 and z = - 6.2 Å. Conformer generation and ligand pose screening were conducted using Glide's default algorithm. Docking parameters such as the maximum number of poses per ligand and the number of ligand poses considered for post-docking minimization were both adjusted to 100. The energy window for retaining ring conformers was set to 2.5 kcal/mol, and an RMSD threshold of 0.5 Å was applied for pose rejection. The optimal pose was primarily chosen through visual inspection of the interactions with residues in the binding pocket and the docking score. Let us note that this protocol predicted correctly the binding pose of the X-ray ligand (indirubin) bound to 7ZUB.

Molecular dynamics simulations

The refinement of the binding mode for the X-ray ligand and compounds **5c**, **5j'** and **12a** was completed through MD dynamics simulations using Amber20 software and the ff19SB force field.[22,23] Ligands were parametrized using gaff2 forcefield,[24] a process that involved fitting the HF/6-31G(d) electrostatic potential generated around each molecule. The simulation systems were embedded in an OPC water box with approximate size of 98 x 93 x 103 $Å^3$,[25] resulting in systems containing around 8000 water molecules. To maintain system neutrality, six chlorine ions were introduced in the simulation system. For all systems, the protocol started with a three-step minimization, gradually minimizing hydrogens, water molecules and ultimately the entire system. Subsequently, a six-step heating-equilibration process was performed to increase the temperature from 100 to 300 K. SHAKE algorithm was applied for bonds involving hydrogen atoms.[26] Electrostatic interactions were treated with Particle Mesh Ewald (PME) and a cutoff of 10 Å was set for the nonbonded interactions.[27] All trajectories were extended until 500 ns and a time step of 2 ps was used for saving the trajectory.

Figure S21. A) Schematic representation of the constrained dihedral angle φ used to estimate the racemization energy profile. B) Structure of derivatives **5c** (R = H) and **5e** (R = Me).

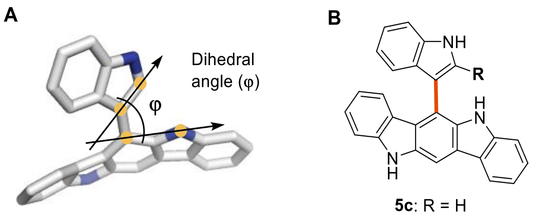

5c: R = H
5e: R = Me

Figure S22. Energy barrier diagram for the rotation around the constrained dihedral bond determined from MP2/6-31G(d,p) calculations and representative views of the flat (**M1**, **M2**) and bent configurations (**TS1**, **TS3**) for the rotation of compound **5c** (R = H). Gray dots denote the location of the energy minima and transition states obtained from full geometry optimizations.

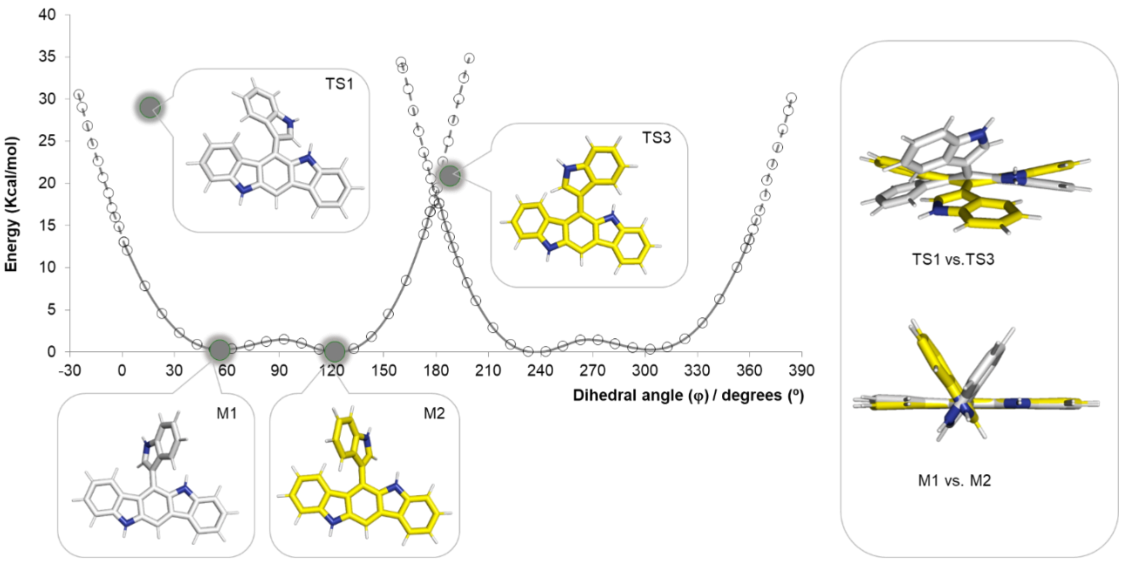

Figure S23. Energy barrier diagram for the rotation around the constrained dihedral bond determined from MP2/6-31G(d,p) calculations and representative views of the flat (**M1**, **M2**) and bent configurations (**TS1**, **TS3**) for the rotation of compound **5e** (R = Me). Orange dots denote the location of the energy minima and transition states obtained from full geometry optimizations.

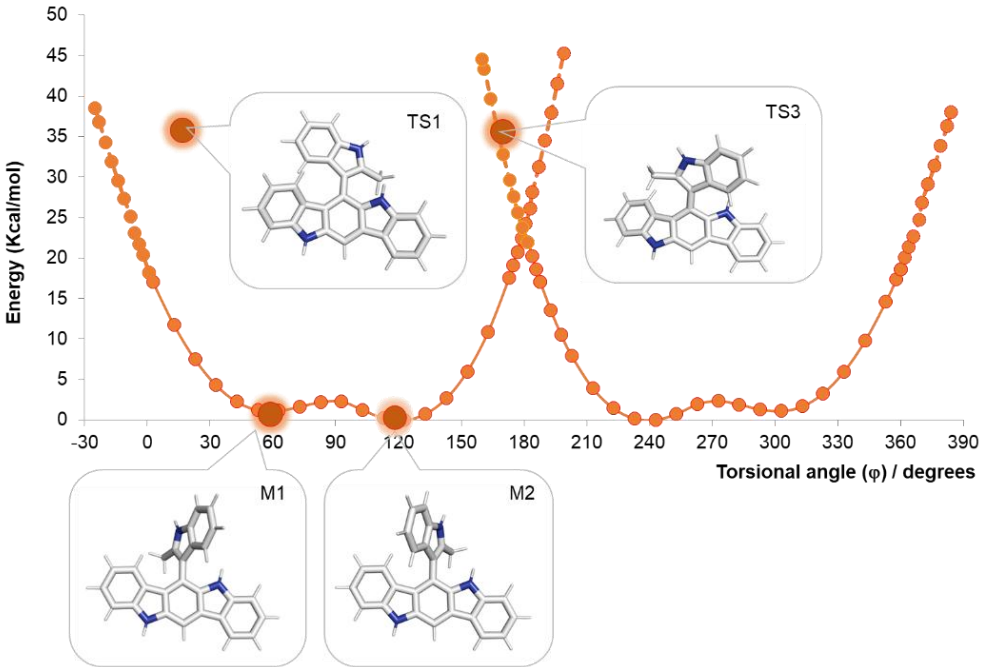

Table S3. Relative stabilities (Kcal/mol) between energy minima (**M1**, **M2**) and transition state (**TS1**, **TS3**) structures determined for the racemization of compounds **5c** (R = H) and **5e** (R = Me) in the gas phase, acetonitrile, and water. The relative stabilities were determined by combining the gas phase free energies with the solvation free energies determined with the SMD and MST continuum solvation models.

	**5c** (R = H)				**5e** (R = Me)			
	Gas	Acetonitrile (SMD)	Water (SMD)	Water (MST)	Gas	Acetonitrile (SMD)	Water (SMD)	Water (MST)
**M1**	0.0	0.0	0.0	0.0	0.0	0.0	0.0	0.0
**M2**	0.2	-0.3	-0.5	-0.6	0.7	-0.2	-0.1	-0.2
**TS1**	29.0	27.8	27.9	27.8	35.7	34.4	34.6	34.6
**TS3**	20.9	20.3	27.8	20.6	35.6	34.8	34.7	35.1

Calculated total energies and geometrical coordinates for the energy minima (**M1**, **M2**) and transition state (**TS1**, **TS3**) structures determined from M062X/6-31G(d,p) calculations.

5c (R = H): energy minimum M1

C,0,-2.3543806305,-2.3318082943,0.3412898608
C,0,-0.9674477646,-2.2075644817,0.5198262609
C,0,-0.3453423133,-0.9672359502,0.5300159893
C,0,-1.148477319,0.1611838977,0.355455976
C,0,-2.5477096003,0.0546775588,0.1759892756
C,0,-3.1475143418,-1.2064594747,0.1692478116
H,0,-2.8064915975,-3.317665611,0.3385888519
H,0,-0.3662962874,-3.101368374,0.6529523687
H,0,0.7270847656,-0.8762359467,0.6679168443
H,0,-4.220610145,-1.3028068197,0.0321152579
C,0,-1.9316252791,2.2686494265,0.1354833964
C,0,-2.0126279771,3.6589598811,0.0231761233
C,0,-3.2996056956,4.1800639779,-0.1817474549
C,0,-4.4194410142,3.3065345385,-0.3020969431
C,0,-4.3250853614,1.9256868364,-0.2008339526
C,0,-3.0538758446,1.4048868581,0.0305518879
H,0,-5.1956471671,1.2846690454,-0.2972261926
N,0,-0.7964427089,1.4928759456,0.3218687367
H,0,0.1319791982,1.8651797607,0.4442773911
C,0,-3.825042609,5.5337188232,-0.2926802445
C,0,-5.2206674613,5.41100263,-0.4992233428
N,0,-5.5539380351,4.074688091,-0.513960565
H,0,-6.4887983139,3.7116646641,-0.5878100458
C,0,-3.2702493189,6.8158218789,-0.2003663094
H,0,-2.2103539941,6.9405868165,-0.0112205023
C,0,-4.093162481,7.9254220216,-0.3340770466
H,0,-3.6677079534,8.920503039,-0.2602157921
C,0,-5.4696624968,7.7782705549,-0.5587404756
C,0,-6.0521741562,6.5224087405,-0.6414095262
H,0,-6.0925325129,8.6608275724,-0.6636976295
H,0,-7.118861627,6.4051943227,-0.803693683
C,0,-0.7865057064,4.4724294362,0.0751018718
C,0,0.231203504,4.4458614422,1.0990749097
C,0,-0.3443137862,5.3496431629,-0.8802529518
C,0,1.253603255,5.335954971,0.6955916787
C,0,0.3586330397,3.7684936077,2.3239341309
C,0,2.3986697928,5.5565655034,1.4671739232
C,0,1.4924880058,3.9806391745,3.087639927
H,0,-0.4272196825,3.0978694086,2.6596339406
H,0,1.4054101418,6.5372168498,-1.0490145042
C,0,2.5033733075,4.8651117075,2.6612222731
H,0,3.1735857932,6.2439962108,1.1431346866
H,0,1.6058839811,3.4650968211,4.0354094086
H,0,3.3794558575,5.0107401221,3.284462068
N,0,0.8714886121,5.8756501256,-0.5108309746
H,0,-0.8169228825,5.6360269573,-1.8091661439
E(RM062X) = -1164.88181568 A.U.

5c (R = H): energy minimum M2

C,0,5.9917217952,-0.2986550033,0.3650498518
C,0,5.8911156284,1.0672893653,0.0581522997
C,0,4.6680585757,1.6586577301,-0.2256586552
C,0,3.5330430865,0.8470953381,-0.1929325959
C,0,3.6153441025,-0.5300137092,0.1207942555
C,0,4.8595591632,-1.1002639907,0.3975253111
H,0,6.9644462925,-0.7276753872,0.5796556722
H,0,6.7892943725,1.6763242754,0.0413263191
H,0,4.5960565793,2.7149434173,-0.4637221203
H,0,4.9375998817,-2.1566449673,0.6373762105
C,0,1.4238009295,0.0408583245,-0.2676731248
C,0,0.0358720218,-0.0553405602,-0.3827010113
C,0,-0.5076927144,-1.3268914912,-0.1424860556
C,0,0.3384904087,-2.4180691334,0.2122547986
C,0,1.7170792779,-2.3077784068,0.3276447022
C,0,2.262802073,-1.0499602943,0.0794870924
H,0,2.3377425896,-3.1556629323,0.5996184591
N,0,2.2155527639,1.167134497,-0.4441226637
H,0,1.8422392594,2.0952215819,-0.5611871143
C,0,-1.8591845557,-1.8610596234,-0.2054226695
C,0,-1.7692609043,-3.2325846022,0.1342059313
N,0,-0.4509773689,-3.5435440013,0.3886792002
H,0,-0.1046863921,-4.4602526395,0.6142330538
C,0,-3.1095864034,-1.3275232033,-0.5400230942
H,0,-3.2052363953,-0.2845627378,-0.8189671414
C,0,-4.2263507487,-2.1512893332,-0.5123483874
H,0,-5.1982856125,-1.7439834715,-0.7688047279
C,0,-4.1158134006,-3.503567203,-0.1573742117
C,0,-2.8891714635,-4.0637652783,0.1681565323
H,0,-5.0045031837,-4.126460586,-0.140506908
H,0,-2.8005324903,-5.1120099293,0.4349568859
C,0,-0.7681641589,1.1374934119,-0.7007224595
C,0,-1.8168665926,1.7202926539,0.1013583182
C,0,-0.6260271942,1.9517699527,-1.795892318
C,0,-2.2697463577,2.873645001,-0.5767253342
C,0,-2.4067712566,1.3658487753,1.3247972843
C,0,-3.2947609661,3.6813163388,-0.0721260623
C,0,-3.4215041527,2.1596582297,1.8263533704
H,0,-2.0725699789,0.4762686924,1.8503921918
H,0,-1.6429347715,3.7042027508,-2.4285452464
C,0,-3.8604038646,3.3064745533,1.1332079692
H,0,-3.6317973278,4.5650974311,-0.6044750517
H,0,-3.8923869355,1.8987675421,2.7681214723
H,0,-4.659825506,3.9073922324,1.5541901211
N,0,-1.5231709028,2.9929347917,-1.7272063253
H,0,0.041310558,1.846990168,-2.6400148845
E(RM062X) = -1164.88130956 A.U.

5c (R = H): transition state TS1

C,0,6.0789917636,0.3096774779,-0.1204752467
C,0,5.9573813269,-0.9832880946,-0.6539063274
C,0,4.7170864253,-1.578119621,-0.8376940851
C,0,3.5881799932,-0.8433529779,-0.4713385379
C,0,3.6914250269,0.4624612519,0.0537330525
C,0,4.9515719722,1.0373480767,0.2323525109
H,0,7.0648226747,0.7420885117,0.0114785655
H,0,6.8528026252,-1.5318047508,-0.9281605308
H,0,4.62830519,-2.5784894449,-1.2489298329
H,0,5.0456374186,2.0413934653,0.6354854391
C,0,1.4634303997,-0.1170426374,-0.1157115907
C,0,0.0523935198,-0.0552943127,-0.0328058949
C,0,-0.4249333852,1.2713220161,0.151618713
C,0,0.45753317,2.2704457621,0.67408395
C,0,1.8292146664,2.1231714669,0.7732956836
C,0,2.3387087462,0.9324639544,0.2666541404
H,0,2.4668555701,2.9053109344,1.1717769365
N,0,2.2561923042,-1.1956110006,-0.5174207824
H,0,1.8874970831,-1.9824089973,-1.0277497181
C,0,-1.6848480179,1.9746929116,-0.0705588265
C,0,-1.5582271412,3.2607924175,0.5087465282
N,0,-0.2821868759,3.3969748707,1.0076407742
H,0,0.1350611138,4.2869438271,1.2256864323

C,0,-2.7676744896,1.7707552884,-0.9323587387
H,0,-2.7674749433,0.9213368639,-1.6005548052
C,0,-3.7766251831,2.7194153486,-1.026481037
H,0,-4.6118967012,2.5490828294,-1.6969943009
C,0,-3.6983440589,3.9174713918,-0.3042627526
C,0,-2.5707244213,4.2176952975,0.4459735068
H,0,-4.4979628113,4.6468895767,-0.3820874818
H,0,-2.4549637992,5.1819581249,0.9303166624
C,0,-0.6675870858,-1.3615985238,0.1195105958
C,0,-2.0181207435,-1.9056610412,-0.0619484087
C,0,0.0362419309,-2.3886701465,0.7233228982
C,0,-1.9867182606,-3.2511967553,0.3942696844
C,0,-3.2755112981,-1.4480734111,-0.4996645007
C,0,-3.078248568,-4.1227152016,0.3676142125
C,0,-4.3685508167,-2.2957179999,-0.5258791361
H,0,-3.4297450149,-0.4222130454,-0.7759190476
H,0,-0.4276281274,-4.363775646,1.3011073551
C,0,-4.2769987288,-3.6335653794,-0.109033325
H,0,-2.9779554304,-5.1434566265,0.723550325
H,0,-5.3233371144,-1.9076256062,-0.8647929704
H,0,-5.1511654082,-4.2750111088,-0.1426639051
N,0,-0.7263811497,-3.5067424096,0.8669151047
H,0,1.0458806538,-2.3766789271,1.1023507121
E(RM062X) = -1164.83811987 A.U.

5c (R = H): transition state TS3

C,0,-4.7835978643,-3.3134052237,0.3849405962
C,0,-5.3524939868,-2.032683656,0.4595603454
C,0,-4.5901209999,-0.8890207833,0.2633351034
C,0,-3.2333376532,-1.0577900773,-0.0112728134
C,0,-2.6425236982,-2.3354789499,-0.0721320474
C,0,-3.4299576405,-3.4717835342,0.122756055
H,0,-5.4100689852,-4.1855185885,0.5376490547
H,0,-6.4122768588,-1.932626329,0.6707568142
H,0,-5.0343663578,0.0997842875,0.315758311
H,0,-2.9881941685,-4.4627557083,0.0756438113
C,0,-1.0265626196,-0.7246187419,-0.4206487041
C,0,0.2205190451,-0.127134734,-0.6623918639
C,0,1.3193274166,-1.004874447,-0.4100464069
C,0,1.099831987,-2.4121536821,-0.3615317894
C,0,-0.1540154394,-3.0055745975,-0.3552736393
C,0,-1.225476081,-2.1270100865,-0.2942082566
H,0,-0.2820447863,-4.0823206932,-0.3198907529
N,0,-2.2667230412,-0.1012311143,-0.2727850715
H,0,-2.3352591951,0.8418632115,0.0766594904
C,0,2.7459499529,-0.8337037369,-0.1157347986
C,0,3.3091886591,-2.1327239898,-0.0404823871
N,0,2.3191018365,-3.061833236,-0.2535922483
H,0,2.4108761074,-4.0396626839,-0.0327945161
C,0,3.5635520816,0.2383930494,0.2819047362
H,0,3.1560140322,1.2387888658,0.3668325735
C,0,4.8903007982,0.0184589044,0.6212735687
H,0,5.5069912475,0.8563858105,0.9280488315
C,0,5.4387446339,-1.2711774566,0.5910713965
C,0,4.6490138864,-2.3638169074,0.2734958243
H,0,6.4823777526,-1.4201282485,0.8478900753
H,0,5.0457335768,-3.373845609,0.2956686674
C,0,0.3889138706,1.2305247869,-1.2289041871
C,0,-0.5557750096,2.2642491057,-1.6811353625
C,0,1.6298469606,1.7098653752,-1.5907065002
C,0,0.2286181858,3.3383788954,-2.1817569876
C,0,-1.9518647632,2.4081913835,-1.8481053108
C,0,-0.2934279055,4.5302275477,-2.6892187464
C,0,-2.4820993969,3.5801497042,-2.3602921485
H,0,-2.6284492281,1.5910115824,-1.6524494901
H,0,2.3226399158,3.5017499346,-2.4608160911
C,0,-1.66648001,4.6517056806,-2.7574866636
H,0,0.3675053484,5.3185041494,-3.0361866532
H,0,-3.55774767,3.6586866554,-2.4793176265
H,0,-2.1130661652,5.5586542772,-3.1507281562
N,0,1.5449828927,2.962262859,-2.1194246951
H,0,2.5815569066,1.2102605983,-1.5607150704
E(RM062X) = -1164.85184803 A.U.

5e (R = Me): energy minimum M1

C,0,-5.2373252089,2.4701977437,0.0005290116
C,0,-5.6108868682,1.136739108,-0.2304180785
C,0,-4.6643187466,0.1321768248,-0.3730123907
C,0,-3.3188064977,0.492308032,-0.2789806507
C,0,-2.9245050387,1.8307909755,-0.0440046743
C,0,-3.8985603314,2.8219729147,0.0944078396
H,0,-6.0046843334,3.229307229,0.1065191729
H,0,-6.6641411484,0.8840357203,-0.2996450472
H,0,-4.9579652037,-0.896924747,-0.5521674867
H,0,-3.6098383536,3.8533531228,0.2747999899
C,0,-1.0619707733,0.4958133033,-0.2080284836
C,0,0.2765505959,0.0972300247,-0.2556324887
C,0,1.2190122382,1.1156907858,-0.0463124535
C,0,0.7999800709,2.4650466612,0.1417039655
C,0,-0.5326500574,2.8505301991,0.1682502937
C,0,-1.4759460356,1.8379254902,0.0002358805
H,0,-0.8263928639,3.8852525583,0.3136261639
N,0,-2.1901914938,-0.29100333,-0.3849190639
H,0,-2.1687685698,-1.2914828364,-0.5056015175
C,0,2.6701718445,1.1208314676,0.0520297609
C,0,3.0584364832,2.4664038772,0.2606079181
N,0,1.9293905747,3.2553787664,0.2975232364
H,0,1.9183560068,4.2406504638,0.4986084193
C,0,3.6574693364,0.1288594356,0.0279538085
H,0,3.379791857,-0.9135846684,-0.0873185595
C,0,4.9888075378,0.4912127835,0.1809617907
H,0,5.7584705913,-0.2730329815,0.1677476556
C,0,5.3519720132,1.8337922996,0.3616919566
C,0,4.395293494,2.8374392999,0.408117037
H,0,6.3994501376,2.0937197124,0.4761942183
H,0,4.6744181359,3.8748740366,0.5619556483
C,0,0.6208937746,-1.3036490816,-0.5470088691
C,0,0.1479876946,-2.4699151903,0.16020205
C,0,1.3853749197,-1.7575459824,-1.5966114584
C,0,0.6734131731,-3.6012405782,-0.5051835934
C,0,-0.6554083962,-2.6565454392,1.2963915258
C,0,0.4130767068,-4.9050856863,-0.0775141542
C,0,-0.9210882703,-3.947864087,1.7203686478
H,0,-1.051431333,-1.7974380385,1.8306847254
H,0,1.9088048905,-3.7117429563,-2.2307117848
C,0,-0.392530926,-5.0604614934,1.0388606714
H,0,0.8257911092,-5.7631259275,-0.5987148954
H,0,-1.5406615826,-4.1086457781,2.5963322995
H,0,-0.6169853577,-6.0596867881,1.397159783
N,0,1.4257905841,-3.1362539474,-1.5602054522
C,0,2.0880793867,-0.9934162677,-2.6667164656
H,0,3.1756632237,-1.035611328,-2.5425207051
H,0,1.8391356399,-1.3853580685,-3.6578278393
H,0,1.7908063696,0.0553673656,-2.620893358
E(RM062X) = -1204.18700210 A.U.

5e (R = Me): energy minimum M2

C,0,-6.0720803817,0.3319554754,0.3986502714
C,0,-5.9888851007,-1.049390599,0.1651728659
C,0,-4.7737029044,-1.6696396462,-0.0903821501
C,0,-3.6292480909,-0.8714457486,-0.1042232286
C,0,-3.693860326,0.5211341103,0.1367835291
C,0,-4.9301874737,1.1204324188,0.3854052662
H,0,-7.038858003,0.7836519328,0.5926116311
H,0,-6.8942743883,-1.6475922415,0.1835865175
H,0,-4.7150811824,-2.7378579731,-0.2723719171
H,0,-4.9946593093,2.1888649863,0.5693979843
C,0,-1.5101119525,-0.0962346236,-0.2222214469
C,0,-0.1211443613,-0.0266102781,-0.3451319832
C,0,0.4394692086,1.2471791764,-0.1619350252
C,0,-0.3906899163,2.3668031599,0.1369864953
C,0,-1.7715370411,2.2826532267,0.2529003461
C,0,-2.3345901767,1.0215697204,0.068295988
H,0,-2.3803661286,3.1518997505,0.4806161059
N,0,-2.3160554462,-1.2207741792,-0.3448685668
H,0,-1.9510218265,-2.1595094911,-0.3752622842
C,0,1.7994184391,1.7550576172,-0.2413105405
C,0,1.7306197544,3.1427655338,0.0300285438
N,0,0.4158151379,3.4872767901,0.2601260577
H,0,0.0844679726,4.4180109817,0.4474622107
C,0,3.0424252018,1.1823242979,-0.5357509187
H,0,3.1205448315,0.1241624154,-0.7576000788
C,0,4.1732699469,1.9870506249,-0.5395363263
H,0,5.1402662664,1.5505965949,-0.7648560587
C,0,4.0836234911,3.3574746548,-0.2547510223

C,0,2.8648032264,3.9549908087,0.031916688
H,0,4.9831950349,3.96476717,-0.2612339416
H,0,2.7933695851,5.0165986528,0.2458322446
C,0,0.6644357028,-1.2430600919,-0.6158343593
C,0,1.6844778761,-1.8213566773,0.2254143414
C,0,0.5238293609,-2.076548859,-1.7015844084
C,0,2.1288145971,-3.0008174405,-0.411526714
C,0,2.2577285205,-1.4427828762,1.4478899235
C,0,3.1269829379,-3.8118520795,0.1348816597
C,0,3.2484790734,-2.2411037761,1.9923949165
H,0,1.9307876848,-0.5323609629,1.9416141756
H,0,1.5244685308,-3.8620335397,-2.2589466899
C,0,3.6777731371,-3.4140628776,1.3417129523
H,0,3.45757592,-4.7162866615,-0.3660549587
H,0,3.7061932336,-1.9613493577,2.9353156227
H,0,4.4571460754,-4.0182170035,1.7944622313
N,0,1.4021770893,-3.1333827774,-1.574900272
C,0,-0.367463511,-1.9491349596,-2.8920561999
H,0,0.2089809768,-1.999005084,-3.8211742233
H,0,-1.1226210259,-2.742328968,-2.9229096038
H,0,-0.8861482667,-0.9899393268,-2.8588106503
E(RM062X) = -1204.18520715 A.U.

5e (R = Me): transition state TS1

C,0,-6.1155297079,0.5619380554,-0.0057213922
C,0,-6.0660487765,-0.6475280207,0.7049443991
C,0,-4.8605191838,-1.2812181797,0.9728643825
C,0,-3.6940364989,-0.6739595456,0.506072339
C,0,-3.7239020373,0.5480045075,-0.2027981243
C,0,-4.9502491813,1.1657357696,-0.4569597625
H,0,-7.075455917,1.0279879116,-0.1997804081
H,0,-6.9899340416,-1.0997264904,1.0509632629
H,0,-4.8261294756,-2.2167570119,1.5221667821
H,0,-4.9896962472,2.1058177632,-0.9992893423
C,0,-1.5407076467,-0.1512012255,0.0259752028
C,0,-0.1298681663,-0.1879305523,-0.0738818123
C,0,0.4170998608,1.1192964397,-0.2306858868
C,0,-0.3868333923,2.1541162596,-0.7986244623
C,0,-1.7548858491,2.0526963247,-0.9986180667
C,0,-2.3455870667,0.9105720323,-0.465241914
H,0,-2.336487852,2.8593557941,-1.4323196959
N,0,-2.3842696909,-1.0873260681,0.6138226066
H,0,-2.0526662352,-1.8157628804,1.224794331
C,0,1.6464306172,1.786956453,0.1853052539
C,0,1.5939687208,3.108408892,-0.3193329956
N,0,0.4017014403,3.2836289077,-0.9905424154
H,0,0.0077842005,4.1943137363,-1.1644021966
C,0,2.6315205144,1.4937917047,1.1402977056
H,0,2.619413008,0.542328513,1.6593484864
C,0,3.6053853767,2.4358047413,1.4389311707
H,0,4.3679766972,2.2011814691,2.1735443327
C,0,3.5983480041,3.69574201,0.8237869939
C,0,2.5814530531,4.0552483347,-0.0481633446
H,0,4.3729919898,4.4161511427,1.0658620497
H,0,2.5277749525,5.0524556178,-0.4730808572
C,0,0.5740885842,-1.4864251471,-0.0935032229
C,0,1.9984430328,-1.8277656646,-0.0086434557
C,0,-0.0693142532,-2.7012155671,-0.3557511692
C,0,2.0910576489,-3.2394250847,-0.0150653519
C,0,3.2166036038,-1.1332977313,-0.1275142777
C,0,3.2910965947,-3.948100022,0.0618855481
C,0,4.4191260423,-1.8199126377,-0.0780177655
H,0,3.2340224733,-0.0736760626,-0.322333683
H,0,0.6174166784,-4.6897754355,-0.4412589172
C,0,4.464095608,-3.2165365922,0.0550720626
H,0,3.2948625701,-5.0334171993,0.0844142265
H,0,5.3450920177,-1.262387265,-0.1724253611
H,0,5.4203204656,-3.7270328642,0.1005307358
N,0,0.8265372598,-3.7319452405,-0.2101112954
C,0,-1.375260392,-3.0594119265,-1.0060903152
H,0,-2.1481388985,-3.4076960188,-0.3150753164
H,0,-1.7826190504,-2.2079516129,-1.550320842
H,0,-1.1849391747,-3.8618947234,-1.725434342
E(RM062X) = -1204.13341364 A.U.

5e (R = Me): transition state TS3

C,0,-4.6704374396,-2.9598147118,0.3749358207
C,0,-5.0587673356,-1.8292881715,1.1116261857
C,0,-4.1818234644,-0.779613736,1.3456057412
C,0,-2.8952860667,-0.8832809885,0.8135133494
C,0,-2.4888203775,-2.0108482579,0.0661543766
C,0,-3.3878061986,-3.0584713474,-0.1450374754
H,0,-5.3824835013,-3.7616974972,0.2127443233
H,0,-6.0679587768,-1.7732044585,1.5069615152
H,0,-4.4843627725,0.0920346794,1.9166996289
H,0,-3.0872733025,-3.9347724221,-0.7119009527
C,0,-0.7464462424,-0.5048718085,0.1984847984
C,0,0.5205579223,0.0916692273,0.0002053617
C,0,1.5220116315,-0.8862306426,-0.2776176347
C,0,1.1478881039,-2.1426840876,-0.8415983489
C,0,-0.1595779344,-2.5948053653,-0.9242062367
C,0,-1.1021351118,-1.7937378872,-0.2891594395
H,0,-0.4130189883,-3.5555205195,-1.35999133
N,0,-1.8375991859,-0.0066121947,0.8967518421
H,0,-1.8698954787,0.9019300224,1.3286292071
C,0,2.9495827898,-1.0167501192,-0.0037528493
C,0,3.382030335,-2.2315908397,-0.5870805442
N,0,2.2987879891,-2.854741741,-1.1681122628
H,0,2.2848269568,-3.8417869436,-1.3687985744
C,0,3.8169493986,-0.3842429183,0.8961129862
H,0,3.4559073016,0.4443073707,1.4934594545
C,0,5.1106559638,-0.8557558462,1.0615668354
H,0,5.7776938737,-0.3614510677,1.7596418808
C,0,5.5538959388,-1.9867924518,0.3604885043
C,0,4.6890522795,-2.7013243759,-0.4552324254
H,0,6.5720034373,-2.3373708668,0.4949840073
H,0,5.0009549835,-3.6213578301,-0.9390557845
C,0,0.6317734671,1.5709434918,-0.0057449246
C,0,-0.5227695642,2.4687548566,-0.1854579083
C,0,1.7374887448,2.4195476739,0.0133812099
C,0,-0.052453904,3.7949161313,-0.0661449593
C,0,-1.8553177024,2.3227568297,-0.6313815764
C,0,-0.8590003928,4.928483865,-0.1839043003
C,0,-2.6635658537,3.4385502833,-0.7779670186
H,0,-2.2498113947,1.3540847535,-0.9057596259
H,0,1.9297231438,4.5155186017,0.0185060968
C,0,-2.1852417255,4.7339481835,-0.5214012022
H,0,-0.4437314435,5.9226834497,-0.0518658222
H,0,-3.6836255971,3.305868259,-1.1228386932
H,0,-2.8456281395,5.5872723117,-0.6336233146
N,0,1.3073288359,3.7252381462,0.0709488539
C,0,3.206765894,2.2675508276,-0.2408637887
H,0,3.8189835649,2.3293449025,0.6627311257
H,0,3.431282779,1.3349652897,-0.7481352426
H,0,3.5166205796,3.0825033302,-0.9027959496
E(RM062X) = -1204.13353013 A.U.

5.3. Variable Temperature NMR Experiment

According to the computational studies, the energy barrier for the rotation of the ICZ-indolyl bond in compound **5c** is *ca.* 20 kcal/mol, and in compound **5e** is *ca*. 35 kcal/mol (Figure S24A, main text and SI, section 4.2). To challenge these computational results, a variable temperature NMR experiment with compound **5g** was performed to check the coalescence of the diastereotopic ^{1}H NMR signals observed at room temperature (Figure S24B). ^{1}H NMR spectra of compound **5g** were acquired on a Bruker 400 MHz NMR spectrometer at increasing temperatures from 298 K (25 ºC) to 423 K (150 ºC, apparatus limitation). Lastly, a spectrum was acquired again at 25 ºC to exclude any degradation after the experiment.

No coalescence was observed between the signals of the methylene hydrogens, while we did observe coalescence with the -OH signal between 120 – 130 ºC (Figure S24C). In this regard, the last spectra at 25 ºC showed loss of multiplicity of the key signals, likely due to -OH exchange. Using the Eyring equation, the energy barrier was roughly estimated to be higher than 21 kcal/mol, consistently with the computational predictions (Figure S24D). Interestingly, the rise of temperature resulted in the downfield shift of one of the methylene hydrogens of about 0.1 ppm (Figure S24C).

Figure S24. A) Structure and predicted rotation energy barriers of compound **5c** and **5e**. B) Zoomed ^{1}H NMR spectra of compound **5g** at 25 ºC and highlighted diastereotopic signals. C) Variable temperature NMR experiment. D) Energy barrier estimation of compound **5g** using the Eyring equation.

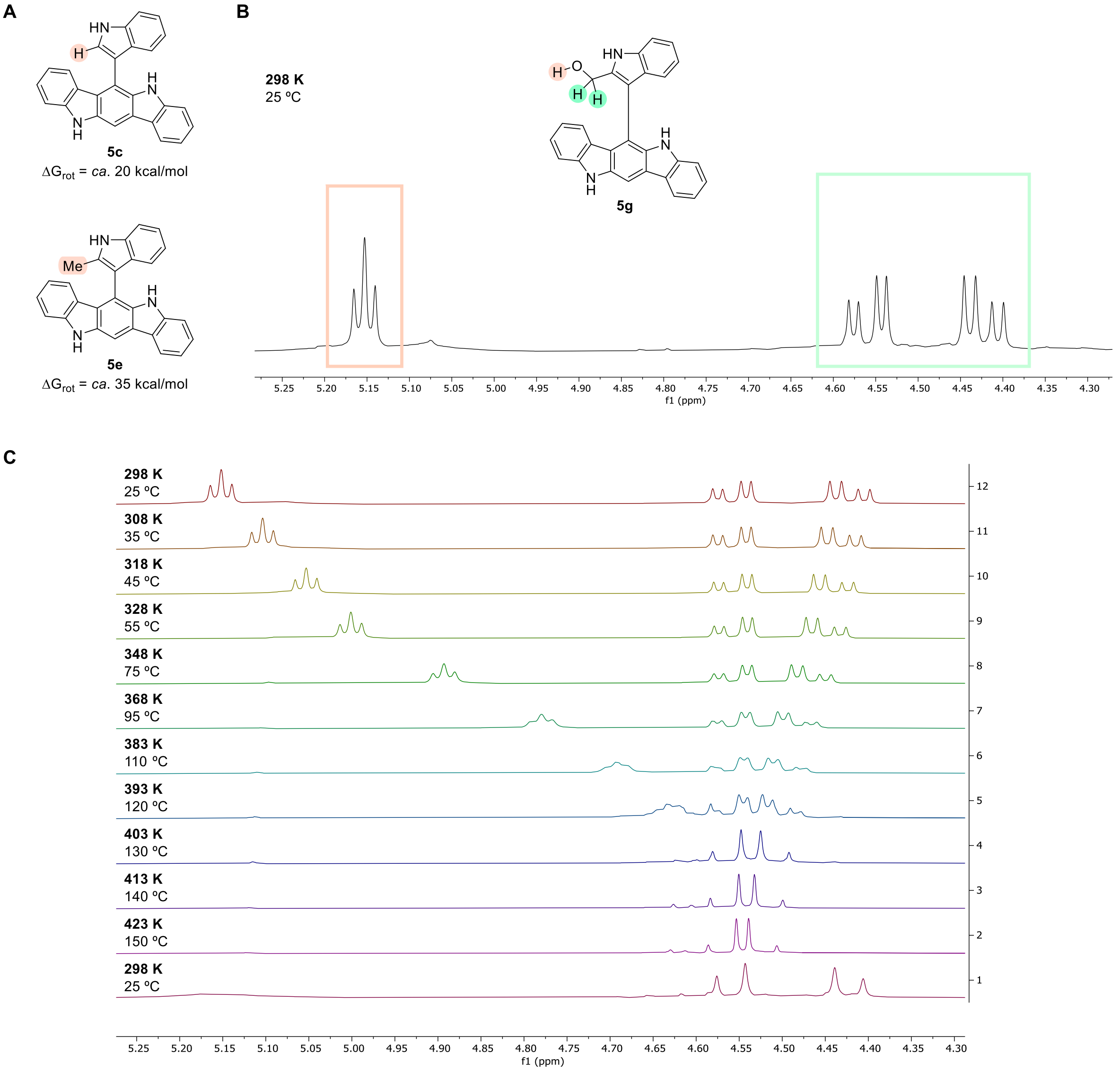

D

$$\Delta G^{\ddagger} = \alpha T_C \left[9.972 + \log\left(\frac{T_C}{\Delta\nu}\right)\right] \quad \begin{cases} \alpha = 4.575x10^{-3} \\ \Delta\nu_{298\,K} = 56\ Hz \\ T_C > 423\ K \end{cases} \quad \Rightarrow \quad \Delta G^{\ddagger} > 21\ kcal/mol$$

6. AhR-Modulating Activity

6.1. Computational Modelling

Figure S25. Binding mode of indirubin. A) The indirubin-bound cytosolic complex of AhR. AhR shown as blue surface (left) or cartoons (right). Indirubin shown as cyan sticks. B) Superposition of the cryo-EM structure of the AhR-indirubin complex (blue; PDB ID 7ZUB) and the last snapshot taken from the MD simulation (red). Ligand shown as cyan sticks. The surface of the interface of AhR with the neighboring subunits is shown in grey. C) Representation of the network of hydrogen bonds formed in the binding pocket of the AhR-indirubin complex. Distances (Å) denote the average value determined in the last 250 ns of the trajectory (the standard deviation generally varies between 0.2 and 0.4 Å). Labels denote the numbering of residues in the cryo-EM structure. D) Zoomed superposition of the cryo-EM structure of the AhR-indirubin complex and **5c**.

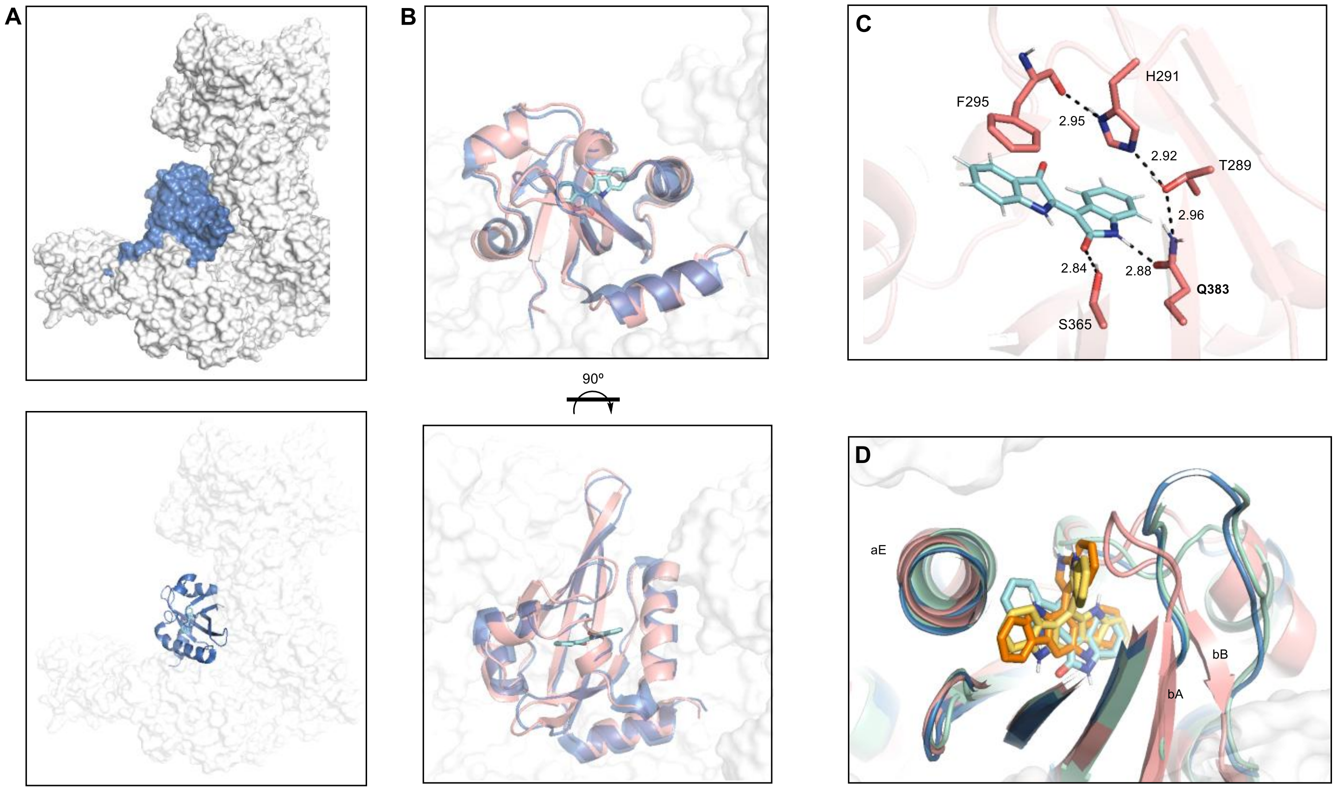

Figure S26. A) Time (ns) evolution of the root-mean square deviation (RMSD; Å) of the protein backbone along the trajectories obtained from MD simulations of the AhR complexes with compounds **5c** (green: M2, pose with N_{11}-H···O hydrogen bond, 2.2 ± 0.1 Å; blue: ent-M2, pose with C_7-H···O hydrogen bond 1.8 ± 0.2 Å) and **5j'** (magenta: 1.8 ± 0.2 Å). Values in parenthesis were determined along the last 250 ns of the trajectory. B) Structures of compounds **5c** (M2 and ent-M2) and **5j'**.

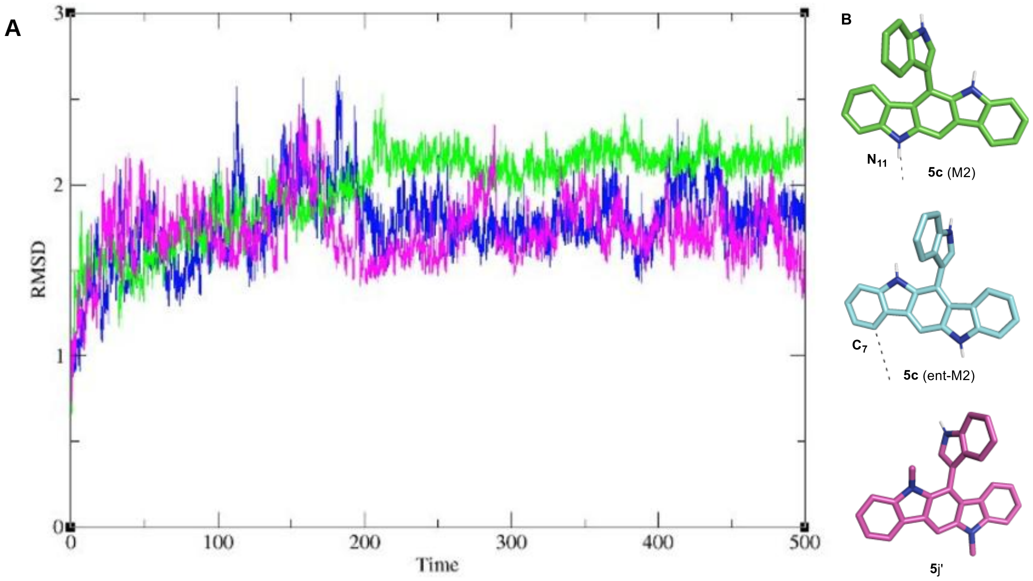

The proposed binding mode suggests that the enantiomeric atropoisomers of **5e** and **5j** may bind with similar affinities mimicking the poses shown in Figure 5c-d. This is supported by the slight decrease in potency observed upon methylation at position C2 of the indole ring, as noted in the 1.4– and 3.2–fold increase in the EC_{50} values determined for compounds **5e** and **5j** relative to **5c** (Figure 5f). This would enable the methyl group at the indole ring to be easily accommodated upon a slight displacement of the side chain of Y322 (Figure S27)."

Figure S27. Effect of methylation at position C2 of the indole ring on the binding mode proposed for **5c** (left: **M2**, yellow sticks; right: **ent-M2**, orange sticks). Selected residues located at the entrance of the pocket are highlighted as sticks. Attachment of the methyl group at position C2 (shown as spheres) can be easily accommodated upon a slight shift in the location of the side chain of Tyr322. Labels denote the numbering of residues in the cryo-EM structure.

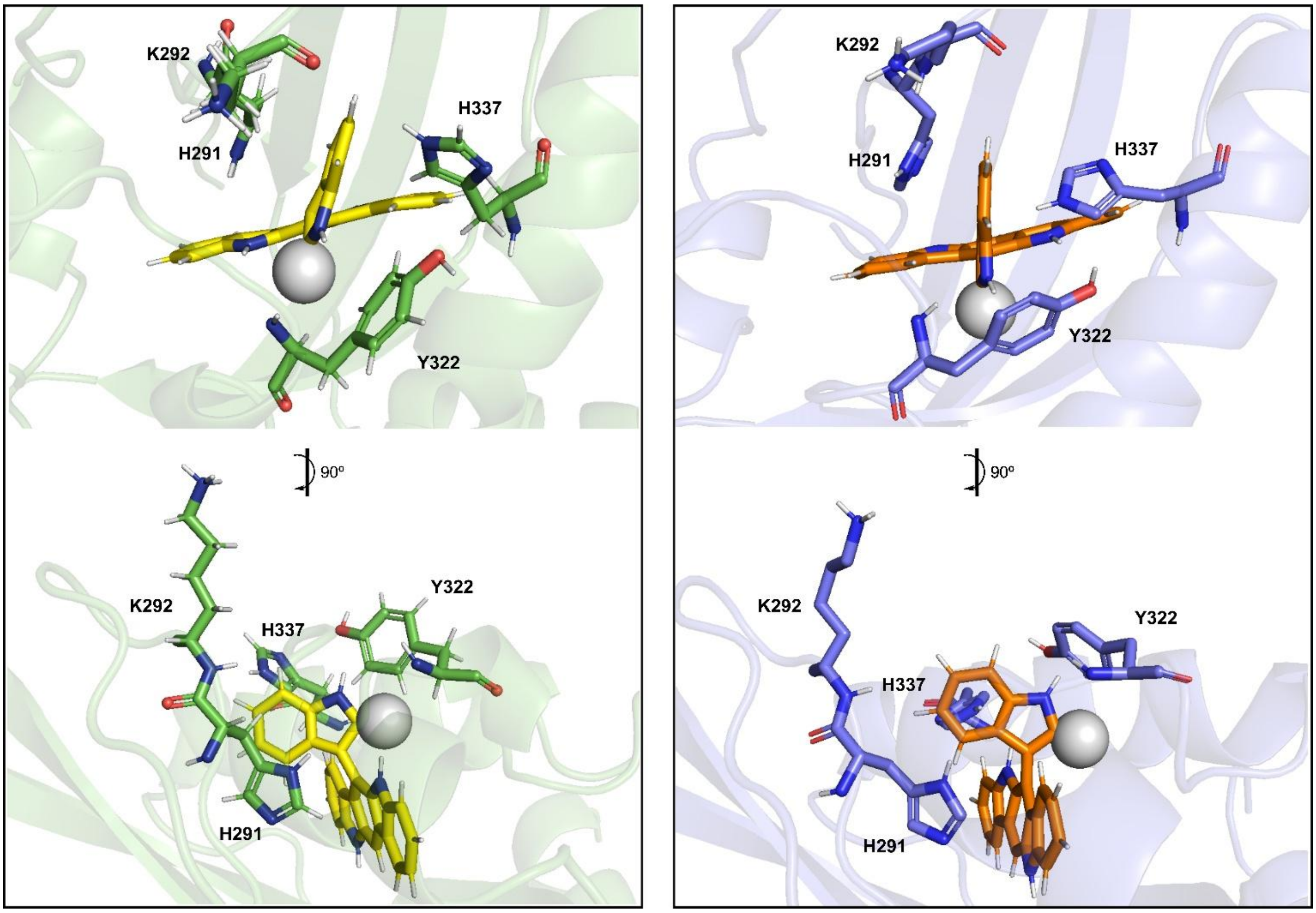

6.2. Biological Assays

6.2.1. Detailed Results

Figure S28. XRE-HepG2 cells were treated with DMSO (0.1 to 0.2%), Tapinarof (0.003, 0.01, 0.03, 0.1, 0.3, 1, 3 or 10 µM), TCDD (0.0001, 0.0003, 0.001, 0.003, 0.01, 0.03, 0.1, 0.3, 1 µM) or compounds **4a**-**d**, **5a**-**m**, **8**, **9a**, **10**, and **12a**-**d** (0.01, 0.03, 0.1, 0.3, 1, 3, 10 or 20 µM). After 8 h, AhR/XRE-dependent luciferase activity was determined. EC_{50} values (mean effective concentration, causing 50% induction of luciferase activity) were calculated with GraphPad prism (version 8.1.2) according to the equation y = bottom + (xHill slope) x (top – bottom)/(xHill slope + EC_{50}Hill slope). n = 3 – 4. Data are shown as mean ± SEM.

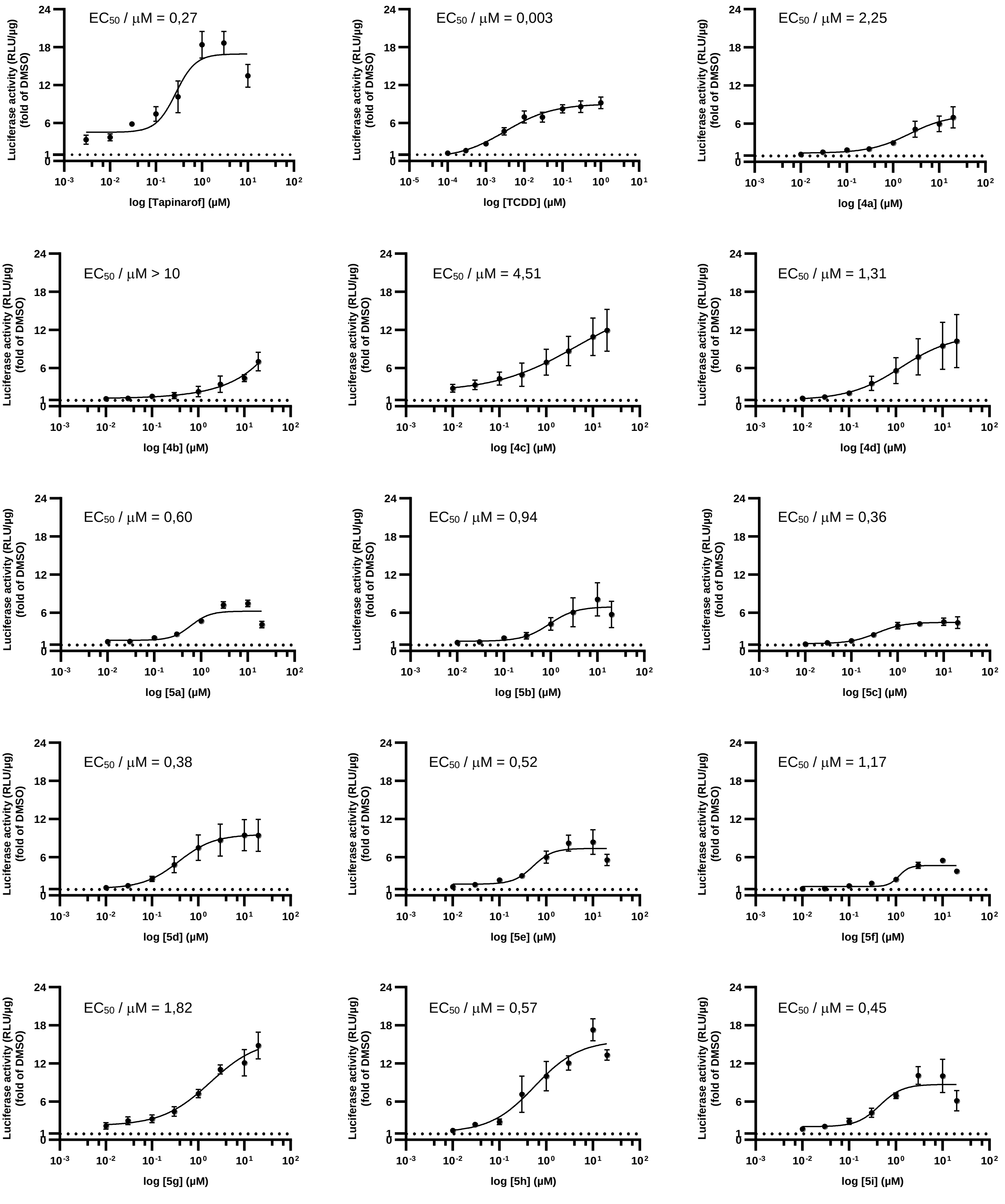

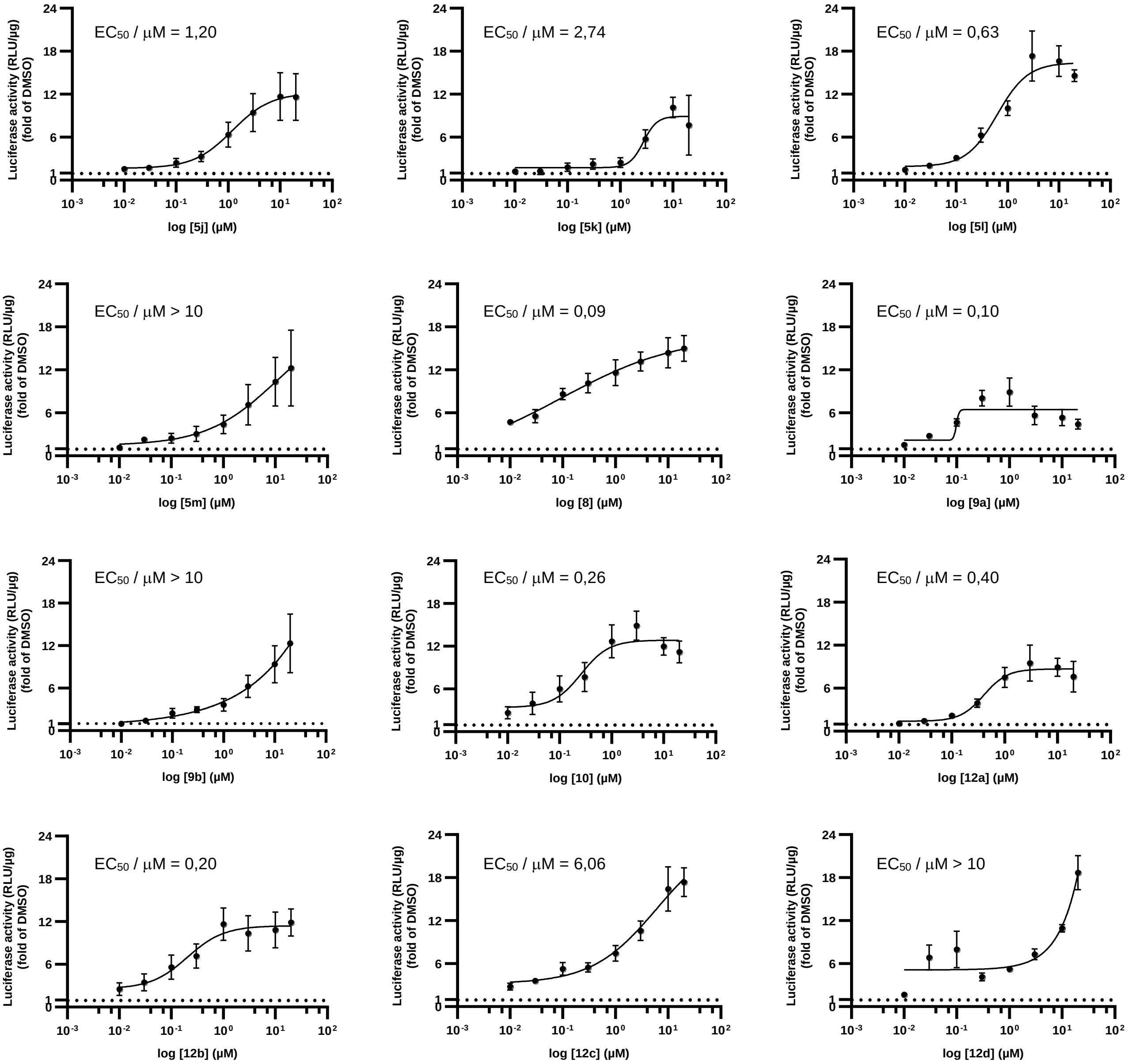

Figure S29. *CYP1A1* (A) and *CYP1B1* (B) induction expressed as fold of 0.1% DMSO control (-) in HaCaT wt cells (grey) *vs*. HaCaT AhR-deficient cells (white). 8 h treatment. Used concentrations. ICZs and **T**: 3 µM. **BaP**: 2,5 µM. n = 3 – 4. For statistical analysis a two-way ANOVA (Tukey post-hoc) was performed and data are shown as mean ± SEM. (*, p ≤ 0.05).

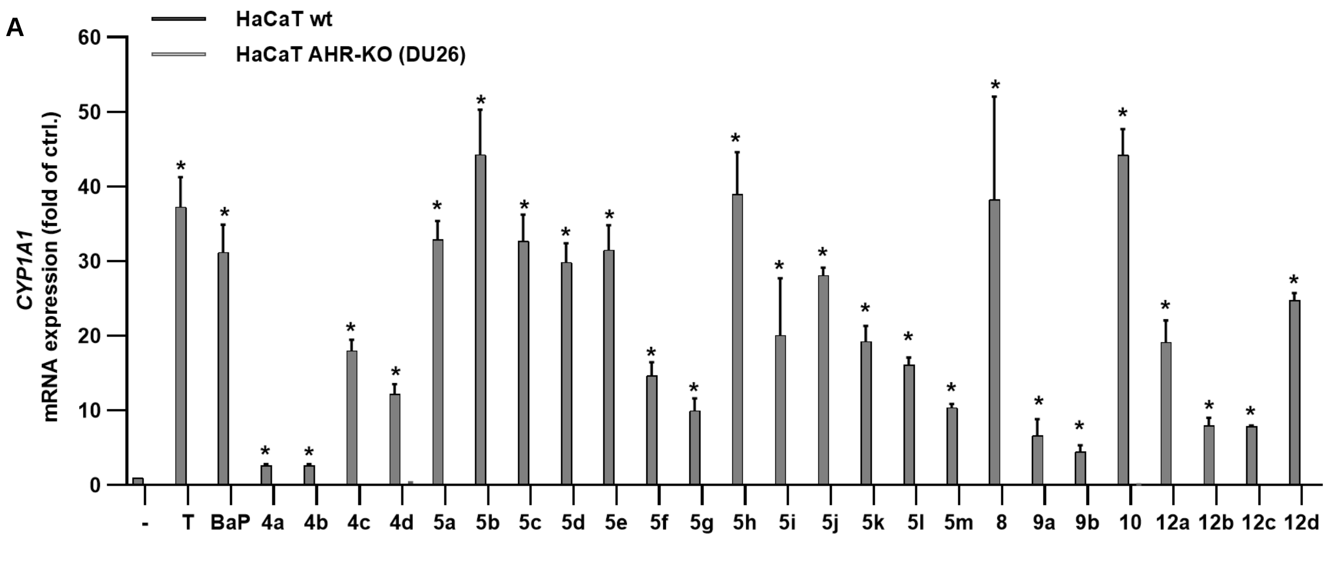

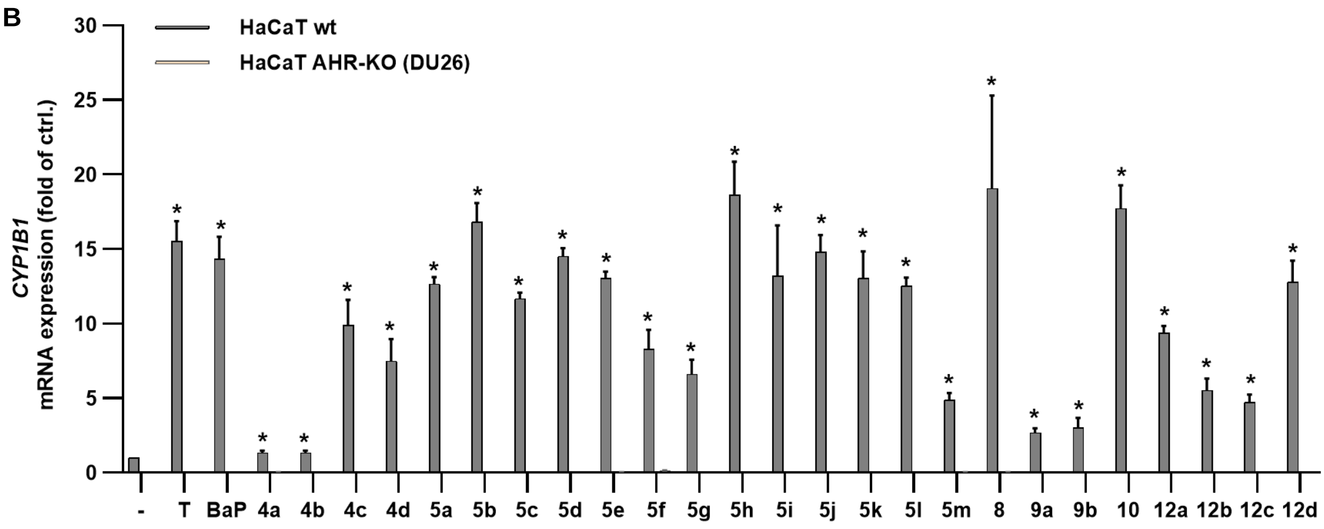

Figure S30. Agonists induce degradation of AhR. Western Blot of Anti-AhR and Anti-Tubulin of MCF7 cells treated with vehicle (DMSO), Tapinarof, **5a**, **5d**, **5e**, **5k**, **12b**, **12c**, and **12d** at a concentration of 10 µM for 5h. The numbers on the Blot indicate the signal intensities of the bands relative to the Tubulin bands, normalized to DMSO treatment as quantified with Empiria Studio.

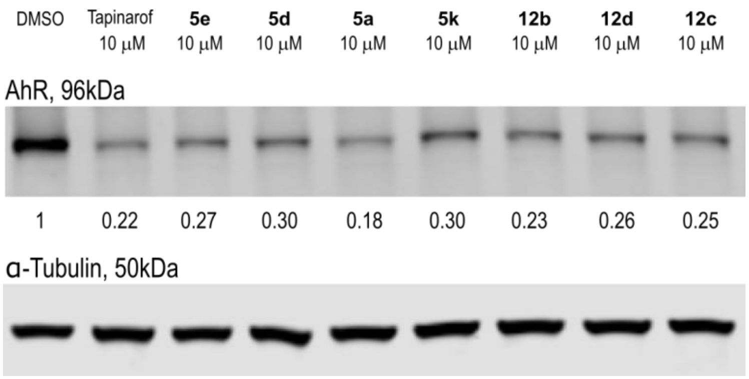

Figure S31. Change in AhR levels relative to vehicle (DMSO) treatment in both a full length AhR (MCF7 AhR-GFP.IRES.mCherry) and a PAS-B deleted (MCF7 AhRΔPasB(Δ275-342)-GFP.IRES.mCherry) reporter cell line treated with Tapinarof (**T**), **12b**, and **12c** at a concentration of 10 µM for 3h., in the presence or absence of TAK243 (1 µM) and MLN4924 (1 µM). Values represent the ratio of the geometric mean of GFP and mCherry values in the gated population.

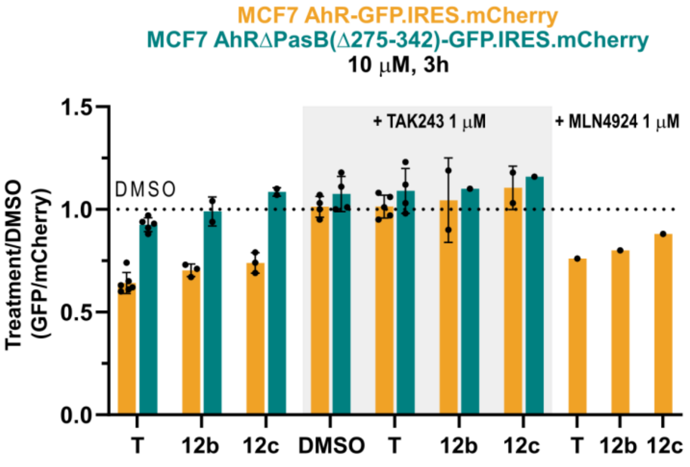

Figure S32. Agonist-induced selective degradation of AhR in MCF7 cells. Change in protein levels relative to vehicle treatment (DMSO) in MCF7 cells treated with Tapinarof at 10 µM for 5 h quantified by TMT labelling and LC MS/MS analysis versus p-Value.

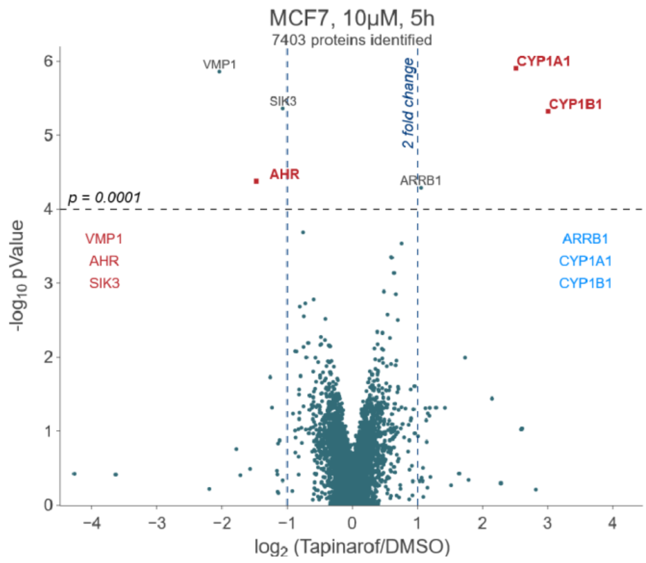

Figure S33. HaCaT keratinocytes were treated with DMSO (0.1%) or compound **12b**, **12c** or **12d** (1 or 10 µM). After 8 h or 24 h, Western Blot analysis of AhR protein level was performed. GAPDH level served as loading control. In the upper panel representative blots, in the lower panel the densitometric quantification is shown. n = 3 – 4. For statistical analysis a 1-way ANOVA (post-hoc: Dunnett's test) was performed and data are shown as mean ± SEM. (*, p ≤ 0.05 compared to the respective DMSO control).

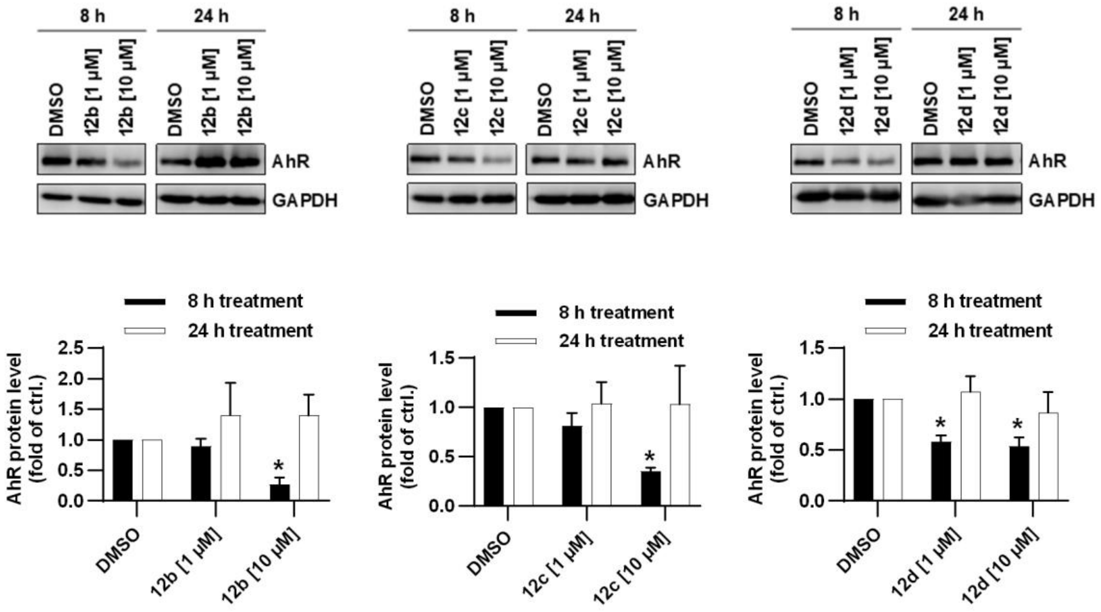

Figure S34. Co-immunoprecipitation assays demonstrating the impact of the compound **12d** on the physical interaction of AhR with Cullin 4B (CUL4B) and AhR-interacting protein (AIP, a component of the inactive cytoplasmatic AhR multiprotein complex that served as positive control). HaCaT-EV keratinocytes were treated with DMSO (0.1%) or compound **12d** [10 µM] for 30 min or 60 min. HaCaT-shAhR cells served as control. Afterwards, Western Blot analysis of AhR, AIP and CUL4B protein level was performed. Levels of the membrane-cytoskeletal protein vinculin, a stably expressed housekeeping protein, served as loading control. Additionally, co-IP analysis with anti-AhR antibody was performed. Representative Blots. n = 2.

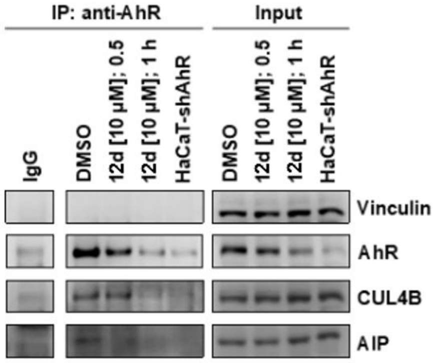

6.2.2. Detailed Methods

Cell culture and treatment

Human HaCaT and HaCaT-AhR-deficient keratinocytes were cultured in DMEM low glucose (1 g/l) medium (PAN Biotech, Aidenbach, Germany) supplemented with 10% (v/v) fetal bovine serum (PAN Biotech) and 1% (v/v) antibiotics/antimycotics (PAN Biotech). The MCF7 cells were purchased from ATCC and grown in DMEM with 10% FCS and 1% penicillin/ streptomycin. The CRISPR/Cas12-based generation and characterization of the HaCaT-AhR-deficient cells (clone DU26) as well as the lentiviral-mediated generation of AHR-knockdown (HaCaT-shAhR) and respective empty vector control (HaCaT-EV) cells has been previously described.[28,29] The culture medium of the HaCaT-shAhR and HaCaT-EV cells was additionally supplemented with 0.84 mg/ml G418 (Roth, Karlsruhe, Germany). The human XRE-HepG2 reporter cell-line was a kind gift of K. Gradin and L. Poellinger (Karolinska Institute, Stockholm, Sweden). The cells were cultured in RPMI 1640 (PAN Biotech) containing 3.7% $NaHCO_3$ (w/v), 10% fetal bovine serum (v/v), 1% (v/v) antibiotic/antimycotic solution, and 0.8 mg/ml G418. All cells were cultivated at 37 °C in a humidified atmosphere containing 5% CO_2. Tapinarof (MedChemExpress, Monmouth Junction, NJ), benzo[a]pyrene (Sigma-Aldrich, Taufkirchen, Germany), TCDD (Amchro, Hattersheim am Main, Germany) and all compounds synthesized in this study were dissolved in dimethyl sulfoxide (DMSO; Roth). Recombinant human IL-13 (PeproTech, Cranbury, NJ, USA) were dissolved in PBS containing 0.01% BSA. Cells were treated with different concentrations of the compounds for different time periods as indicated in the figure legends. For the IL-13 co-treatment, cells were grown confluent for 72 h prior treatment.

Quantitative real-time PCR

The isolation of total RNA, reverse transcription, and quantitative real-time PCR was conducted as described previously.[26] Transcript level of CYP1A1, CYP1B1, CCL26, IL-19 and CXCL1 were normalized to β-actin expression. Primer sequences are as follows:

- **CYP1A1**: TTCATGCAGAAGATGGTC (forward (5'>3')); TCTCCTGACAGTGCTCAATC (reverse (5'>3')).
- **CYP1B1**: CGGCTGGATTTGGAGAACGTA (forward (5'>3')); TGATCCAATTCTGCCTGCACT (reverse (5'>3')).
- **CCL26**: GGGAGTGACATATCCAAGACCTG (forward (5'>3')); CAGACTTTCTTGCCTCTTTTGGTA (reverse (5'>3')).
- **IL-19**: CGTTCTACGTGGACAGGGTGTT (forward (5'>3')); CAGTGACACTGCCTCTGTTCCT (reverse (5'>3')).
- **CXCL1**: GCCCAAACCGAAGTCATAGCC (forward (5'>3')); ATCCGCCAGCCTCTATCACA (reverse (5'>3')).
- **β-actin**: CCCCAGGCACCAGGGCGTGAT (forward (5'>3')); GGTCATCTTCTCGCGGTTGGCCTTGGGGT (reverse (5'>3')).

Reporter gene assay

AhR-dependent reporter gene activity was determined in human HepG2 cells stably transfected with an XRE-harboring luciferase plasmid (XRE-HepG2 cells). The assay was carried out as described earlier.[30] Luciferase activity was normalized to protein content.

SDS-PAGE/Western blot analysis

Cells were lysed in RIPA buffer on ice and subsequently centrifuged for 15 min at 4 °C at maximum speed. Protein samples were separated by 10% and 12% SDS-polyacrylamide gel electrophoresis and blotted onto PVDF (GE Healthcare, Freiburg, Germany) or Nitrocellulose (BioRad #1704158) membranes. Blots were blocked with 5% skim milk or bovine serum albumin in TBS-Tween-20 (0.1%) for or Intercept® (TBS) Blocking Buffer (LI-COR Biosciences) 1 h at room temperature and subsequently incubated overnight at 4 °C with primary antibodies. Blots were washed and then incubated for 1 h with a 1:5000 dilution of HRP-conjugated secondary antibodies in 5% bovine serum albumin in TBS-Tween-20 (0.1%) or anti-Mouse (Licor, IRDye® 680RD Goat anti-Mouse IgG, 1:10k dilution) or anti-Rabbit secondary antibody (Licor, IRDye® Goat anti-Rabbit IgG, 1:10k dilution) at room temperature. Bands were visualized using WesternBright ECL HRP substrate (Advansta, San Jose, CA) and a C-DiGit Western Blot Scanner (LI-COR Biotechnology, Lincoln, NE) or a Licor Odyssey CLx imager. Primary antibodies used in this study are as follows. **AhR** (Cat.No. 83200; Cell Signaling Technology, Leiden, The Netherlands). **AIP** (Cat.No. sc-59730; Santa Cruz Biotechnology, Dallas, TX). **Cul4B** (Cat.No. abx004735; Abbexa, Houston, TX). **GAPDH** (Cat.No. 2118; Cell Signaling Technology). **Vinculin** (Cat.No. 13901, Cell Signaling Technology). α-Tubulin (Abcam, ab7291, 1:10k dilution).

Co-immunoprecipitation

HaCaT-EV and HaCaT-shAhR cells were treated with either vehicle (DMSO) or 10 µM 12d for 0.5 h and 1 h. Prior to cell lysis, proteins were intracellularly crosslinked with the cell permeable disuccinimidyl suberate (DSS, 2 mM) from Thermo Fisher Scientific (Waltham, MA) according to the manufacturer's instructions. The quenching reaction was carried out 30 min later with Tris, pH 7.5 at a final concentration of 10 mM for 15 min. Subsequently, cells were lysed with Pierce IP-lysis buffer (Thermo Fisher Scientific, Waltham, MA) containing protease and phosphatase inhibitor cocktail. For co-immunoprecipitation (co-IP) procedure, 1 mg of whole cell lysate was used. Initially, the double amount of 35 µl protein A/G agarose beads (Santa Cruz Biotechnology) was incubated for at least 1 h on a rotating wheel in 500 µl Pierce IP-lysis buffer and was then centrifuged at 2500 x g for 5 min at 4 °C. The supernatant was carefully removed, and beads were washed with IP-lysis buffer. Whole cell lysates were then added to the beads and incubated for 45 min at 4 °C on a rotation wheel, prior to centrifugation at 2500 x g at 4 °C for 10 min. 4 µl of AhR antibody (Cell Signaling Technology) were added to the collected supernatant and incubated on a rotating wheel for at least 1 h at 4 °C, before addition of 35 µl of freshly washed protein A/G beads. Rabbit IgG (Cell Signaling Technology) was used as negative control. Please note that according to the manufacturer rabbit IgG in combination with the respective mouse anti-rabbit IgG (Cat.No. 3678, Cell Signaling Technology) may result in an unspecific Western blot band in the height of approx. 100 kDa which is close to the molecular weight of AhR and Cul4B. Next, samples were incubated overnight on a rotating wheel at 4 °C. Elution was carried out by centrifugation at 2500 x g at 4 °C for 5 min to remove supernatant followed by 4 washing steps with IP-lysis buffer. Finally, the samples were resuspended in 50 µl 4x laemmli sample buffer containing reducing agent (250 mM Tris-HCl, pH 8.0; Glycerol, 20% (w/v); SDS, 5% (w/v); bromophenol blue, 0.001% (w/v); 100 mM dithiothreitol; 16% β-mercaptoethanol) and boiled for 10 min at 100 °C. After centrifugation at 10.000 x g for 5 min, the supernatant was used for SDS-PAGE/Western blot analysis. For each SDS-Page 20 µl of the supernatants were loaded.

Generation of reporter cell lines

The MCF7.AhR-GFP.IRES.mCherry and MCF7.AhRdPasB(d275-342)-GFP.IRES.mCherryreporter cell line was generated via lentivirus transduction. The coding sequence of the full length AhR (NM_001621.5), as well as the PasB deleted AhR (d275-342) were synthesized and cloned into a Lenti virus transfer plasmid (Artichoke, Addgene #73320), with hereby the resistance was swapped from puromycin to hygromycin. To produce the virus, 550k Lenti-X 293T cells (Takeda #632180) were seeded in a six-well plate with 3 mL of growth medium. The following day, a plasmid solution containing psPAX2 (500 ng), pVSV-G (5 ng), and AhR transfer plasmid (500 ng) was diluted in 21.5 µL of OPTIMEM, reaching a total volume of 32.5 µL. A second solution consisting of 9 µL OPTIMEM and 9 µL TransIT-LT1 was prepared, incubated at room temperature for 5 minutes, and then gently mixed into the plasmid solution. The mixture was incubated at room temperature for 30 minutes before being added to the Lenti-X cells. The cells were allowed to grow for 48 hours, after which the virus-containing supernatant was centrifuged (800g, 2min), aliquoted in 300 µL volumes, and stored at -80°C.

For the transduction, 3 million MCF7 cells were suspended in 2.7 mL of growth medium in a six-well plate, and 300 µL of a previously thawed virus aliquot was added. The plate was centrifuged at 800 g for 2 hours at 37°C, then incubated for 48 hours. Following this, the cells were subjected to selection with hygromycin (100 µg/mL) for a week. Hereafter the cells were expanded and sorted for GFP and mCherry positive cells by FACS (BD FACSAria). The sorted cells were further expanded in standard growth media and used for flow cytometry experiments.

Flow cytometry experiments

For the flow cytometry experiments 150k cells were seeded in a 24 well-plate format in 0.5 mL growing medium and left to grow in the incubator for 16 h. They were then treated with compounds at the indicated concentration and for the indicated duration. The media was aspirated, the cells washed once with PBS and 0.05% Trypsin was added to detach the cells. The trypsin was neutralizied by the addition of growing medium and the cells collected via centrifugation (400 g for 2 min) and resuspended in 150uL PBS. They were subsequently measured on a BD LSR II and the data analyzed with FlowJo software (version 10.5.3, TreeStar).

Sample preparation for TMT and PRM LC-MS/MS analysis

500k cells were lysed in 50 µl lysis buffer (1% sodium deoxycholate (SDC), 0.1 M TRIS, 10 mM TCEP, pH = 8.5) using strong ultra-sonication (10 cycles, Bioruptor, Diagnode). Sample aliquots containing 60 µg of total proteins were reduced for 10 min at 95 °C and alkylated at 15 mM chloroacetamide for 30 min at 37 °C. Proteins were digested by incubation with sequencing-grade modified trypsin (1/50, w/w; Promega, Madison, Wisconsin) for 12 h at 37°C. Tryptic digests were acidified (pH<3) using TFA and desalted cleaned up using iST cartridges (PreOmics, Munich) according to the manufacturer's instructions. Samples were dried under vacuum and stored at -20 °C until further use.

TMT labelling

Sample aliquots comprising 10 µg of peptides were labeled with isobaric tandem mass tags (TMTpro 18-plex, Thermo Fisher Scientific). Peptides were resuspended in 10 µl labeling buffer (2 M urea, 0.2 M HEPES, pH 8.3) by sonication and 2.5 µl of each TMT reagent were added to the individual peptide samples followed by a 1 h incubation at 25°C shaking at 500 rpm. To quench the labelling reaction, 0.75 µl aqueous 1.5 M hydroxylamine solution was added, and samples were incubated for 5 min at 25°C shaking at 500 rpm followed by pooling of all samples. The pH of the sample pool was increased to 11.9 by adding 1 M phosphate buffer (pH 12) and incubated for 20 min at 25°C and 500 rpm shaking to remove TMT labels linked to peptide hydroxyl groups. Subsequently, the reaction was stopped by adding 2 M hydrochloric acid until a pH < 2 was reached. Finally, peptide samples were further acidified using 5 % TFA, desalted using BioPureSPN MACRO™ SPE cartridges (Nest group) according to the manufacturer's instructions and dried under vacuum.

TMT LC-MS/MS analysis

TMT-labeled peptides were fractionated by high-pH reversed phase separation using a XBridge Peptide BEH C18 column (3.5 µm, 130 Å, 1 mm x 150 mm, Waters) on an Ultimate 3000 system (Thermo Scientific). Peptides were loaded on column in buffer A and the system was run at a flow of 42 µl/min. The following gradient was used for peptide separation: from 2% B to 15% B over 3 min to 45% B over 59 min to 80% B over 3 min followed by 9 min at 80% B then back to 2% B over 1 min followed by 15 min at 2% B. Buffer A was 20 mM ammonium formate in water, pH 10 and buffer B was 20 mM ammonium formate in 90% acetonitrile, pH 10. Elution of peptides was monitored with a UV detector (205 nm, 214 nm) and a total of 36 fractions were collected, pooled into 12 fractions using a post-concatenation strategy as previously described[31] and dried under vacuum.

Dried peptides were resuspended in 0.1% aqueous formic acid and subjected to LC–MS/MS analysis using an Orbitrap Eclipse Tribrid Mass Spectrometer fitted with a Ultimate 3000 nano system, a FAIMS Pro interface (all Thermo Fisher Scientific) and a custom-made column heater set to 60°C. Peptides were resolved using a RP-HPLC column (75 µm × 30 cm) packed in-house with C18 resin (ReproSil-Pur C18–AQ, 1.9 µm resin; Dr. Maisch GmbH) at a flow rate of 0.3 µl/min. The following gradient was used for peptide separation: from 2% B to 12% B over 5 min to 30% B over 70 min to 50% B over 15 min to 95% B over 2 min followed by 18 min at 95% B then back to 2% B over 2 min followed by 18 min at 2% B. Buffer A was 0.1% formic acid in water and buffer B was 80% acetonitrile, 0.1% formic acid in water.

The mass spectrometer was operated in DDA mode with a cycle time of 3 s. Throughout each acquisition, the FAIMS Pro interface switched between CVs of −40 V and −70 V with cycle times of 1.5 s and 1.5 s, respectively. MS1 spectra were acquired in the Orbitrap at a resolution of 120,000 and a scan range of 400 to 1600 m/z, AGC target set to "Standard" and maximum injection time set to "Auto". Precursors were filtered with precursor selection range set to 400–1600 m/z, monoisotopic peak determination set to "Peptide", charge state set to 2 to 6, a dynamic exclusion of 45 s, a precursor fit of 50% in a window of 0.7 m/z and an intensity threshold of 5e3.

Precursors selected for MS2 analysis were isolated in the quadrupole with a 0.7 m/z window and collected for a maximum injection time of 35 ms with AGC target set to “Standard”. Fragmentation was performed with a CID collision energy of 30% and MS2 spectra were acquired in the IT at scan rate “Turbo”.

MS2 spectra were subjected to RTS using a human database containing 20362 entries downloaded from Uniprot on 20200417 using the following settings: enzyme was set to “Trypsin”, TMTpro16plex (K and N-term) and Carbamidomethyl (C) were set as fixed modification, Oxidation (M) was set as variable modifications, maximum missed cleavages was set to 1 and maximum variable modifications to 2. Maximum search time was set to 100 ms, the scoring threshold was set to 1.4 XCorr, 0.1 dCn, 10 ppm precursor tolerance, charge state 2 and “TMT SPS MS3 Mode” was enabled. Subsequently, spectra were filtered with a precursor selection range filter of 400–1600 m/z, precursor ion exclusion set to 25 ppm low and 25 ppm high and isobaric tag loss exclusion set to “TMTpro”. MS/MS product ions of precursors identified via RTS were isolated for an MS3 scan using the quadrupole with a 2 m/z window and ions were collected for a maximum injection time of 200 ms with a normalized AGC target set to 200%. SPS was activated and the number of SPS precursors was set to 10. Isolated fragments were fragmented with normalized HCD collision energy set to 55% and MS3 spectra were acquired in the orbitrap with a resolution of 50,000 and a scan range of 100 to 500 m/z.

The acquired raw-files were analysed using the SpectroMine software (v 3.2, Biognosis AG, Schlieren, Switzerland). Spectra were searched against a human database consisting of 20372 protein sequences (downloaded from Uniprot on 20220222). Standard Pulsar search settings for TMT 16 pro (“TMTpro_Quantification”) were used and resulting identifications and corresponding quantitative values were exported on the PSM level using the “Export Report” function. Acquired reporter ion intensities were employed for automated quantification and statistical analysis using the in-house developed SafeQuant R script (v2.3).[32] This analysis included adjustment of reporter ion intensities, global data normalization by equalizing the total reporter ion intensity across all channels, data imputation using the knn algorithm, summation of reporter ion intensities per protein and channel and calculation of protein abundance ratios. To meet additional assumptions (normality and homoscedasticity) underlying the use of linear regression models and t-tests, MS-intensity signals were transformed from the linear to the log-scale. The summarized protein expression values were used for statistical testing of between condition differentially abundant proteins. Here, empirical Bayes moderated t-tests were applied, as implemented in the R/Bioconductor limma package (http://bioconductor.org/packages/release/bioc/html/limma.html). The resulting per protein and condition comparison p-values.

7. Toxicity Studies

7.1. Cytotoxicity

Cell viability method

A phosphatidylserine exposure was used to determine cell viability. Cells were washed with PBS, pelleted, and then incubated with annexin binding buffer containing Annexin V-APC (Life Technologies) for 15 min in the dark. Then, samples were analyzed by flow cytometry using FACSCanto II and FACSDiva (BD Biosciences, Franklin Lakes, NJ, USA). Data represents the percentage of non-apoptotic cells (Ann-APC$^-$).

Detailed Results

The cytotoxicity of a selection of the synthesized compounds was assessed after 24 hours of treatment in two human cell lines, Jurkat and HEK293T, coming from either tumoral T-cell lymphocytes or non-tumoral embryonic kidney fibroblasts, respectively. Interestingly, the pro-apoptotic activity was not significant below 10 μM for most compounds in both cell lines, except for **5i**, **5k** and **5l** in both cell lines, as well as **9a** in Jurkat and **5e** HEK293T cells (Figure S35A-B).

Moreover, the cytotoxicity of compounds **4d**, **5b**, **5h**, **8**, **10**, and **12d** at a higher concentration (100 μM) was assessed in both cell lines, Jurkat and HEK293T. The cytotoxicity of compounds **5b**, **5h**, and **12d** could not be properly tested as the compounds were not soluble in the growing media at the concentration of 100 μM. Note that a slight turbidity was observed in the media during the analysis of compounds **8** and **10**. Interestingly, these compounds exhibited a minimal pro-apoptotic activity at 100 μM, with only slight cytotoxic effects observed for compound **4d** in Jurkat cells and compound **8** in HEK293T cells (Figure S35C).

Figure S35. A) Jurkat and B) HEK293T cells were treated with 1 and 10 µM doses of the corresponding compound for 24 hours. Indicated doses of fluorizoline were used as a positive control of cell death. C) Jurkat and HEK293T cells were treated with 100 µM of the corresponding compound for 24 hours. Viability was measured by flow cytometry, and it is expressed as the mean ± SEM (n = 3) of the percentage of non-apoptotic cells (Ann-APC$^-$). * $p < 0.05$, ** $p < 0.01$ *** $p < 0.001$.

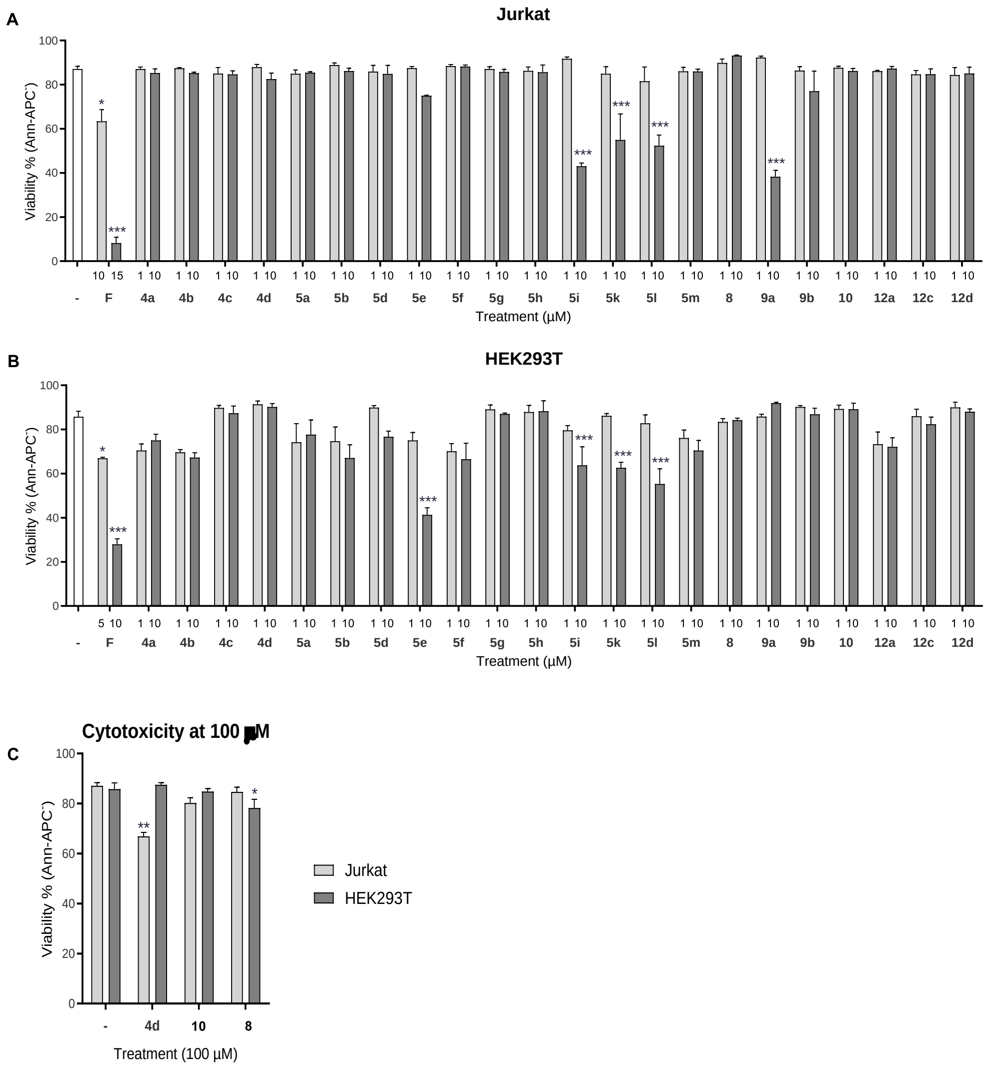

7.2. Phototoxicity

Irradiation with UVA and caspase 3 activity measurement

HaCaT cells were incubated with DMSO (0.1%) or **FICZ**, Tapinarof (**T**), **8**, **10**, **5b**, **5h**, **4b** (100 nM or 1 μM) for 1 h. Afterwards the cell culture media was exchanged with PBS and the cells were sham or UVA (5 J/cm^2) irradiated using the BS-02 irradiation chamber (Opsytec Dr. Gröbel, Ettlingen Germany). Media was added to the cells and after 4 h incubation, caspase 3 activity was measured as described previously.[33] Protein quantification was done with the Bio-Rad DC protein assay kit (Bio-Rad Laboratories, Hercules, CA) according to the manufacturer protocol.

Detailed Results

We compared the phototoxic potential of five of our test compounds (**8**, **10**, **5b**, **5h**, **4b**) as well as the FDA-approved AhR activator tapinarof with the UVA phototoxicity of FICZ. In contrast to FICZ, neither tapinarof nor our 6-ICZ compounds enhanced UVA radiation-induced caspase activity at a concentration of 100 nM (Figure S36A). At a 10-fold higher concentration (1 µM) two of the test compounds (**8** and **5b**) exhibited similar effects to FICZ, whereas compounds **10** and **4b** did not show any signs of phototoxicity, similarly to tapinarof (Figure S36B).

Figure S36. Phototoxicity levels of tested compounds **FICZ**, tapinarof (**T**), **8**, **10**, **5b**, **5h**, **4b** at 100 nM (**A**) or 1 µM (**B**). n = 6 – 11. For statistical analysis a two-way ANOVA (Tukey post-hoc) was performed and data are shown as mean ± SEM. (*, p ≤ 0.05).

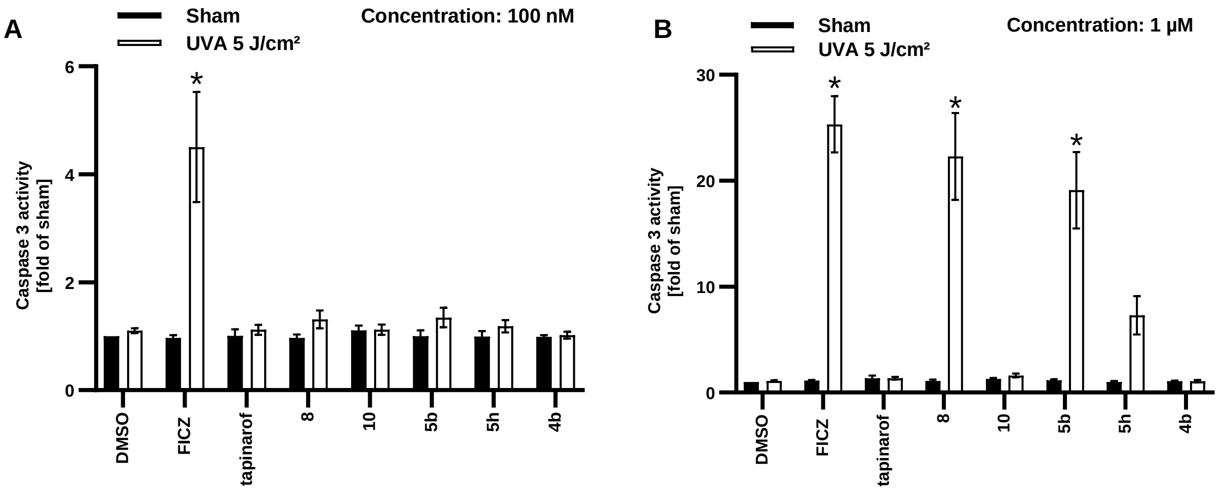

8. X-Ray Crystallography

Crystallographic data for **5b** and **11** were collected on a Bruker APEX-II CCD diffractometer at 296(2) K (**5b**) or 295(2) K (**11**), using a graphite monochromated Mo Kα radiation (λ = 0.71073 Å). Data reduction was performed using SAINT V6.45A and SORTAV in the diffractometer package.[34] Data were corrected for Lorentz and polarization effects and for absorption by SADABS.[35] The structural resolution procedure was made using SHELXT.[36] Non-hydrogen atoms were refined using anisotropic thermal parameters. Hydrogen atoms were introduced in calculated positions and refined riding on their parent atoms.

8.1. Compound 5b

Table S4. Crystal data and refinement parameters of compound **5b**. The crystal structure of compound **5b** contains a disordered dichloromethane molecule with unequal occupancies, 80.1(7):19.9(7).

**CCCD Number**	**2360557**
Chemical formula	$C_{27}H_{18}BrCl_2N_3$
M_r	535.25
Crystal system	Monoclinic, $P2_1$
Space group	P 21/c
Temperature	296(2) K
Wavelength	0.71073 Å
Unit cell dimensions	a = 14.867(12) Å α= 90°.<br>b = 5.908(5) Å β= 101.933(18)°.<br>c = 26.45(2) Å γ = 90°.
Volume	2273(3) Å^3
Z	4
Density (calculated)	1.564 Mg/m^3
Absorption coefficient	2.065 mm^{-1}
F(000)	1080
Crystal size	0.360 x 0.110 x 0.050 mm^3
Theta range for data collection	1.574 to 30.036°
Index ranges	-20<=h<=20, -7<=k<=7, -34<=l<=35
Reflections collected	35136
Independent reflections	5864
Completeness to theta = 25.242°	99.3%
Refinement method	Full-matrix least-squares on F^2
Data / restraints / parameters	5864 / 2 / 306
Goodness-of-fit on F^2	0.824
Final R indices [I>2sigma(I)]	R1 = 0.0712, wR2 = 0.0933
R indices (all data)	R1 = 0.3390, wR2 = 0.1144
Largest diff. peak and hole	0.324 and -0.461 e.Å^{-3}

Figure S36. A) Structure of compound **5b**. B) ORTEP view of compound **5b**, including the disordered dichloromethane molecule. C) Top view of compound **5b** and the dihedral (φ) and butterfly (α) angles. D) Packing arrangement of compound **5b**. The dichloromethane molecule was omitted for clarity.

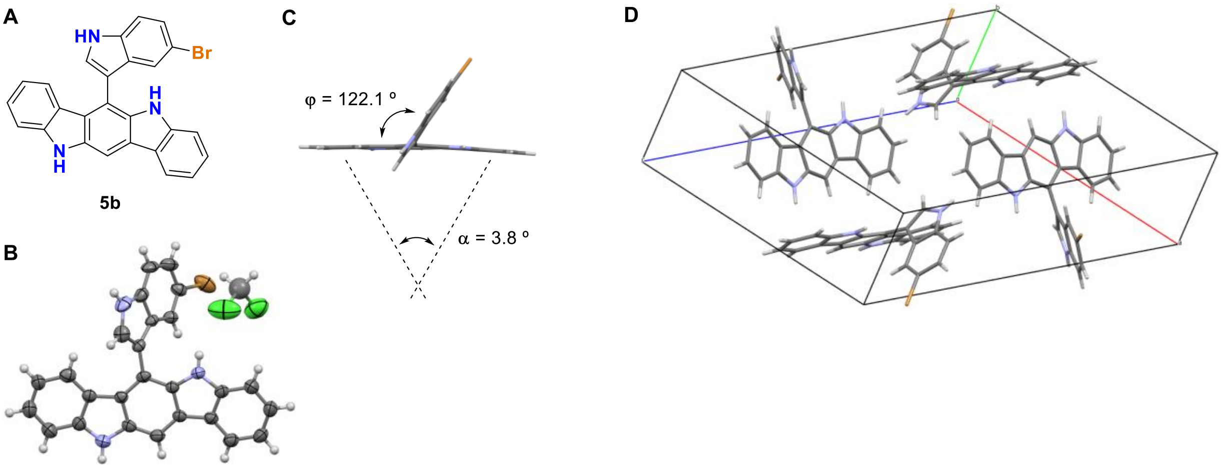

8.2. Compound 11

Table S5. Crystal data and refinement parameters of compound **11**. The Crystal structure of compound **11** exhibits three tetrahydrofuran molecules per asymmetric unit.

**CCCD Number**	**2360558**
Empirical formula	$C_{39}H_{41}N_3O_6$
Formula weight	647.75
Crystal system	Triclinic
Space group	P -1
Temperature	295(2) K
Wavelength	0.71073 Å
Unit cell dimensions	a = 8.785(3) Å α= 83.538(8)°.<br>b = 12.264(4) Å β= 87.724(9)°.<br>c = 16.237(6) Å γ = 84.290(8)°.
Volume	1728.9(10) $Å^3$
Z	2
Density (calculated)	1.244 Mg/m^3
Absorption coefficient	0.084 mm^{-1}
F(000)	688
Crystal size	0.290 x 0.120 x 0.100 mm^3
Theta range for data collection	1.679 to 28.494°
Index ranges	-11<=h<=11, -16<=k<=16, -21<=l<=21
Reflections collected	51531
Independent reflections	8718
Completeness to theta = 25.242°	100.0 %
Refinement method	Full-matrix least-squares on F^2
Data / restraints / parameters	8718 / 0 / 434
Goodness-of-fit on F^2	0.919
Final R indices [I>2sigma(I)]	R1 = 0.0889, wR2 = 0.2163
R indices (all data)	R1 = 0.2228, wR2 = 0.2633
Largest diff. peak and hole	0.312 and -0.215 e.$Å^{-3}$

Figure S37. A) Structure of compound **11**. B) ORTEP view of compound **11**, including the three tetrahydrofuran molecules. D) Packing arrangement of compound **11**. The tetrahydrofuran molecules and the hydrogen atoms were omitted for clarity.

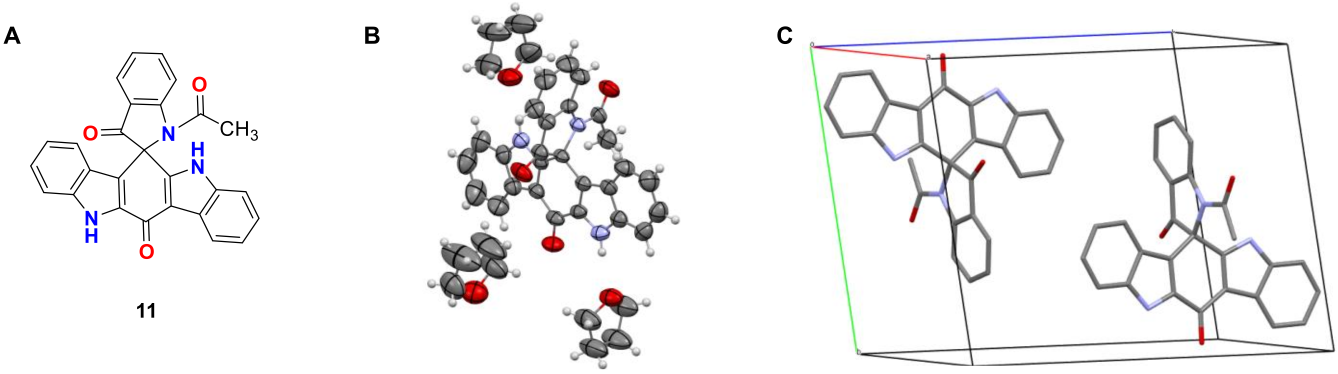

9. References

[1] Brahmachari, G., & Das S. *L*-Proline catalyzed multicomponent one-pot synthesis of gem-diheteroarylmethane derivatives using facile grinding operation under solvent-free conditions at room temperature. *RSC Adv.* **4**, 7380–7388 (2014).

[2] Renzetti, A. et al. Yonemitsu-type condensations catalysed by proline and Eu(OTf)$_3$. *RSC Adv*. **4**, 47992–47999 (2014).

[3] Criado, A., Vilas-Varela, M., Cobas, A., Pérez, D., Peña, D. & Guitián, E. Acid-promoted aromatization of perylene-based endoxides. Heterocycles, **88**, 1625–1632 (2014).

[4] Álvarez, B. et al. Aryne-based synthesis of cyclobutadiene-containing oligoacenes and related extended biphenylene derivatives. *Adv. Synth. Catal.* **366**, 961–969 (2024).

[5] SPARTAN, Wavefunction, Inc., 18401, Von Karman Avenue, Suite 435, Irvine CA, 92612, USA.

[6] Zaghdane, H. et al. New indole amide derivatives as potent CRTH2 receptor antagonists. *Bioorg. Med. Chem. Lett*. **21**, 3471–3474 (2011).

[7] Rajasekar, S. & Anbarasan, P. Tandem Rh (ii) and chiral squaramide relay catalysis: enantioselective synthesis of dihydro-β-carbolines via insertion to C–H bond and aza-michael reaction. *Org. Lett*. **21**, 3067–3071 (2019).

[8] Staroń, J. et al. Rationally designed N-phenylsulfonylindoles as a tool for the analysis of the non-basic 5-HT6R ligands binding mode. *Eur. J. Med. Chem*. **209**, 112916 (2021).

[9] Sun, X. et al. 1-Indanone and 1,3-indandione derivatives as ligands for misfolded α-synuclein aggregates. *ChemMedChem* **17**, e202100611 (2021).

[10] Riyaz, Sd., Indrasena, A., Naidu, A. & Dubey, P. Novel, recyclable, and thermally stable task-specific ionic liquid (TBA acetate) medium/catalyst for the synthesis of indolylidinecyclic-1,3- and -1,4-diketones. *Synth. Commun*. **244**, 368–373 (2013).

[11] Magar, R. L. et al. Synthesis of some novel 3-substituted indole derivatives using polyamine functionalized heterogeneous catalyst. *J. Heterocycl. Chem*. **52**, 1684–1692 (2015).

[12] Singh, P., Kaur, M. & Verma, P. Design, synthesis and anticancer activities of hybrids of indole and barbituric acids—Identification of highly promising leads. *Bioorg. Med. Chem. Lett.* **19**, 3054–3058 (2009).

[13] Lv, Y., Guo, Y., Xu, J. & Shao, S. Simple indole-based colorimetric sensors with electron-withdrawing chromophores: Tuning selectivity in anion sensing. *J. Fluor. Chem.* **132**, 973–977 (2011).

[14] Zhao, Y. & Truhlar, D. G. The M06 suite of density functionals for main group thermochemistry, thermochemical kinetics, noncovalent interactions, excited states, and transition elements: two new functionals and systematic testing of four M06-class functionals and 12 other functionals. *Theor. Chem. Acc*. **120**, 215–241 (2008).

[15] Hehre, W. J., Ditchfield, R. & Pople, J. A. Self—consistent molecular orbital methods. XII. Further extensions of gaussian—type basis sets for use in molecular orbital studies of organic molecules. *J. Chem. Phys.* **56**, 2257–2261 (1972).

[16] Hariharan, P. C. & Pople, J. A. The influence of polarization functions on molecular orbital hydrogenation energies. *Theor. Chim. Acta*. **28**, 213–222 (1973).

[17] Frisch, M. J. et al. Gaussian, Inc., Wallingford CT (2016).

[18] Head-Gordon, M., Pople, J. A. & Frisch, M. J. MP2 energy evaluation by direct methods. *Chem. Phys. Lett.* **153**, 503–506 (1988).

[19] Marenich, A. V., Cramer, C. J. & Truhlar, D. G. Universal solvation model based on solute electron density and on a continuum model of the solvent defined by the bulk dielectric constant and atomic surface tensions. *J. Phys. Chem. B* **113**, 6378–6396 (2009).

[20] Soteras, I., Curutchet, C., Bidon-Chanal, A., Orozco, M. & Luque, F. J. Extension of the MST model to the IEF formalism: HF and B3LYP parametrizations. *J. Mol. Struct. (THEOCHEM)* **727**, 29–40 (2005).

[21] Schrödinger Release 2021-2: Glide, Schrödinger, LLC, New York, NY (2021).

[22] Case, D.A. et al. AMBER, University of California, San Francisco (2020).

[23] Lindorff‐Larsen, K. Et al. Improved side-chain torsion potentials for the Amber ff99SB protein force field. *Proteins* **78**, 1950‐1958 (2010).

[24] Wang, J., Wolf, R. M., Caldwell, J. W., Kollman, P. A. & Case, D. A. Development and testing of a general amber force field. *J. Comput. Chem.* **25**, 1157–1174 (2004).

[25] Xiong, Y., Shabane, P. S. & Onufriev, A. V. Melting points of OPC and OPC3 water models. *ACS Omega* **5**, 25087–25094 (2020).

[26] Ryckaert, J.-P., Ciccotti, G. & Berendsen, H. J. C. Numerical integration of the cartesian equations of motion of a system with constraints: molecular dynamics of *n*-alkanes. *J. Comput. Phys.* **23**, 327–341 (1977).

[27] Darden, T., York, D. L. Pedersen, D. Particle mesh Ewald: An *N*·log(*N*) method for Ewald sums in large systems. *J. Chem. Phys.* **98**, 10089–10092 (1993).

[28] Vogeley, C. et al. Unraveling the differential impact of PAHs and dioxin-like compounds on AKR1C3 reveals the EGFR extracellular domain as a critical determinant of the AHR response. *Environ. Int*. **158**, 106989 (2022).

[29] Fritsche, E. et al. Lightening up the UV response by identification of the arylhydrocarbon receptor as a cytoplasmatic target for ultraviolet B radiation. *Proc. Natl. Acad. Sci.* **104**, 8851–8856 (2007).

[30] Frauenstein, K. et al. Activation of the aryl hydrocarbon receptor by the widely used Src family kinase inhibitor 4-amino-5-(4-chlorophenyl)-7-(dimethylethyl)pyrazolo[3,4-*d*]pyrimidine (PP2). *Arch. Toxicol.* **89**, 1329–1336 (2015).

[31] Wang, Y. et al. Reversed-phase chromatography with multiple fraction concatenation strategy for proteome profiling of human MCF10A cells. *Proteomics* **11**, 2019–2026 (2011).

[32] Ahrné, E., Glatter, T., Viganò, C., von Schubert, C., Nigg, E. A. & Schmidt, A. Evaluation and improvement of quantification accuracy in isobaric mass tag-based protein quantification experiments. *J. Proteome Res.* **15**, 2537–2547 (2016).

[33] Rolfes K. M. et al. Inhibition of 6-formylindolo[3,2-b]carbazole metabolism sensitizes keratinocytes to UVA-induced apoptosis: Implications for vemurafenib-induced phototoxicity. *Redox Biol.* **46**, 102110 (2021).

[34] Blessing, R. H. An empirical correction for absorption anisotropy. *Acta Cryst. A* **51**, 33–38 (1995).

[35] Bruker AXS Inc., SAINT, Madison, Wisconsin, USA.

[36] Sheldrick, G. M. SHELXT – Integrated space-group and crystal-structure determination. *Acta Cryst. A* **71**, 3–8 (2015).

10. Copies of NMR Spectra

10.1. Starting Materials

(1H-Indole-2-yl)methanol (3h)

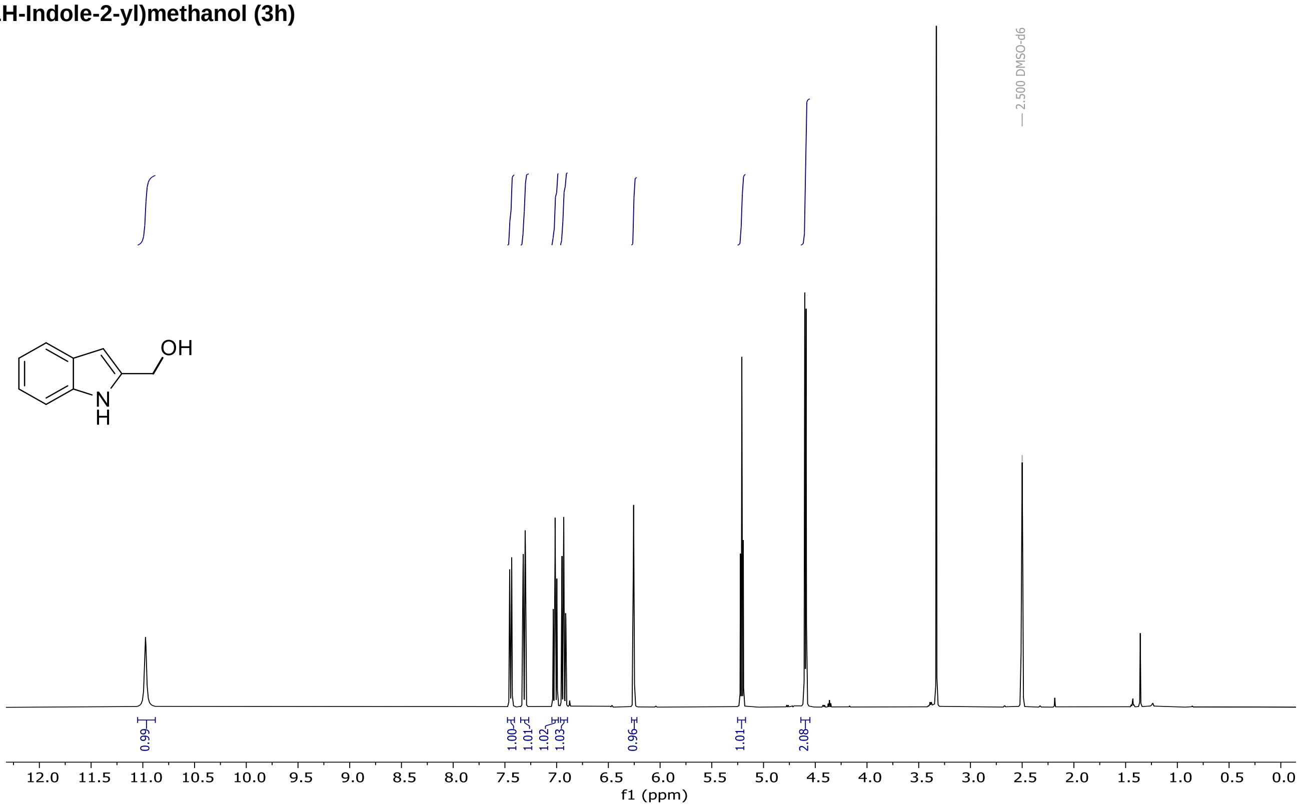

Indole-2-carboxaldehyde (1a)

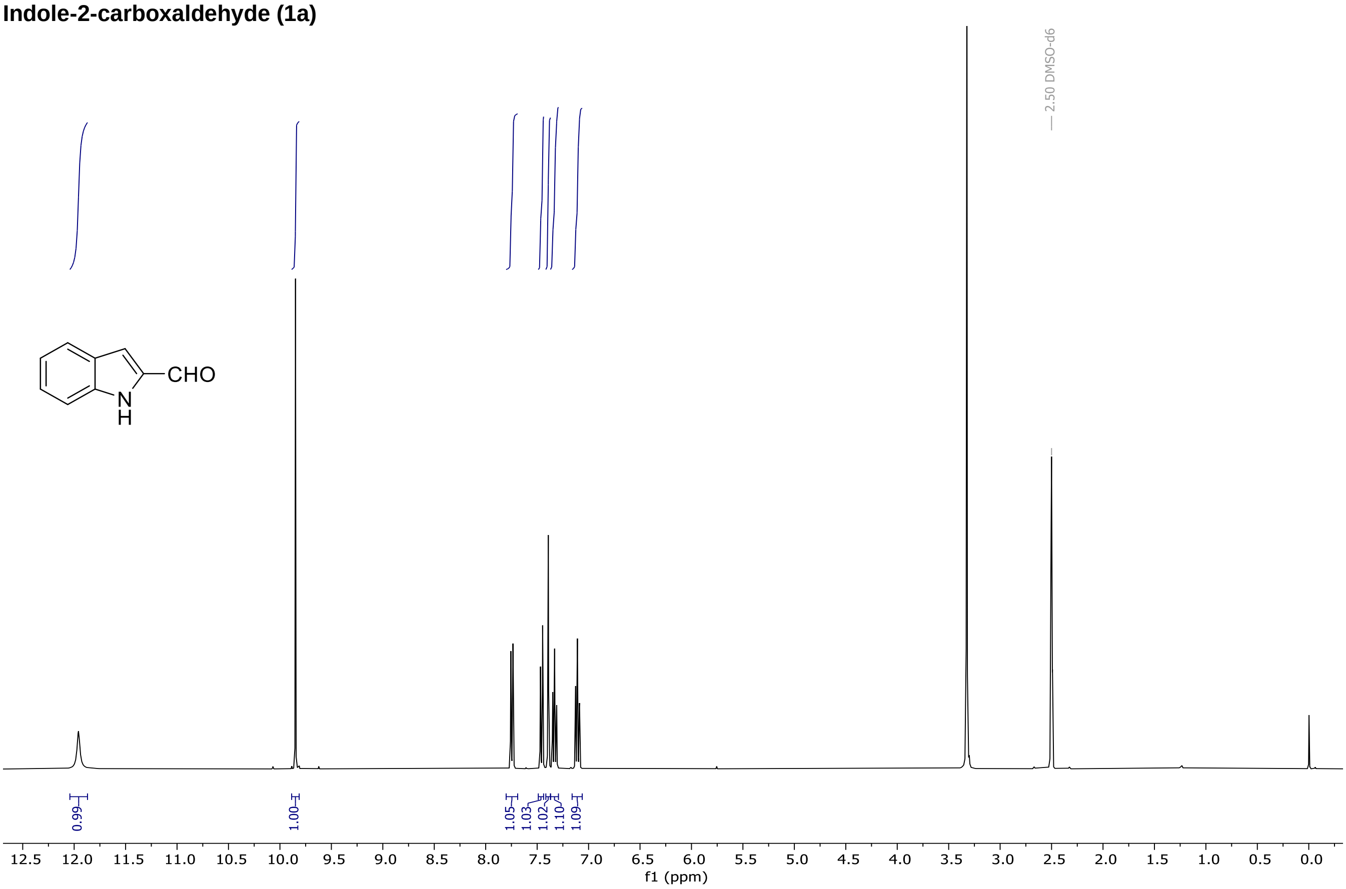

Ethyl 5-fluoro-1*H*-indole-2-carboxylate (S6)

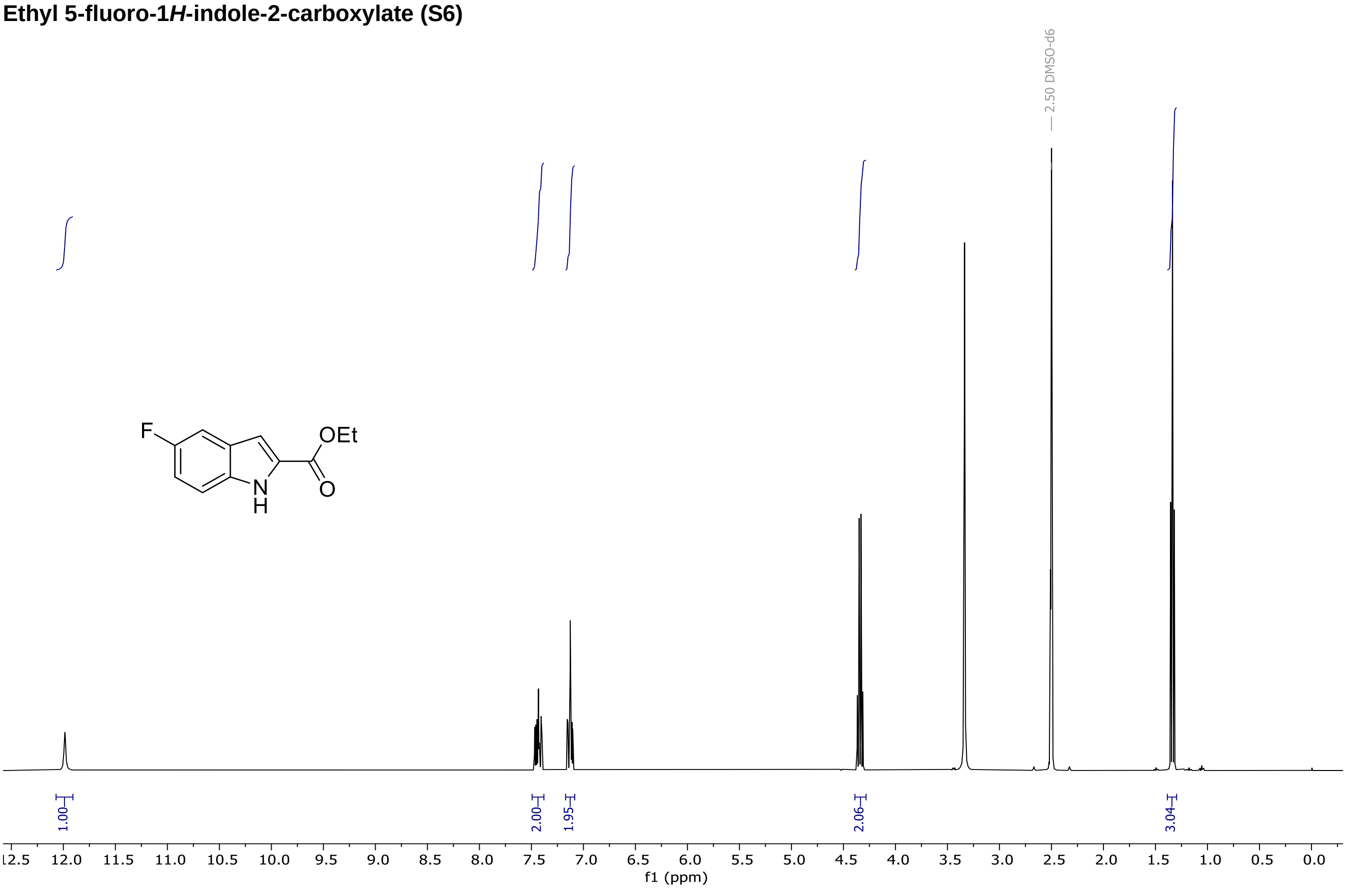

(5-Fluoro-1*H*-indol-2-yl)methanol (S7)

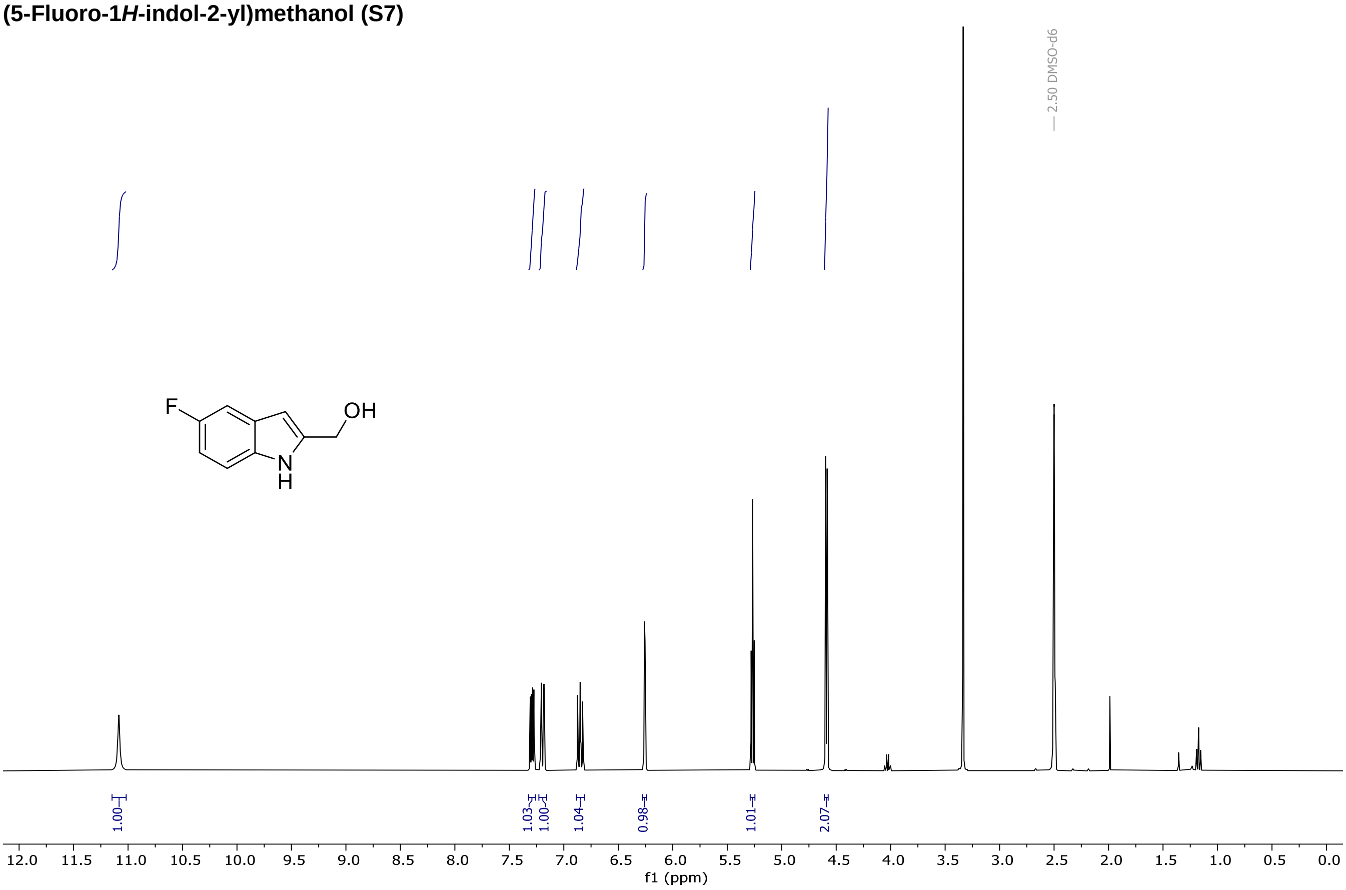

5-Fluoro-1*H*-indole-2-carbaldehyde (1d)

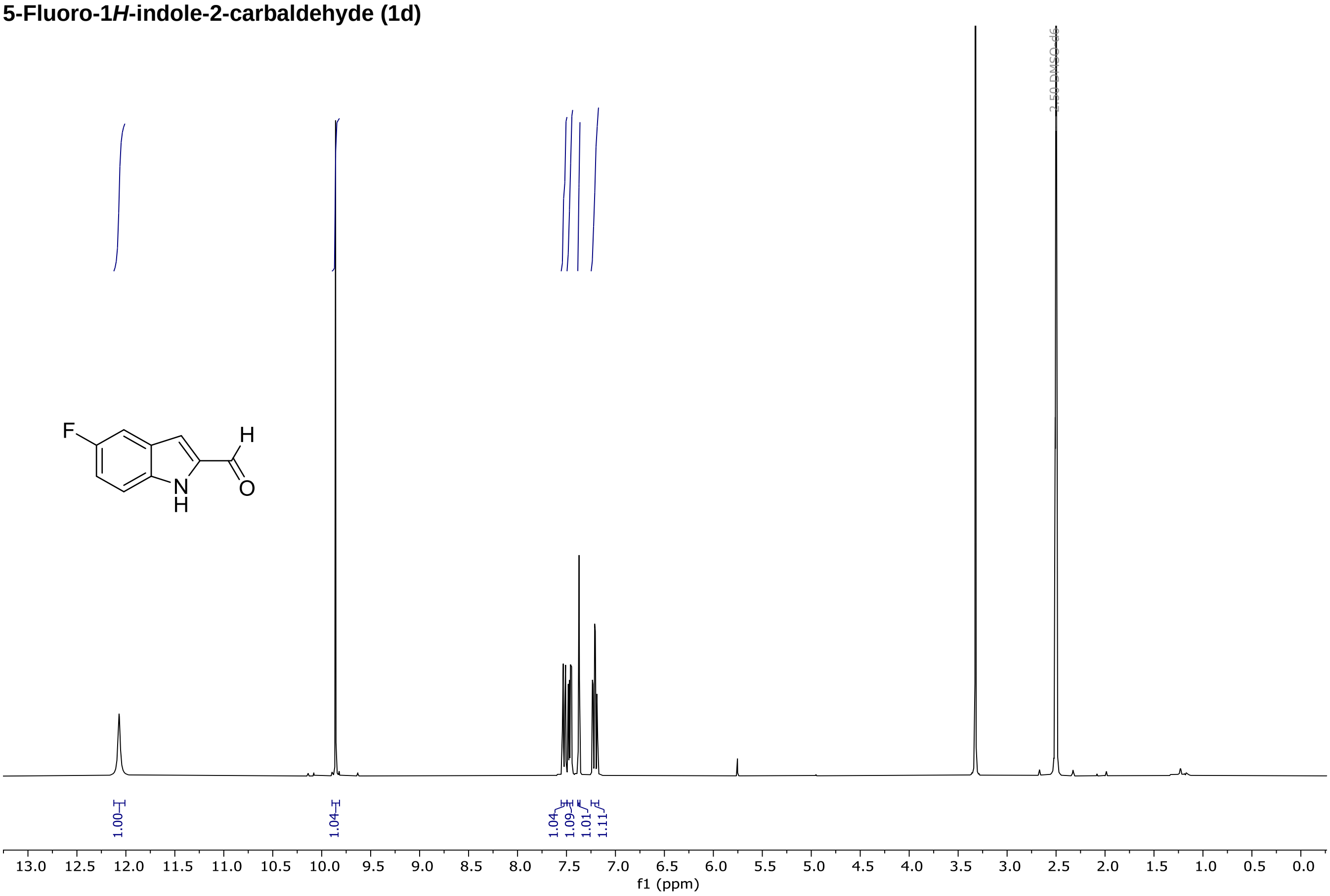

1-Methyl-1*H*-indole (3e)

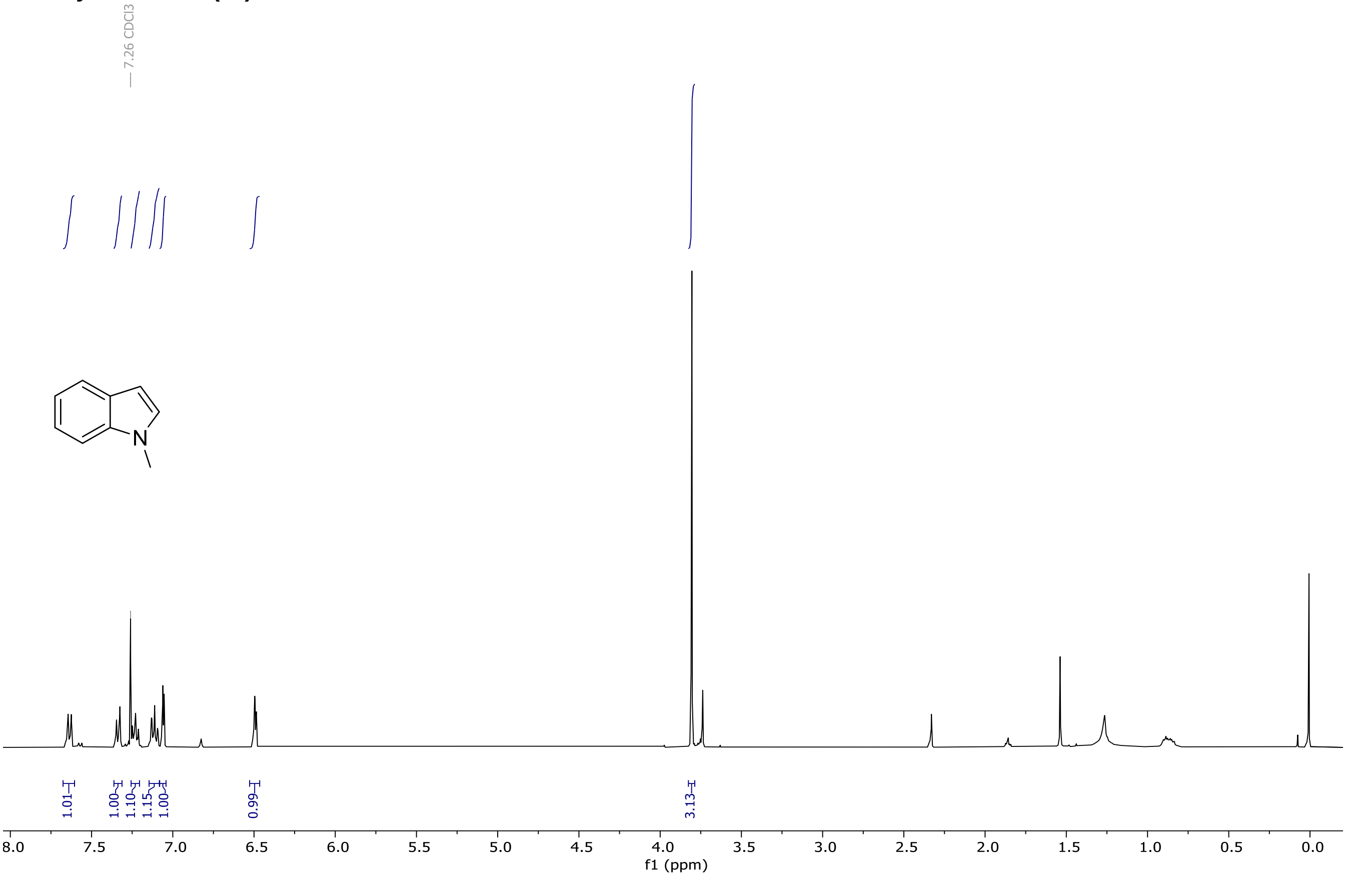

1-Methyl-1*H*-indole-2-carboxaldehyde (1c)

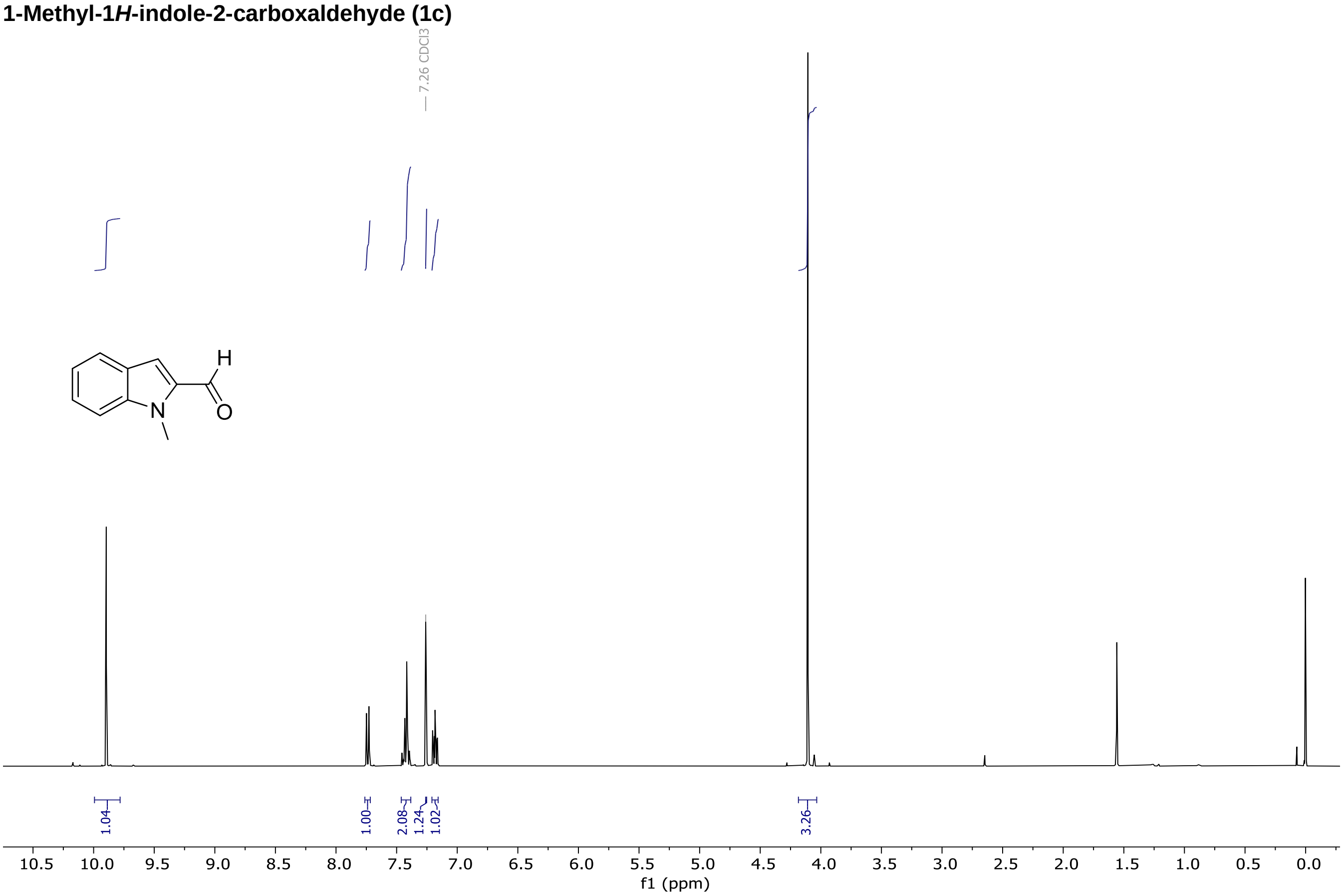

5-Methoxy-1-(phenylsulfonyl)-1*H*-indole (3c)

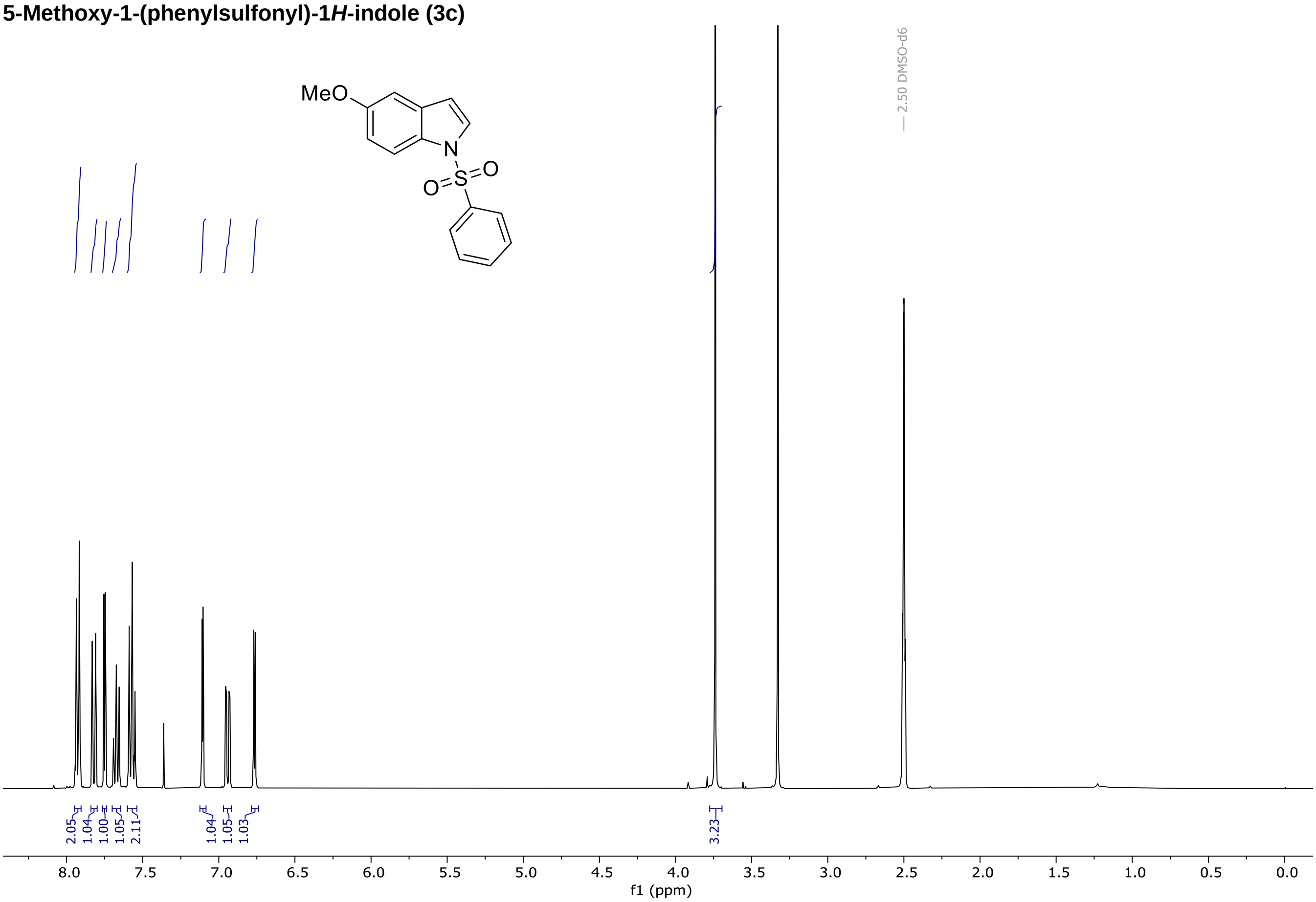

5-Methoxy-1-(phenylsulfonyl)-1*H*-indole-2-carboxaldehyde (1e)

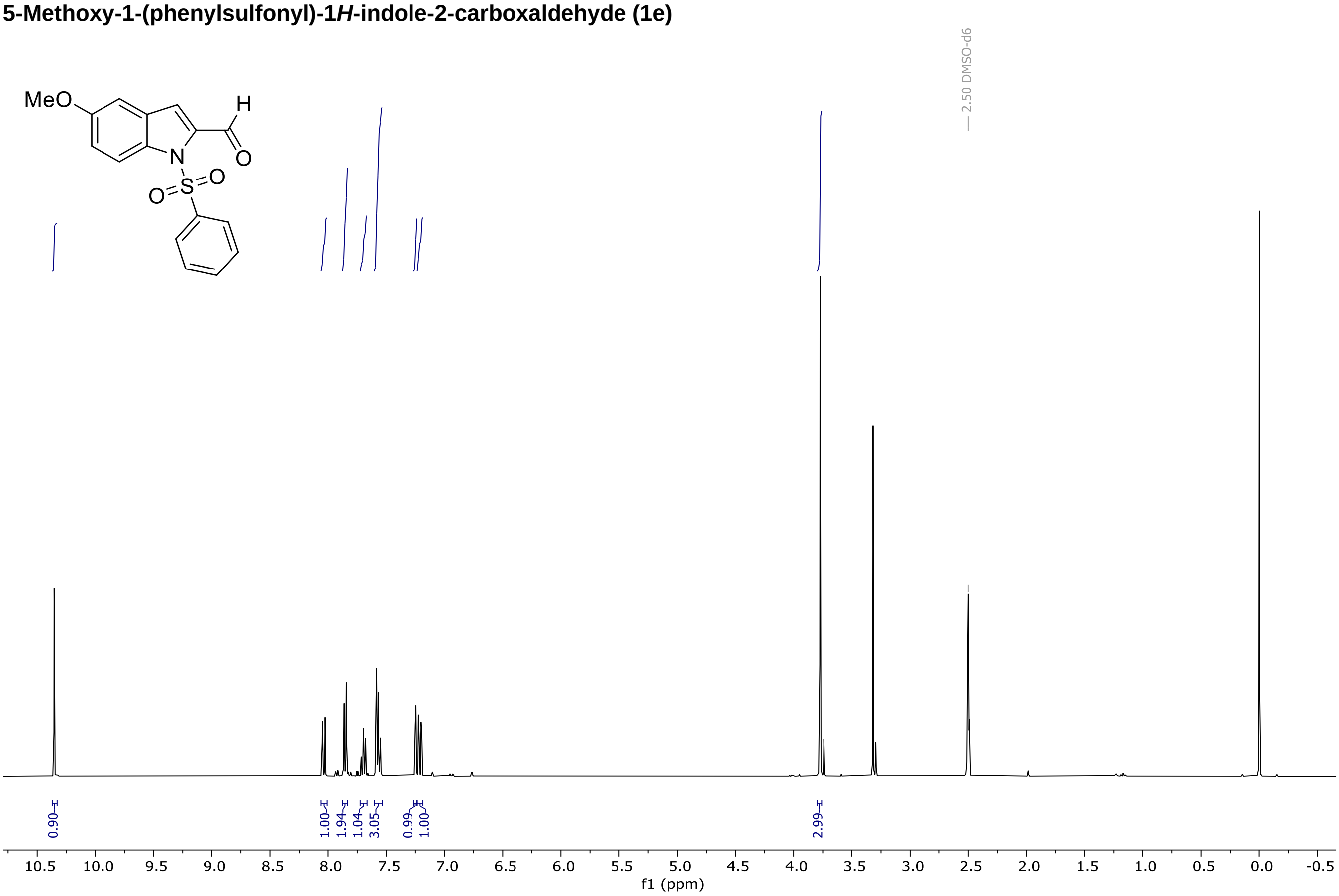

5-Methoxy-1*H*-indole-2-carboxaldehyde (1f)

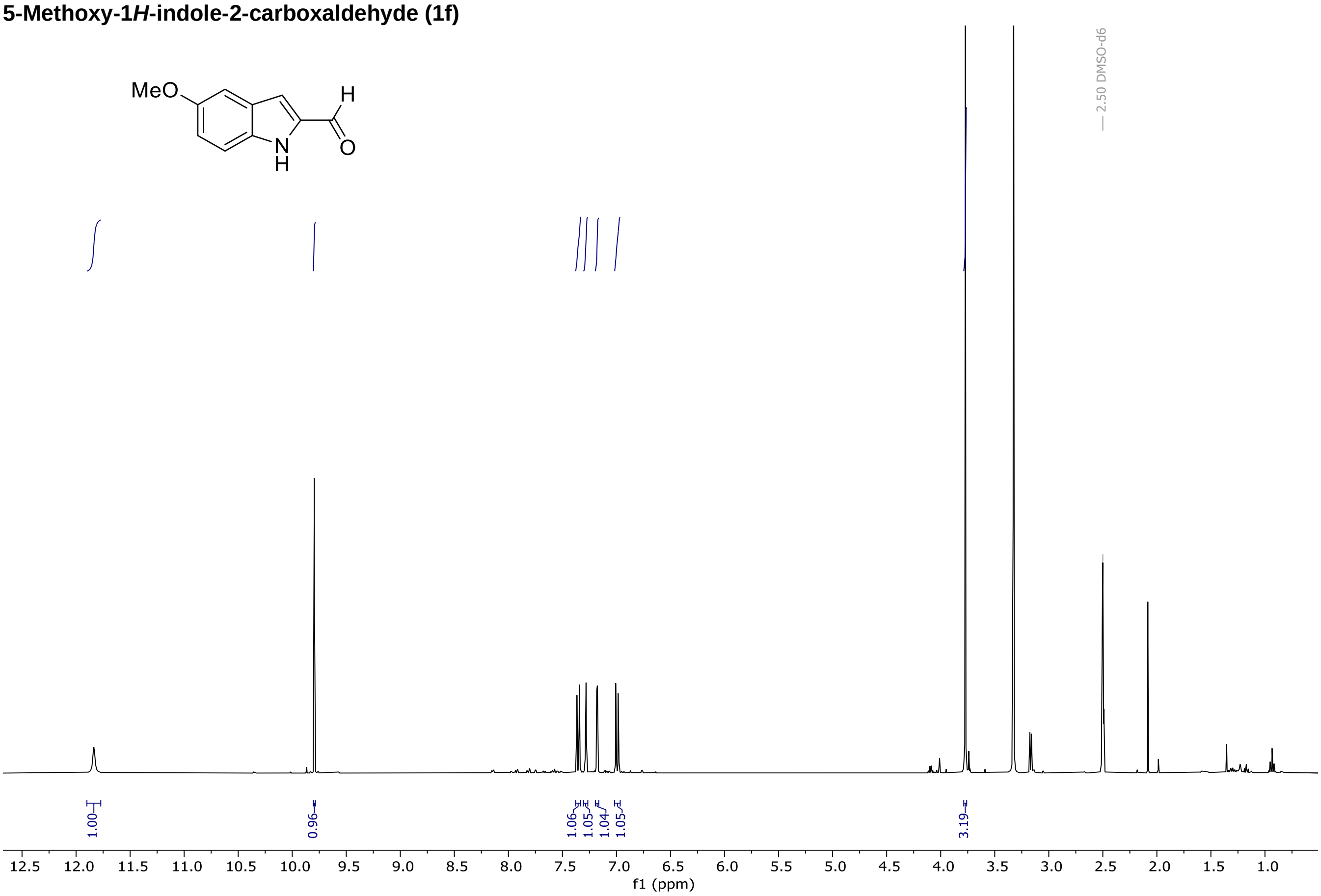

Methyl 1*H*-indole-6-carboxylate (3k)

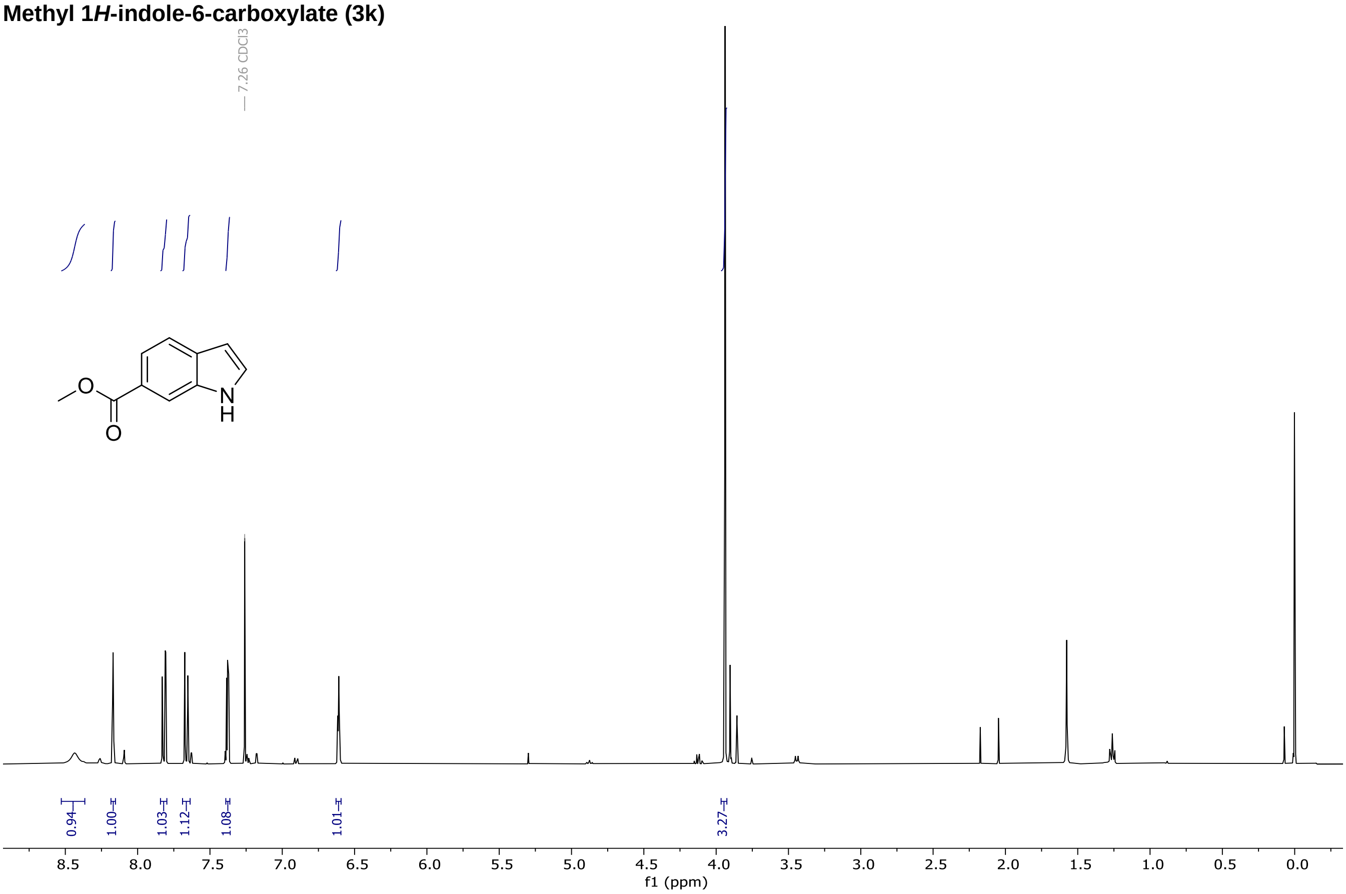

1-(1*H*-Indol-2-yl)pentan-1-ol (Id)

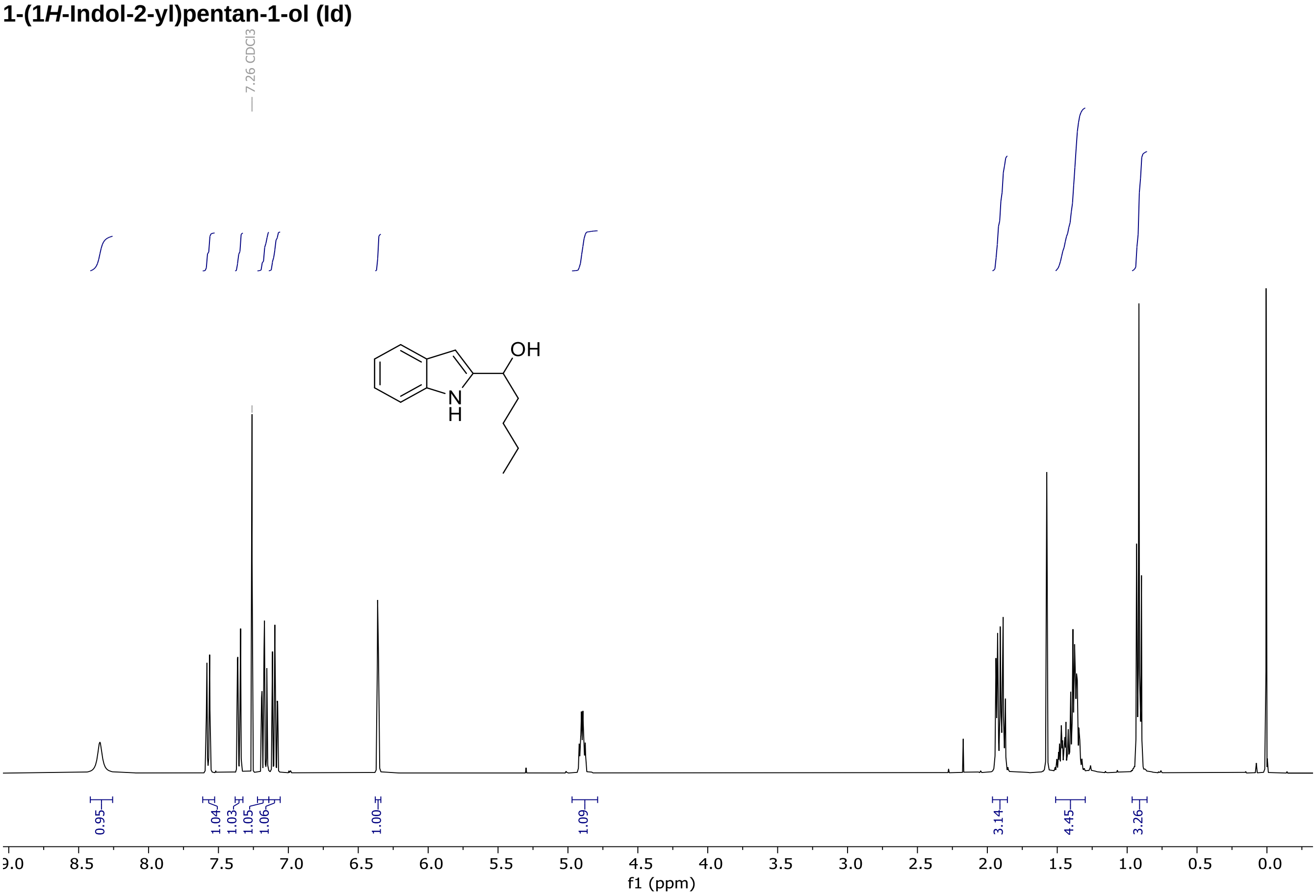

1-(1*H*-Indol-2-yl)but-3-en-1-ol (3i)

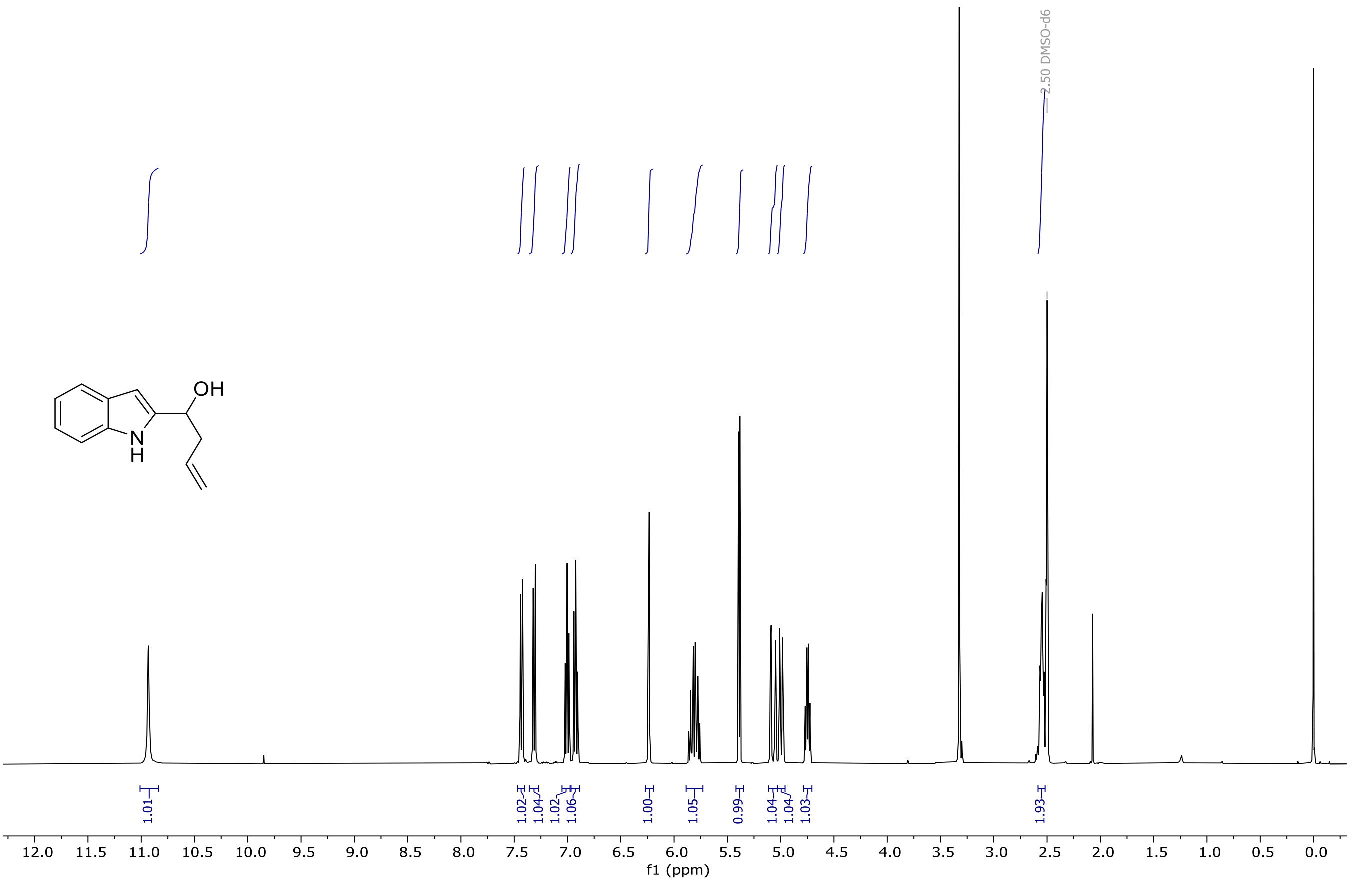

10.2. 6-ICZs (Series 4-5)

2-(5,11-Dihydroindolo[3,2-*b*]carbazol-6-yl)-3-hydroxy-5,5-dimethylcyclohex-2-en-1-one (4a)

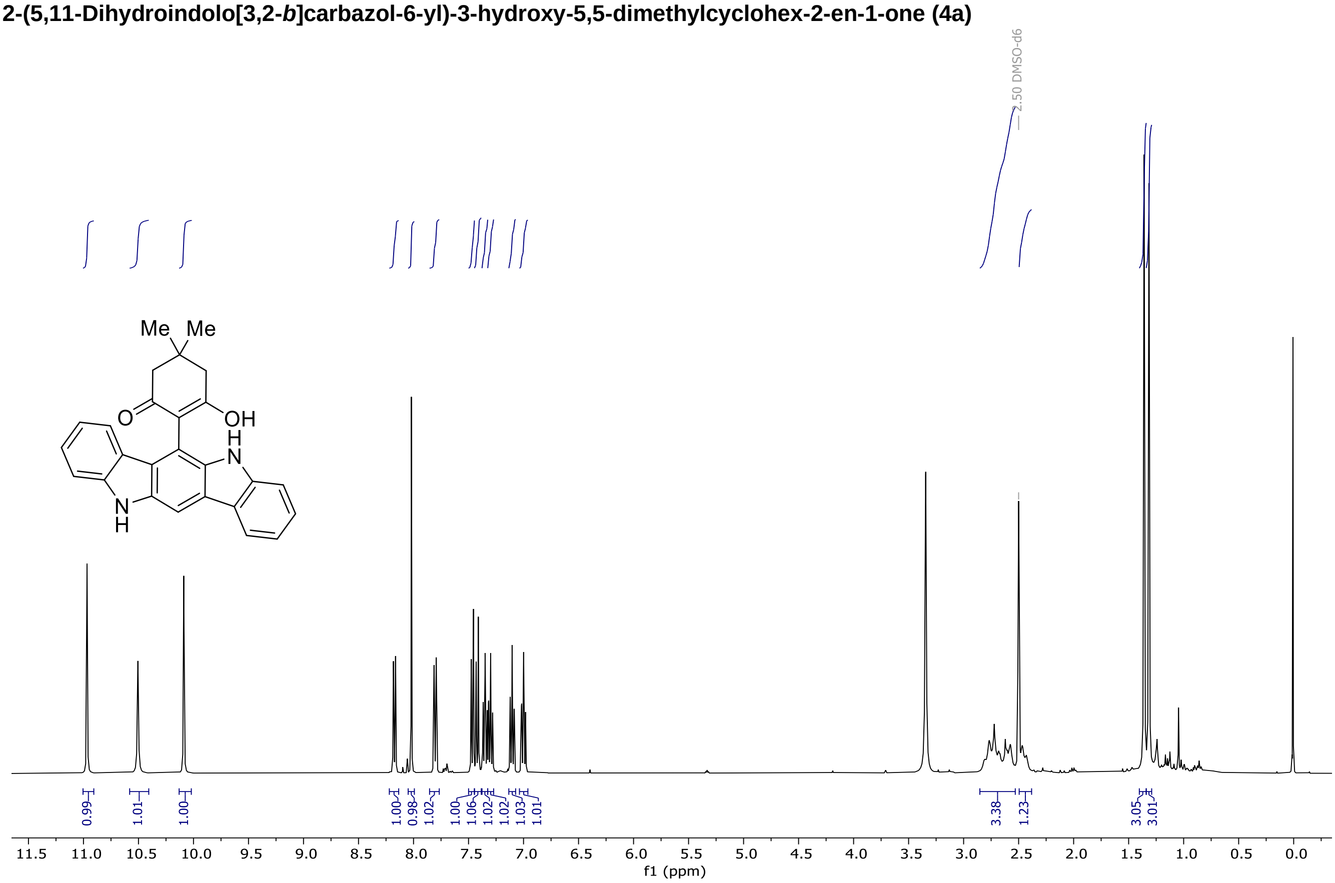

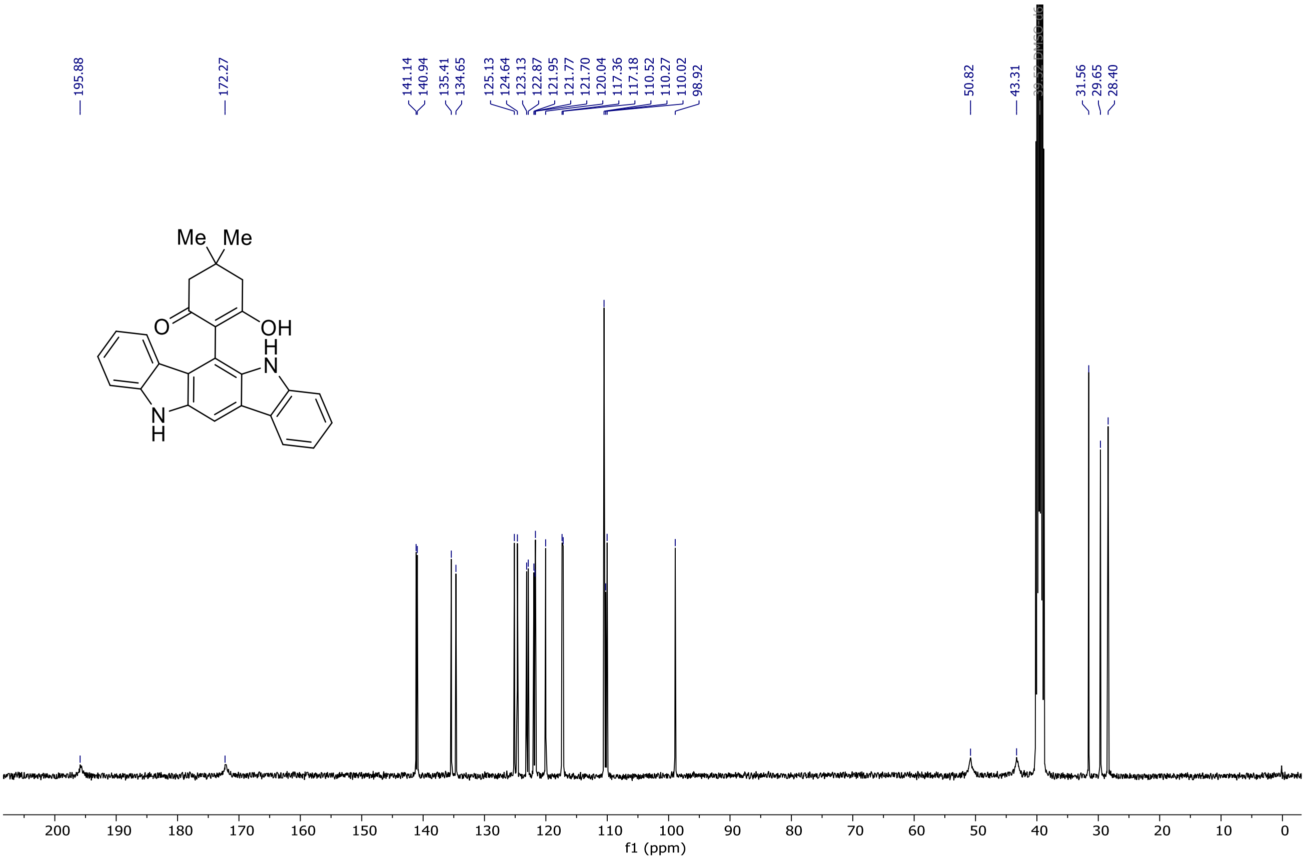

4-(5,11-Dihydroindolo[3,2-*b*]carbazole-6-yl)-5-hydroxyfuran-3(2*H*)-one (4b)

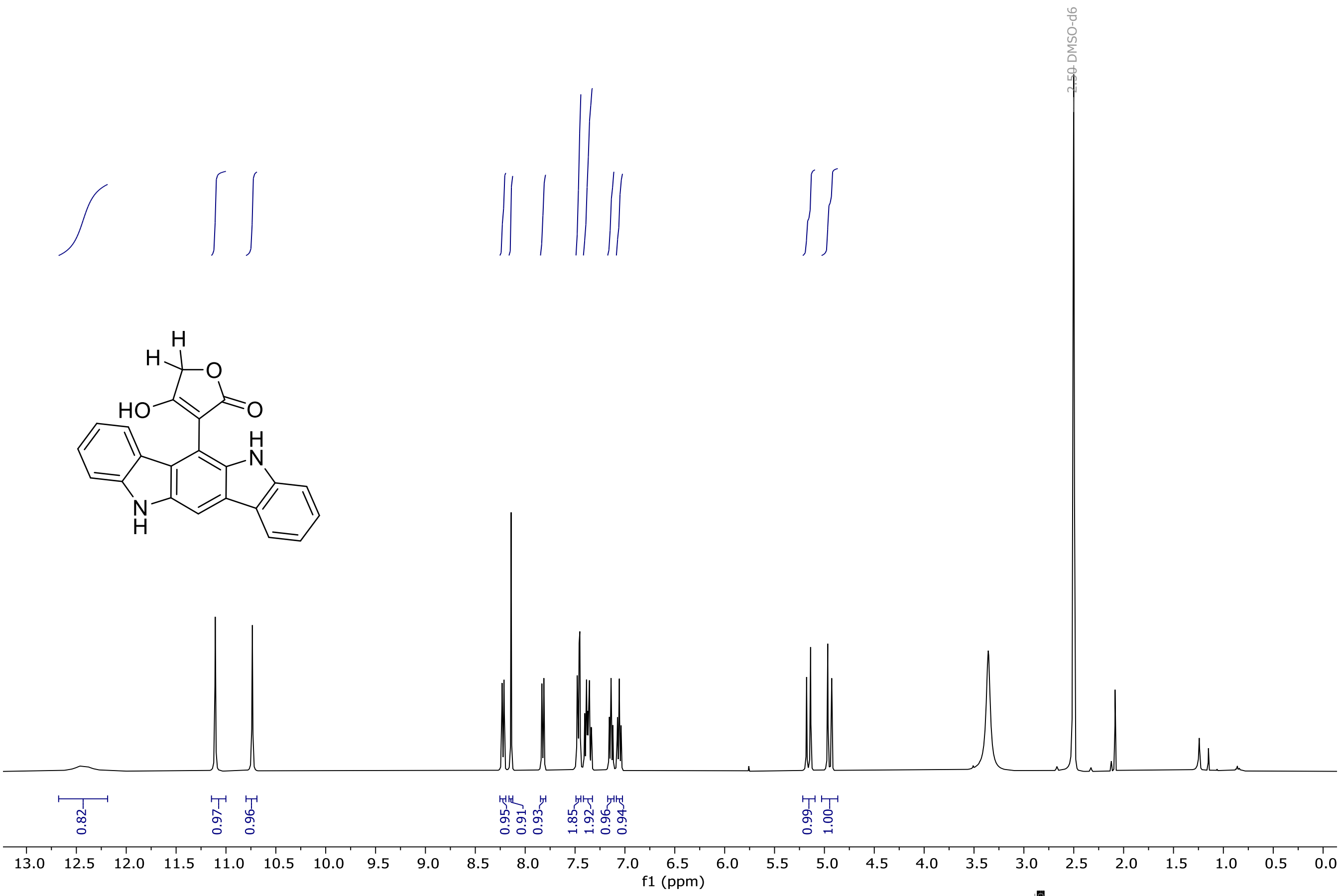

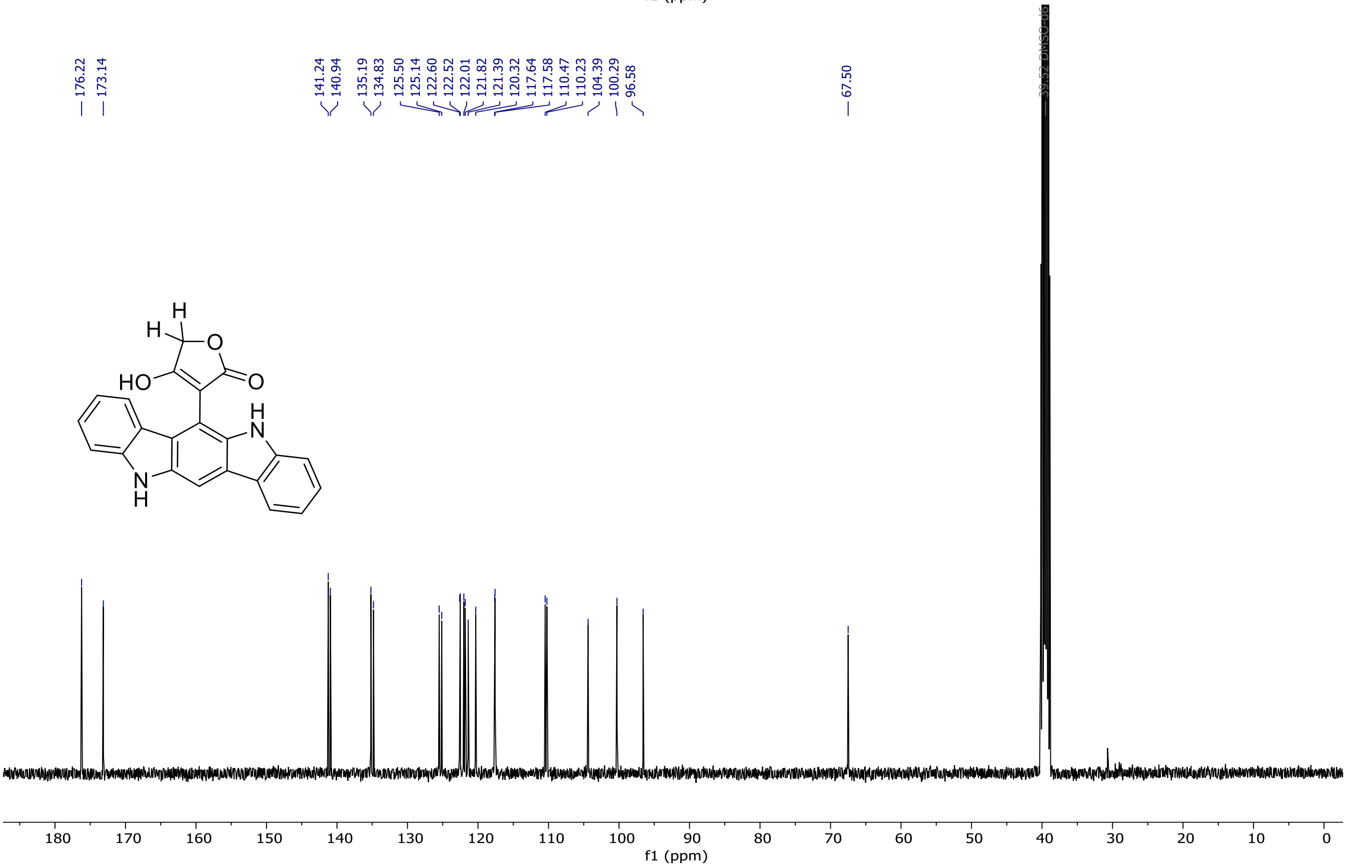

3-(5,11-Dihydroindolo[3,2-*b*]carbazol-6-yl)-4-hydroxy-2*H*-chromen-2-one (4c)

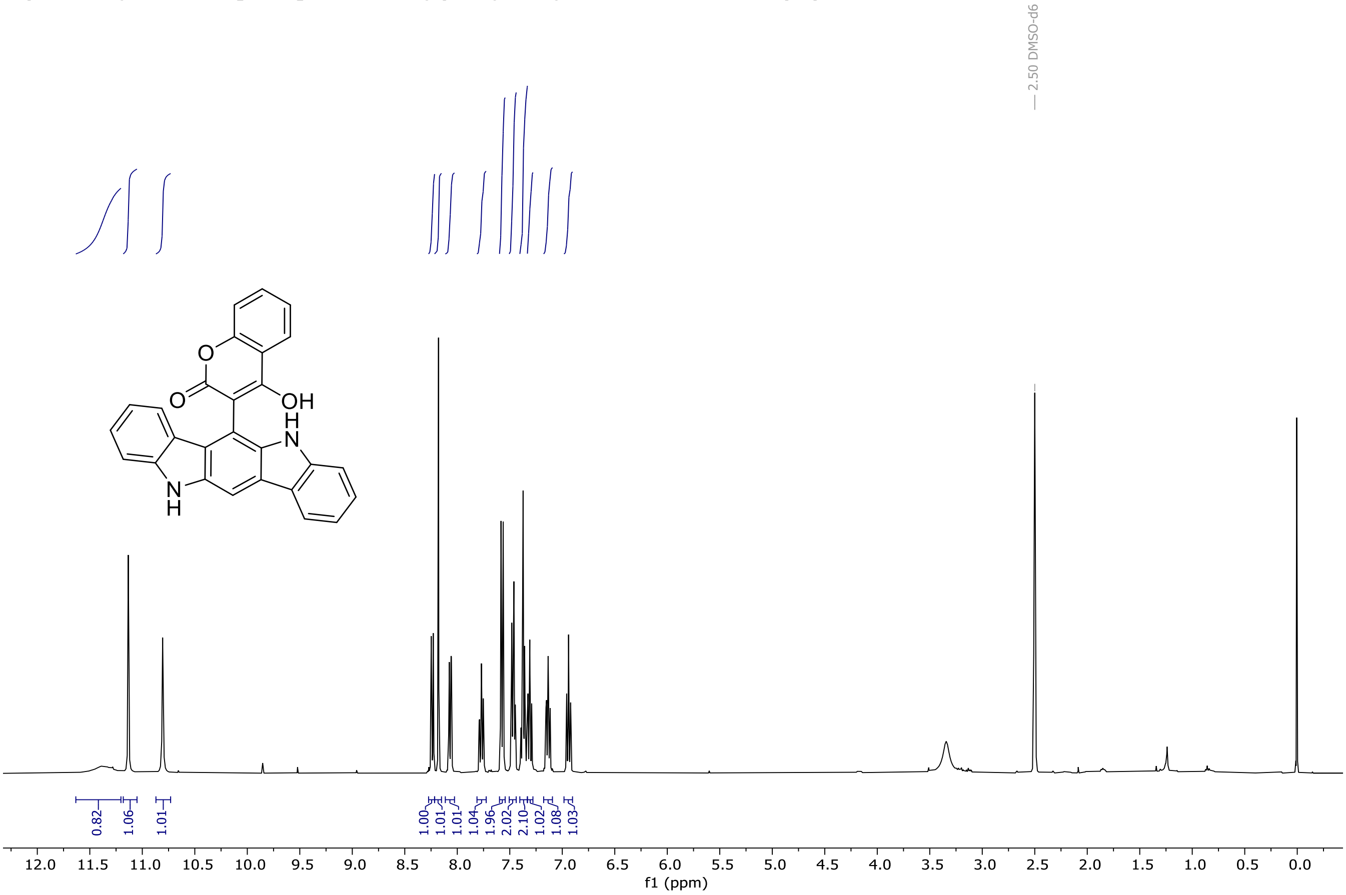

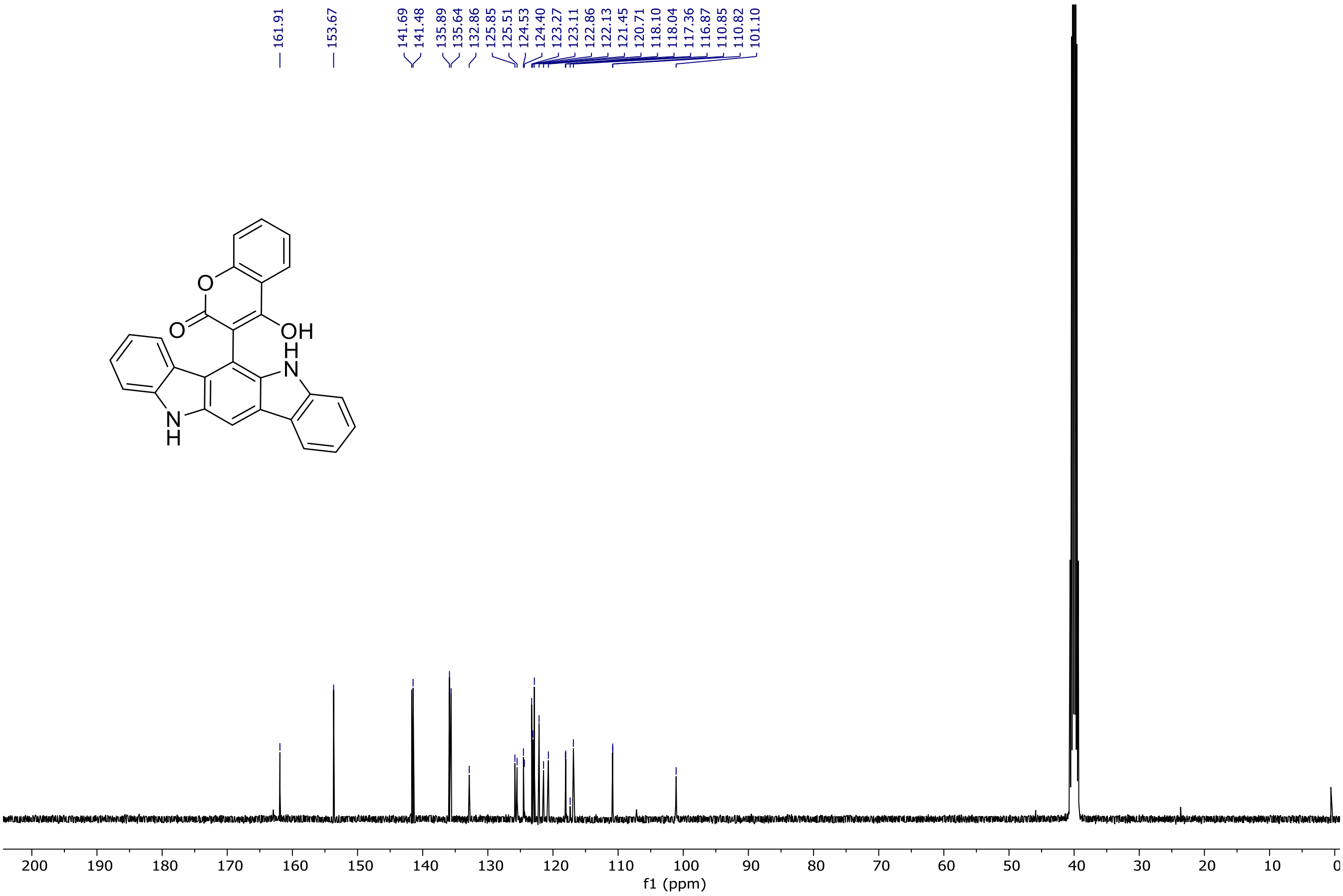

2-(5,11-Dimethyl-5,11-dihydroindolo[3,2-*b*]carbazol-6-yl)-3-hydroxy-5,5-dimethylcyclohex-2-en-1-one (4d)

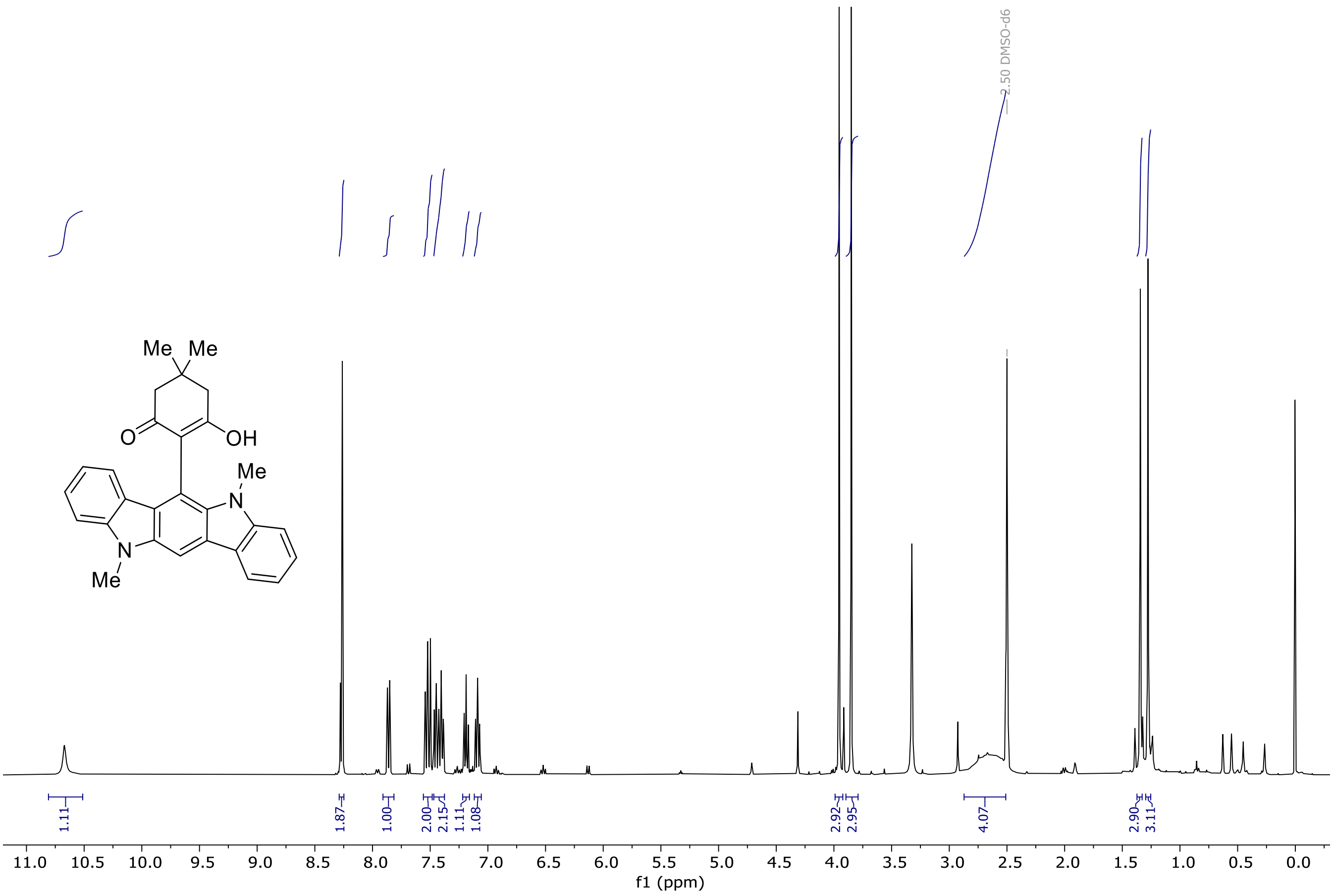

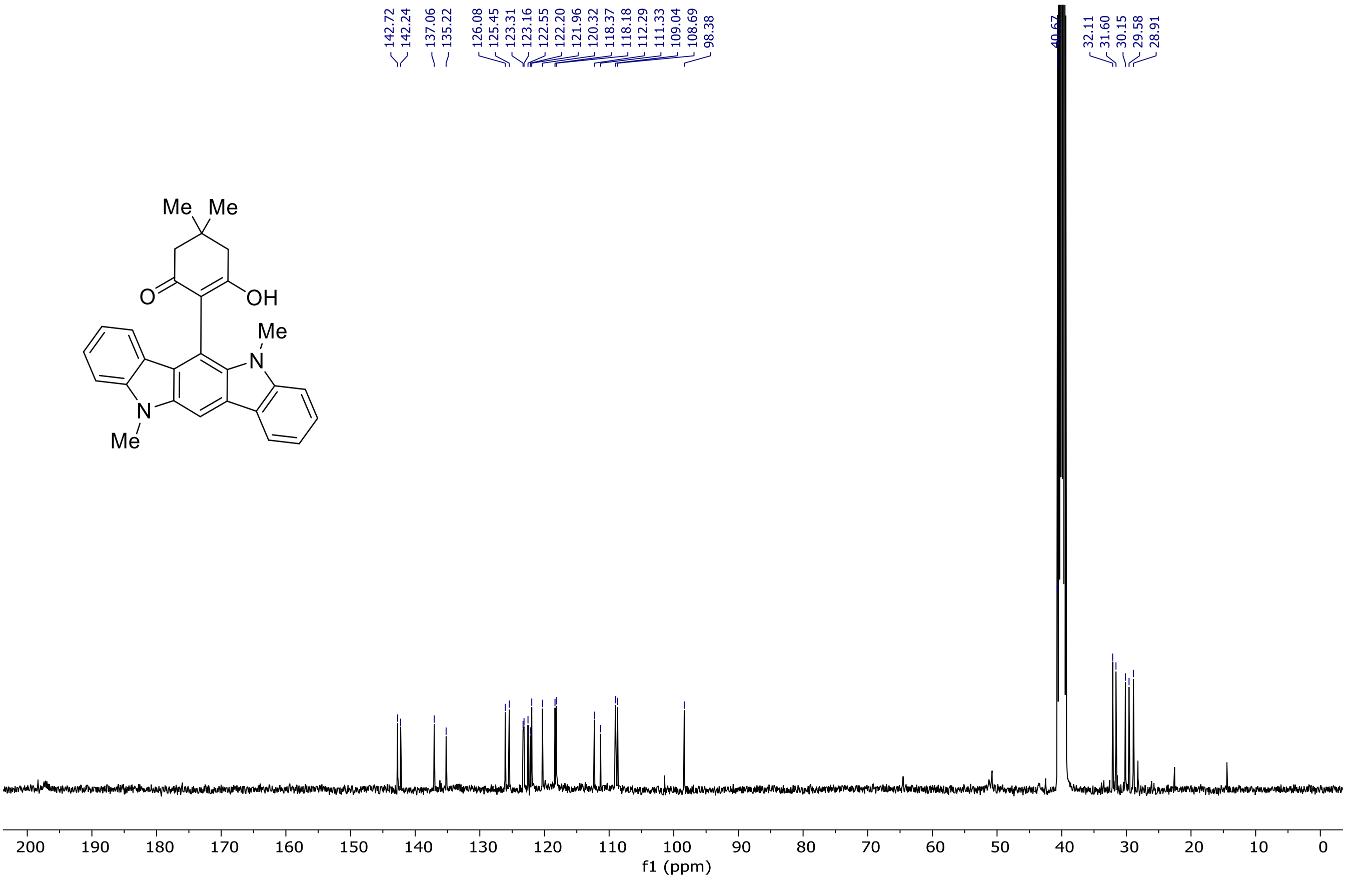

6-(5-Methoxy-1*H*-indol-3-yl)-5,11-dihydroindolo[3,2-*b*]carbazole (5a)

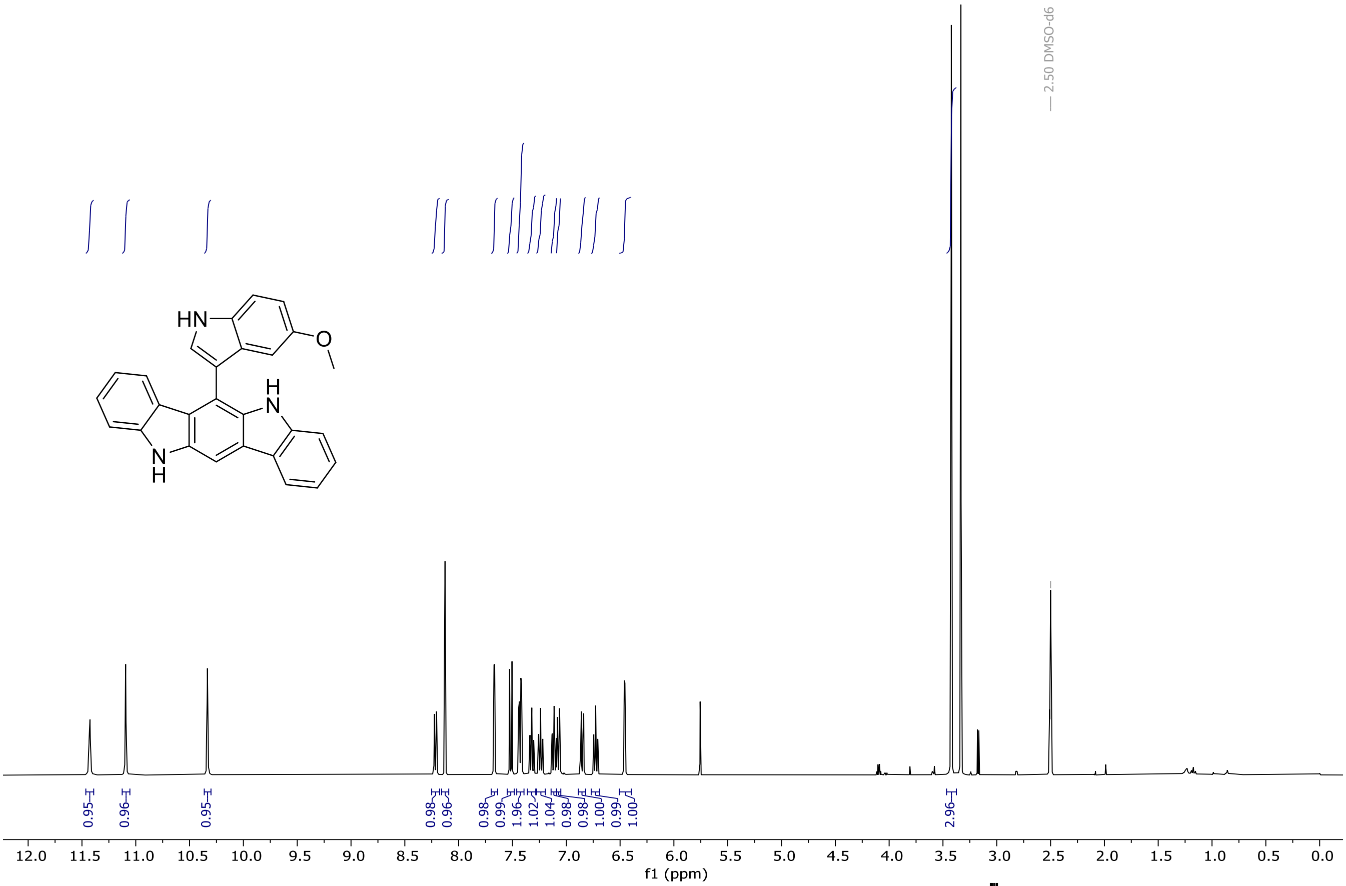

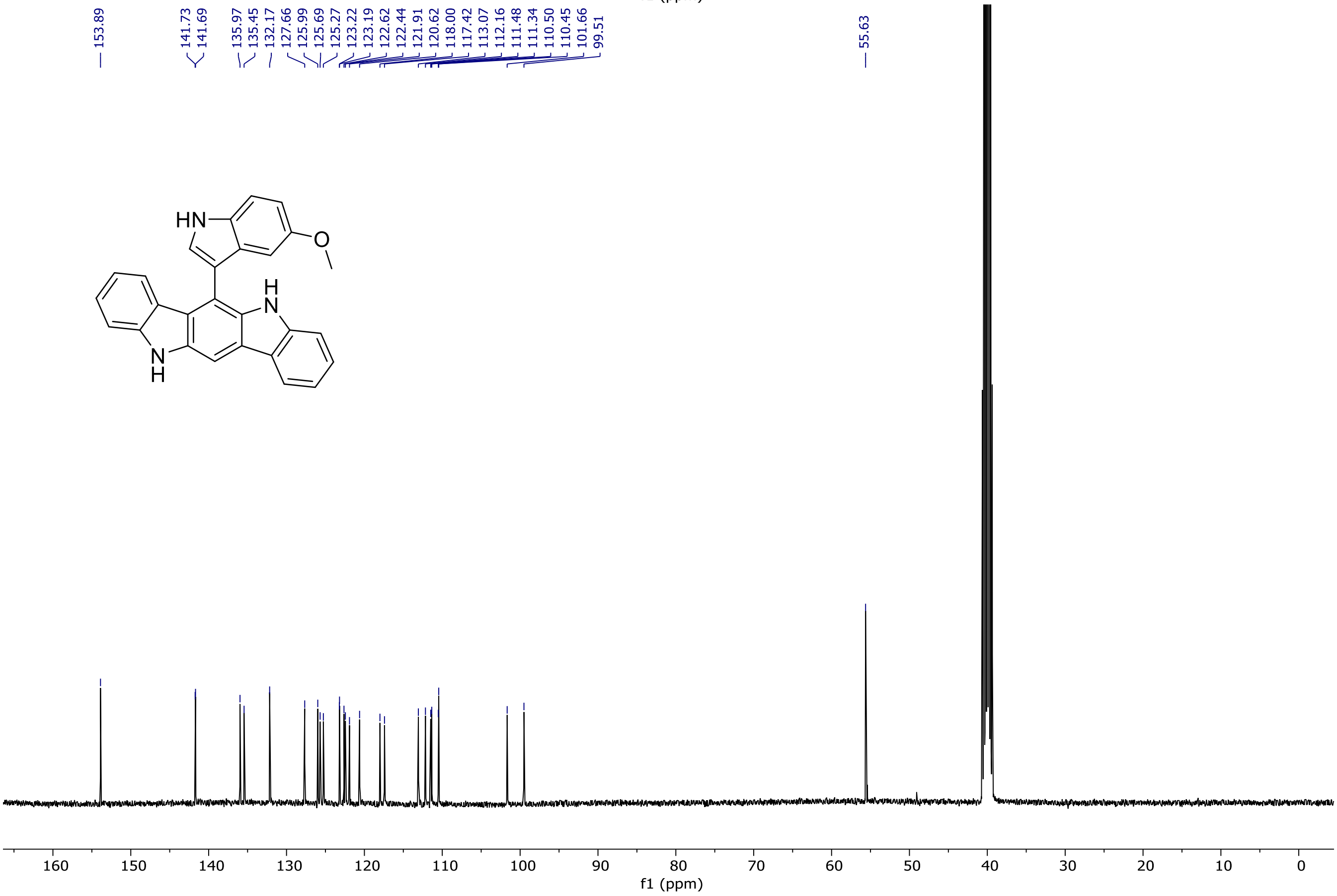

6-(5-Bromo-1*H*-indol-3-yl)-5,11-dihydroindolo[3,2-*b*]carbazole (5b)

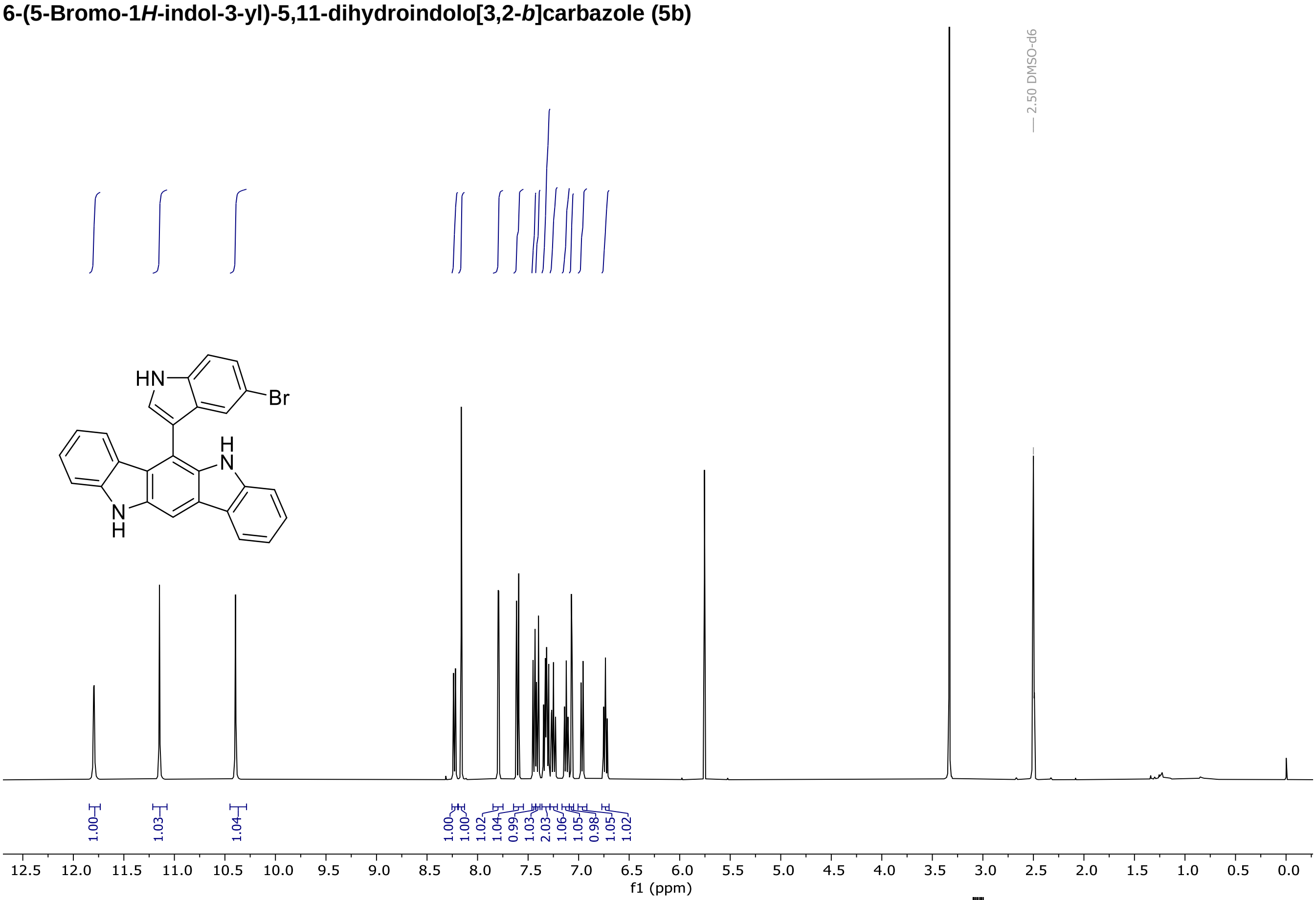

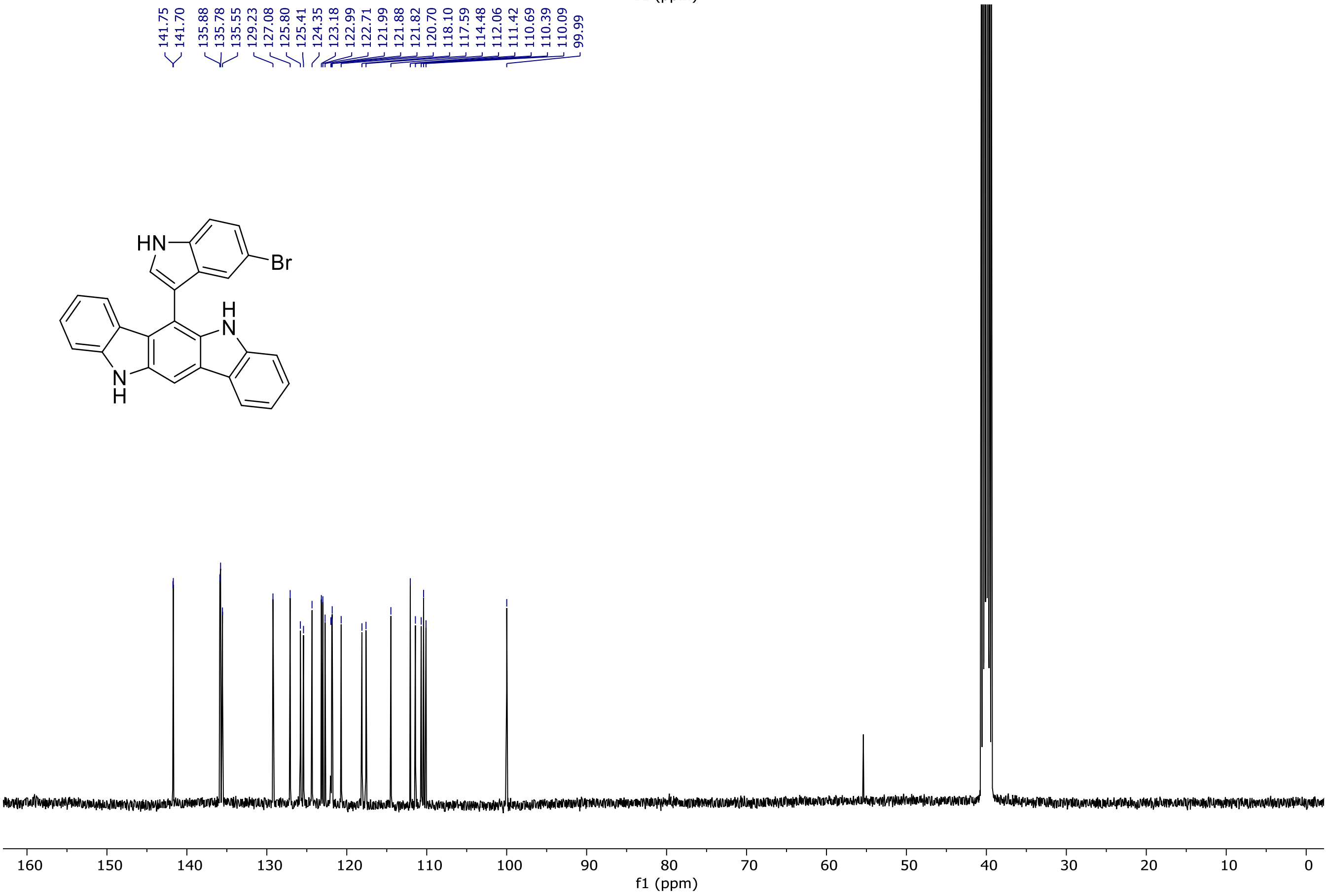

6-(1*H*-Indol-3-yl)-5,11-dihydroindolo[3,2-*b*]carbazole (5c)

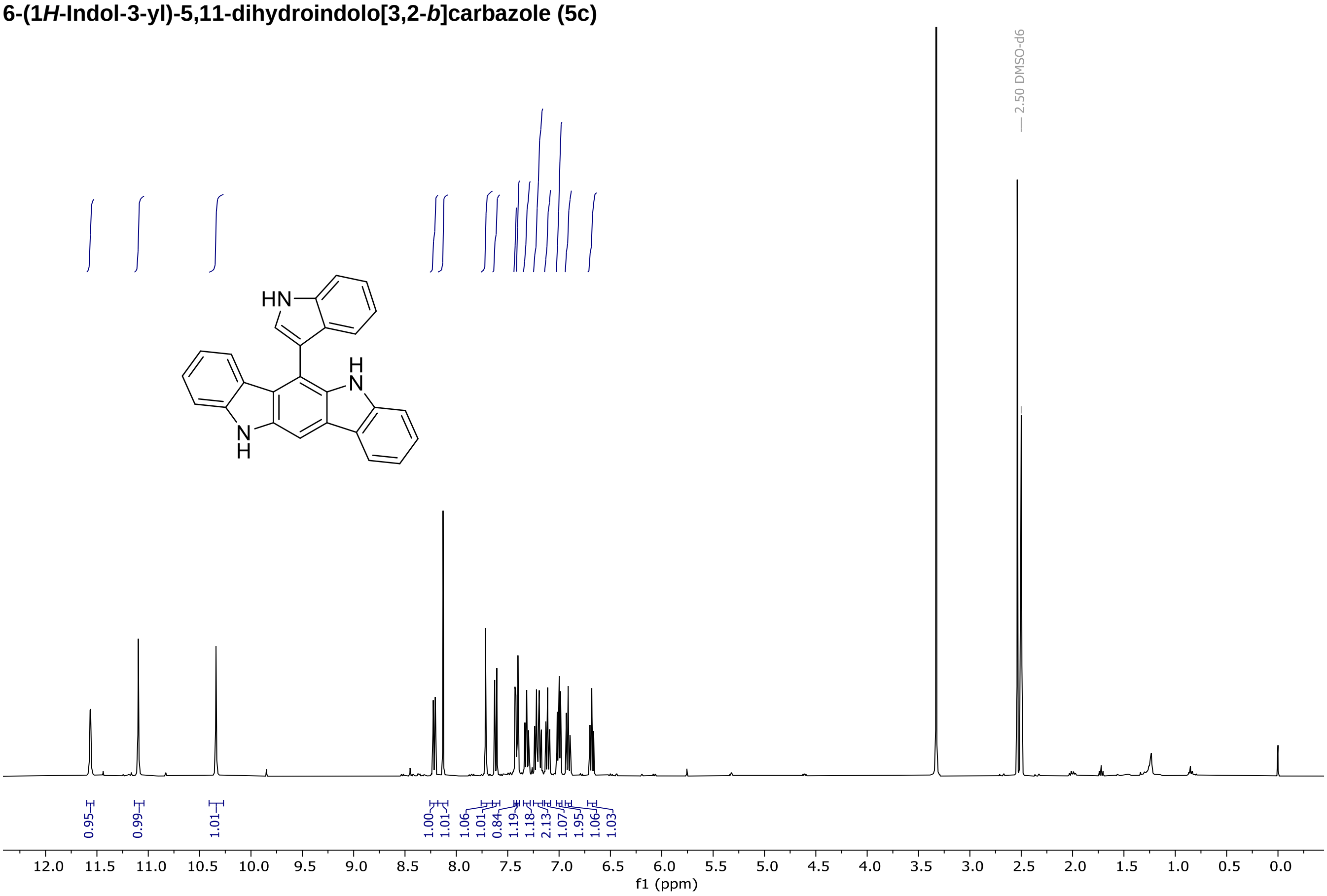

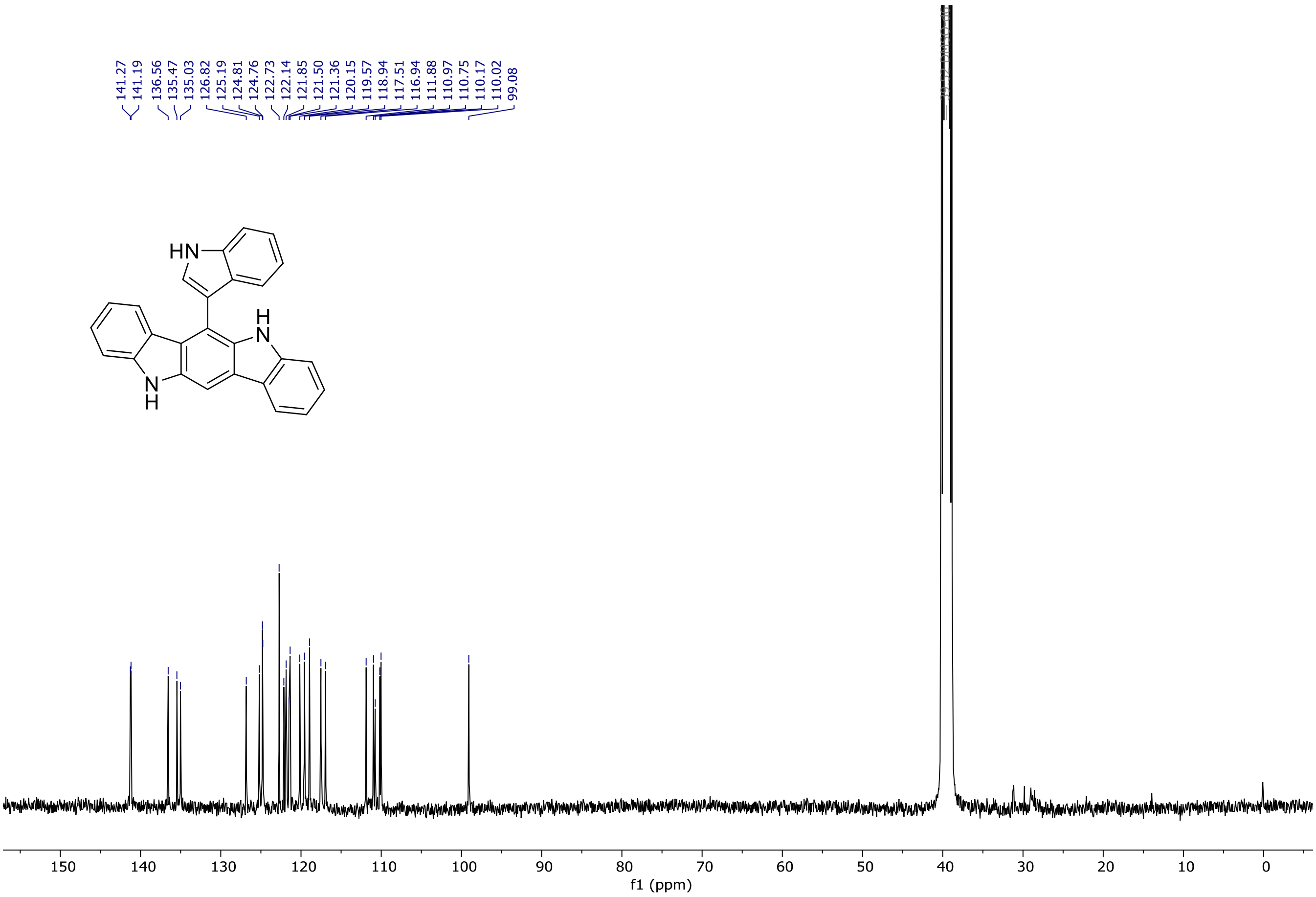

6-(1-Methyl-1*H*-indol-3-yl)-5,11-dihydroindolo[3,2-*b*]carbazole (5d)

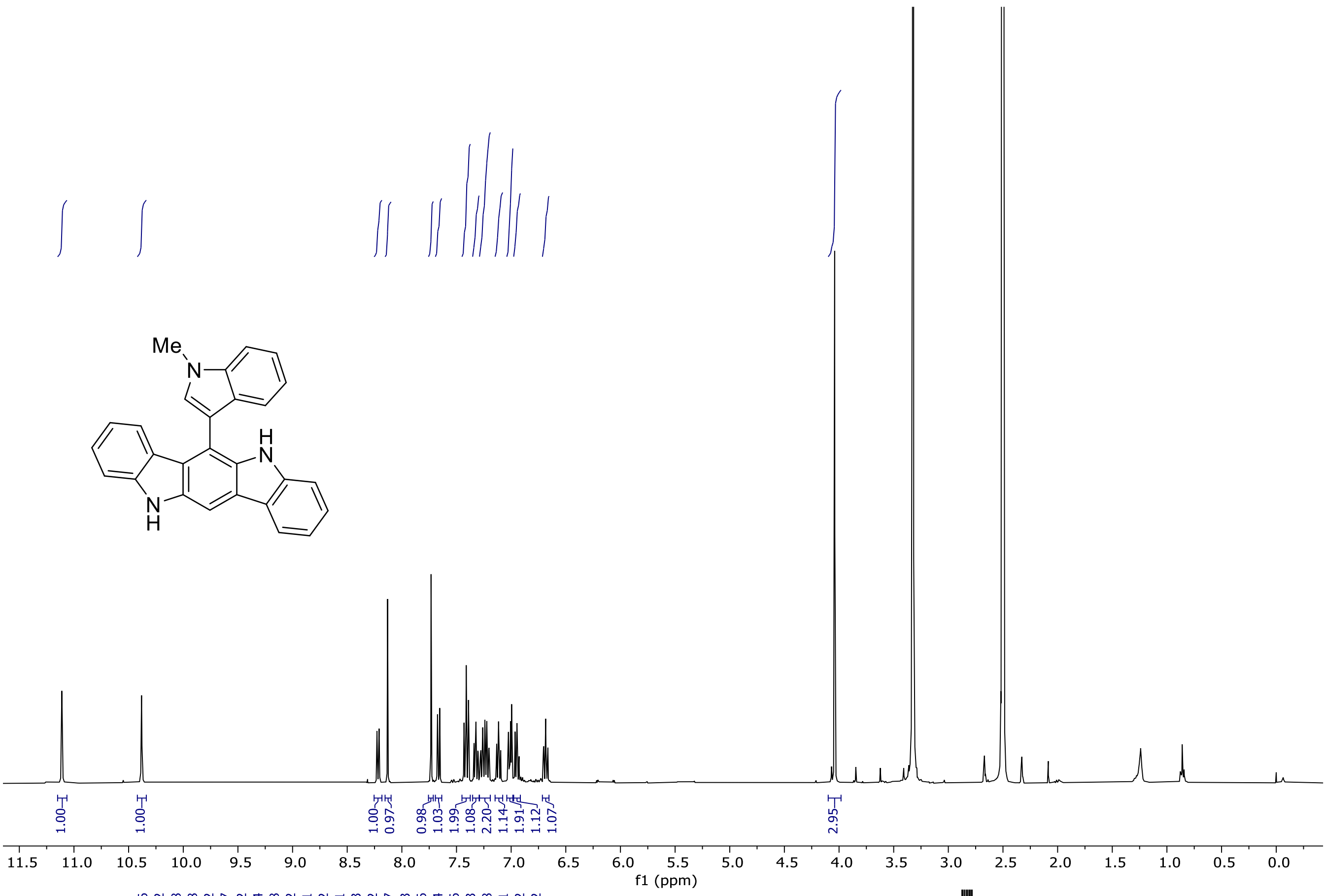

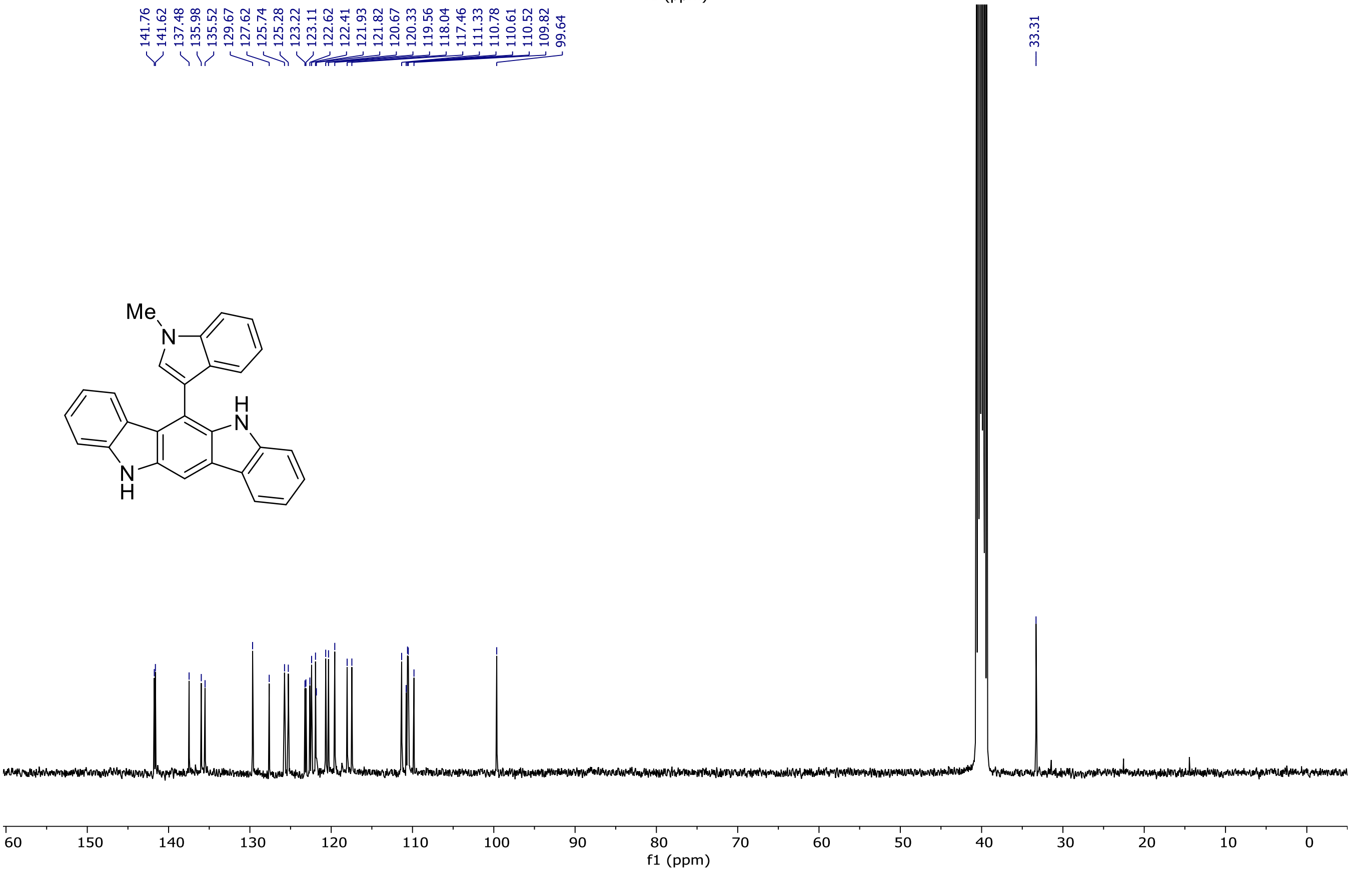

6-(2-Methyl-1*H*-indol-3-yl)-5,11-dihydroindolo[3,2-*b*]carbazole (5e)

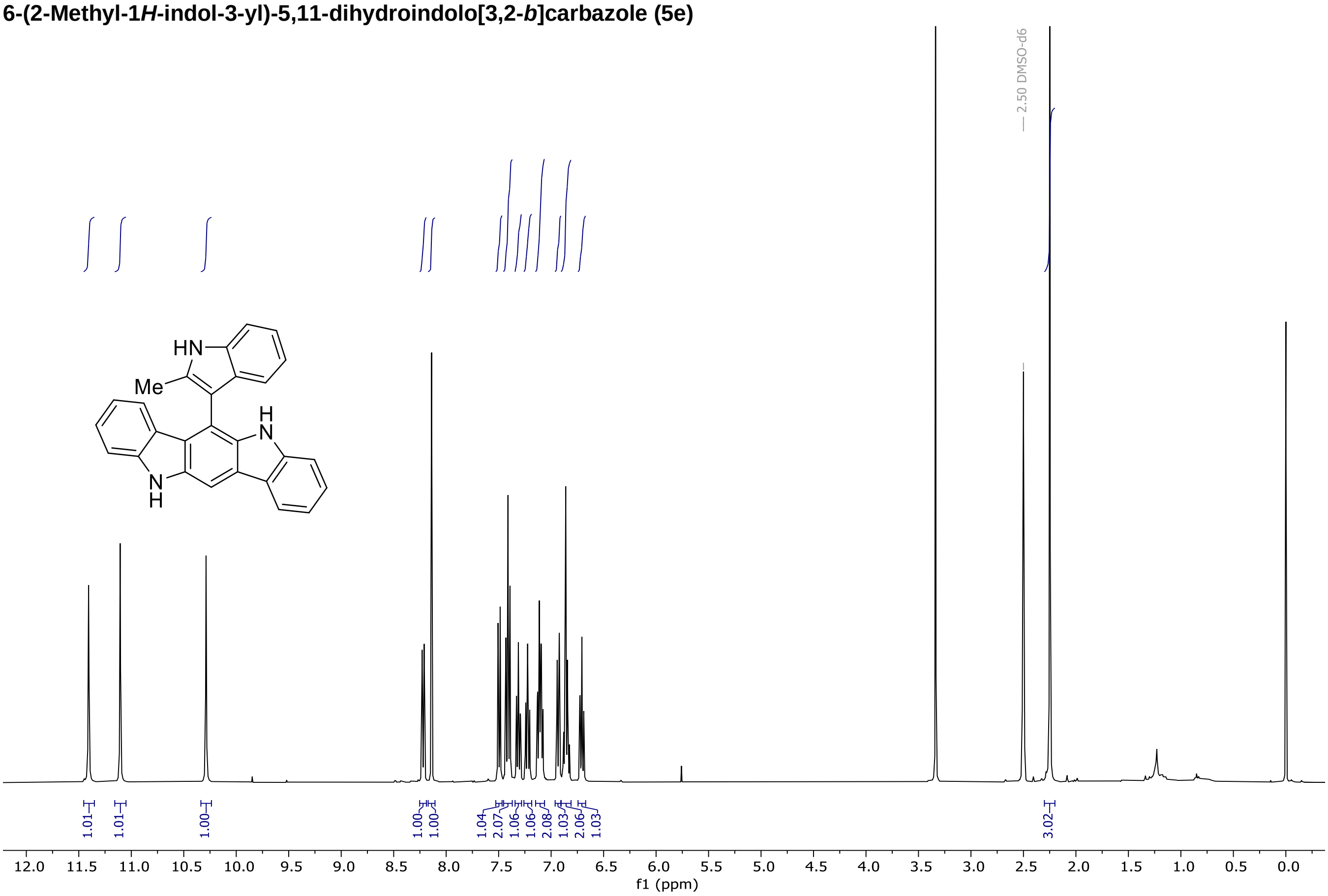

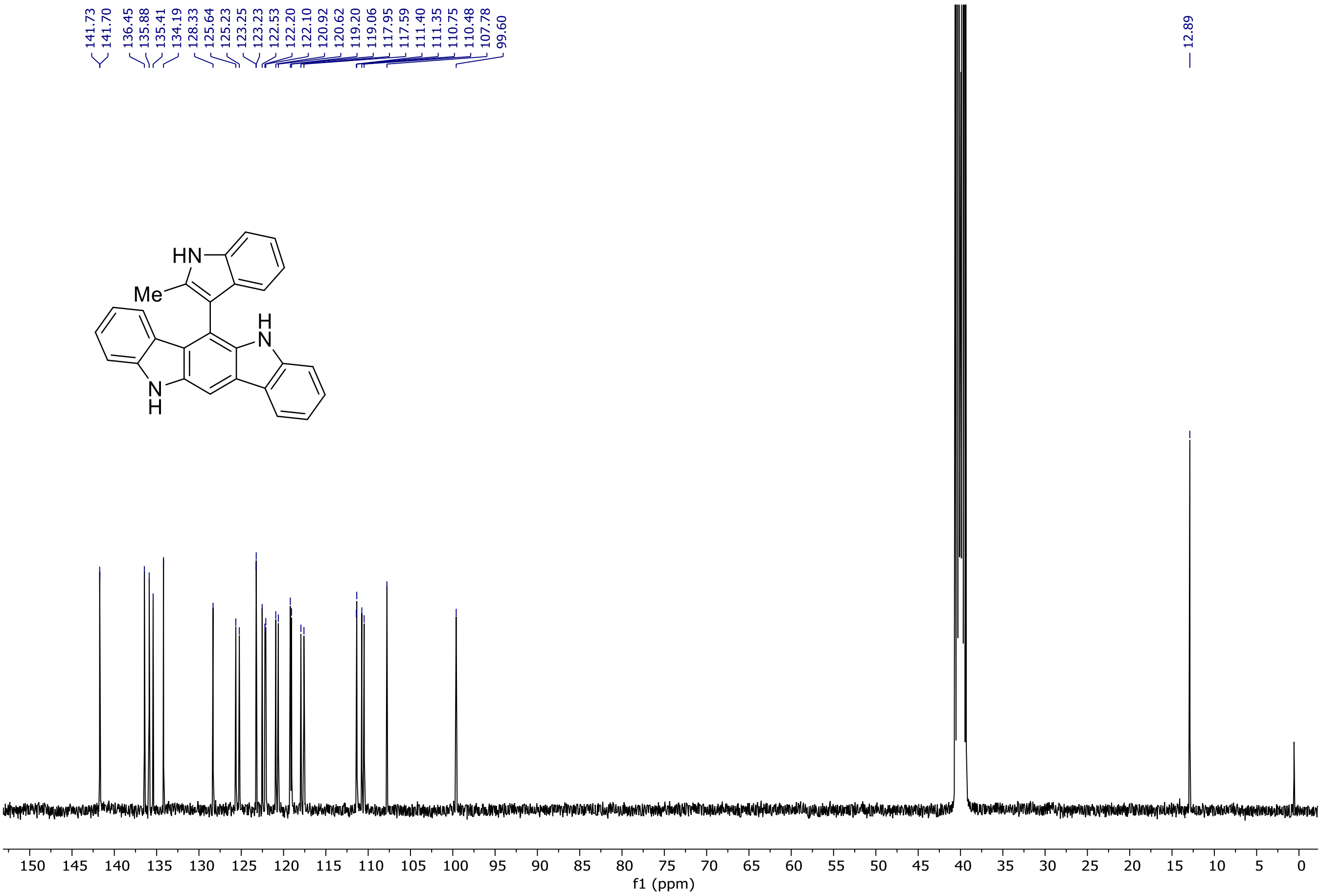

6-(2-Phenyl-1*H*-indol-3-yl)-5,11-dihydroindolo[3,2-*b*]carbazole (5f)

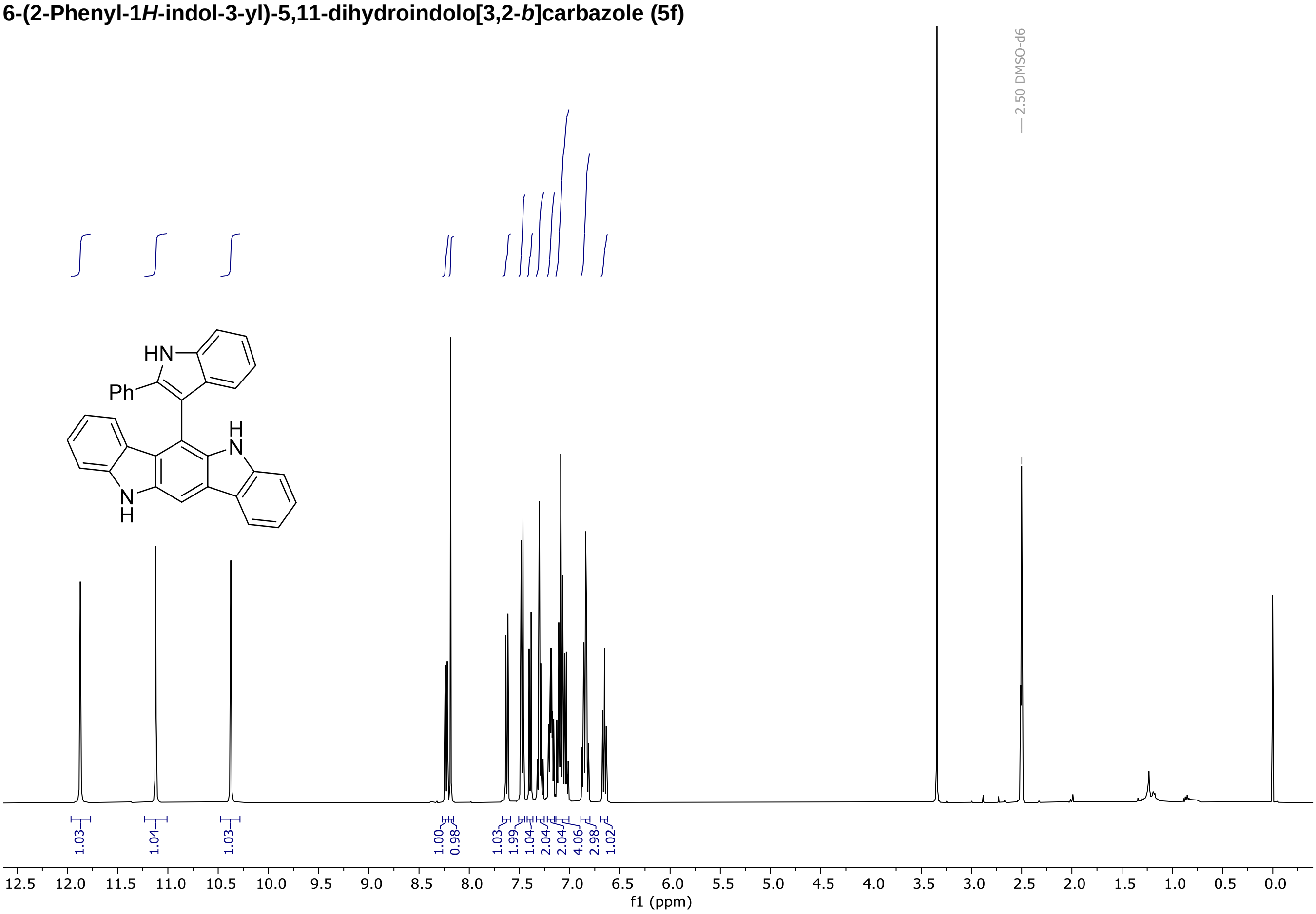

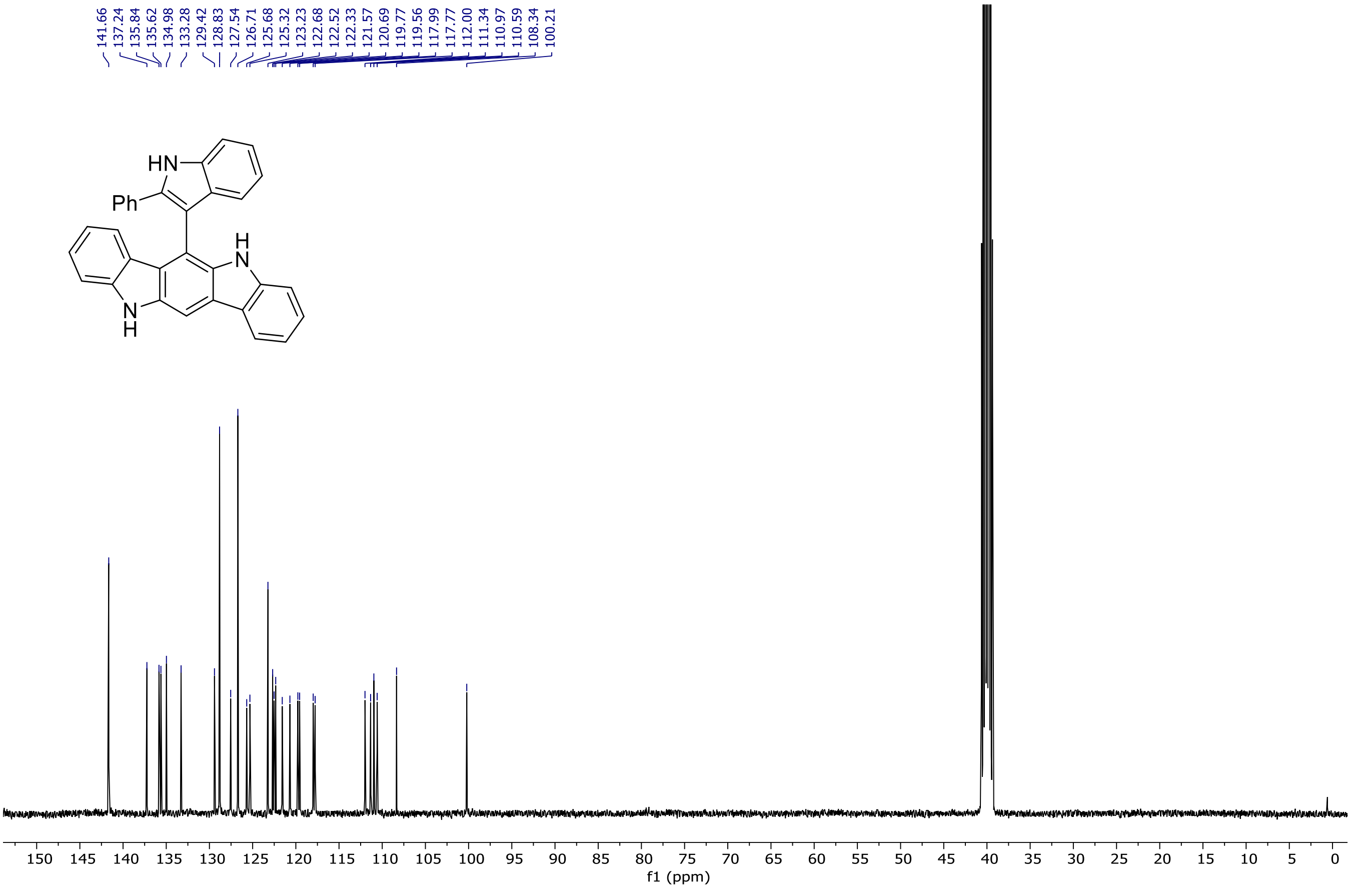

(3-(5,11-Dihydroindolo[3,2-*b*]carbazol-6-yl)-1*H*-indol-2-yl)methanol (5g)

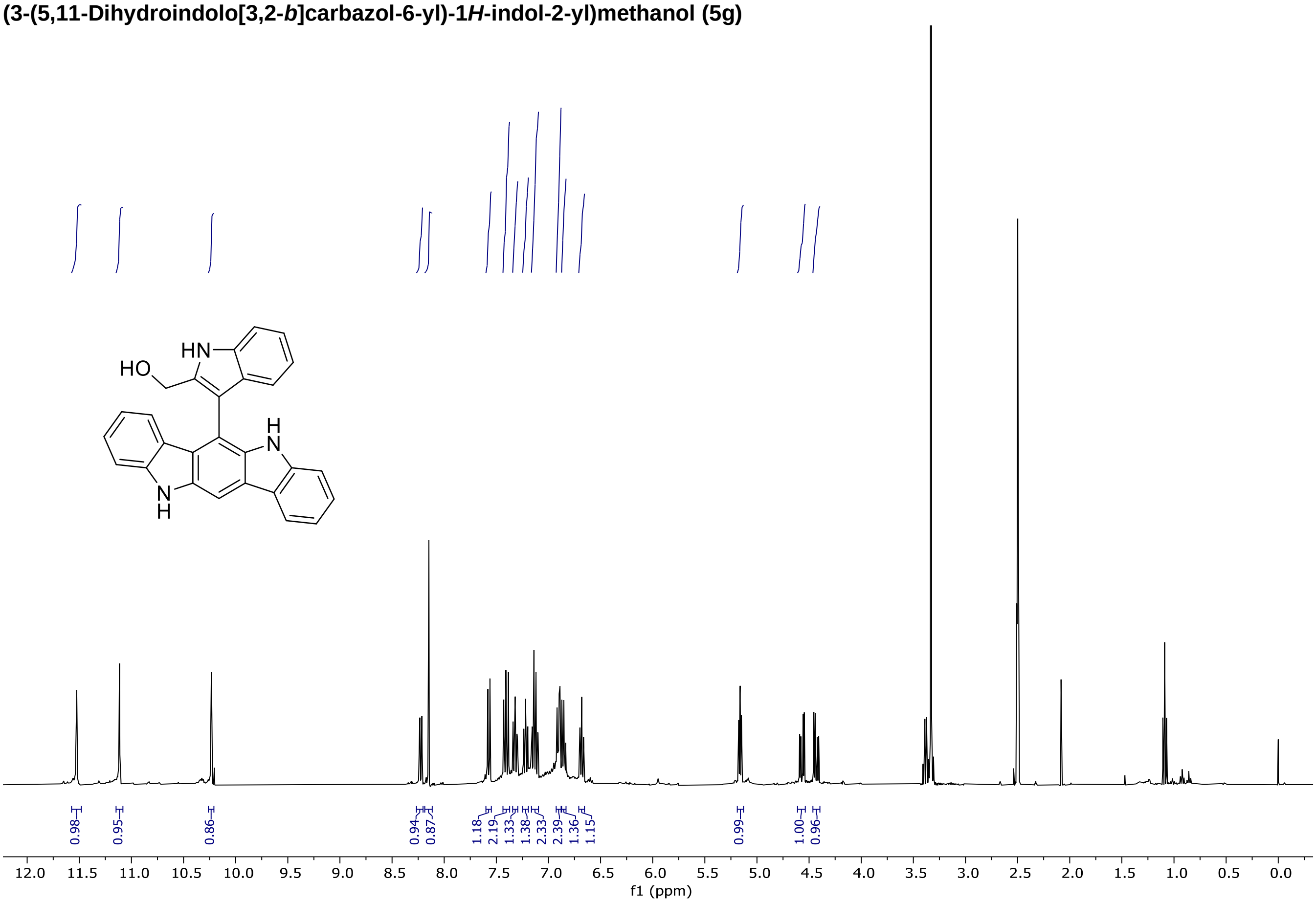

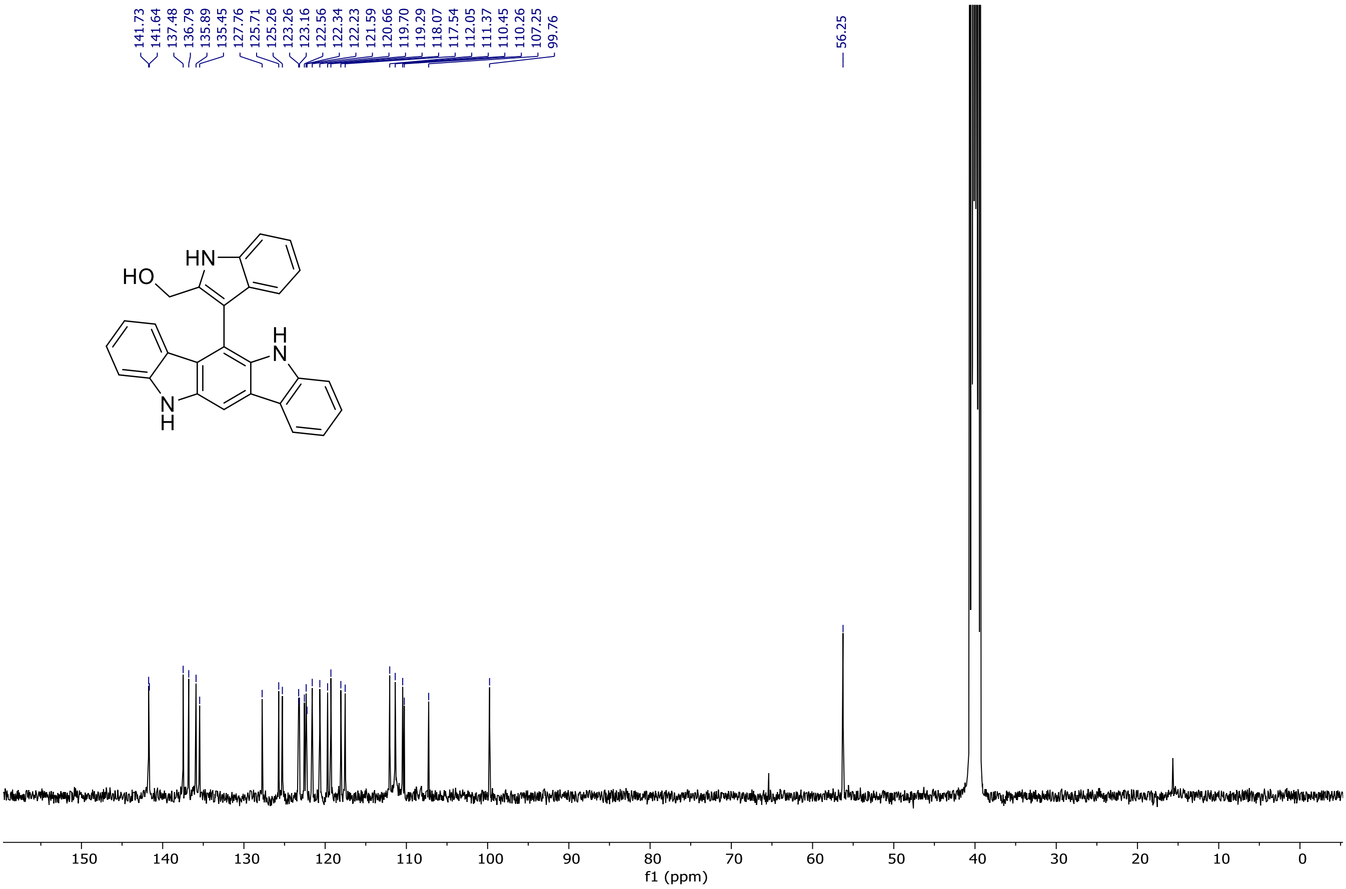

6-(4-Methyl-1*H*-indol-3-yl)-5,11-dihydroindolo[3,2-*b*]carbazole (5h)

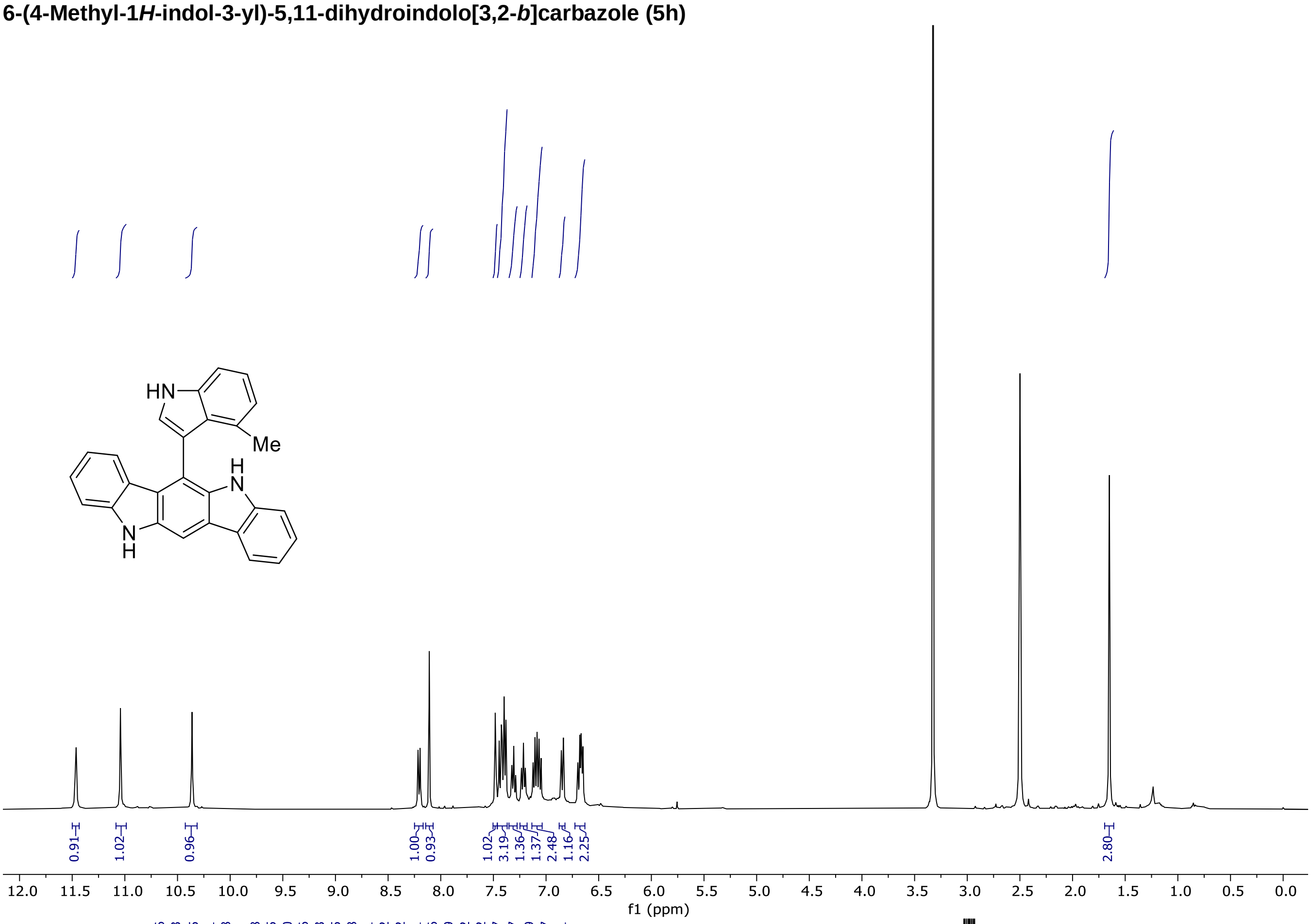

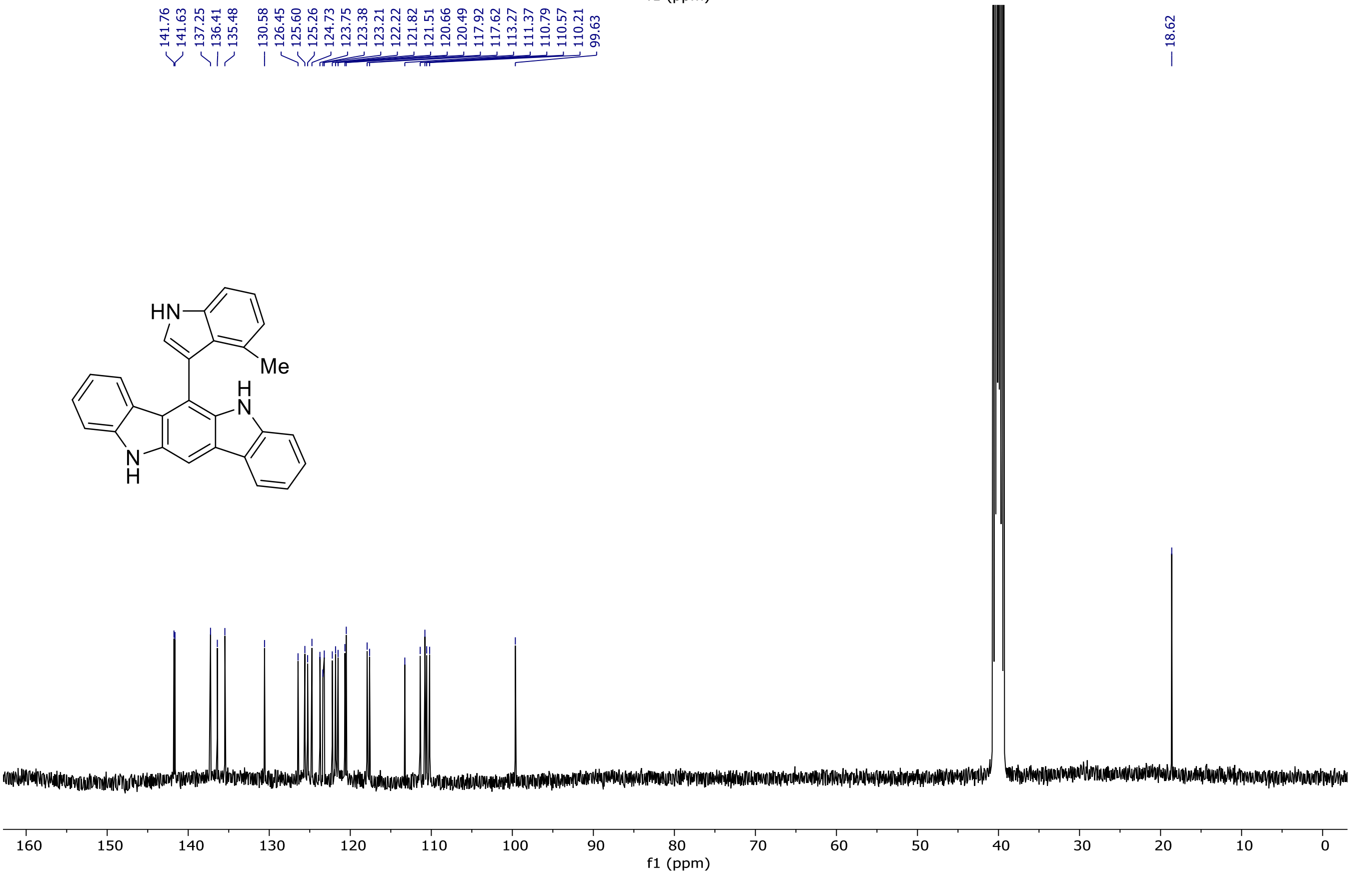

6-(7-Methyl-1*H*-indol-3-yl)-5,11-dihydroindolo[3,2-*b*]carbazole (5i)

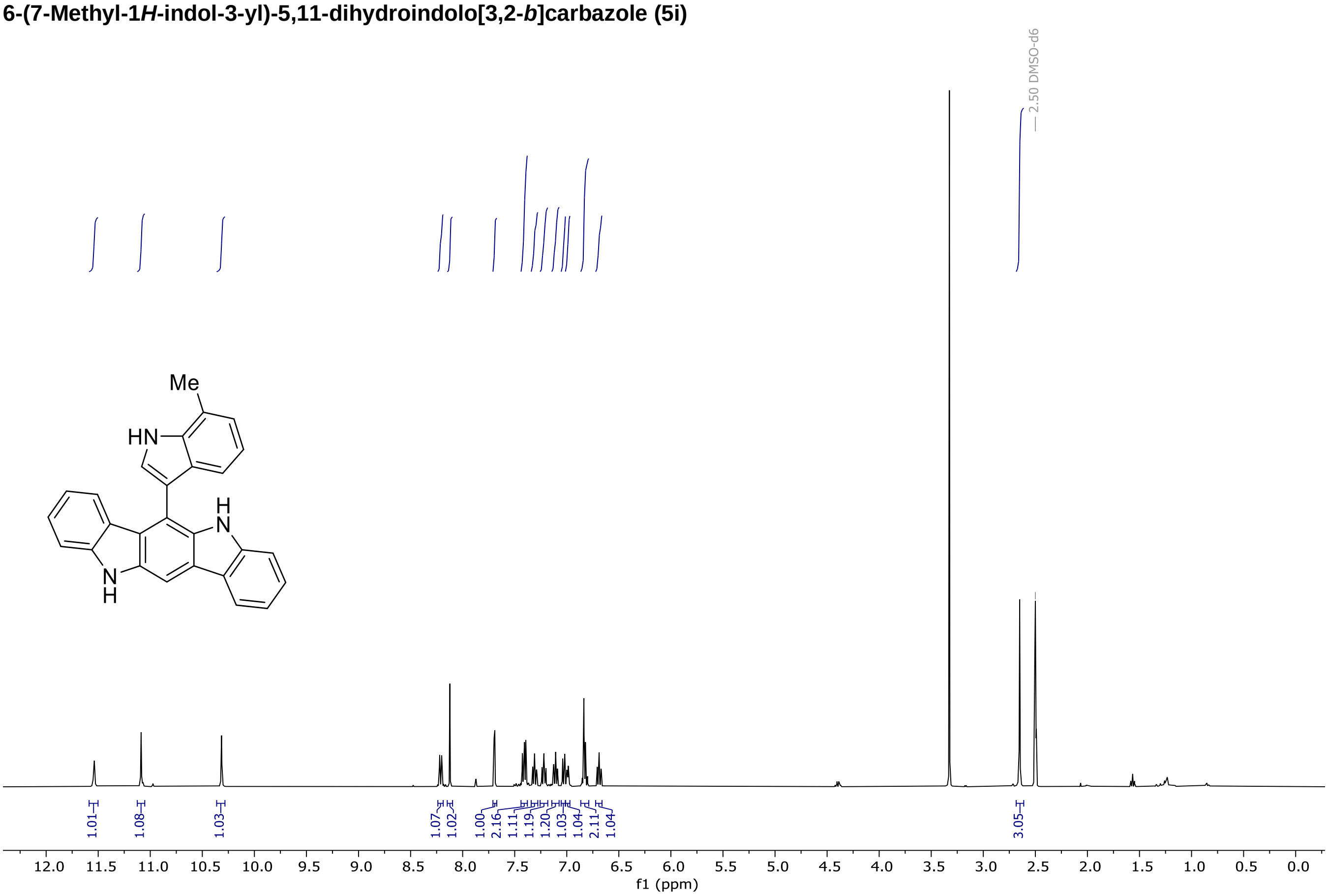

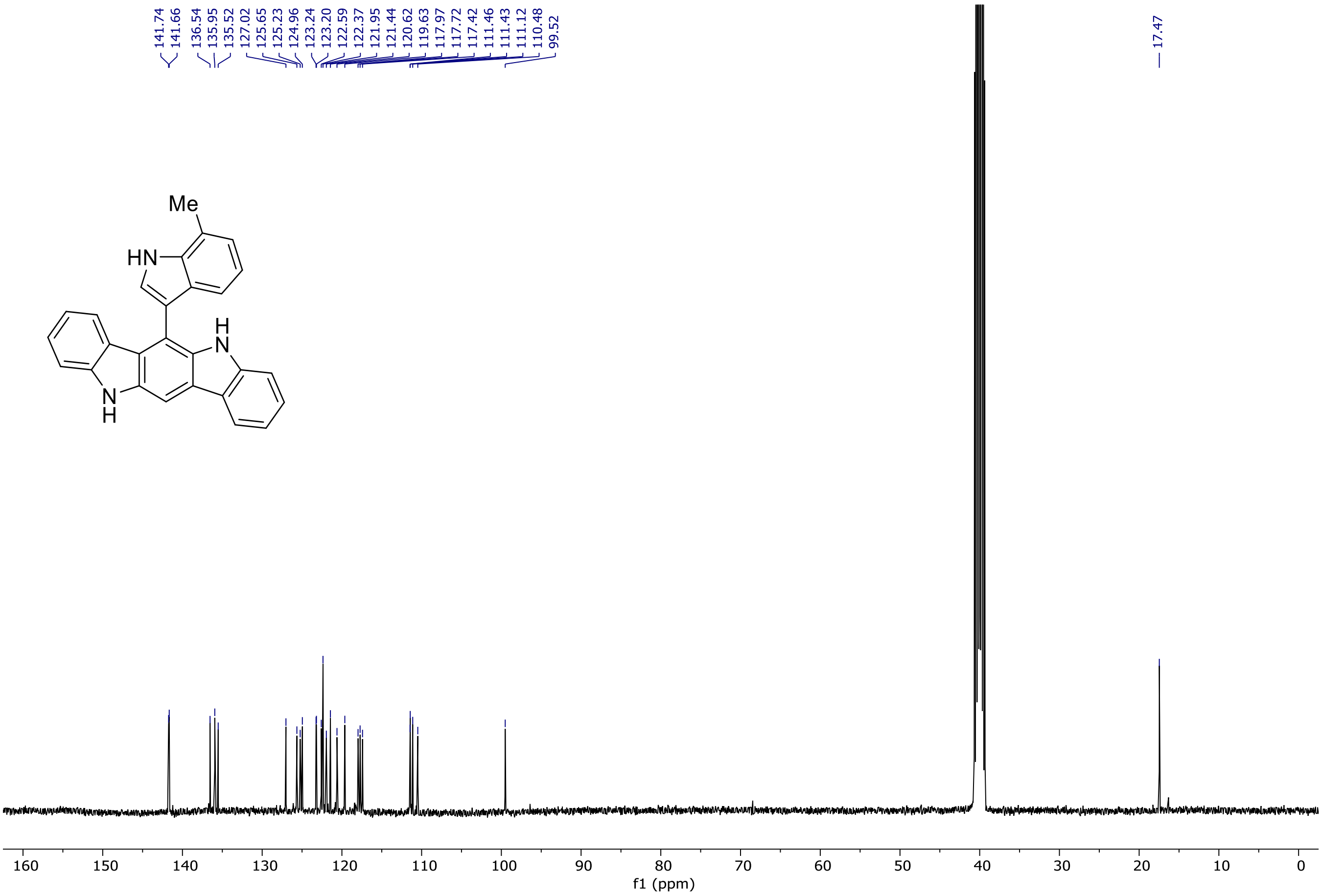

5,11-Dmethyl-6-(2-methyl-1*H*-indol-3-yl)-5,11-dihydroindolo[3,2-*b*]carbazole (5j)

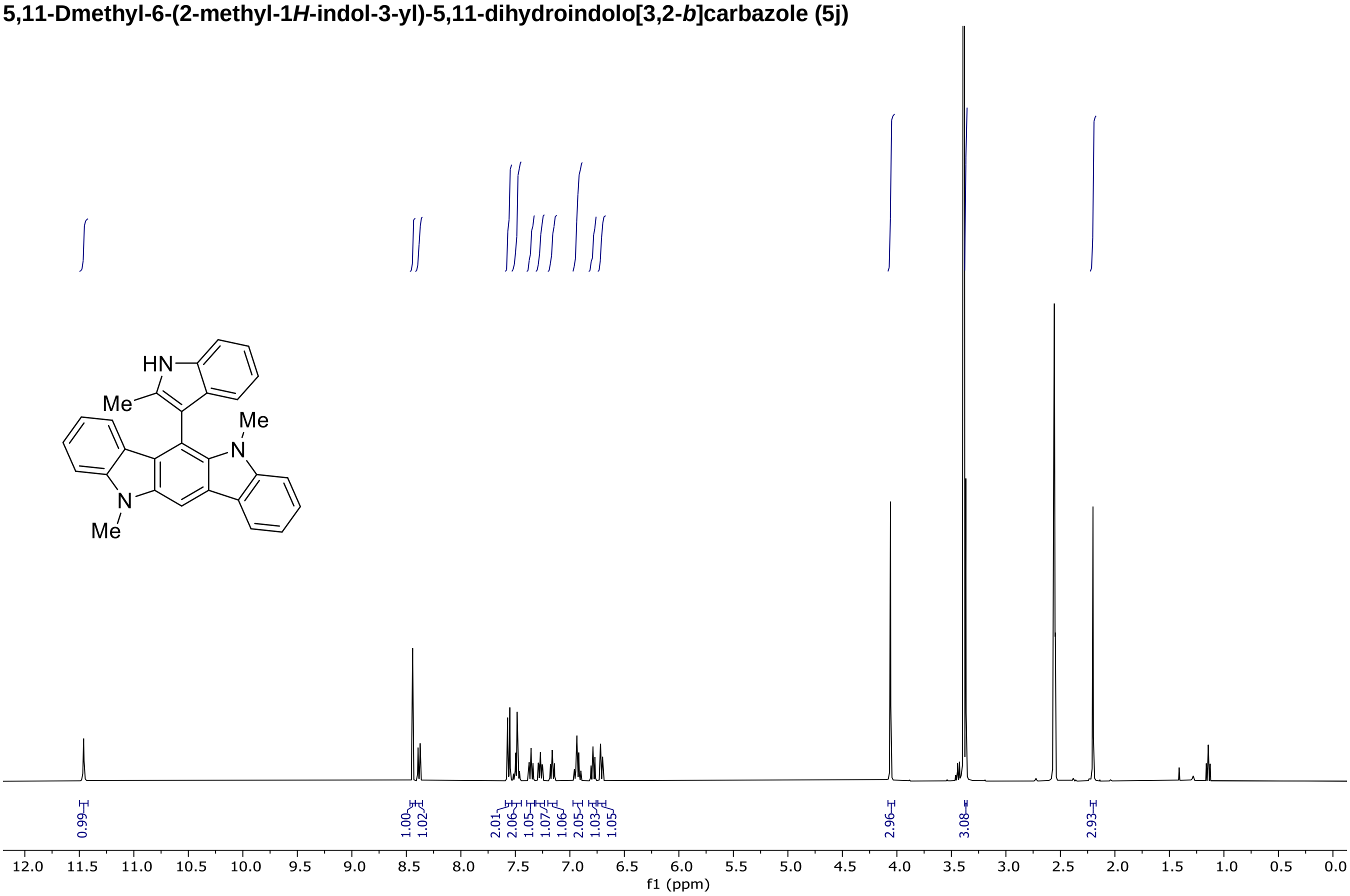

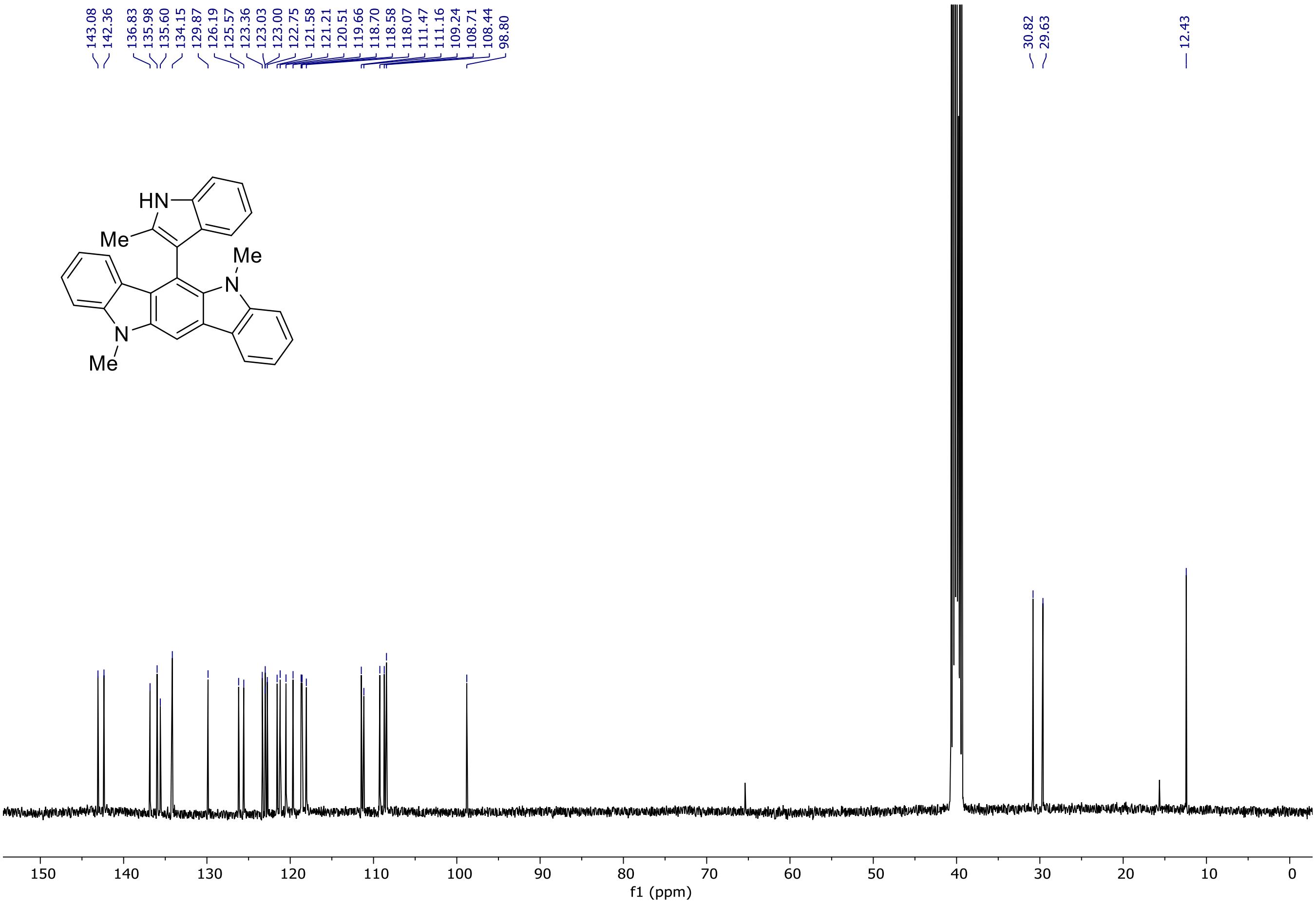

2,8-Difluoro-6-(2-methyl-1*H*-indol-3-yl)-5,11-dihydroindolo[3,2-*b*]carbazole (5k)

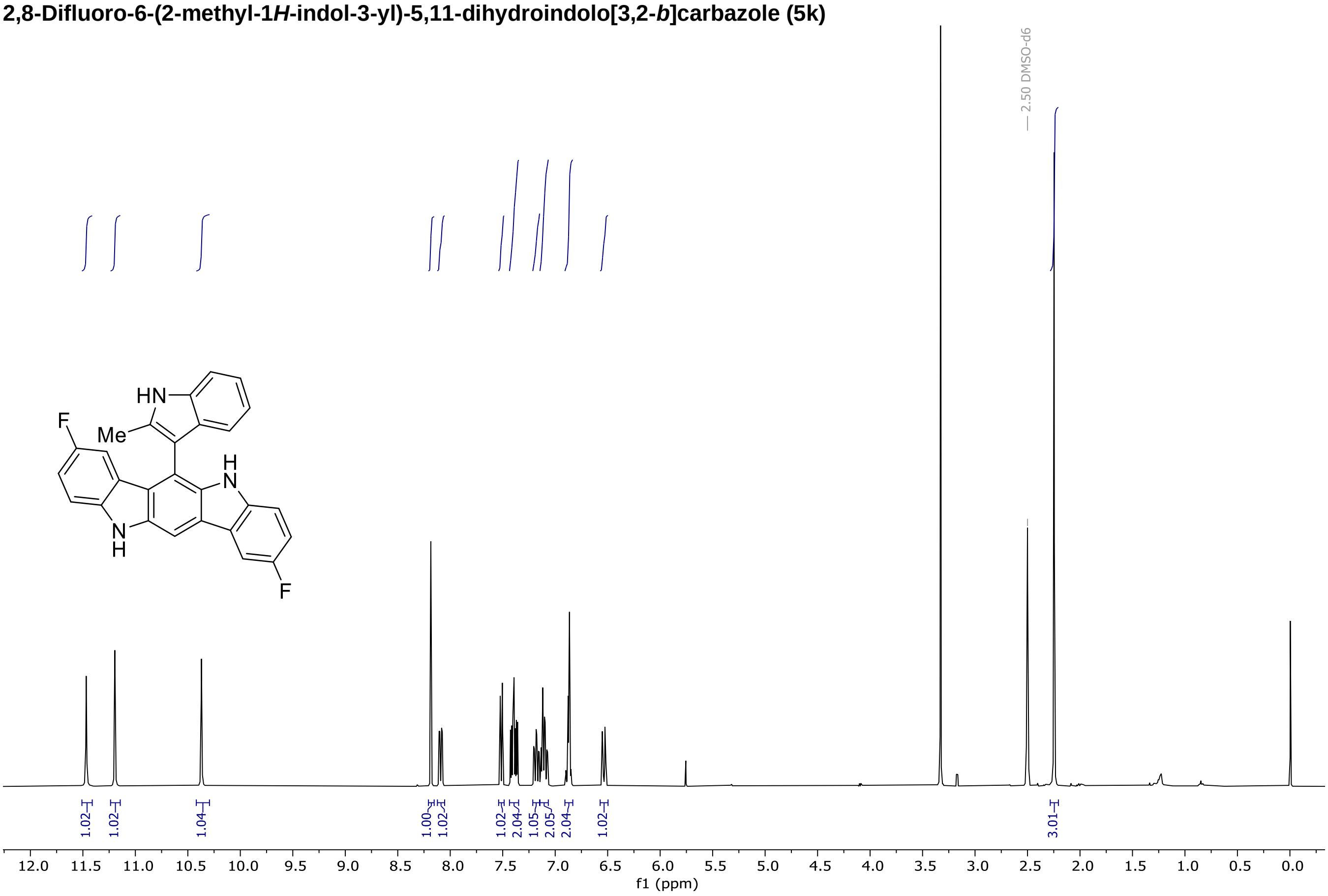

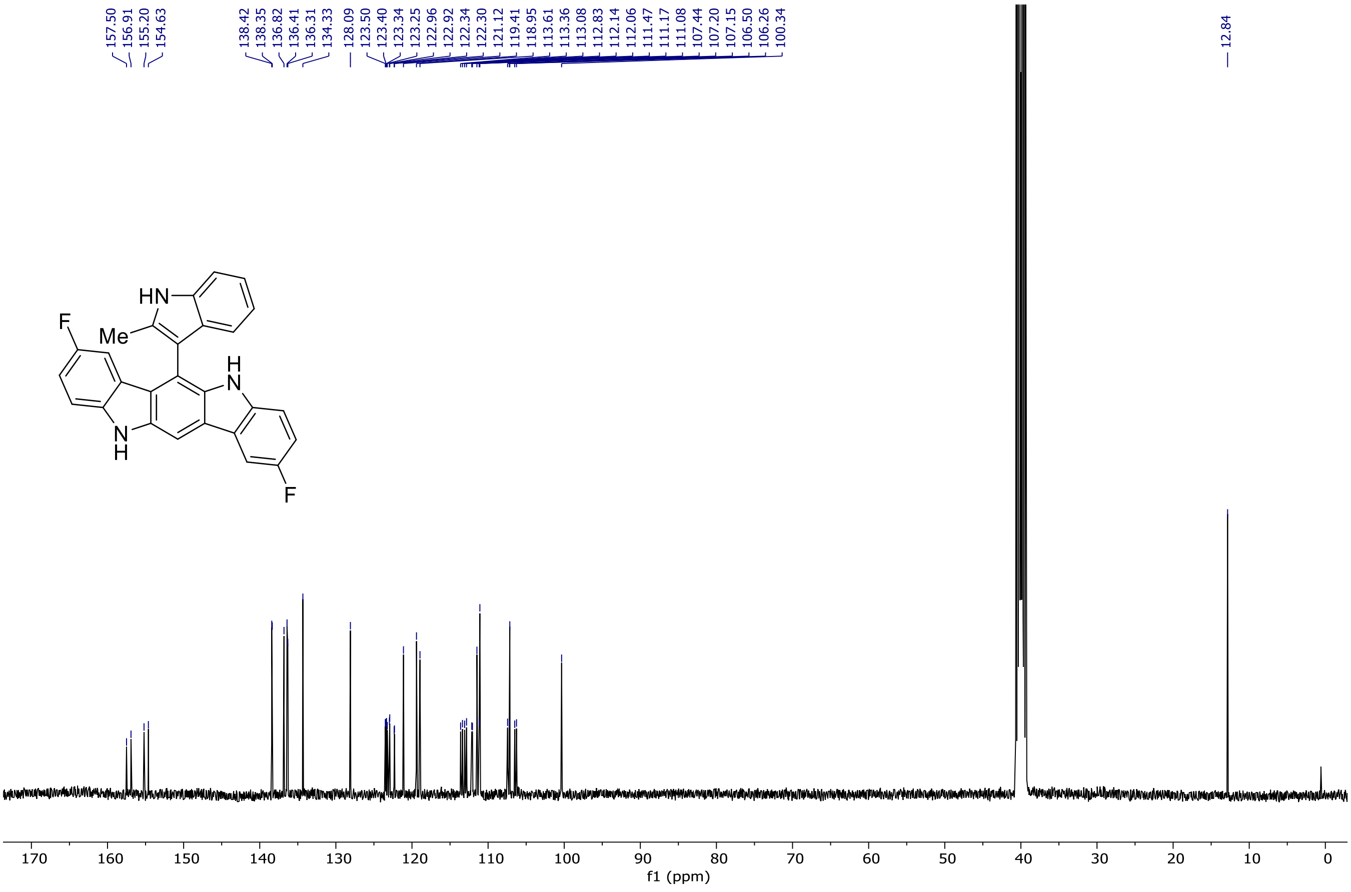

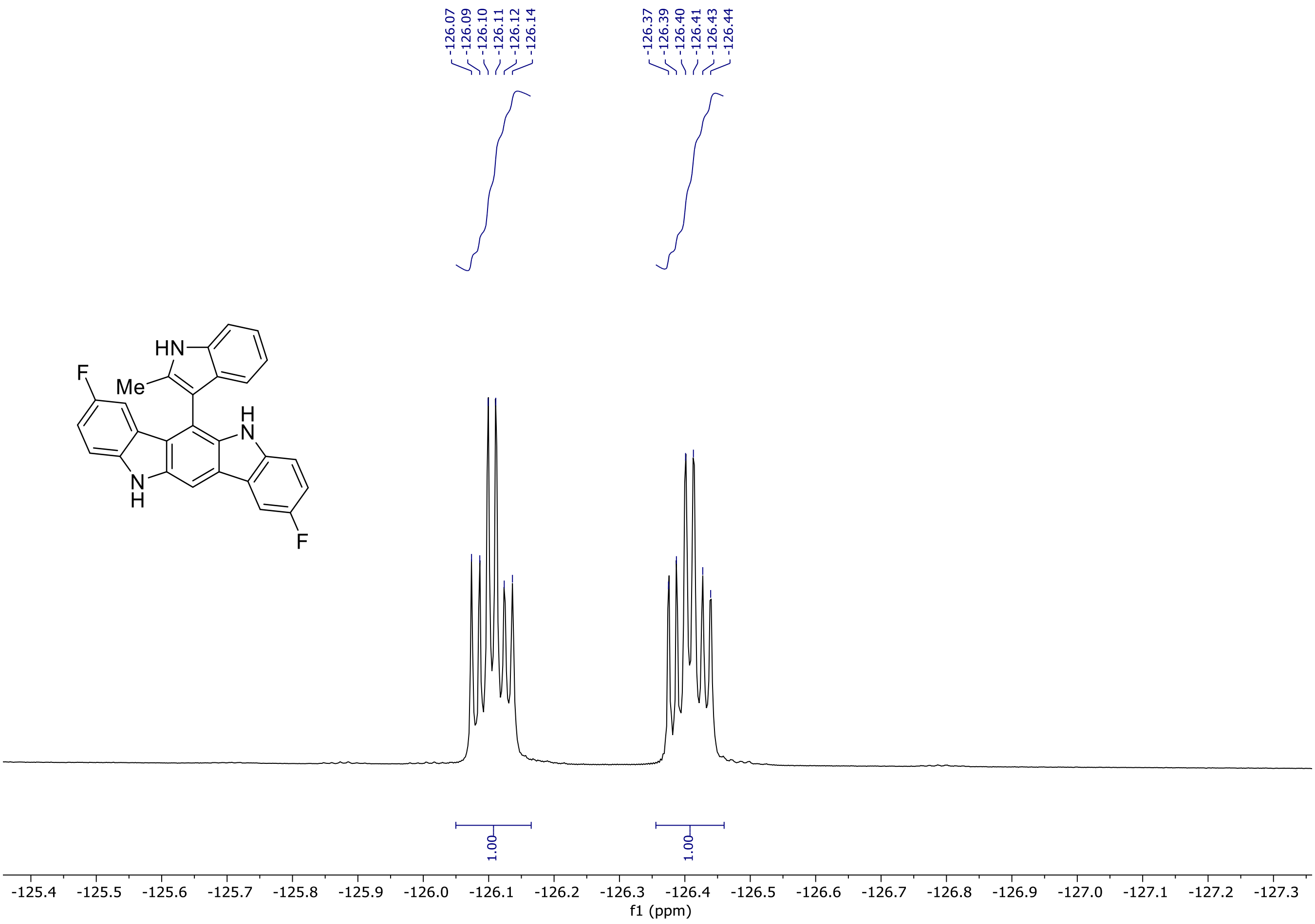
-126.07
-126.09
-126.10
-126.11
-126.12
-126.14
-126.37
-126.39
-126.40
-126.41
-126.43
-126.44
HN
Me
F
H
N
N
H
F
1.00
1.00
-125.4 -125.5 -125.6 -125.7 -125.8 -125.9 -126.0 -126.1 -126.2 -126.3 -126.4 -126.5 -126.6 -126.7 -126.8 -126.9 -127.0 -127.1 -127.2 -127.3
f1 (ppm)

2,8-Dimethoxy-6-(2-methyl-1*H*-indol-3-yl)-5,11-dihydroindolo[3,2-*b*]carbazole (5l)

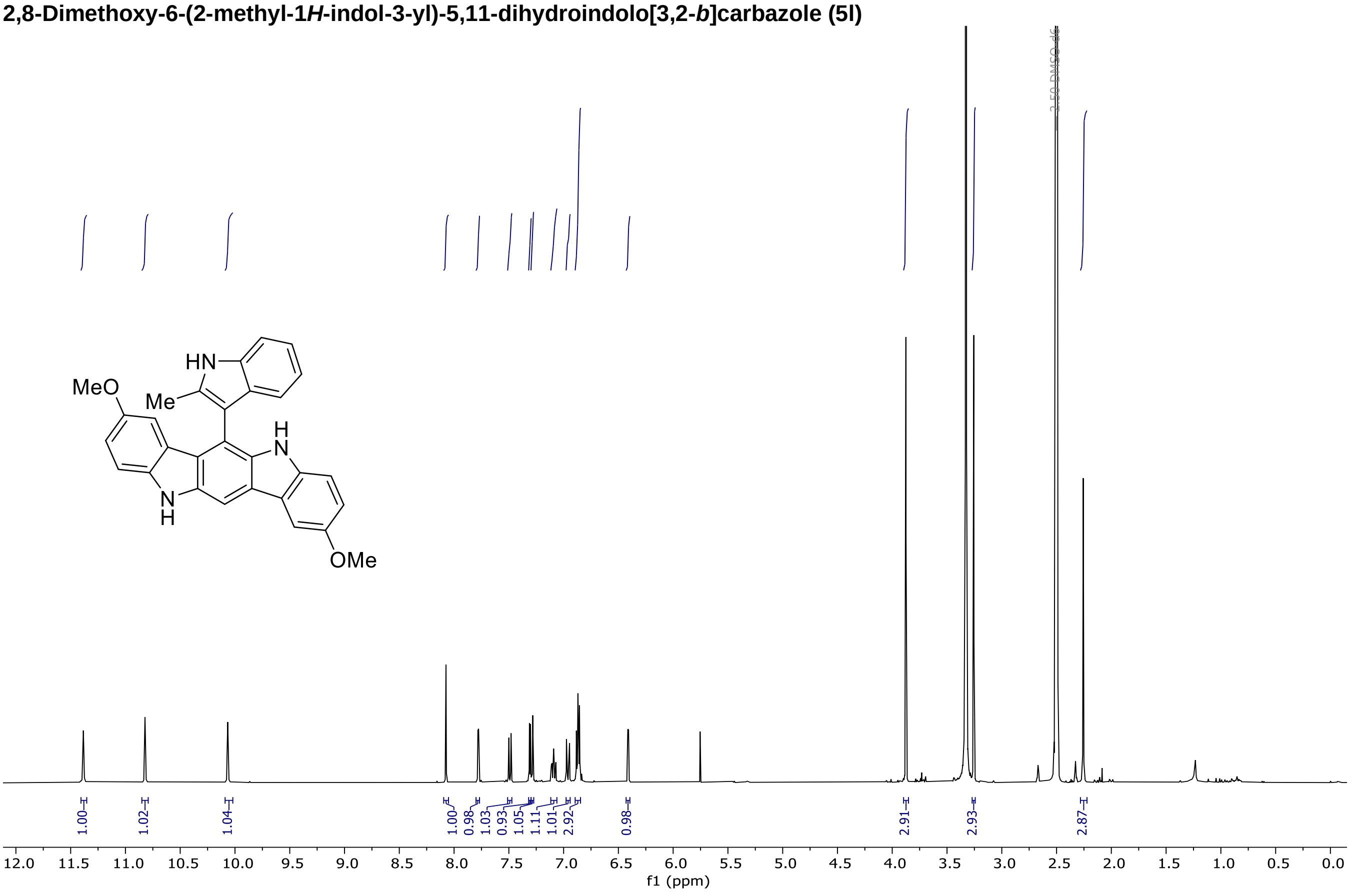

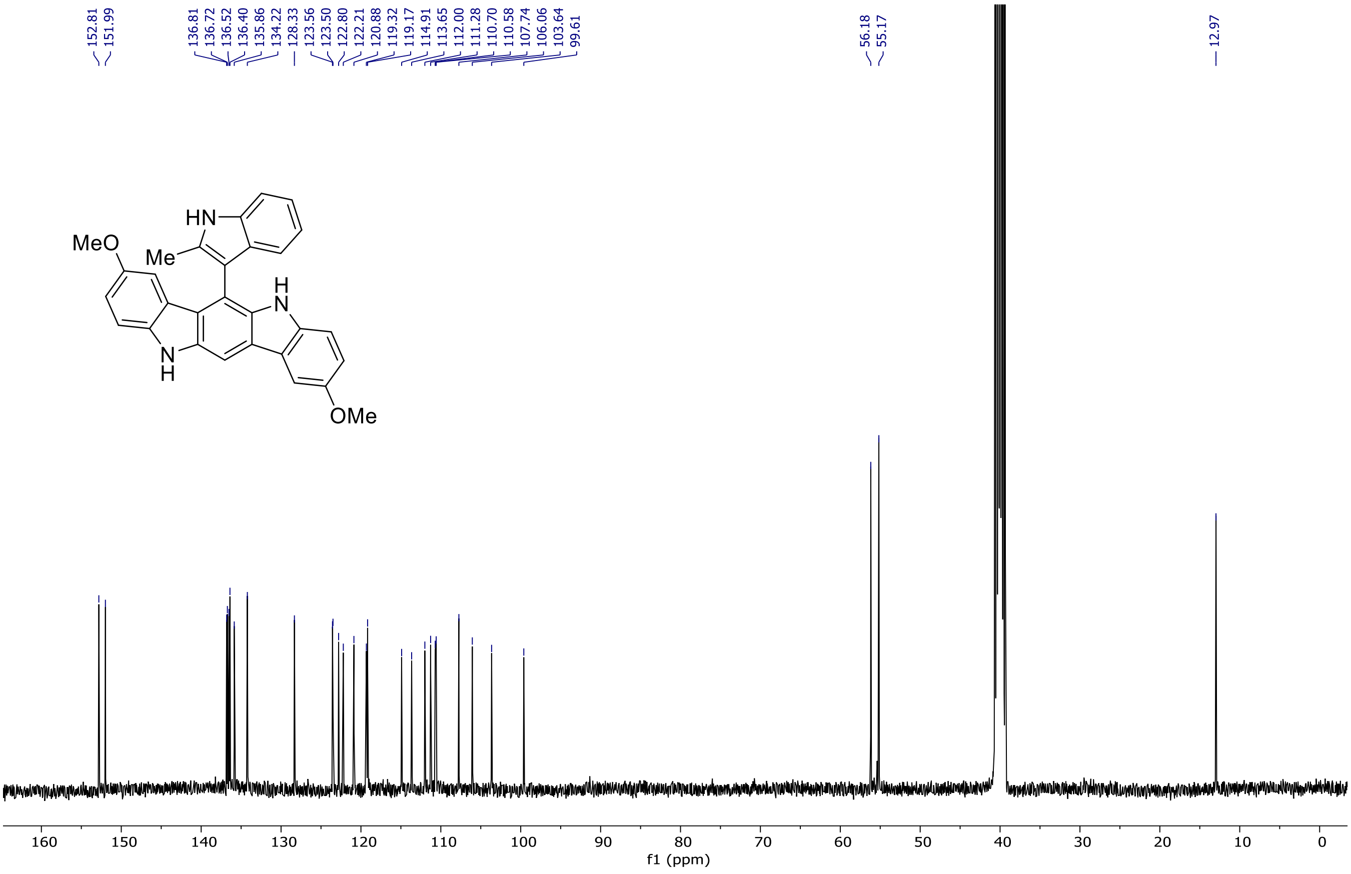

3-(5,11-Dihydroindolo[3,2-*b*]carbazole-6-yl)-1*H*-indole-6-carboxylic acid (5m)

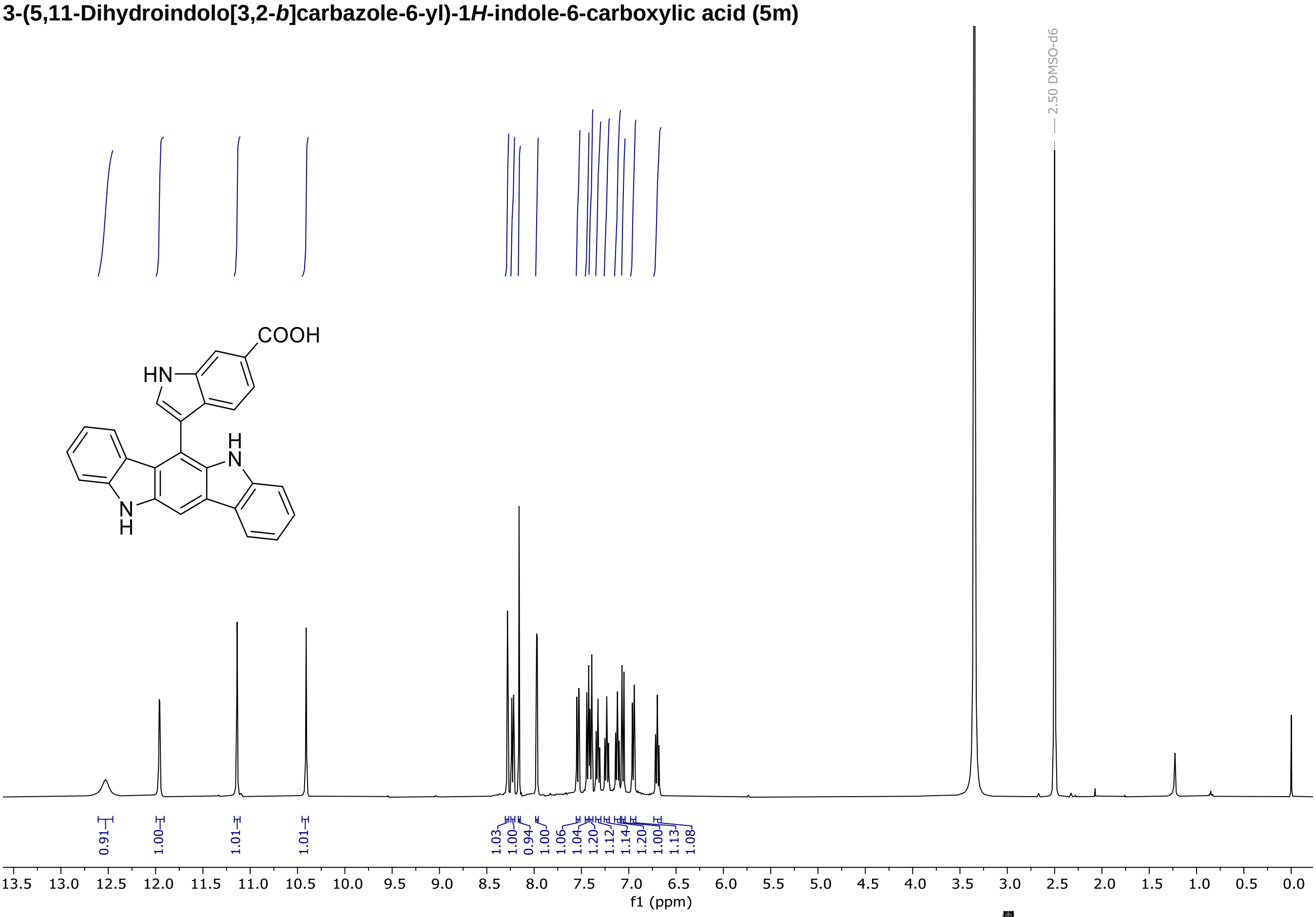

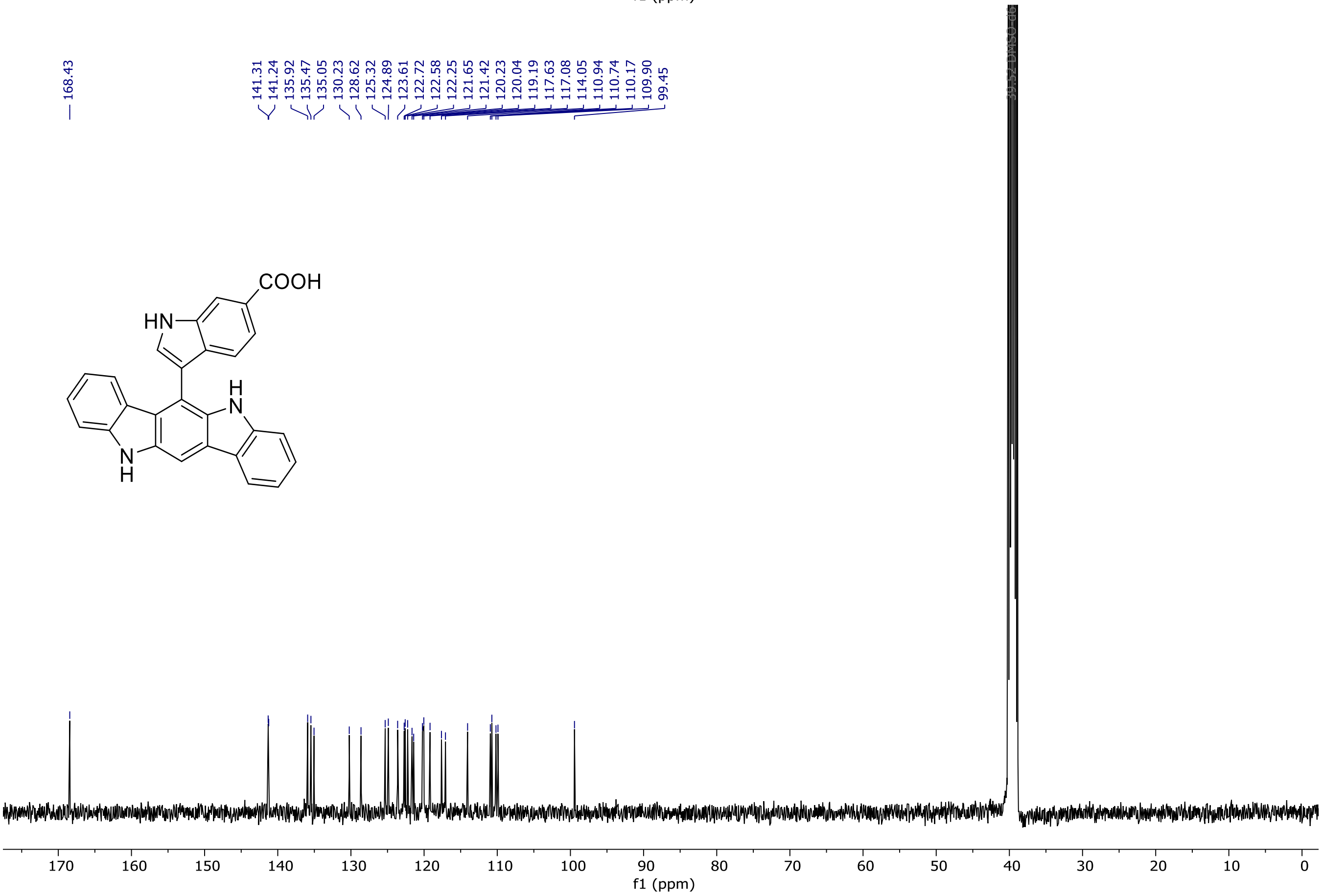

10.3. Knoevenagel Adducts and Bis- / Tris-indolyl *C*ompounds (Series 6-7)

5-((1*H*-Indol-2-yl)methylene)-1,3-dimethylpyrimidine-2,4,6(1*H*,3*H*,5*H*)-trione (6a)

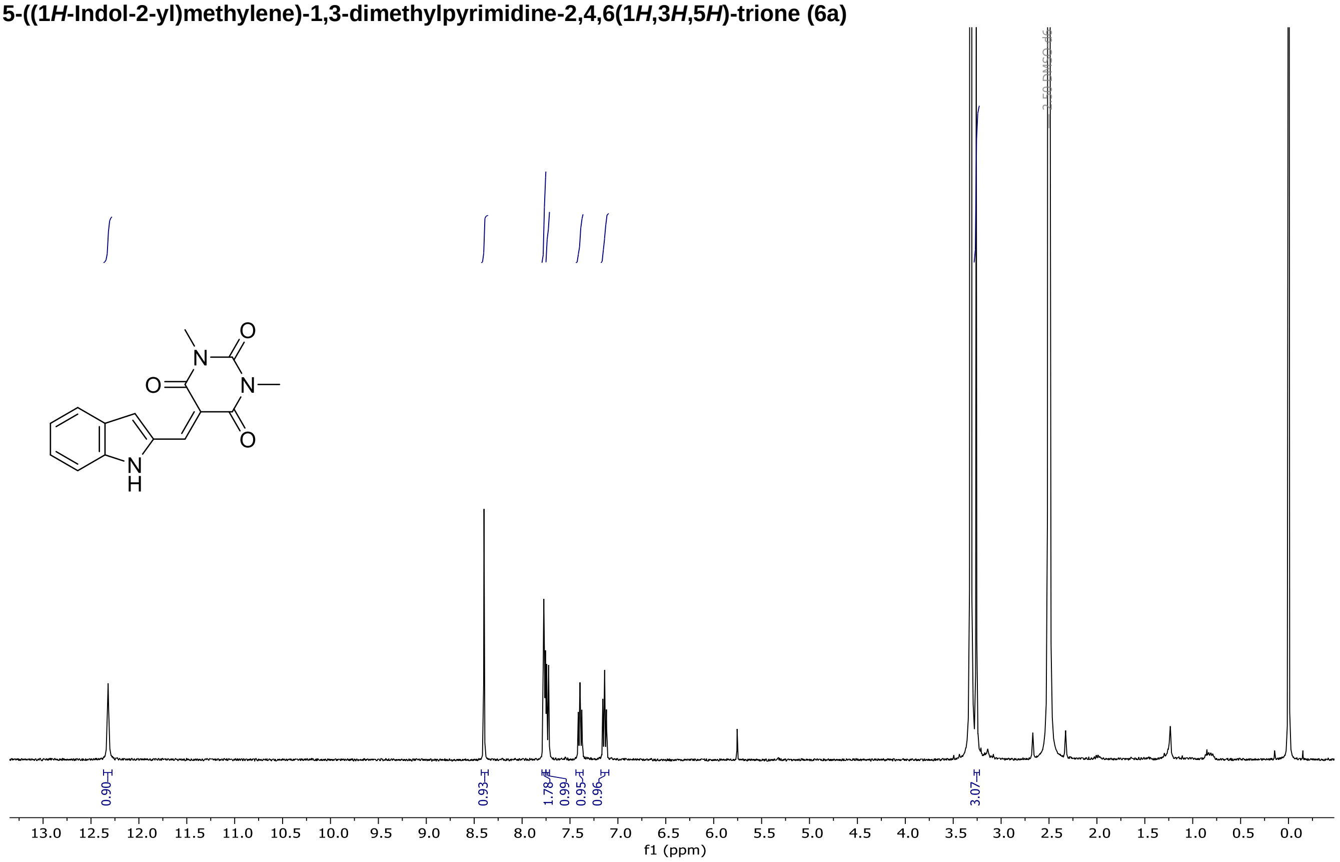

2-((1*H*-Indol-2-yl)methylene)-1*H*-indene-1,3(2*H*)-dione (6b)

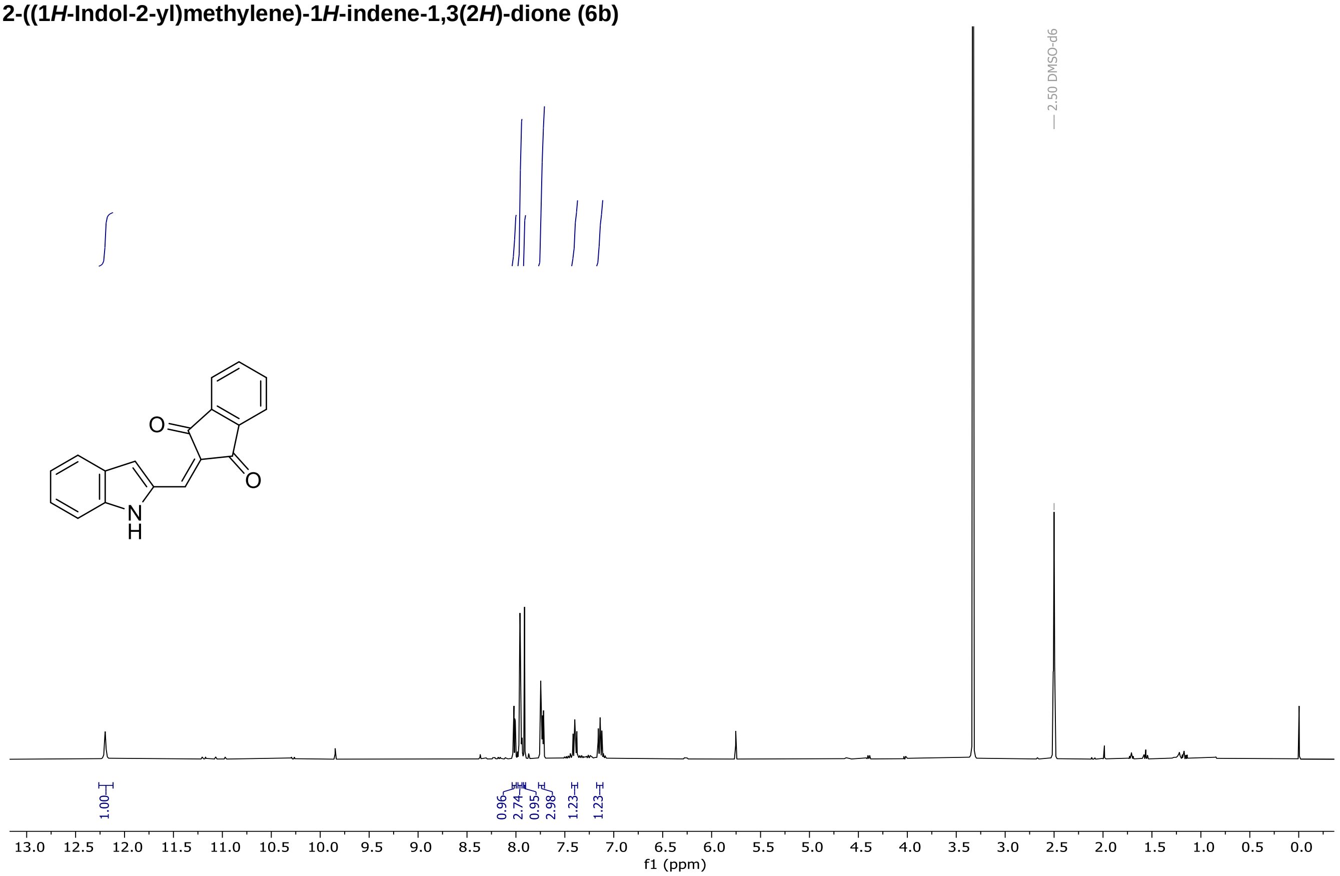

2-((1*H*-Indol-3-yl)methylene)-5,5-dimethylcyclohexane-1,3-dione (6c)

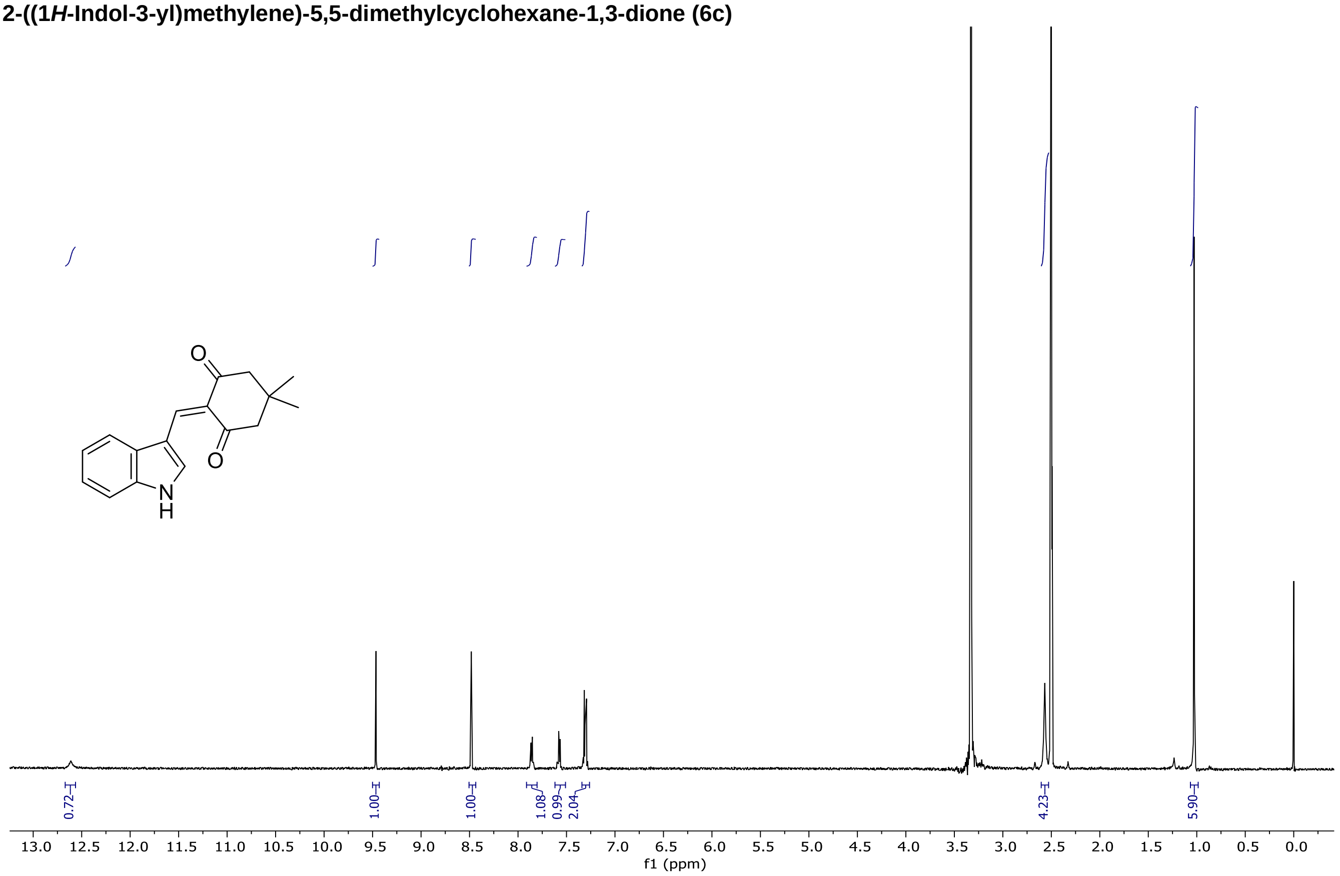

3-((1*H*-Indol-3-yl)methylene)chromane-2,4-dione (6d)

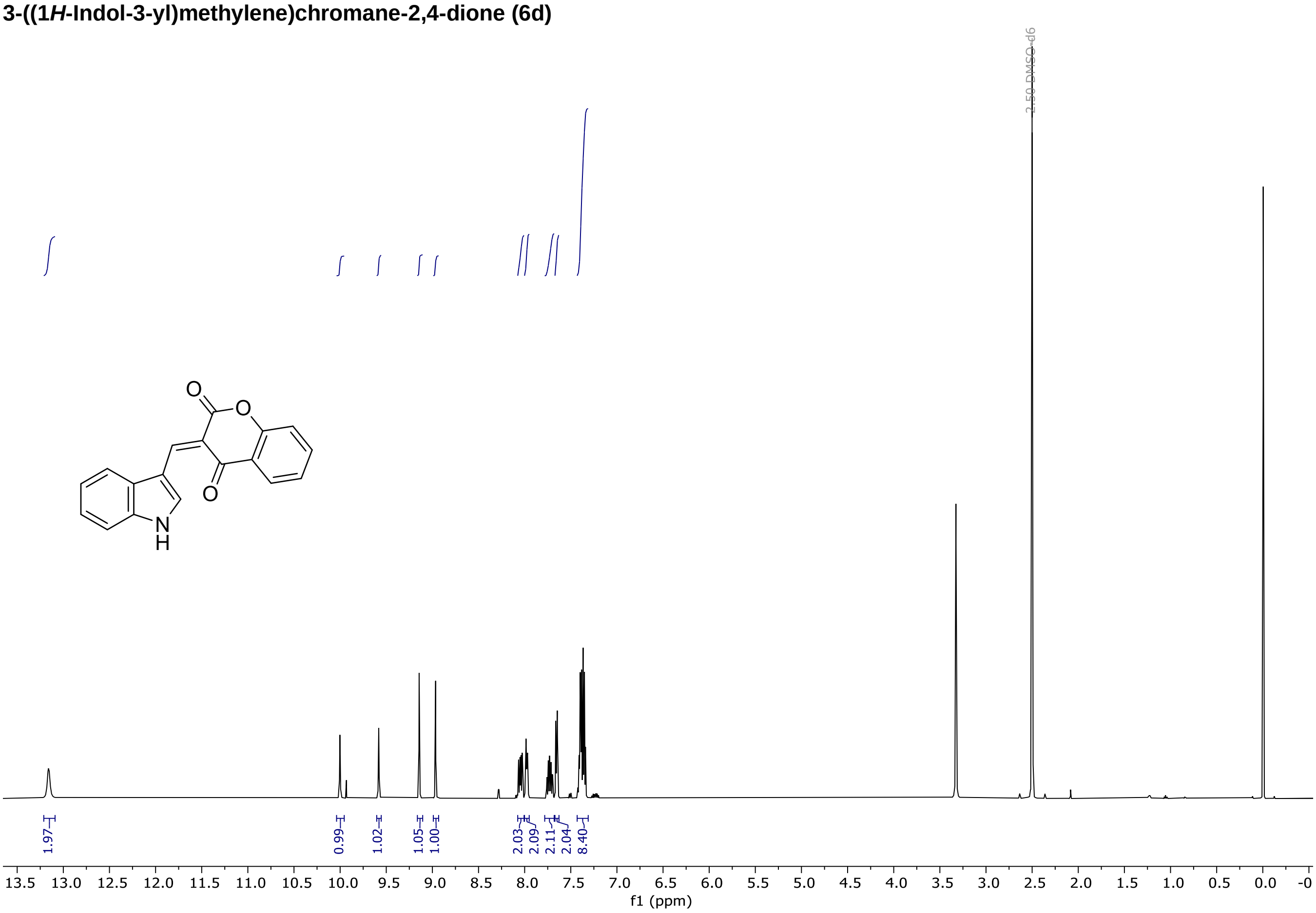

5-((1*H*-Indol-3-yl)methylene-1,3-dimethylpyrimidine-2,4,6(1*H*,3*H*,5*H*)-trione (6e)

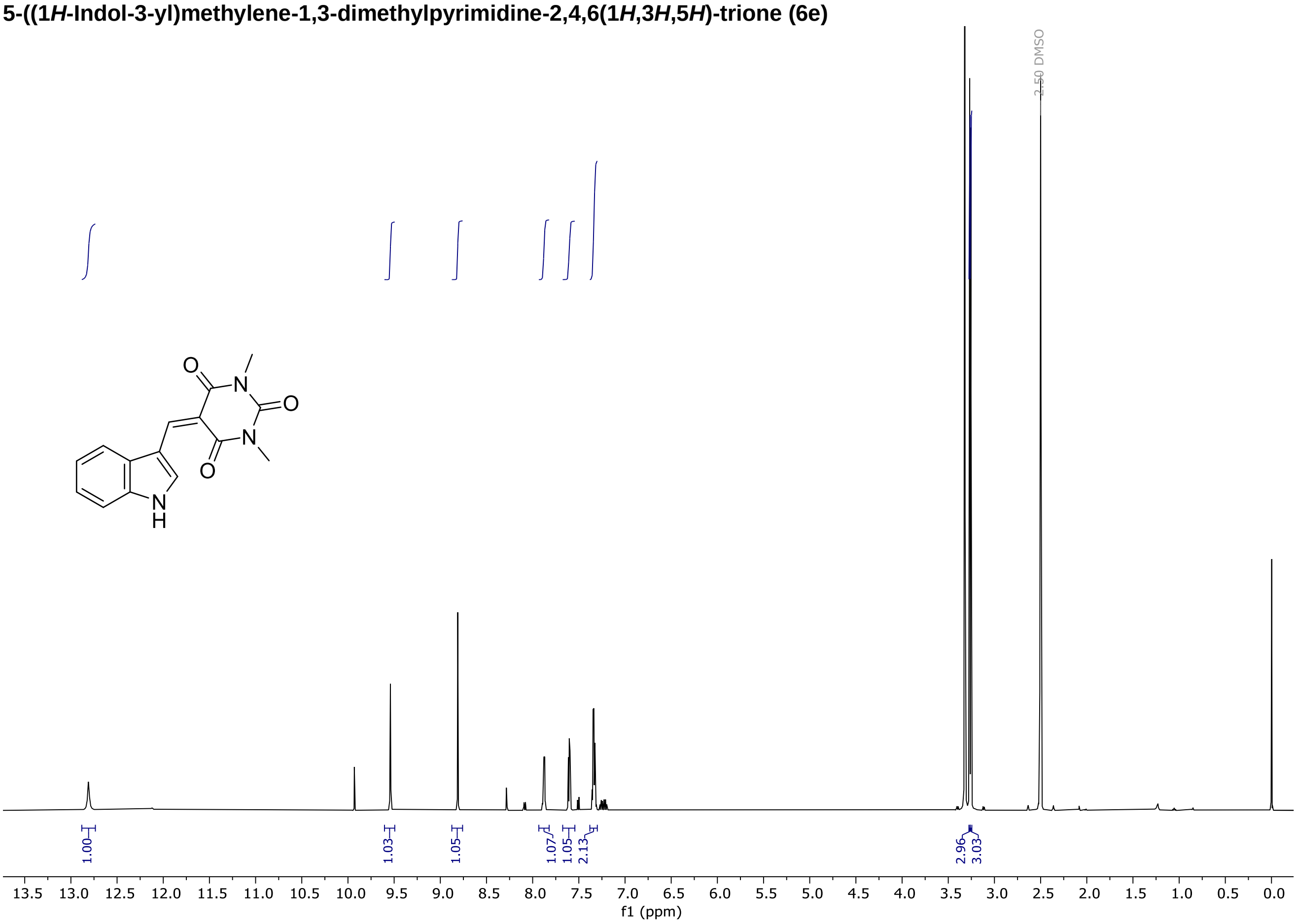

2-((1*H*-Indol-3-yl)methylene)-1*H*-indene-1,3(2*H*)-dione (6f)

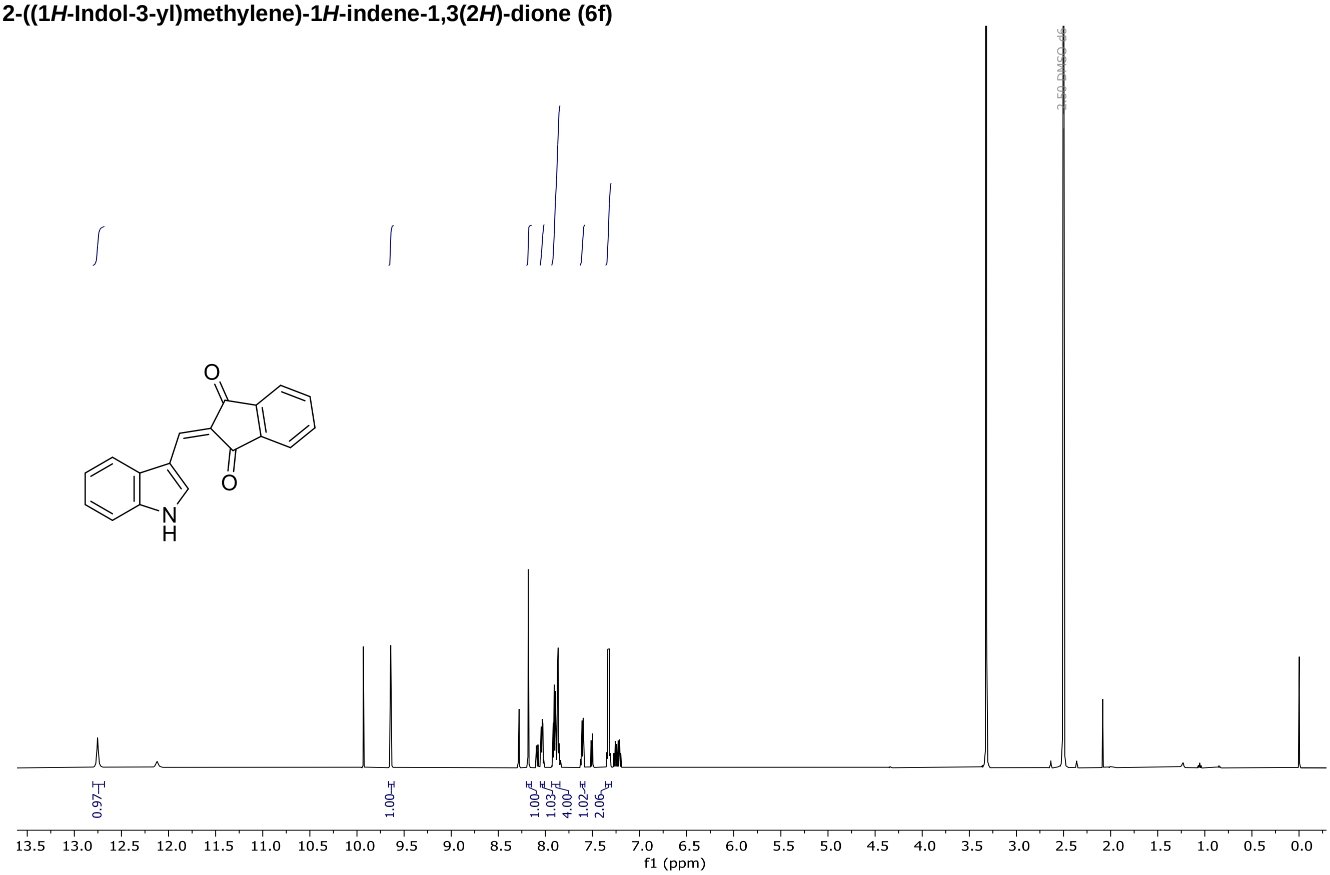

3,3'-((5-methoxy-1-(phenylsulfonyl)-1*H*-indol-2-yl)methylene)bis(2-methyl-1H-indole) (7a)

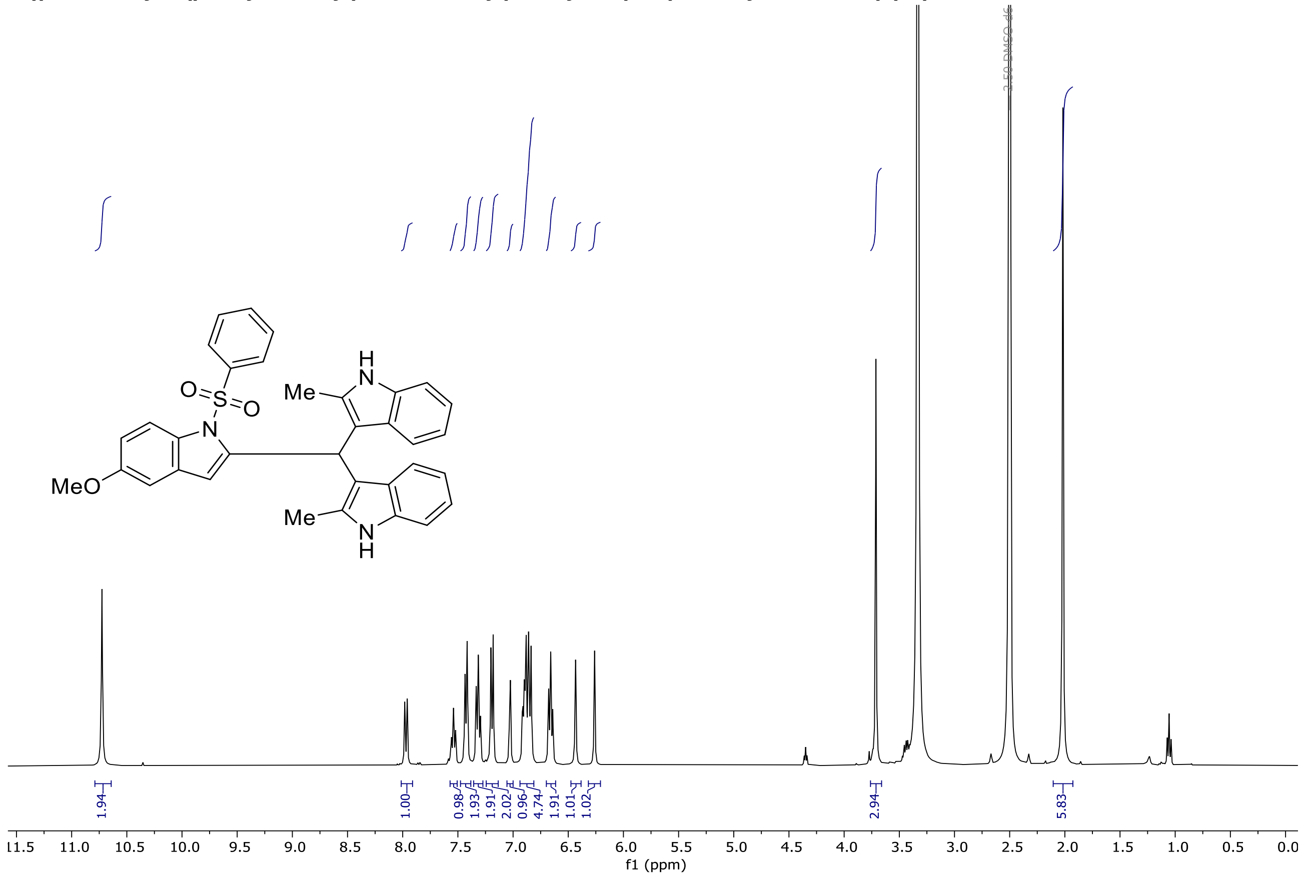

3,3'-((3,4,5-Trimethoxyphenyl)methylene)bis(2-methyl-1*H*-indole) (7b)

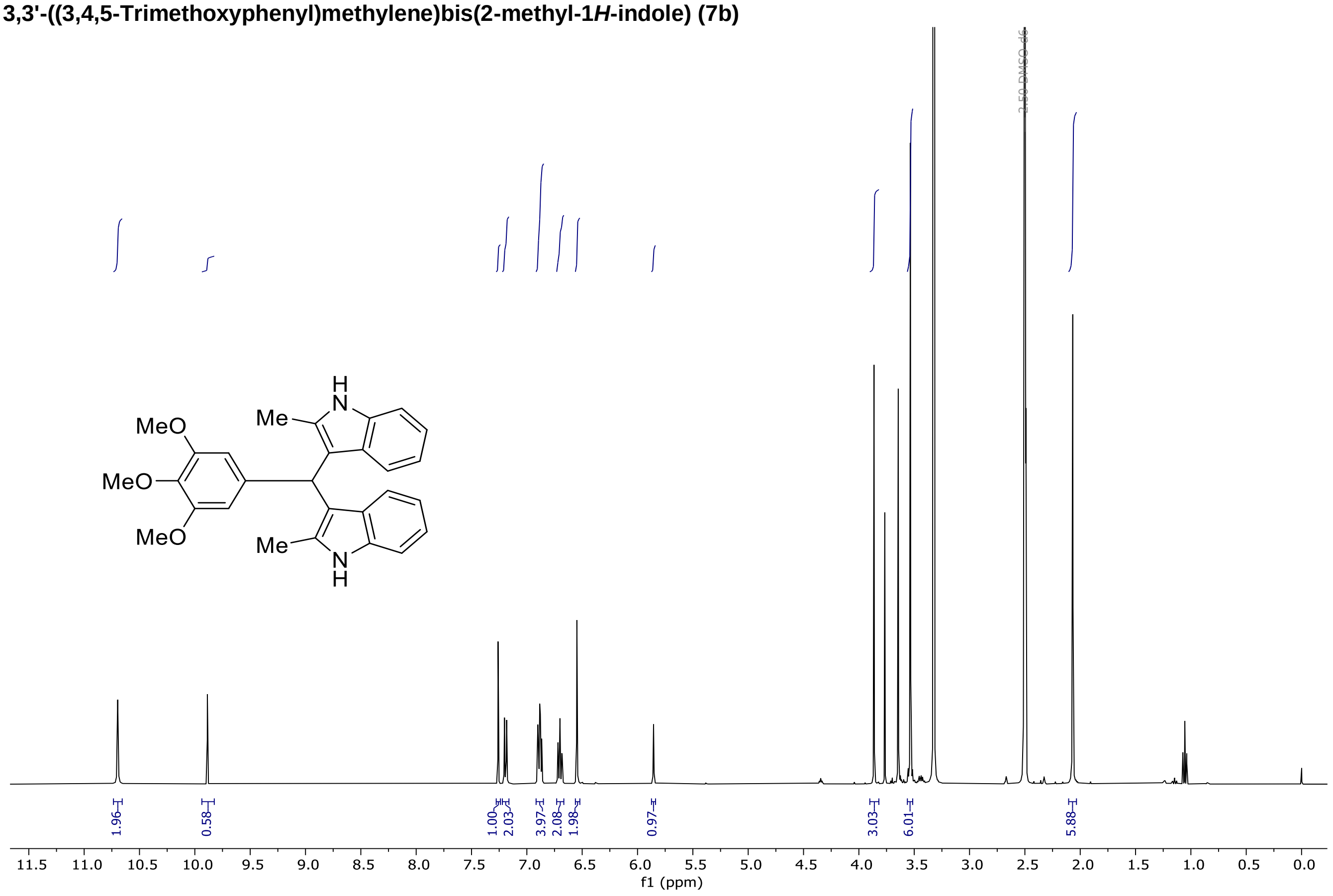

10.4. 6-Ethoxy-ICZ (8)

6-Ethoxy-5,11-dihydroindolo[3,2-*b*]carbazole (8)

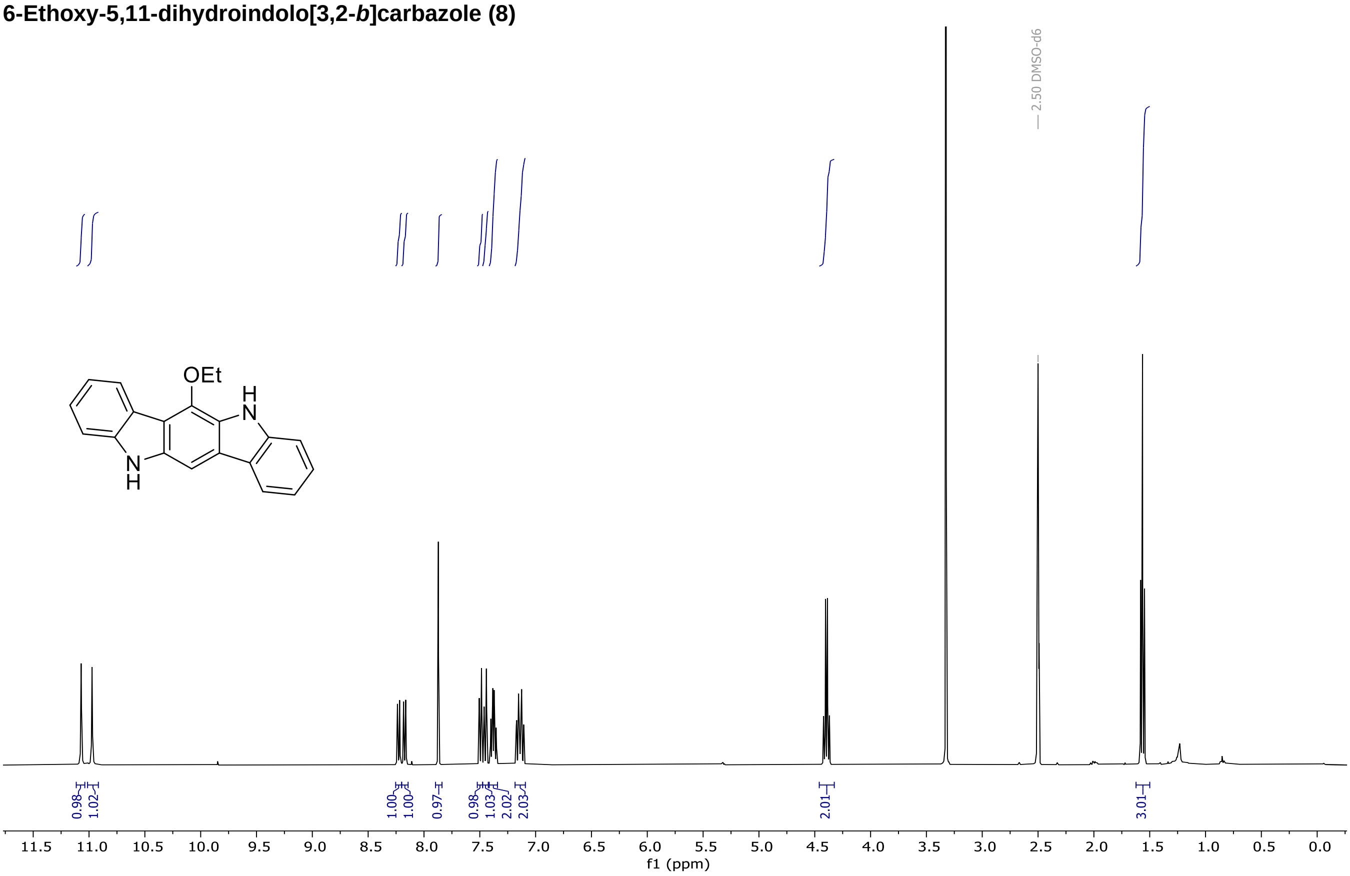

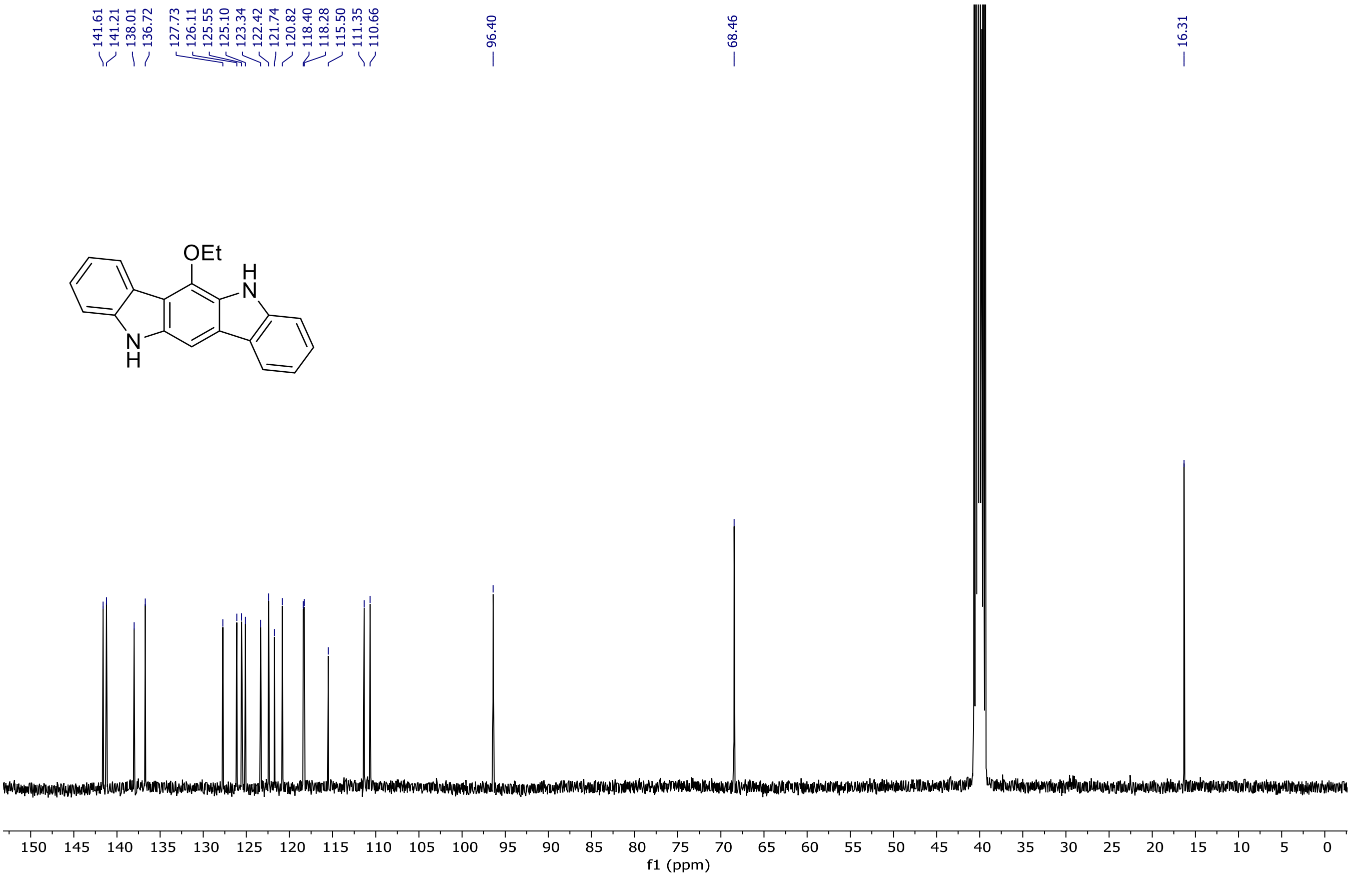

10.5. Post-transformation products (Series 9-11)

N-(4-(3-(5,11-Dihydroindolo[3,2-*b*]carbazol-6-yl)-1*H*-indol-5-yl)phenyl)methanesulfonamide (9a)

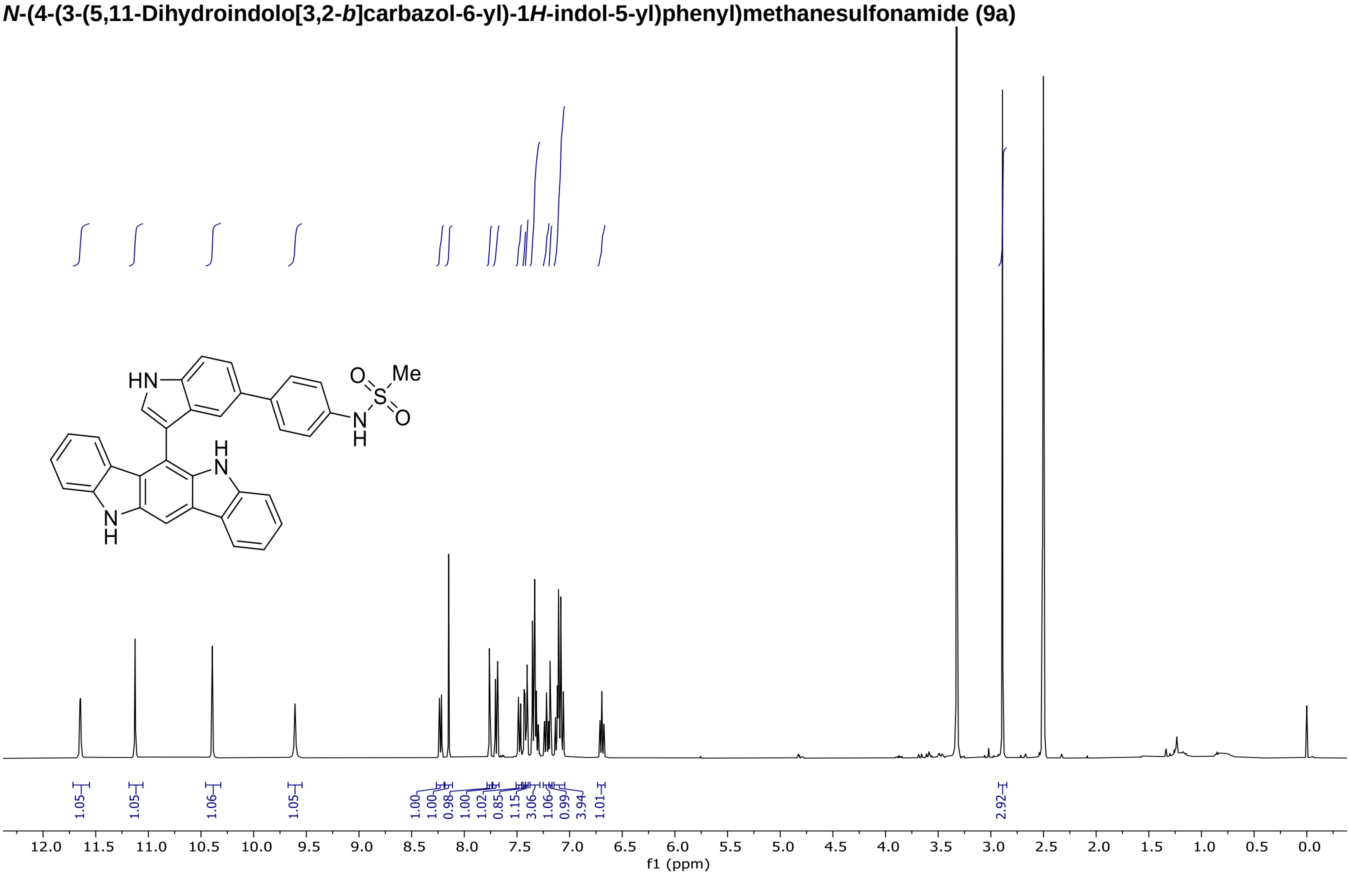

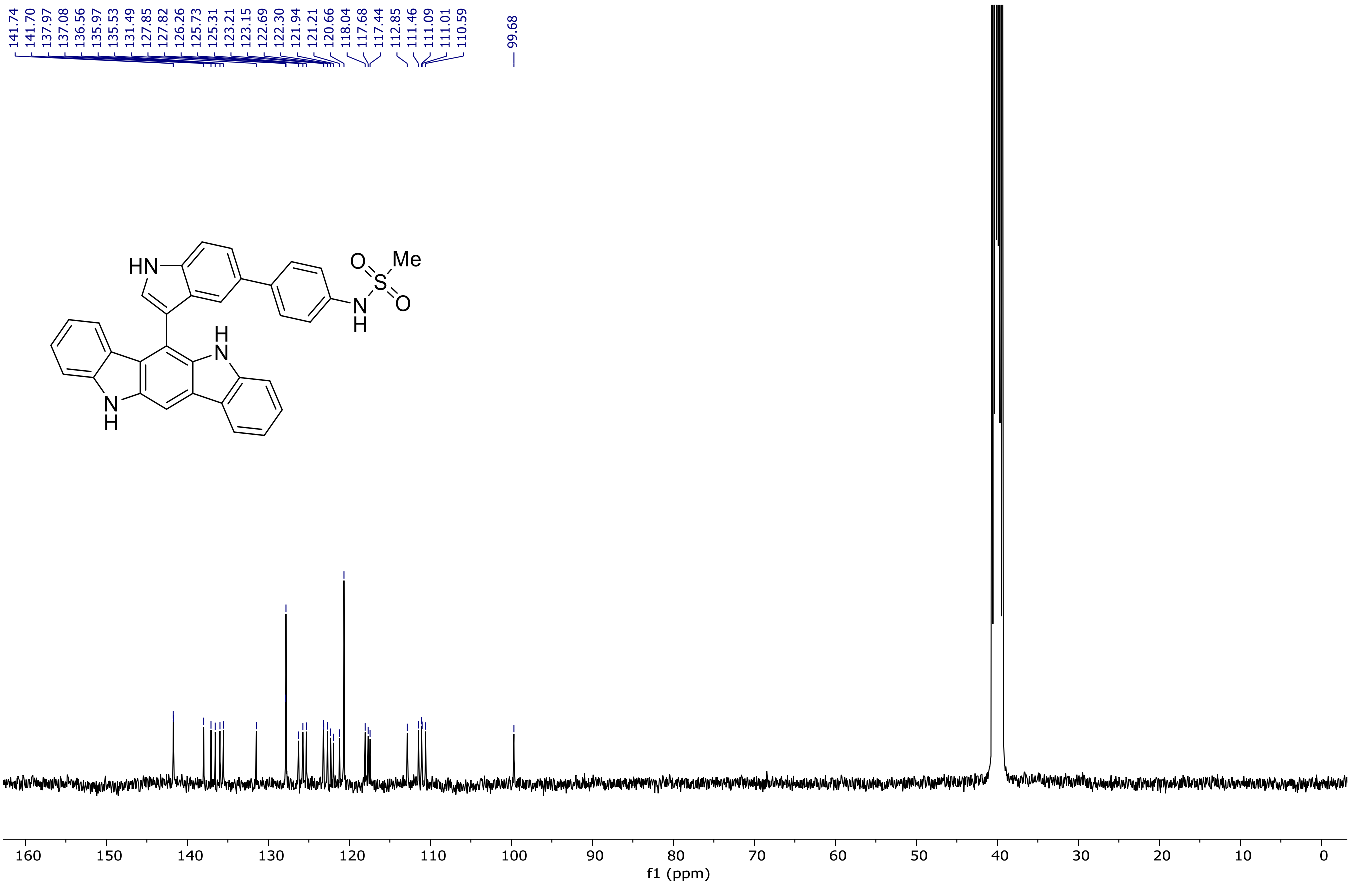

6-(5-(*p*-Tolylethynyl)-1*H*-indol-3-yl)-5,11-dihydroindolo[3,2-*b*]carbazole (9b)

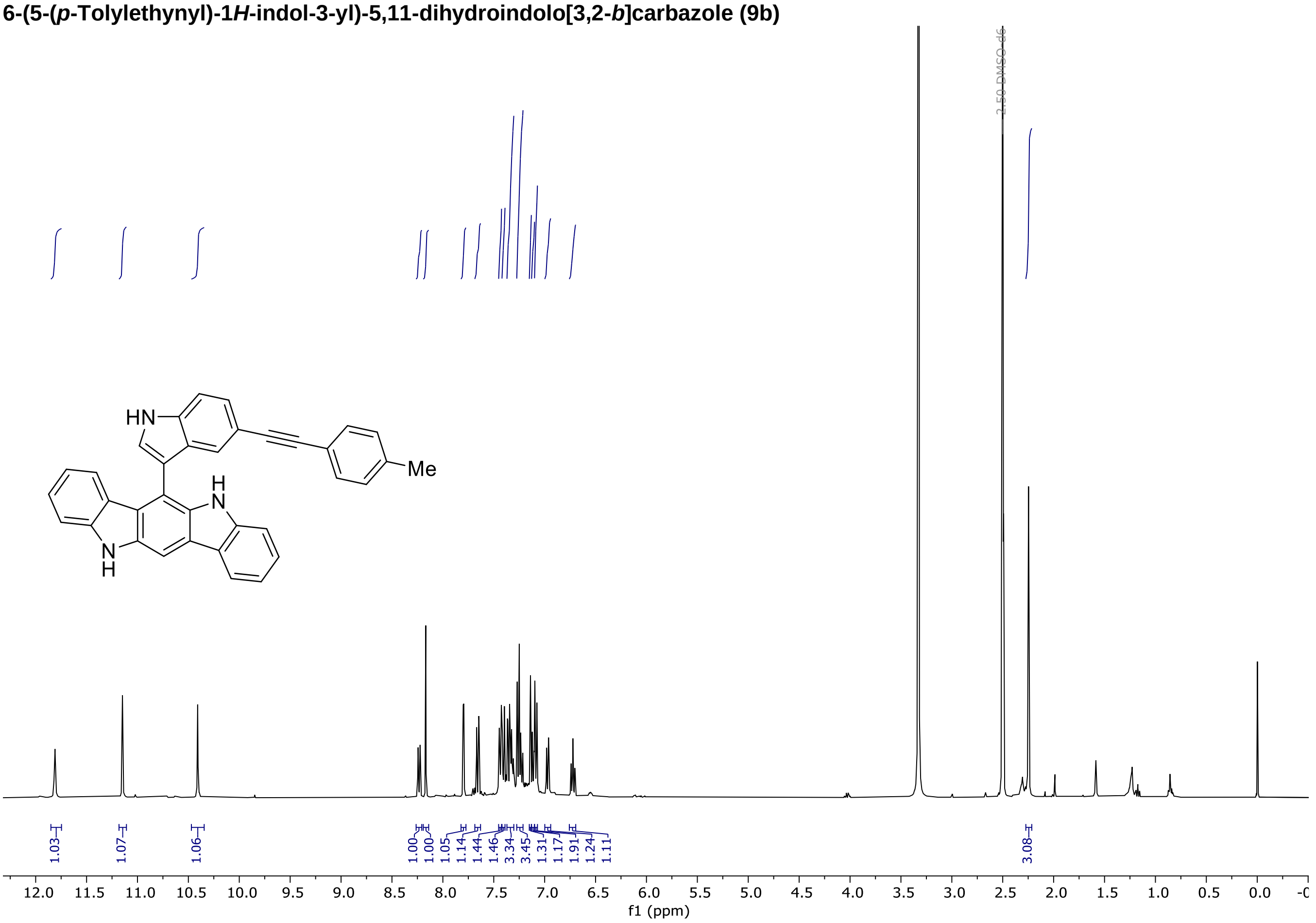

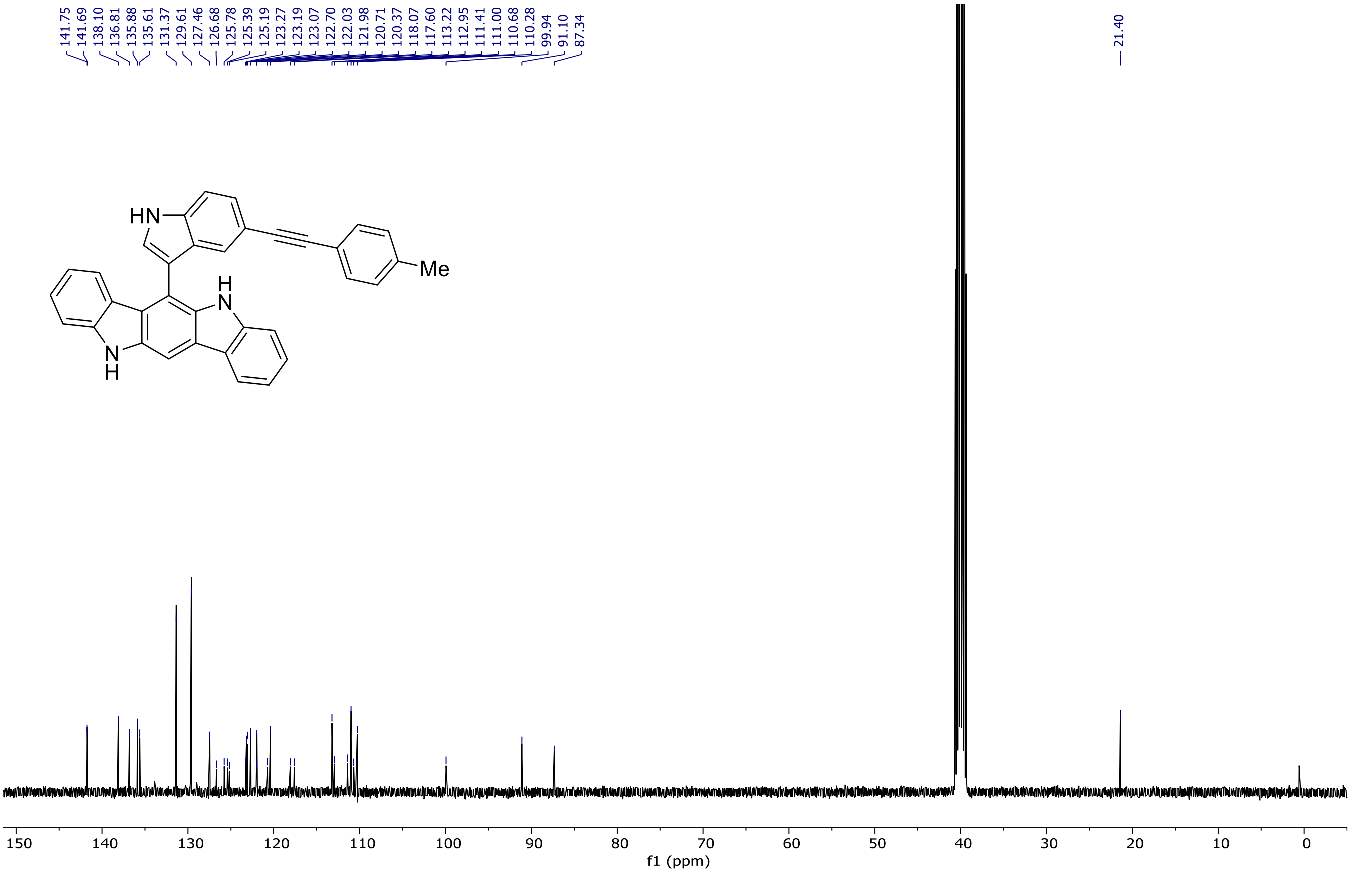

_N_-(2-(5,11-Dihydroindolo[3,2-_b_]carbazole-6-carbonyl)phenyl)acetamide (10)

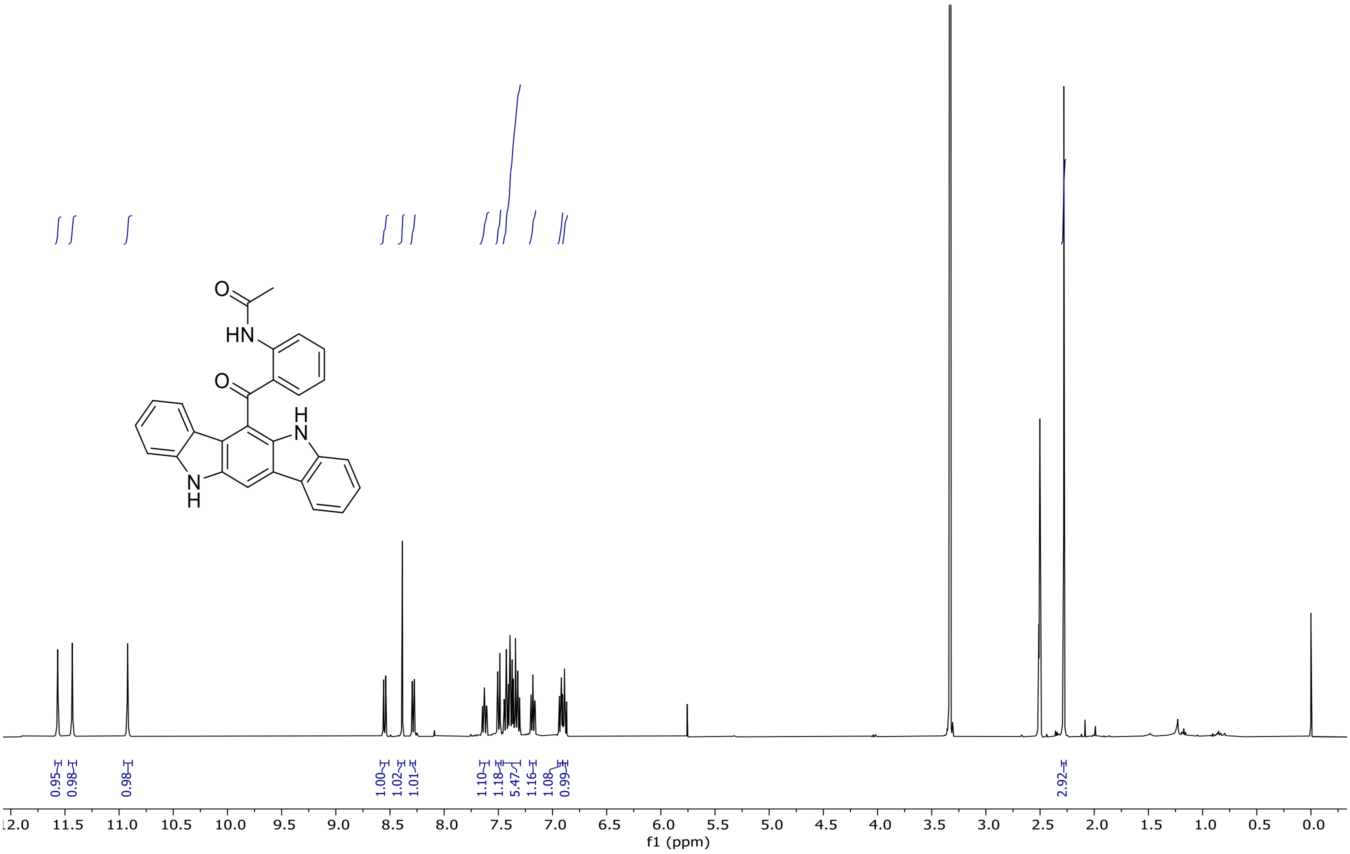

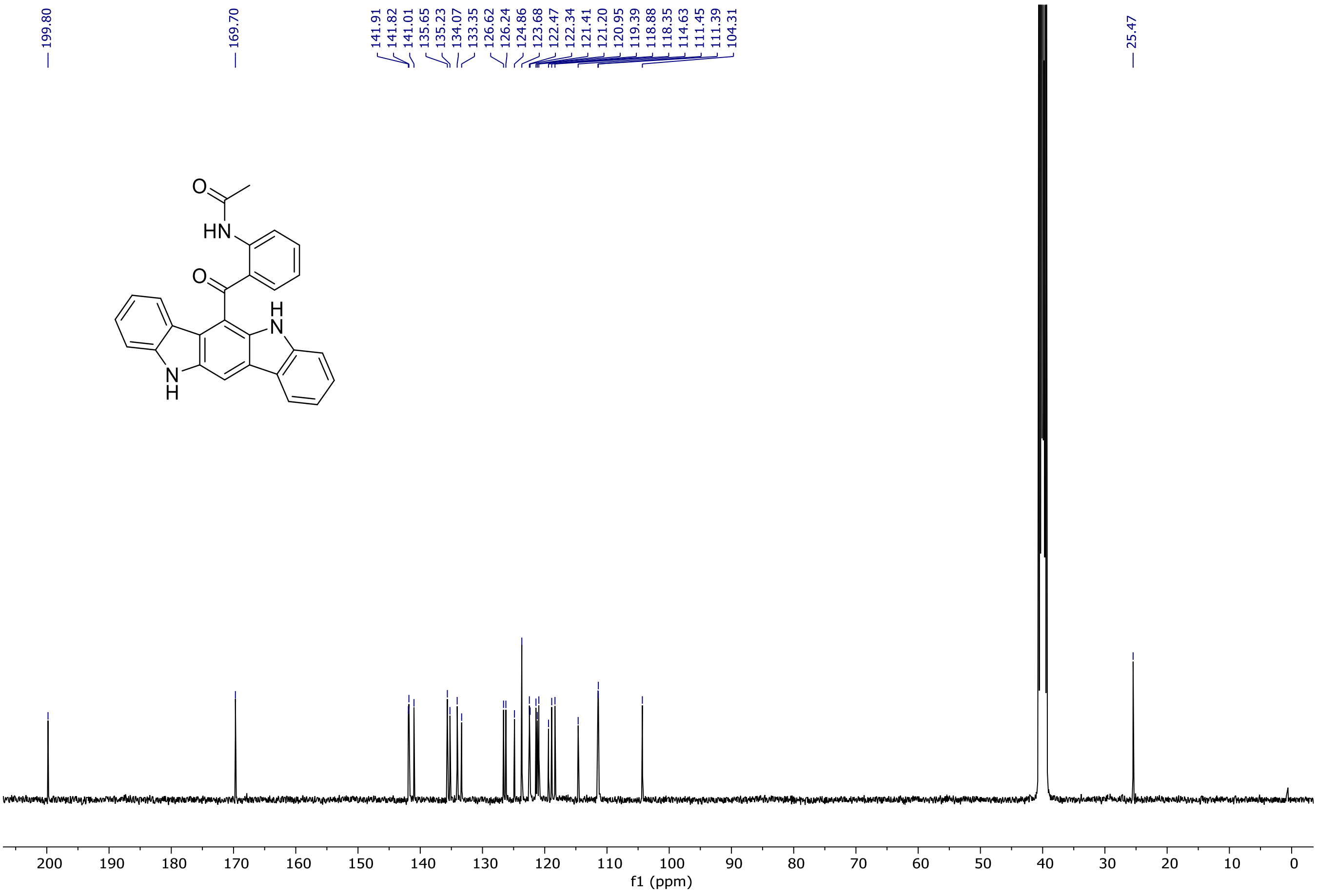

1-Acetyl-5'*H*-spiro[indoline-2,6'-indolo[3,2-*b*]carbazole]-3,12'(11'*H*)-dione (11)

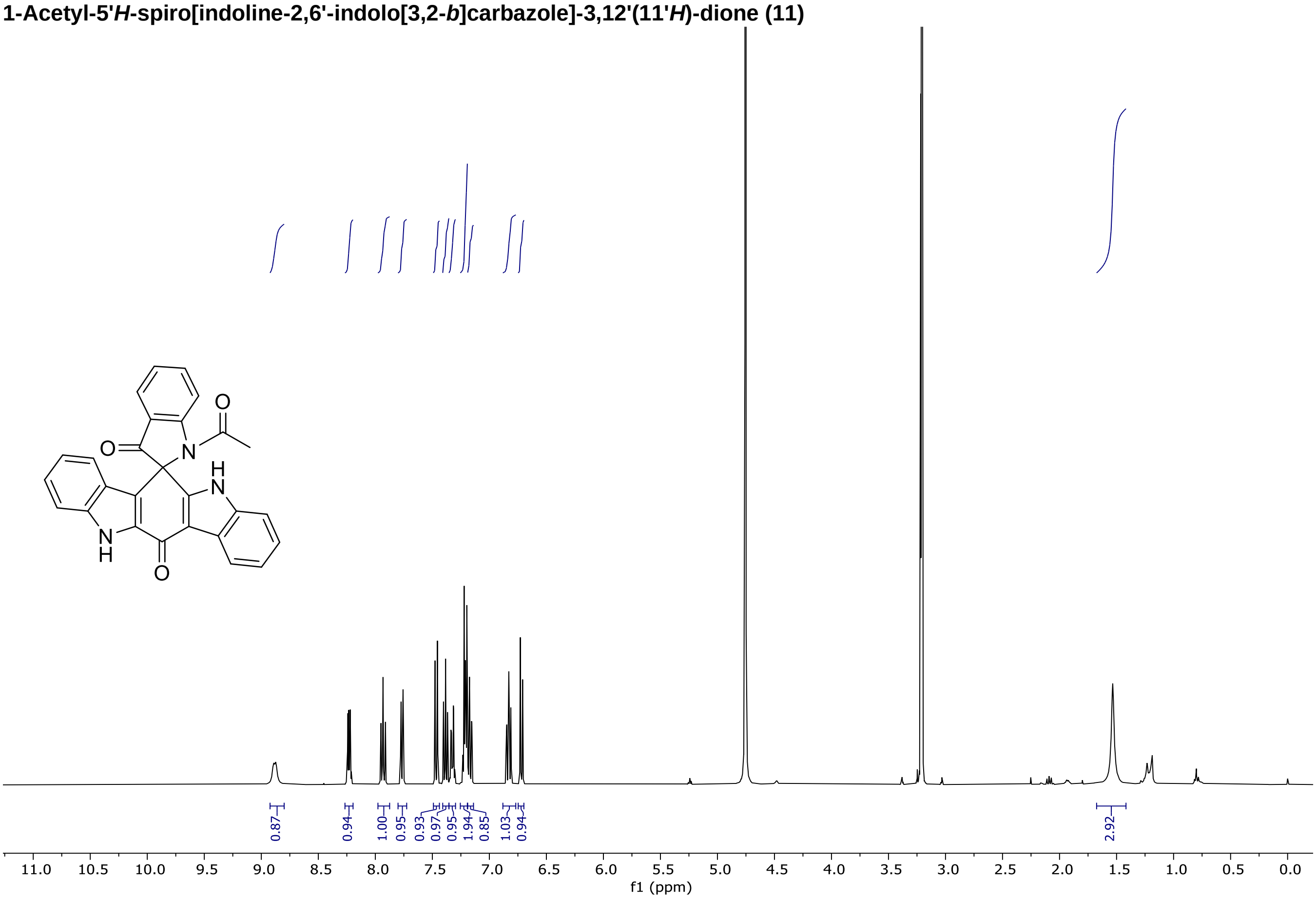

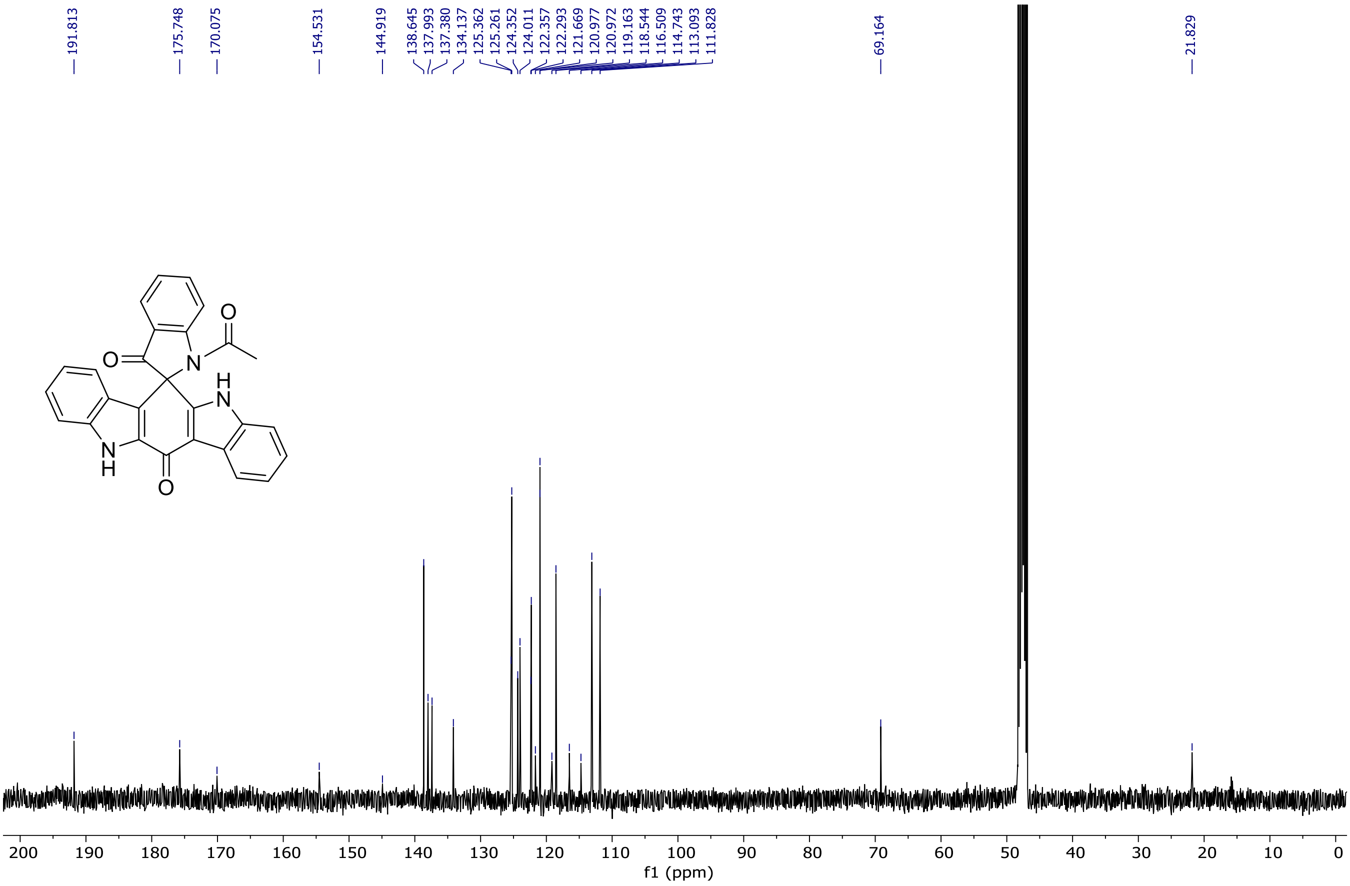

10.6. Conjuated 6-ICZs and bifunctional AhR probes (Series 12)

3-(5,11-Dihydroindolo[3,2-*b*]carbazole-6-yl)-*N*-pentyl-1*H*-indole-6-carboxamide (12a)

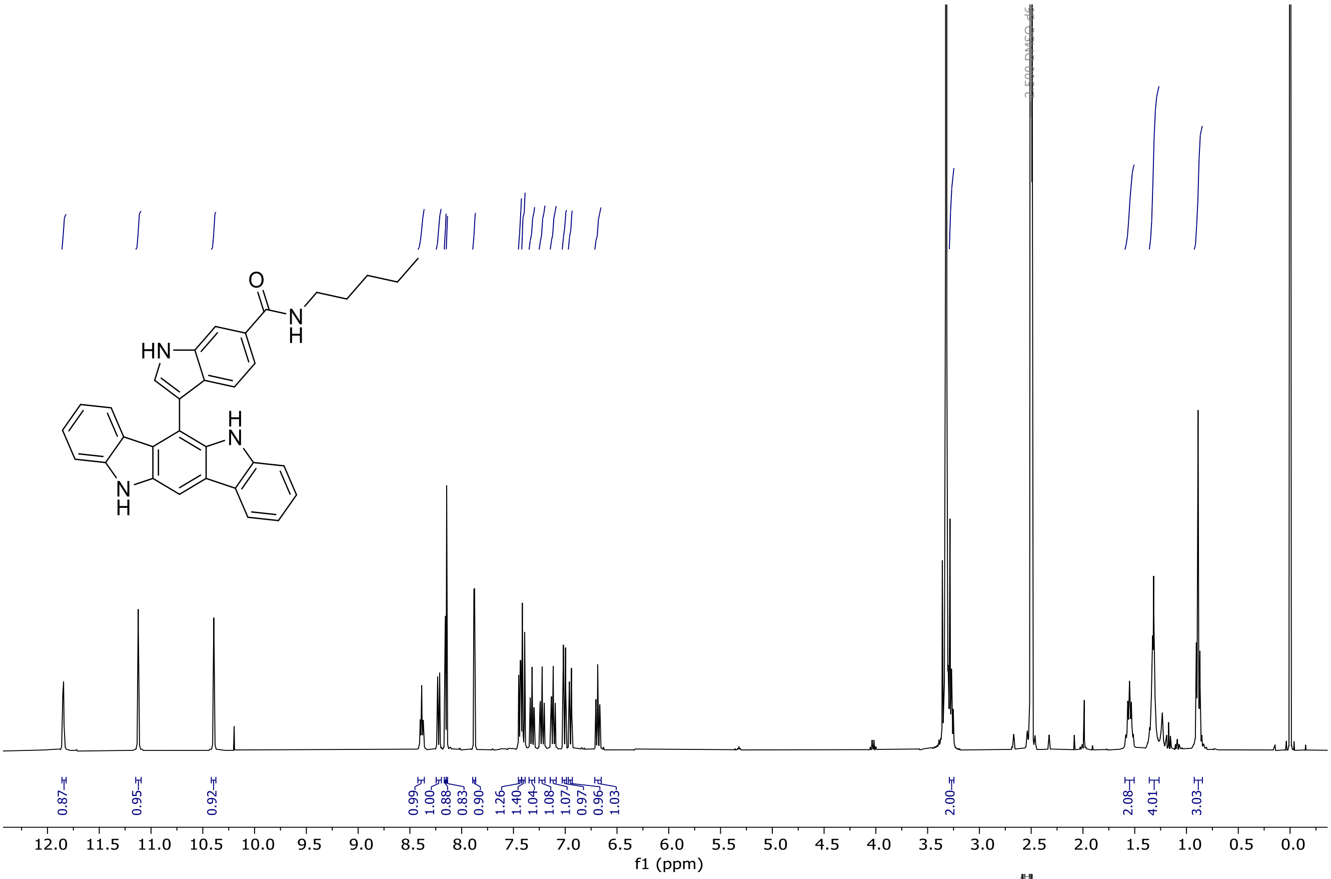

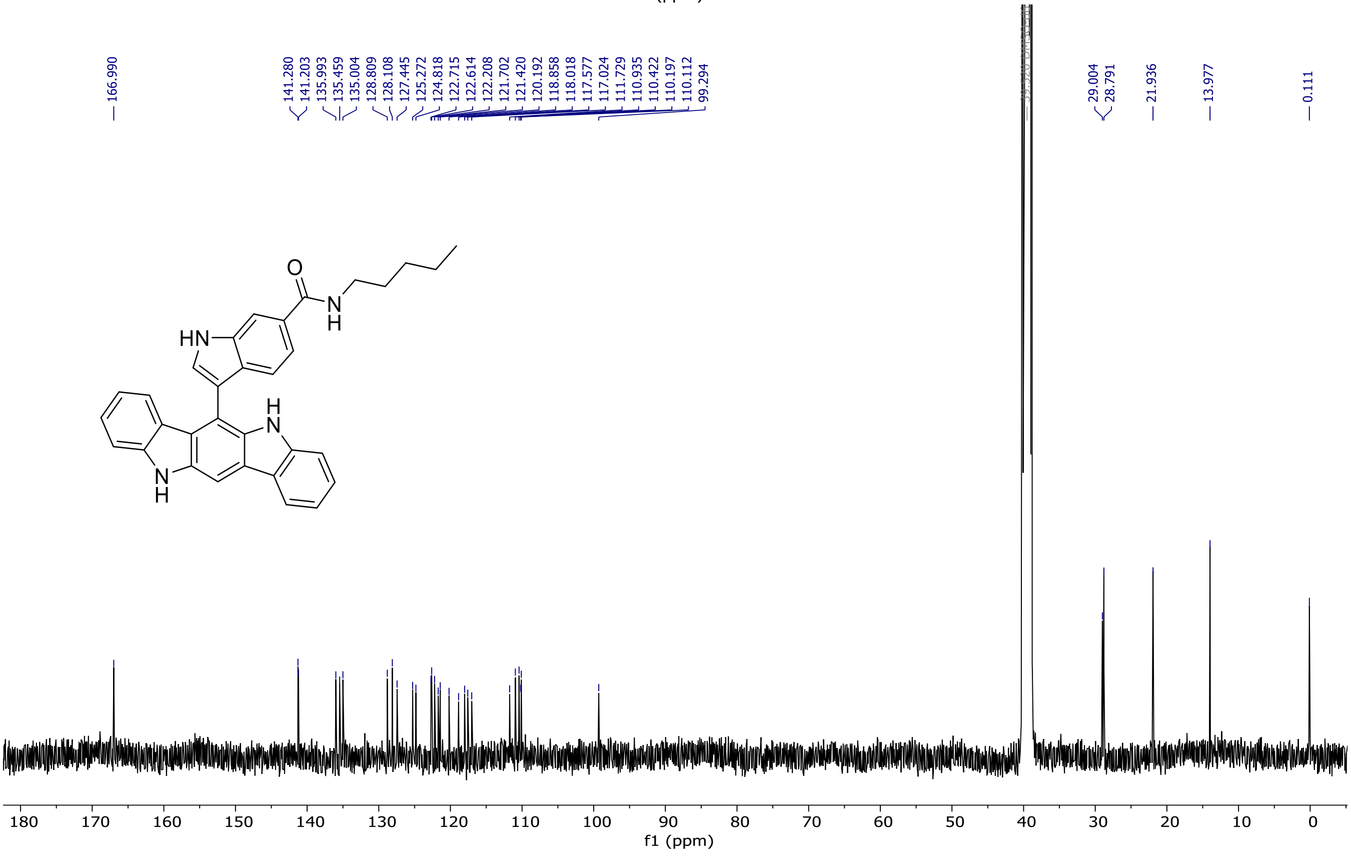

5m-PEG3-thalidomide (12b)

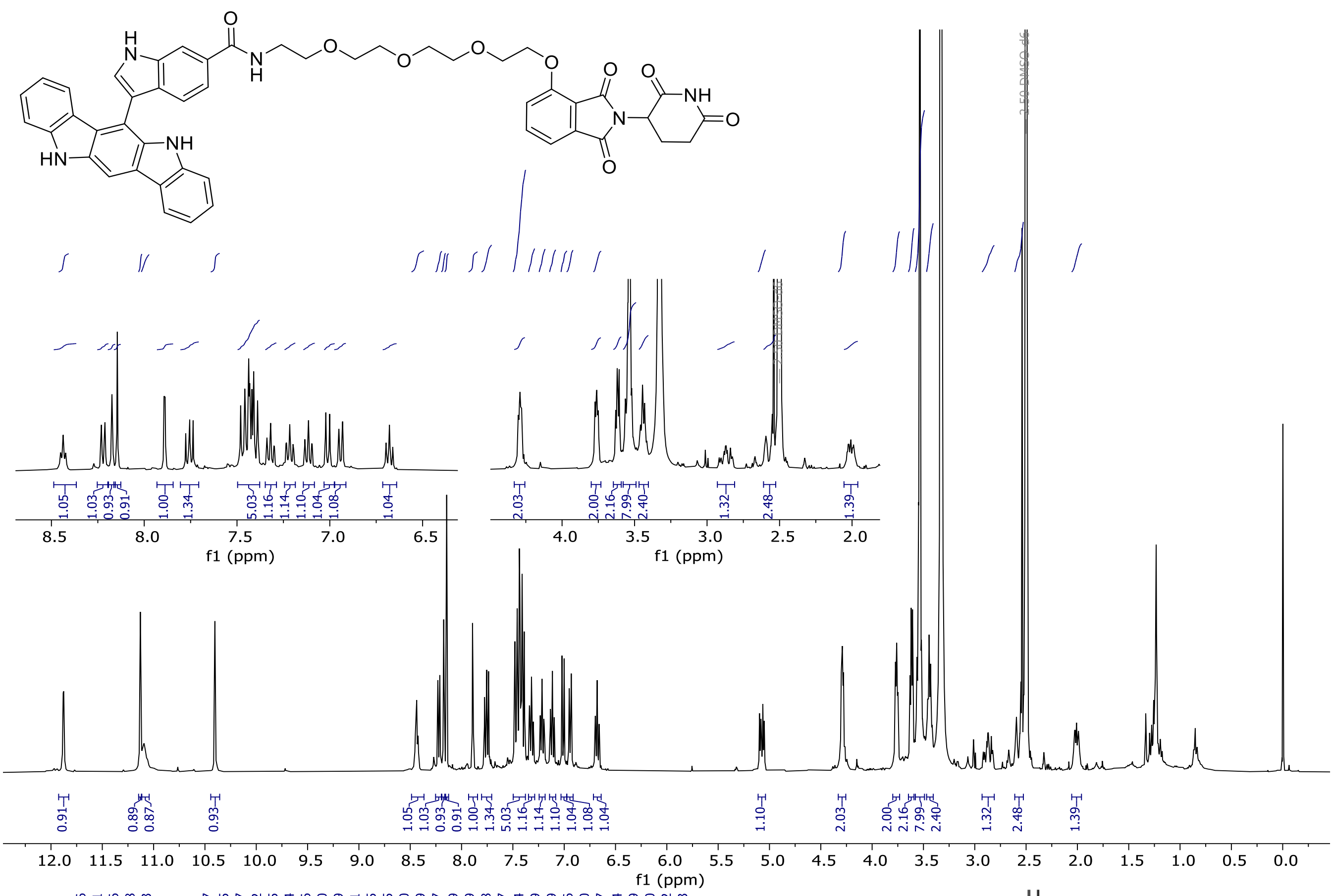

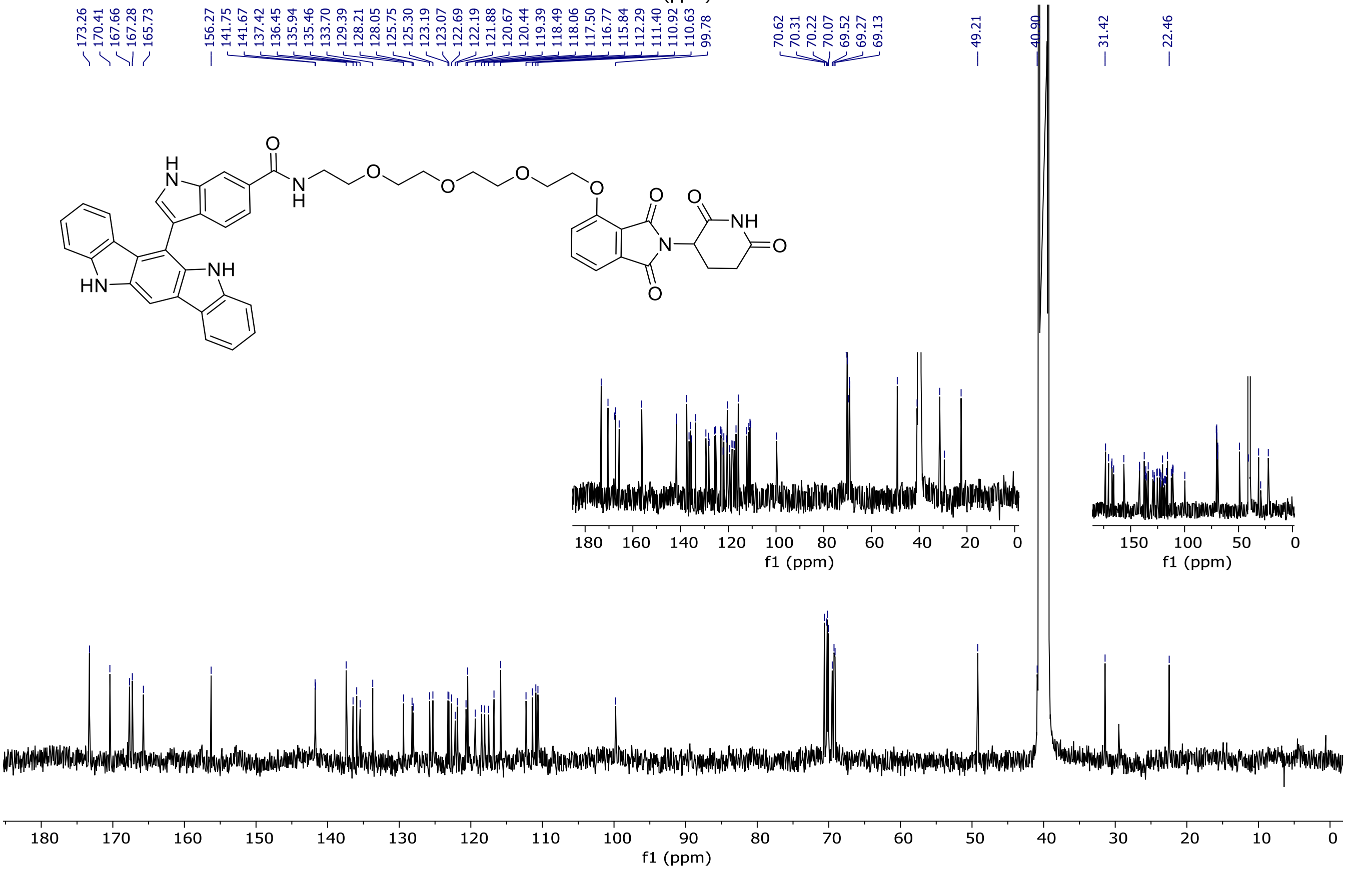

Bis-5m-diaminodecane (12c)

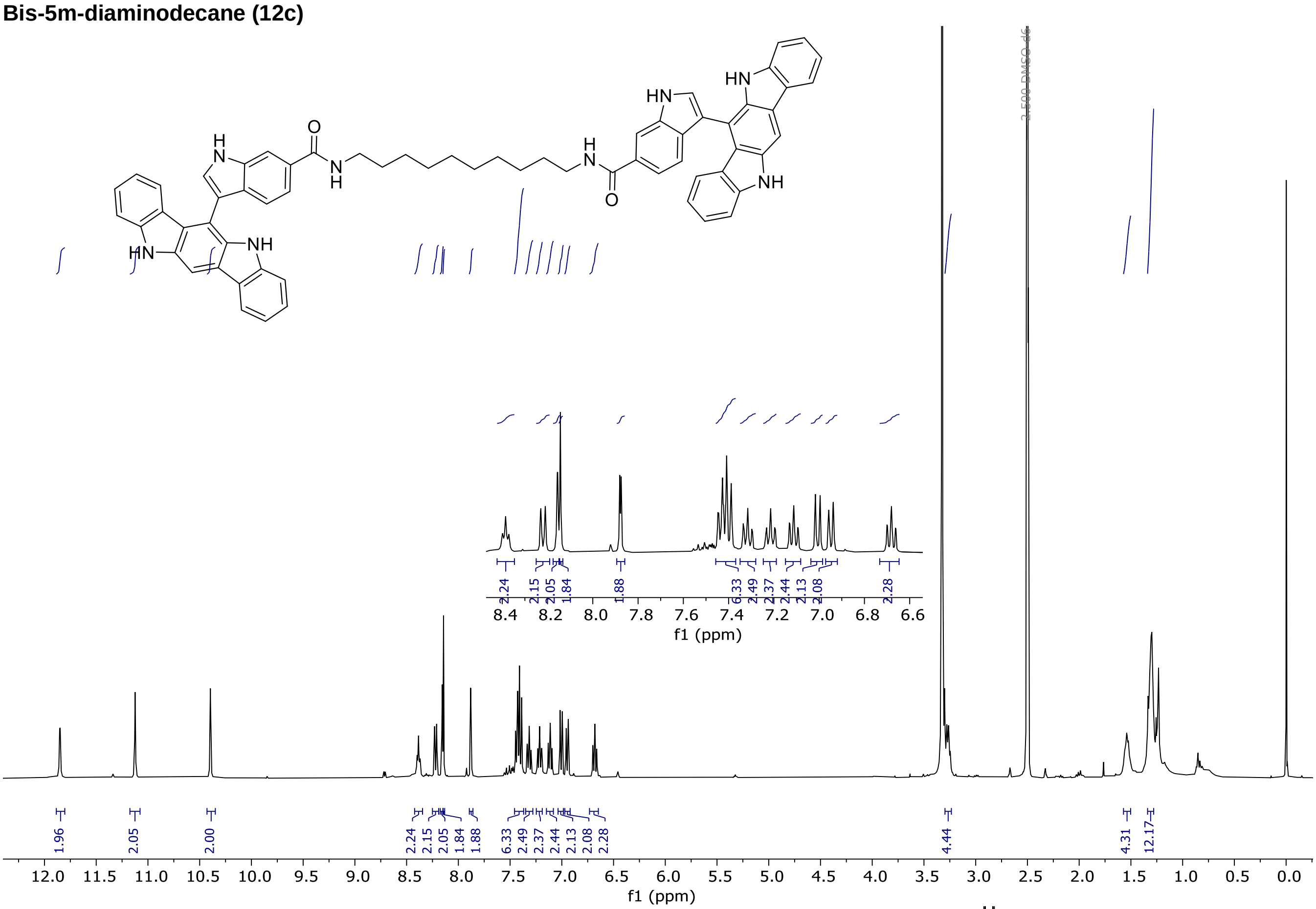

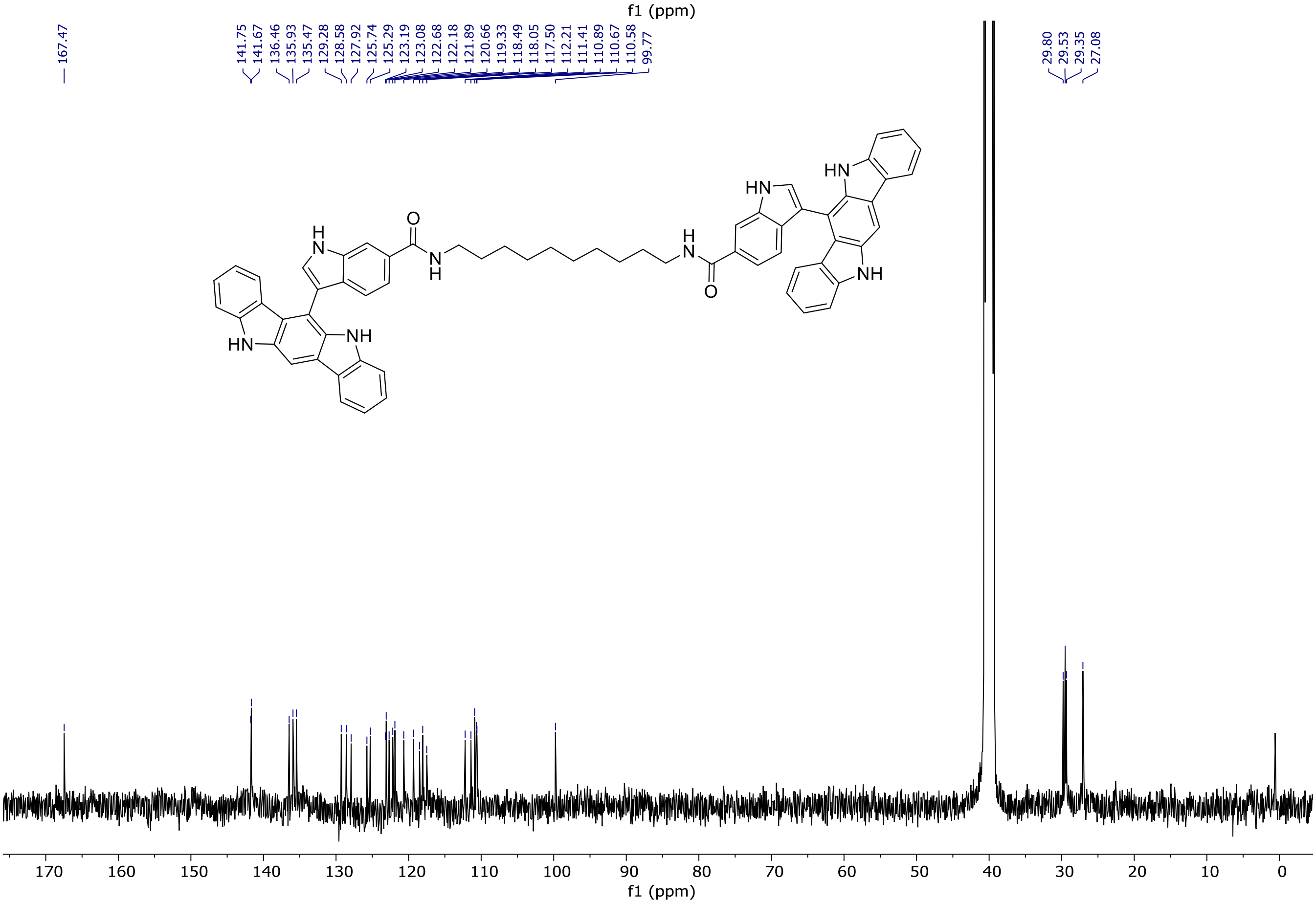

Bis-5m-PEG3 (12d)

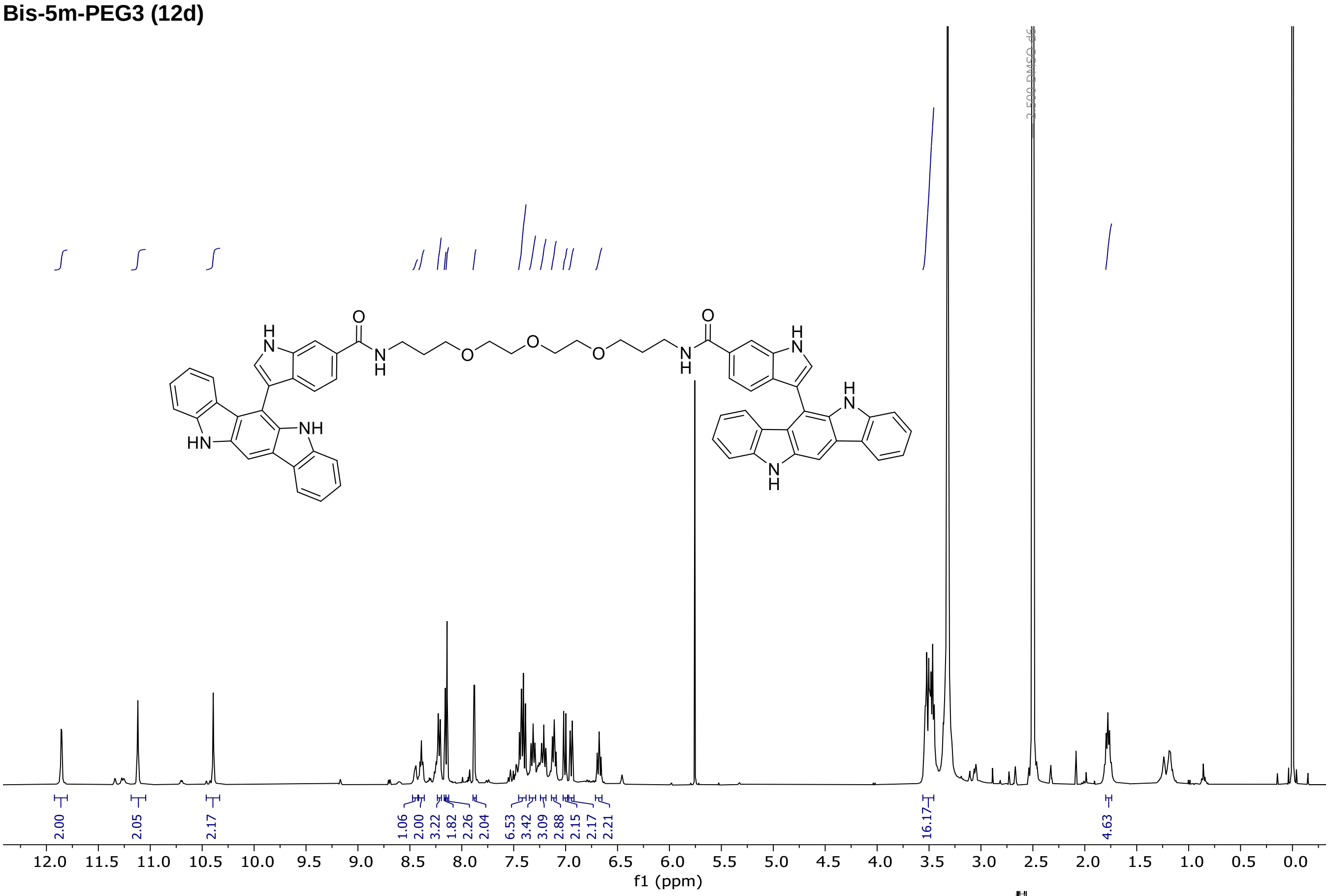

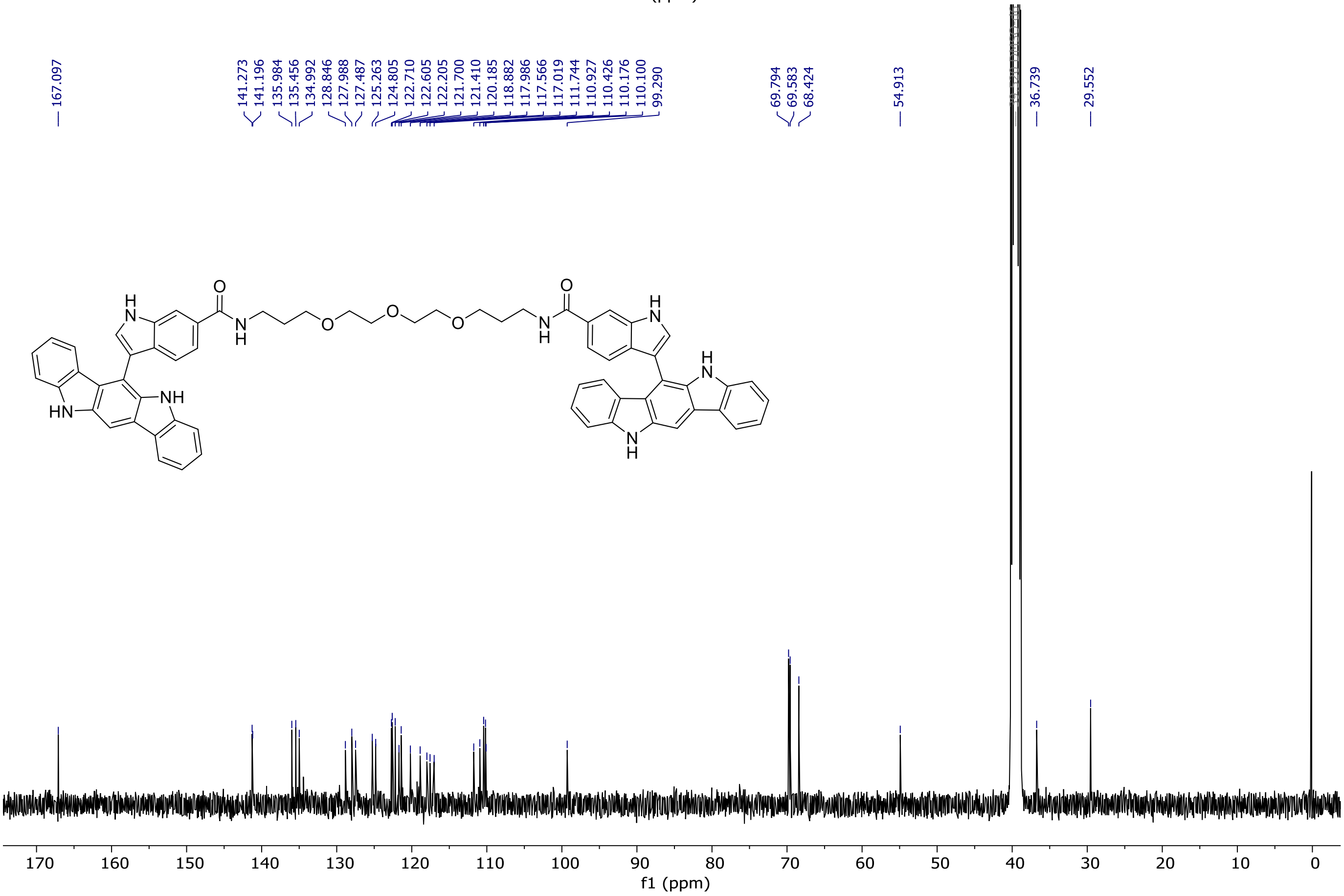

10.7. Compounds S

N-((1-cyclohexyl-1H-tetrazol-5-yl)(1H-indol-2-yl)methyl)-4-methylaniline (S1)

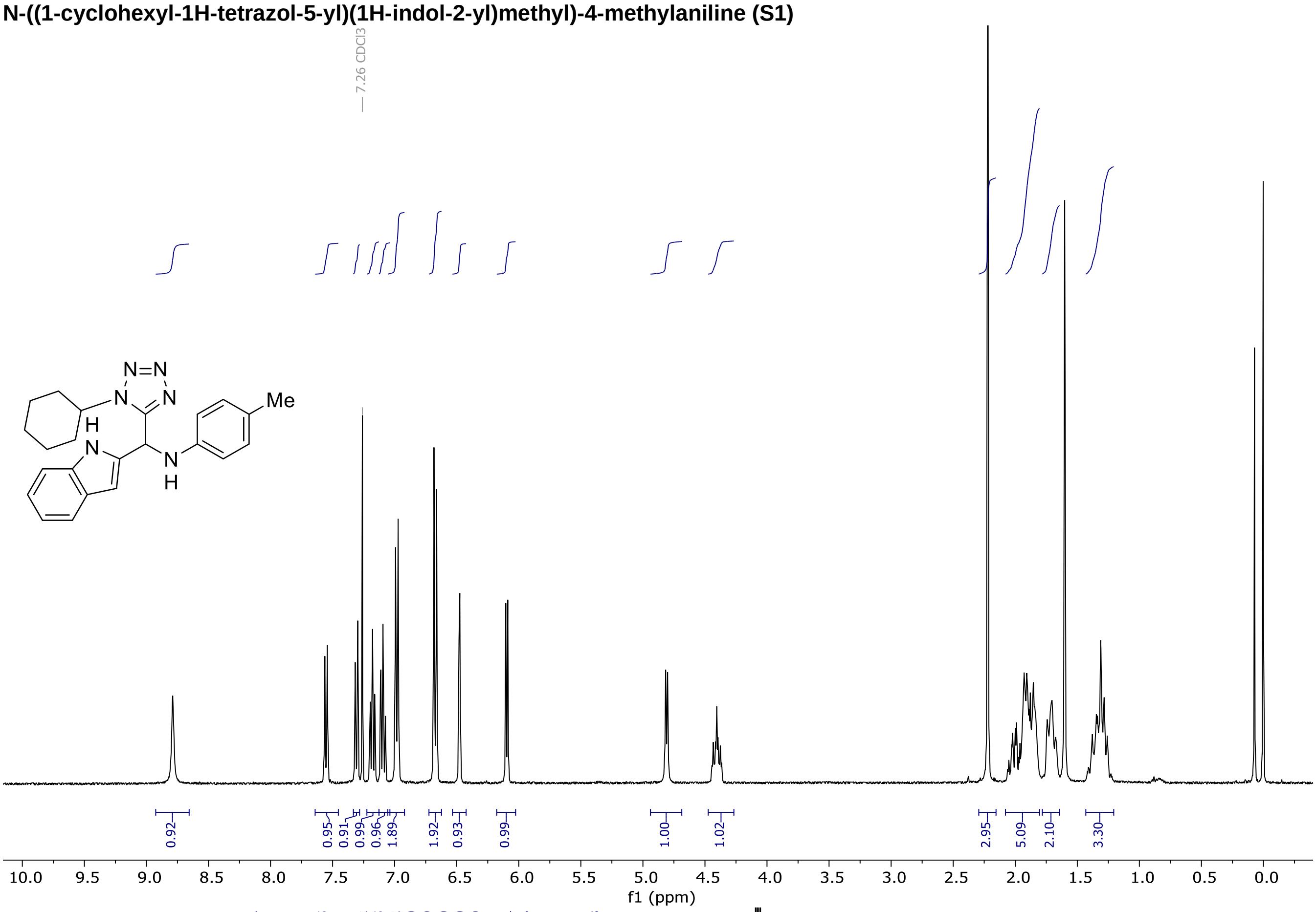

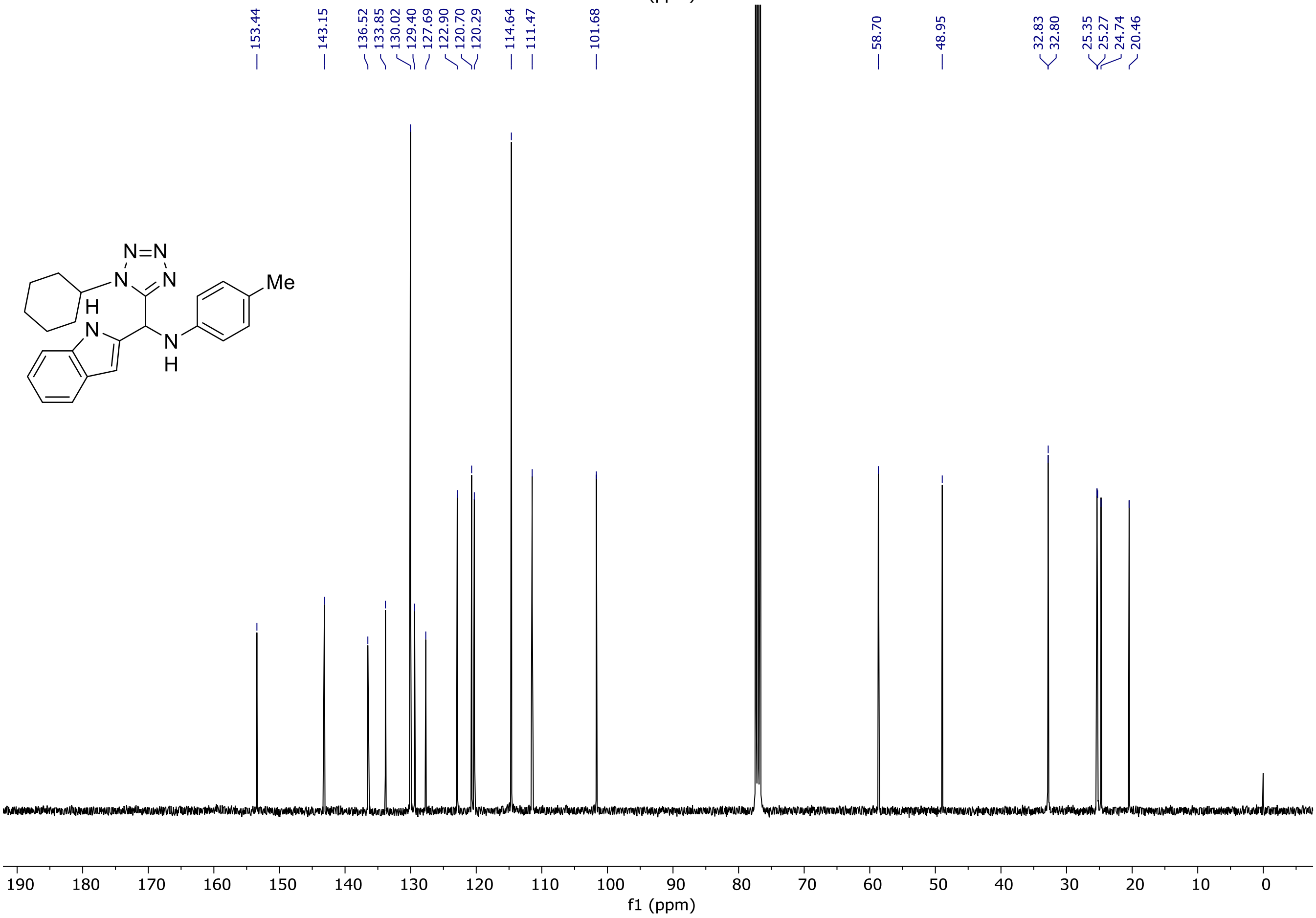

Supplement: Supplementary file 4 — oc5c00194_si_004.pdf [file oc5c00194_si_004.pdf]
