# Supplementary material for: A Multicomponent Reaction-Based Platform Opens New Avenues in Aryl Hydrocarbon Receptor Modulation
Source: ACS Cent Sci. 2025 Apr 10;11(4):629–41. doi: 10.1021/acscentsci.5c00194 (PMC12022909; doi:10.1021/acscentsci.5c00194)
Supplement: Supplementary file 5 — oc5c00194_si_005.pdf [file oc5c00194_si_005.pdf]

oc-2025-00194y.R1

Name: Peer Review Information for "A Multicomponent Reaction-Based Platform Opens New Avenues in Aryl Hydrocarbon Receptor Modulation"

First Round of Reviewer Comments

Reviewer: 1

Comments to the Author

The aryl hydrocarbon receptor (AHR) is a crucial chemosensory protein and an emerging therapeutic target for the treatment of various diseases, including cancers, metabolic disorders, and inflammatory conditions.

Ghashghaei and colleagues have conducted remarkable organic synthesis work to establish synthetic pathways for creating derivatives (referred to as 6-ICZ derivatives) of the natural AHR ligand (FICZ), utilizing a multicomponent reaction approach. These derivatives could lead to potential therapeutic molecules (activators, inhibitors or degraders) or pharmacological tools (e.g. fluorescent probes). They have used a multidisciplinary approach combining chemical synthesis, molecular modeling, docking, molecular dynamics simulations or cell-based assays to characterize the activity of their compounds.

The work was conducted in a thorough and highly rigorous manner. As a non-specialist in organic chemistry, I find the approach both original and promising for the development of new AHR receptor modulators. While the current work on bifunctional molecules has not yet led to the identification of effective inhibitors (such as degraders or antagonists), the protocols for synthesizing such molecules are established. Further research will be necessary to enhance the efficacy of the compounds, particularly by optimizing the length and composition of the linkers. On the other hand, one of the compounds obtained appears to have anti-inflammatory properties superior or at least equal to those of Tapinarof, the only FDA-approved AHR-based drug to date.

I have relatively minor comments:

- P2, L12-15: The sentence is unclear. What kind of screening is being referred to, experimental or in silico screening? Why is screening being contrasted with structure-guided rational design? It seems that both approaches are necessary and should be combined. When structural information on AHR is introduced at the start of a sentence, the authors should reference recent experimental studies instead of, or in addition to, modeling work.

- In several instances (P8, L59; P9, L9) the authors inappropriately mention X-ray structures instead of cryo-EM structures.

- P9, L5: the term "overlap" is confusing and appears not appropriate in the context in my view.

- For certain compounds (e.g., 5a, 5b, 5c, 5g, 5l), significant discrepancies are observed between their efficacy in reporter gene assays (Fig. S26) and their ability to induce endogenous gene expression (e.g., CYP1A1) (Fig. 5g). Do the authors have an explanation for this observation?

- P11, L19, 20: It's unclear why the authors associate the agonist properties of their compounds with the potential to create bifunctional AHR probes based on this activity. The key criterion in this case is receptor binding capacity, not whether the compounds are agonists or antagonists.

- Some of the newly synthesized ligands seem to behave more like partial agonists or even antagonists. This aspect is not addressed in the manuscript, and the compounds are not tested for their antagonistic activity. When combined with docking and MD simulations, this could have led to an interesting study on structure-function relationships. However, this may be beyond the scope of the current manuscript.

- P11, L32: The compounds discussed in the main text (12b-d) and in Figure 6f are different.

Reviewer: 2

Comments to the Author

In the submitted manuscript, Rodriguez et al. take inspiration form their recent work (ref 27) to develop a powerful Yonemitsu-type tricomponent reaction combining indole 2-carboxaldehydes with diverse nucleophiles to yield a series of 6-substituted indolocarbazoles. The chemistry work encompasses the reaction synthetic development, experimental reaction mechanism and computational structural studies, as well as post-functionalization of the primary adducts and extension of the reaction to several cellular probes. It is nicely completed with biological studies aiming at evaluating the activity of the compounds as aryl hydrocarbon receptor (AhR) agonists and proposed degraders with anti-inflammatory behavior through molecular docking and cellular experiments. The article is very clearly written and illustrated, making it particularly straightforward to read. The rational and relevance of the approach is well-exposed and easy to follow. It inspires several minor comments though.

The remark on the lack of reactivity of isomeric indole 3-CHO vs 2-CHO (p4, line 45) is surprising and one can wonder what was expected since the nucleophilic reactivity of the 3-position is hampered. The structure of the compounds expected from this indole 3-CHO precursor could thus be explained: how could it give compounds 4/5 as indicated in Fig S3 ? Regarding the proposed key intramolecular electrophilic cyclization step (conversion of intermediate II to II, Fig 2c), proceeding in acidic or basic medium. The Supp Info indicates "a neutral conjugated moiety suffering the attack of an ionized indole nucleophile" operating under basic conditions. The electrophilic species involved, potentially resulting from a dehydration, would gain to be indicated, at least in the Supp Info. The formation of a cationic intermediate may be considered in acidic medium and a prior report on a related process could be cited (Studies on the Acid-Catalyzed Dimerization of 2-Prenylindoles, Tetrahedron 1996, 52, 9455-9468, 10.1016/0040-4020(96)00482-6). However, how this cyclization gives rise in basic conditions to 6-ethoxy substituted compound 8 (Fig 2c), 6-unsubstituted ICZ (Fig S6d) and 5g,n-p (S9b), could be commented. Another point is the control experiments with preformed alcohol precursors Ia-d (Fig2d). They were found to evolve intermolecularly with a second equivalent of indole 2-CHO instead of cyclizing intramolecularly. However the relevance of these models may be discussed: the observed reaction pathway with such preformed carbinols may not be directly compared with that of

the standard one since the expected domino process have not been observed either under normal conditions with this substituents (H, Bu, allyl, 4-F-Ph).

The study of the atropoisomerism of compounds 4 and 5 is interesting. It should be noted however that the structure of conformer M2' in Fig4c is incorrect, the current structure actually represents M1, and not the enantiomer of M2, as it should. Besides, why not simply naming these two atropoisomers M2 and ent-M2 ?

Regarding the biological aspects, the docking experiments give an interesting view of the ligands binding mode and its similarity with that of the known binder indirubin. It is however not really convincing to explain the good activity of the N,N'-Me analogues 4d and 5j since a key interaction with Q383 is lost. A data that is puzzling is the very good activity of the 6-ethoxy substituted compound 8, suggesting that the indolyl moiety is not significantly contributing to the activity profile, while reinforcing the similarity with indirubin. In this regard, structural variations of 8 using substituted alcohols would be welcome. Besides, although molecular dynamics does not indicate a clear a binding gap between enantiomeric M2 and M2' species, it would be interesting to chromatographically resolve the two atropoisomers of 5e and test them separately. Enantiomeric identity of drugs can have a favorable impact not only of target engagement but also on their overall pharmacological profile.

The design of the probes is also interesting. The activity retained by the model compound 12a is however surprising since one could have expected a unfavorable effect of the lipophilic appendage exposed to the water environment. The rational for inactivating the protein by inducing its dimerization should be substantiated by literature references and the effect of 12c,d commented. One can have a naive comment regarding the relevance of the PROTAC strategy: if the natural destiny of Ahr is to be degraded after its activation (Fig1a), how to distinguish between normal versus PROTAC-induced Ahr degradation, in particular when no ligase seem to be clearly identify. This would incidentally also explain the degrading activity of the dimeric compounds 12c,d that may (solely) act as multivalent binders ?

Overall, the submitted article is particularly dense in hypotheses and design as well experimental and theoretical results yielding uncommon molecules with promising biological profiles (although water solubility and metabolic stability issues should be considered for further development) toward a validated target protein. It deserves to be published in ACS Central Science after consideration of the above mentioned minor comments.

Reviewer: 3

Comments to the Author

This manuscript describes the development of a rewired Yonemitsu multicomponent reaction to afford tunable 6-substituted indolocarbazole derivatives as potent AhR modulators with promising anti-inflammatory properties. Systematic characterization of the scope and mechanism of the rewired Yonemitsu MCR was performed, further structural modification also enriched the chemical diversity of the 6-ICZ derivatives. Preliminary biological evaluation identified favorable safety and anti-inflammatory property of compound 8. However, there are several issues need to be illustrated before ready for publication.

Firstly, several bifunctional AhR probes are presented in Figure 6, exhibiting comparable AhR downregulation effect to compound 8, however, their anti-inflammatory properties have not been evaluated. Is there any special application for these probes? The purpose of this modification strategy is not clear enough. Please comment on this.

Secondly, additional biological comparison with FICZ is recommended to highlight the bio-safety of its derivatives in this study. Moreover, current manuscript presents limited therapeutic potential for these AhR agonists without further biological evaluation.

Finally, several minor flaws: an improper bond is presented in the bottom right of Figure 1. Meanwhile, the commas in the EC50 values in Figure 5f should be revised.

Author's Response to Peer Review Comments:

Dear Editor,

Please find attached a point-by-point response letter to the reviewers' comments.

We have also addressed all the points concerning editorial issues and have uploaded the revised Manuscript and Supporting information files (both clean copies and with tracked changes).

Best regards,

Ouldouz Ghashghaei, PhD (she/her)

Laboratory of Medicinal Chemistry

Faculty of Pharmacy and Food Sciences

University of Barcelona

Website: https://www.ub.edu/medicinalchemistrypharmacology/

E-mail: ghashghaei@ub.edu

Phone: (+34)934024532

A Multicomponent Reaction-Based Platform Opens New Avenues in Aryl Hydrocarbon Receptor Modulation

(Point-by-Point Responses to the Reviewers' Comments)

The authors *sincerely thank the editors and the reviewers for their constructive feedback, comments, and suggestions. We have addressed all points of concern raised during the peer-review process and have modified the Manuscript and Supporting Information accordingly. We believe that the clarity, readability, and consistency of the manuscript have improved. We strongly hope the revised version would be considered suitable for publication in ACS Central Science.*

Formatting Needs and Editorial Considerations:

Author List: Please include the email address(es) of the corresponding author(s) on the first page of the manuscript.

Authors: *The email addresses of the corresponding authors have been added next to their respective affiliations.*

Supporting Information: Please add a full header to the top of the file designated "Supporting Information for Publication." Provide the title (in title case), authors' names, and affiliations on the top of the first page, matching those of the manuscript file exactly.

Authors: *The first page of Supporting information has been modified.*

Synopsis: ACS Central Science requires a brief synopsis. The synopsis should be no more than 200 characters (including spaces) and should reasonably correlate with the Table of Contents (TOC) graphic. The synopsis is intended to explain the importance of the article to a broader readership across the sciences. Please place your synopsis in the manuscript file after the TOC graphic.

TOC Graphic: Include a TOC graphic illustrating the significance of the paper. The TOC graphic should be something that is representative of your entire work. Color schemes or illustrations typically make good choices. TOC graphic must be original and free from any copyright issues. Confirm that all text is legible. Present the TOC graphic on the last page of the manuscript by itself. Please label the TOC as "TOC Graphic". A caption describing the TOC is not needed.

Authors: *A TOC graphic and a synopsis have been added in the last page of the manuscript.*

Moreover, during the revision process, some typos have been corrected and some of the funding in the Acknowledgement section has been revised and modified. The "tracked changes" version shows said modifications.

Reviewer 1

Comments:

The aryl hydrocarbon receptor (AHR) is a crucial chemosensory protein and an emerging therapeutic target for the treatment of various diseases, including cancers, metabolic disorders, and inflammatory conditions. Ghashghaei and colleagues have conducted remarkable organic synthesis work to establish synthetic pathways for creating derivatives (referred to as 6-ICZ derivatives) of the natural AHR ligand (FICZ), utilizing a multicomponent reaction approach. These derivatives could lead to potential therapeutic molecules (activators, inhibitors or degraders) or pharmacological tools (e.g. fluorescent probes). They have used a multidisciplinary approach combining chemical synthesis, molecular modeling, docking, molecular dynamics simulations or cell-based assays to characterize the activity of their compounds. The work was conducted in a thorough and highly rigorous manner. As a non-specialist in organic chemistry, I find the approach both original and promising for the development of new AHR receptor modulators. While the current work on bifunctional molecules has not yet led to the identification of effective inhibitors (such as degraders or antagonists), the protocols for synthesizing such molecules are established. Further research will be necessary to enhance the efficacy of the compounds, particularly by optimizing the length and composition of the linkers. On the other hand, one of the compounds obtained appears to have anti-inflammatory properties superior or at least equal to those of Tapinarof, the only FDA-approved AHR-based drug to date.

Authors: *Thank you for your appraisal.*

I have relatively minor comments:

- P2, L12-15: The sentence is unclear.

What kind of screening is being referred to, experimental or in silico screening? Why is screening being contrasted with structure-guided rational design?

It seems that both approaches are necessary and should be combined. When structural information on AHR is introduced at the start of a sentence, the authors should reference recent experimental studies instead of, or in addition to, modeling work.

Authors: *Thank you for the comment. It was not our intention to contrast the two methodologies, and we agree both approaches are necessary. To avoid confusion and to better illustrate the point, we have reshaped the entire paragraph, including the new references* ***17-19****. Also, reference* ***53*** *in the original manuscript is now reference* ***12****:*

"Finally, AhR-based drug discovery mainly relied on functional screening of compound libraries. In recent years, thanks to the recent advances in AhR structural biology,[12-16] relevant reports have tackled the development of improved ligands via a combined biological screening approach and structure-guided rational design.[17-19] Nevertheless, AhR-based therapies are still underdeveloped, and the bacterial metabolite tapinarof is the only FDA-approved AhR ligand to date (psoriasis treatment, 2022, Figure 1b).[20]"

- In several instances (P8, L59; P9, L9) the authors inappropriately mention X-ray structures instead of cryo-EM structures.

Authors: *Thank you. They have been corrected along the text.*

- P9, L5: the term "overlap" is confusing and appears not appropriate in the context in my view.

Authors: *Thank you. The phrase "overlaps with" has been substituted by "**stacks against**".*

- For certain compounds (e.g., 5a, 5b, 5c, 5g, 5l), significant discrepancies are observed between their efficacy in reporter gene assays (Fig. S26) and their ability to induce endogenous gene expression (e.g., CYP1A1) (Fig. 5g). Do the authors have an explanation for this observation?

Authors: *We thank the reviewer for this critical comment. Please note that the reporter gene assay is based on human HepG2 hepatoma cells, whereas the gene expression (qPCR) data are derived from human HaCaT keratinocytes. A reason for the mentioned discrepancies might be that even minor structural alterations may change the affinity of xenobiotic-metabolizing enzymes and/or drug transporters toward the different test compounds. Cell-specific differences in the expression/activity of the respective enzymes may thus account for differences in compound kinetics. Moreover, comparisons between two AHR ligand screening assays, here reporter gene assay (isolated XRE elements) vs. qPCR analysis (full gene promoter), frequently exhibit differences in induction strength. Please see the following references in this regard:*

- ***Reporter gene assays vs. CYP1A enzyme activity assay**s (doi: 10.1371/journal.pone.0074917)*
- ***Reporter gene assays vs. gel retardation assays** (doi: 10.1093/toxsci/55.1.107)*
- ***Reporter gene assays vs. gene expression analysis** (doi: 10.1016/j.jhazmat.2007.07.022)*

- P11, L19, 20: It's unclear why the authors associate the agonist properties of their compounds with the potential to create bifunctional AHR probes based on this activity. The key criterion in this case is receptor binding capacity, not whether the compounds are agonists or antagonists.

Authors: *We agree with the reviewer and thank them for highlighting this point. Given that we do not have AhR binding data for these compounds, we chose to take our computational evidence and their effect on receptor activity as indications for AhR engagement. To more precisely convey this point, we have modified the aforementioned section in the manuscript as follows:*

*"This computational and experimental evidence suggests considerable engagement with AhR and confirmed the suitability of adduct **5m** as a conjugatable AhR-ligand for the development of bifunctional AhR probes."*

- Some of the newly synthesized ligands seem to behave more like partial agonists or even antagonists. This aspect is not addressed in the manuscript, and the compounds are not tested for their antagonistic activity. When combined with docking and MD simulations, this could have led to an interesting study on structure-function relationships. However, this may be beyond the scope of the current manuscript.

Authors: *We thank the reviewer for this important comment and also believe investigating this point would open interesting therapeutic opportunities for AhR modulation.*

While we agree with the interpretation, we find it difficult to comment on partial agonism without having a readout for AhR binding. As the compounds were tested in cellular assays, we cannot exclude poorer binding, off-target engagement, or cell permeability which would ultimately phenotype partial agonism.

*Incidentally, we did some co-treatments with two of our compounds (**4b** and **9b**) and **BaP** and observed synergistic effects on AhR-dependent reporter gene activity. However, we prefer not to include this preliminary data in the current manuscript and plan to perform a more detailed*

study. We agree with the referee that these studies are beyond the scope of the present manuscript and could be addressed in the future.

- P11, L32: The compounds discussed in the main text (12b-d) and in Figure 6f are different.

***Authors**: Thank you. It has been corrected.*

Reviewer: 2

Comments:
In the submitted manuscript, Rodriguez et al. take inspiration form their recent work (ref 27) to develop a powerful Yonemitsu-type tricomponent reaction combining indole 2-carboxaldehydes with diverse nucleophiles to yield a series of 6-substituted indolocarbazoles. The chemistry work encompasses the reaction synthetic development, experimental reaction mechanism and computational structural studies, as well as post-functionalization of the primary adducts and extension of the reaction to several cellular probes. It is nicely completed with biological studies aiming at evaluating the activity of the compounds as aryl hydrocarbon receptor (AhR) agonists and proposed degraders with anti-inflammatory behavior through molecular docking and cellular experiments. The article is very clearly written and illustrated, making it particularly straightforward to read. The rational and relevance of the approach is well-exposed and easy to follow.

***Authors:** Thank you for the appraisal.*

It inspires several minor comments though.

The remark on the lack of reactivity of isomeric indole 3-CHO vs 2-CHO (p4, line 45) is surprising and one can wonder what was expected since the nucleophilic reactivity of the 3-position is hampered. The structure of the compounds expected from this indole 3-CHO precursor could thus be explained: how could it give compounds 4/5 as indicated in Fig S3?

***Authors:** Thank you. The MCR with indole-3-CHO could, in principle, lead to the same compounds 4-5, as the free C2 position could cyclize to give the indolocarbazole scaffold, although we never detected them experimentally. Although at reduced rates, 3-substituted indoles suffer electrophilic substitutions at C2 position. Also, indole 3-CHOs react as electrophiles in the formyl group. To clarify this point, we included indole 3-CHO in our charting studies.*

*To address the reviewer's comment, **Figure S3** has been modified accordingly to show the putative mechanism, and the following phrase was added to section **3.2.2. Scope Limitations:***

"A likely explanation could be the lower nucleophilicity of indole C-2 (vs. C-3), together with the lower electrophilicity of the formyl group at indole C-3 in comparison when it is located at C-2, taking into account its pronounced vinylogous amide character in the former case."

Regarding the proposed key intramolecular electrophilic cyclization step (conversion of intermediate II to II, Fig 2c), proceeding in acidic or basic medium. The Supp Info indicates "a neutral conjugated moiety suffering the attack of an ionized indole nucleophile" operating under basic conditions. The electrophilic species involved, potentially resulting from a dehydration, would gain to be indicated, at least in the Supp Info.

***Authors:** Thank you for the insightful suggestion. We have included an additional section and figure in the supporting information (**Section 3.3.2** and **Figure S11**). We have explicitly explained*

*the putative mechanisms leading to compounds **4-5** under acidic and basic conditions, including more detailed key species likely participating in the electrophilic cyclization step.*

This new section has been mentioned in the main text as follows:

"For a more detailed discussion of the reaction mechanism, see SI Section 3.3.2."

The formation of a cationic intermediate may be considered in acidic medium and a prior report on a related process could be cited (Studies on the Acid-Catalyzed Dimerization of 2-Prenylindoles, Tetrahedron 1996, 52, 9455-9468, 10.1016/0040-4020(96)00482-6).

***Authors:** Thank you for mentioning this important reference. It is now cited in the main text as (New Ref **47**) as follows:*

"Interestingly, a precedent work reported the formation of trace amounts of a 6-ICZ adduct from the acid-catalyzed dimerization of an allyl carbinol.[47]"

*Furthermore, we conducted a similar experiment to examine this pathway and observed analogous results to those reported by Lee et al.: the self-condensation of carbinol **1b** under acidic conditions resulted in a highly complex mixture. We detected the formation of the 6,12-ICZ adduct and trimeric species, both in small amounts. However, the 6-ICZ compound, which they isolated in trace amounts, was not observed in our case.*

However, how this cyclization gives rise in basic conditions to 6-ethoxy substituted compound 8 (Fig 2c), 6-unsubstituted ICZ (Fig S6d) and 5g,n-p (S9b), could be commented.

***Authors:** Thank you. The detailed putative reaction mechanism under basic conditions, including key species in the cyclization step, has been further commented on in the Supporting Information (**Modified Section 3.3.2** and **Figure S11**).*

*Moreover, the added section and figure also comment on the formation of compounds **8**, **S8** (**ICZ**), and **5g,n-p**:*

- *As for compound **8**, its generation has been observed under both acidic and basic conditions. We believe it occurs as a competitive pathway to that of 6-ICZs, as its formation is more pronounced when using weaker nucleophiles (see Section 3.3.1).*
- *Moreover, the formation of compound **S8** (the unsubstituted **ICZ**) could result from the reduction of a diol intermediate, mediated by the ethoxide anion. Two references have been added in the Supporting Information (Refs **3-4**) that support this hypothesis.*
- *Lastly, regarding the formation of compounds **5g** and **5n-p**, we propose that the corresponding carbinols **Ia-d** act as a 2-substituted indole nucleophilic species, and thus the formation mechanism does not differ from the other indole nucleophiles **3**.*

Another point is the control experiments with preformed alcohol precursors Ia-d (Fig2d). They were found to evolve intermolecularly with a second equivalent of indole 2-CHO instead of cyclizing intramolecularly. However the relevance of these models may be discussed: the observed reaction pathway with such preformed carbinols may not be directly compared with that of the standard one since the expected domino process have not been observed either under normal conditions with this substituents (H, Bu, allyl, 4-F-Ph).

***Authors:** Thank you for highlighting this point. We agree with the referee that the synthesized precursors **Ia-d** are not the exact representatives of the **carbinols I** suggested as the reaction intermediates. However, as mentioned in the manuscript and Supporting Information, we could*

*neither synthesize nor detect the exact replicates (with nucleophiles **2**-**3**) after numerous trials. Thus, we intended to investigate the behavior of the closest possible carbinols that we could synthesize.*

Moreover, apart from their mechanistic relevance, these precursors could have theoretically provided valuable synthetic access to non-symmetric 6-ICZs and further variations on the C-6 position. Therefore, we believe their inclusion in the studied scope has been worth the effort.

The study of the atropoisomerism of compounds 4 and 5 is interesting. It should be noted however that the structure of conformer M2' in Fig4c is incorrect, the current structure actually represents M1, and not the enantiomer of M2, as it should. Besides, why not simply naming these two atropoisomers M2 and ent-M2 ?

Authors:** Thank you. The structure of M2' has been corrected in Figure 4c. Also, following the recommendation of the reviewer, the names of the atropoisomers have been changed to more accepted notations: **M2** and **ent-M2.

Regarding the biological aspects, the docking experiments give an interesting view of the ligands binding mode and its similarity with that of the known binder indirubin. It is however not really convincing to explain the good activity of the N,N'-Me analogues 4d and 5j since a key interaction with Q383 is lost.

***Authors:** We thank the reviewer for this important comment. At first sight, the good activity of the N,N'-Me analogues **4d** and **5j** is seemingly surprising, but this may be justified according to the following considerations.*

I. *It is true that the hydrogen-bond (HB) between the ligand and Q383 is lost, but Q383 forms another HB with S365 and the HB network with T289 and H291 is preserved.*
II. *Although the contribution of a HB depends on the physicochemical features of the local environment, its contribution has been estimated to be 1.0-1.5 kcal/mol, as noted by Nick Pace et al. (FEBS Lett. 2014, 588, 2177). Notably, this work also remarked that this contribution is similar to the gain in stability observed for the burial of a CH_2 group upon protein folding (about 1.1 ± 0.5 kcal/mol).*
III. *Finally, it has been shown that the binding affinity is largely driven by hydrophobicity (Nat. Biotech. 2007, 25, 71). In this context, the insertion of the pentacyclic ring in a highly hydrophobic area is expected to be the major component of the binding affinity.*

*Taken together, these considerations provide a basis to justify the good activity of the N,N'-methylated analogues **4d** and **5j**. To better illustrate this point, the following sentence has been added to the main text, including the aforementioned references (New Refs **59,60**)*

"In this regard, the cost of losing said HB may be counterbalanced by the gain in stability due to the burial of the methyl group in the hydrophobic cavity,[59] taking into account that the binding affinity of drug-like compounds is largely driven by hydrophobicity.[60]"

A data that is puzzling is the very good activity of the 6-ethoxy substituted compound 8, suggesting that the indolyl moiety is not significantly contributing to the activity profile, while reinforcing the similarity with indirubin. In this regard, structural variations of 8 using substituted alcohols would be welcome.

***Authors:** We thank the reviewer for remarking on this issue. Indeed, the indole ring is located at the mouth of the binding cavity, forming a partial stacking interaction with the imidazole ring of*

*His291. Although the presence of the indole ring does not impede the formation of hydrogen bonds between the ligand and the protein (Figure **5c-d**), it is not expected to make a significant contribution to the binding affinity, which in turn is mainly determined by the burial of the pentacyclic ring into the apolar cavity. This opens the possibility to explore the replacement of the indole ring by chemical fragments that offer alternative anchoring points for derivatization.*

In this regard, the 6-ethoxy-ICZ has only been generated through a side reaction with the solvent of the developed MCR. We agree with the reviewer that the incorporation of other alcohols at the C-6 position would be interesting, both from a synthetic and structural perspective. Indeed, optimizing the formation of the 6-ethoxy adduct as well as broadening the alcohol scope is currently a matter of investigation in our laboratories.

Besides, although molecular dynamics does not indicate a clear a binding gap between enantiomeric M2 and M2' species, it would be interesting to chromatographically resolve the two atropoisomers of 5e and test them separately. Enantiomeric identity of drugs can have a favorable impact not only of target engagement but also on their overall pharmacological profile.

***Authors:** This is an interesting remark. The similar EC_{50} values determined for **5c** (H at indole C2; EC_{50} = 0.36 μM) and **5e** (Me at indole C2; EC_{50} = 0.52 μM) suggest that the two atropoisomers of this latter compound may possibly bind following the poses shown in Figure 5c-d, where binding involves the formation of a HB with S365 and Q383, respectively. In light of the binding modes proposed for **5c** (Figures 5c and 5d, respectively), binding of **5e** and **ent-5e** would require a slight displacement of the side chain of Y322, which is located at the edge of the pocket, to accommodate the methyl group, leading to similar binding affinities. Indeed, this provides a rationale for the limited (ca. 3.2-fold) decrease in EC_{50} observed for the trimethylated derivative **5j** (EC_{50} = 1.20 μM) compared to compound **5c**.*

On the basis of these comments, considering that the C-2 substituted derivatives do not exhibit an increased potency with respect to the C2-H compounds, we believe that the separation of enantiomers can be postponed for the development of more promising derivatives.

Nevertheless, to better illustrate the impact of C-2 substitution in the binding mode of the 6-ICZs with AhR, the following sentence has been added to the main text:

"Finally, the proposed binding mode supports the slight decrease in activity of the indolyl C-2 substituted derivatives due to the proximity to the side chain of Y322 (Figures 5f-g and S27)."

*Also, a new figure and a brief explanation were added to the supporting information (New Figure **S27**):*

*"The proposed binding mode suggests that the enantiomeric atropoisomers of **5e** and **5j** may bind with similar affinities mimicking the poses shown in Figure 5c-d. This is supported by the slight decrease in potency observed upon methylation at position C2 of the indole ring, as noted in the 1.4– and 3.2–fold increase in the EC_{50} values determined for compounds **5e** and **5j** relative to **5c** (Figure 5f). This would enable the methyl group at the indole ring to be easily accommodated upon a slight displacement of the side chain of Y322 (Figure S27)."*

The design of the probes is also interesting. The activity retained by the model compound 12a is however surprising since one could have expected an unfavorable effect of the lipophilic appendage exposed to the water environment.

***Authors:** Thank you for pointing this out. Let us remark that the aliphatic chain in compound **12a** is at large extent occluded from bulk solvent, since it is filling a pocket shaped by hydrophobic residues, such as the aliphatic chain of Lys292, Leu293, Ile341, Leu369, and Tyr371. To better illustrate this point, the aforementioned residues have been highlighted in **Figure 6b** and the following sentence has been added to the main text:*

*"…as the alkyl chain protrudes into the bulk solvent **filling a hydrophobic gorge formed by the apolar residues L293, I341, L369, and Y371, and the methylene chain of K292**, without interfering…"*

The rational for inactivating the protein by inducing its dimerization should be substantiated by literature references and the effect of 12c,d commented.

***Authors:** We thank the reviewer for this important comment. Modulation of protein activity by chemically induced homodimerization or oligomerization has been employed as a strategy previously (e.g. BCL6 PMID: 33208943, FKBP12 PMID: 7694365, PMID: 8774884). We agree that this strategy was not explained in sufficient detail in the manuscript. To better illustrate this point, we have modified the manuscript as follows and added the aforementioned references (new Refs **67-69**):*

"The modulation of protein function through small molecule induced homo-dimerization or higher-order oligomerization has been demonstrated to be an effective strategy across various examples.[67-69]"

One can have a naive comment regarding the relevance of the PROTAC strategy: if the natural destiny of Ahr is to be degraded after its activation (Fig1a), how to distinguish between normal versus PROTAC-induced Ahr degradation, in particular when no ligase seems to be clearly identified. This would incidentally also explain the degrading activity of the dimeric compounds 12c,d that may (solely) act as multivalent binders?

***Authors:** Thank you for this important comment. We do agree that for the assessment of the molecules **12b-d,** distinguishing agonist-driven degradation of AhR via its natural mechanism vs. PROTAC-driven degradation will be a challenge.*

Presumably, whereas the PROTAC-driven degradation should lead to a dramatic decline in the AhR response (e.g., CYP1 message) due to ongoing degradation of newly synthesized AhR protein, the "degradation-upon-activation" allows re-synthesis and accumulation of some AhR protein in the cytoplasm which then can be again activated by ligand-binding. This explains why upon treatment of cell cultures with metabolically stable AhR ligands, such as TCDD, the CYP1 gene expression is maximally induced for several days.

Furthermore, as we do not precisely know the degron/binding interface of the agonist-driven AhR degradation, one would have to optimize PROTAC candidates to degrade AhR without modulating its transcriptional activity. Then, to definitively map the mechanism of degradation, one would have to measure AhR levels under treatment in wild type and genetic deletions for the recruited E3 ligase (PMID: 32494016). This was unfortunately out of scope for this particular study and could be an interesting future development primed by the synthetic platform shown here.

Overall, the submitted article is particularly dense in hypotheses and design as well experimental and theoretical results yielding uncommon molecules with promising biological profiles (although water solubility and metabolic stability issues should be considered for further

development) toward a validated target protein. It deserves to be published in ACS Central Science after consideration of the above-mentioned minor comments.

Authors: *Thank you for your appraisal. We agree that further development of the hit compounds would need additional assessment, which will be addressed in an ongoing project.*

Reviewer 3

Comments:
This manuscript describes the development of a rewired Yonemitsu multicomponent reaction to afford tunable 6-substituted indolocarbazole derivatives as potent AhR modulators with promising anti-inflammatory properties. Systematic characterization of the scope and mechanism of the rewired Yonemitsu MCR was performed, and further structural modification also enriched the chemical diversity of the 6-ICZ derivatives. Preliminary biological evaluation identified favorable safety and anti-inflammatory property of compound 8.

However, there are several issues that need to be illustrated before ready for publication.

Firstly, several bifunctional AhR probes are presented in Figure 6, exhibiting comparable AhR downregulation effect to compound 8, however, their anti-inflammatory properties have not been evaluated.

Authors: *We thank the referee for highlighting this point. For the proof-of-concept anti-inflammatory assessments, we chose the most representative analogs of each sub-family not only in terms of EC_{50} measurements, but also in gene expression (qPCR) essays. Thus, we chose compounds **5b**, **5h**, **8** and **10**. Our developed bifunctional probes did not exhibit increased AhR-activating properties compared to the selected compounds (Figures S28 and S29), while having much higher molecular weights and lower solubilities. Thus, we excluded them in the first round of screening.*

Is there any special application for these probes? The purpose of this modification strategy is not clear enough. Please comment on this.

Authors: *We appreciate the reviewer's inquiry. Our aim was to develop bifunctional molecules capable of modulating AhR activity through induced proximity. We utilized docking studies to identify an appropriate exit vector in our molecular design. Although we cannot yet showcase clear effects from the potential induced proximity, we believe our developed platform provides a solid foundation for future investigations in this direction. Especially important in this regard are the developed computational model and the streamlined synthetic methodology, which can be utilized in the future to adjust the characteristics of the linker (suitable length, chemical structure, etc.) for more optimized proximity induced probes.*

*As mentioned above to reviewer 2, in order to better illustrate the purpose of this strategy, we have modified the manuscript and added new Refs **67-69.** The full paragraph now reads as follows:*

*"This computational and experimental evidence suggests considerable engagement with AhR and confirmed the suitability of adduct **5m** as a conjugatable AhR-ligand for the development of bifunctional AhR probes through two different strategies. In a first approach, we linked thalidomide-PEG-amine to **5m** to conveniently obtain **12b** (78%), which bears thalidomide, a ligand of the E3 ubiquitin ligase CRBN.[64] Such bivalent molecules have been termed PROteolysis Targeting Chimeras (PROTACs) for their ability to induce targeted protein degradation (TPD) in*

*cells.[65,66] For our second approach, we hypothesized that dual AhR ligands could elicit their inhibitory effect by forming an inactive homo-dimer and synthesized the bivalent species **12c** (36%) and **12d** (71%) in a single step from unprotected diamine-type linkers (Figure 6c). The modulation of protein function through small molecule induced homo-dimerization or higher-order oligomerization has been demonstrated to be an effective strategy across various examples.[67-69]"*

Secondly, additional biological comparison with FICZ is recommended to highlight the bio-safety of its derivatives in this study.

Authors: *We thank the reviewer for this critical comment. The most prominent adverse effect of FICZ treatment is probably its pronounced phototoxicity in response to UVA radiation. Accordingly, we decided to compare the phototoxic potential of a subset of our compounds with the UVA phototoxicity of FICZ. In contrast to FICZ, none of the indolocarbazole compounds enhanced UVA radiation-induced caspase activity at a concentration of 100 nM. Two of the test compounds, however, exhibited similar effects to FICZ when testing them in a concentration of 1 µM. The data are shown in a new section in the supporting information (Section 7.2), including the new Figure S36.*

*Moreover, we have added the following paragraph to the manuscript, including the new references **61** and **62**.*

*"Furthermore, we tested the potential phototoxicity of our 6-ICZ derivatives, as FICZ is a nanomolar sensitizer for UVA radiation.[61,62] In contrast to FICZ, none of the selected compounds showed any signs of phototoxicity at a concentration of 100 nM, similarly to FDA-approved tapinarof. Notably, some of the our most potent 6-ICZs such as compounds **8** and **10** showed minimal overlap between their effective and toxic concentrations, the latter – our closest structural analogue to FICZ – even exhibiting no phototoxicity at 1 µM (Figure S36). Altogether, these results showcase the potential of our synthetic platform to obtain safe and potent AhR activators."*

Moreover, the current manuscript presents limited therapeutic potential for these AhR agonists without further biological evaluation.

Authors: *We thank the reviewer for this valuable comment. As mentioned above, the present manuscript primarily focuses on the development of a unified platform to tackle different aspects of AhR-based drug-discovery through an innovative approach. In this way, we have developed non-cytotoxic and potent AhR agonists. Moreover, we have included initial proof-of-concept experiments to display their anti-inflammatory activity.*

We completely agree with the reviewer that additional biological evaluation would be needed to further explore their therapeutic potential. In fact, we are already planning to study potential therapeutic applications of some of the synthesized indolocarbazoles in contexts where AhR agonism is beneficial. These studies are beyond the scope of the current manuscript. However, they will be addressed through the tools provided within the framework of our developed platform.

Finally, several minor flaws: an improper bond is presented in the bottom right of Figure 1. Meanwhile, the commas in the EC50 values in Figure 5f should be revised.

Authors: *Thank you. Figure 1 has been corrected and the commas in the EC50 values in Figure 5f have been changed to periods.*

oc-2025-00194y.R2

Name: Peer Review Information for "A Multicomponent Reaction-Based Platform Opens New Avenues in Aryl Hydrocarbon Receptor Modulation"

Second Round of Reviewer Comments

Reviewer: 3

Comments to the Author

I think the authors have addressed almost all the critiques raised by the reviewer and the manuscript was improved properly, in my opinion, the revised manuscript can be accepted for publication in ACS Central Science.

Reviewer: 2

Comments to the Author

The authors have systematically taken into consideration my comments with care, detail and precision, in the article text as in the SI, therefore significantly clarifying the manuscript and upgrading its quality. The revised version of the later should thud be published as it stands now in ACS Central Science.

Author's Response to Peer Review Comments:

Dear Editor,

We have addressed all the requested modifications as follows:

All the supporting files mentioned in the text, are labeled "as for publication".

The checkCIF files and the .CIF files of the crystals were removed. They have been uploaded on the CCDC webpage and their CCDC numbers are mentioned in the text.

Supporting Information page numbers have been modified to S1, S2, S3, etc.

The synopsis has been shortened to 200 characters and it reads as follows:

"A multicomponent reaction platform yields 6-substituted indolocarbazoles as potent, safe, and modular activators of the Aryl hydrocarbon Receptor (AhR), opening new avenues in AhR research."

TOC Graphic has been labeled.

Finally, we would like to dedicate this paper to a late colleague if possible. We would like to add the following phrase: "Dedicated to the memory of Prof. Antonio Delgado (University of Barcelona)" if ACS Central Science supports such dedicatory phrases.

Thank you for handling our manuscript along the process.

Best regards,

Ouldouz Ghashghaei, PhD

Laboratory of Medicinal Chemistry

Faculty of Pharmacy and Food Sciences

University of Barcelona

E-mail: ghashghaei@ub.edu

Phone: (+34)934024532
